# Investigation of the Functional Components in Health Beverages Made from *Polygonatum cyrtonema* Rhizomes Provides Primary Evidence to Support Their Claimed Health Benefits

**DOI:** 10.3390/metabo14070376

**Published:** 2024-07-03

**Authors:** Qiyan Song, Youwu Chen, Ye Shao, Weiting Pu, Bihuan Ye, Xiaoxiao Shi, Jianjun Shen, Haibo Li

**Affiliations:** 1 Zhejiang Academy of Forestry, Hangzhou 310023, China; zjlktg@163.com (Q.S.);; 2 School of Forestry and Biotechnology, Zhejiang Agriculture and Forestry University, Hangzhou 311300, China

**Keywords:** beverages, functional components, health benefits, medicinal plant, Chinese liquor, metabolomics

## Abstract

This study aims to understand the functional component compositions of traditional herbal health beverages made from *Polygonatum cyrtonema* rhizomes and to reveal the pharmacodynamic chemical basis for their claimed health benefits. Two traditional methods, rhizome decoction and rhizome infusion, were used to make health herbal beverages, including “*Huangjin*” tea and “*Huangjin*” wine, respectively. The secondary metabolites of “*Huangjin*” beverages were investigated and compared by widely targeted metabolomics. The results clearly showed that the major functional components in “*Huangjin*” beverages were phenolic acids, flavonoids, and alkaloids. The “*Huangjin*” wine has a greater variety of flavonoids and alkaloids than “*Huangjin*” tea, and the functional components in “*Huangjin*” wine were more abundant than those in “*Huangjin*” tea. Homoisoflavones and amide alkaloids were the dominating flavonoids and alkaloids in “*Huangjin*” wine, respectively. Continuous rhizome infusion could not increase the content of functional components in “*Huangjin*” wine. In conclusion, this study not only provides primary evidence to support the claimed health benefits of “*Huangjin*” beverages but also suggests that making traditional herbal beverages by rhizome infusion has superior health benefits than making them by rhizome decoction, which is attributed to the higher yields of functional components extracted by Chinese liquor than hot water. Therefore, Chinese liquor shows advantages in its use as a superior binary ethanol–water solvent in making herbal health beverages to enhance the solubility of poorly water-soluble functional components.

## 1. Introduction

Most species in the genus *Polygonatum* are popular medicinal plants and belong to the Asparagaceae family. The species *P. cyrtonema* Hua (“Duohua *Huangjin*” in Chinese) was recorded in the Chinese Pharmacopoeia and has a long application history in Chinese folk medicine [1,2]. The processed *P. cyrtonema* rhizomes, as an important edible food and medicinal resource, have been widely used as materials to manufacture herbal health food and healthcare medicines; they have been used in the treatment of cough, diabetes, hypertension, chronic hepatitis, and cardio-cerebrovascular diseases for clinical needs [3]. The reason is that *P. cyrtonema* rhizomes are abundant in functional medicinal components, including polysaccharides and secondary metabolites such as saponins, flavonoids, and alkaloids [4,5]. In recent years, developing natural functional food has received increasing attention along with the growth in the living standards of Chinese people. This situation has led to an increasing number of health herbal foods made from *P. cyrtonema* rhizomes, such as “*Huangjin*” health tea, “*Huangjin*” health wine, and “*Huangjin*” health candy, in Chinese folk culture and on the health product market.

Directly eating unprocessed *P. cyrtonema* rhizomes (crude medicine) can lead to adverse reactions, including throat irritation and numbness of the tongue [6]. Therefore, the crude medicine of *P. cyrtonema* is usually processed with a traditional method called “nine-steaming and nine-drying” (steaming and drying it nine times) to reinforce its efficacy and reduce its toxicity [7]. Moreover, the extraction methods of water decoction and Chinese liquor infusion are an integral part of traditional Chinese medicine and essential for their widely acknowledged efficacy [8,9]. In Chinese folk medicine, the processed *P. cyrtonema* rhizomes are usually eaten directly as “*Huangjin*” health candy or used as food material to make health beverages, including “*Huangjin*” health tea by rhizome decoction with water or “*Huangjin*” health wine by rhizome infusion with Chinese liquor. The functional medicinal components with biological activities are endowed with the healthy function of the *P. cyrtonema* rhizomes. However, whether these “*Huangjin*” health beverages made from the processed rhizomes contain large amounts of functional medicinal components which can support their claimed health benefits is currently lacking in evidence.

Secondary metabolites, including alkaloids, flavonoids, amines, glycosides, and steroids, play an important role in the interaction of plants with their environment and have been used in the drug and pharmaceutical industries to treat various disorders [10,11,12,13]. Secondary metabolites of medicinal plants are not only the material basis of medicinal materials due to their clinically curative effects but are also important indicators for evaluating the quality of medicinal materials [14]. Accordingly, a comprehensive investigation into the secondary metabolites of traditional “*Huangjin*” health beverages is crucial to understanding their functional component compositions and revealing the pharmacodynamic chemical basis for their claimed health benefits. Metabolomics is considered to be an important functional genomics tool because metabolites are links of genotypes and phenotypes [15,16,17,18,19]. In recent years, metabolomics has been employed widely in the research fields of medicinal plants, biological interaction, fruit nutritional quality, and biological activity [20]. In our present study, we performed metabolomic analysis to investigate the functional component compositions of “*Huangjin*” health beverages and initially identify the differentially extracted secondary metabolites between “*Huangjin*” health tea and “*Huangjin*” health wine. Our objectives were to understand the functional component compositions of traditional health beverages made from *Polygonatum cyrtonema* rhizomes and to reveal the pharmacodynamic chemical basis for their claimed health benefits. Additionally, our study will also verify whether the traditional concept that the quality of the herbal wine is better when it has been stored longer is reasonable.

## 2. Materials and Methods

### 2.1. Selection, Pretreatment, and Processing of Plant Materials

Nine fresh, healthy, and five-year-old *Polygonatum cyrtonema* Hua. plants were collected from plantations in Quzhou (26°339200 N, 104°459500 E; altitude of 2022 m), Zhejiang Province (Southeast China), in April 2023*. P. cyrtonema* rhizomes were harvested from these plants and cleaned with water. They were further cut into slices, mixed, and stored at 4 °C until further processing. The processing of the *P. cyrtonema* rhizomes followed a previously described method [21]. In brief, fresh rhizomes were placed in a steamer to steam for 6 h. Subsequently, they were moisturized overnight and then dried in an oven at 50 °C for 8 h and 9 cycles. After 9 processing cycles, these rhizomes were used as materials to make “*Huangjin*” health tea and “*Huangjin*” health wine.

### 2.2. Making “Huangjin” Health Tea and “Huangjin” Health Wine

In Chinese folk medicine, rhizome decoction is usually used to make herbal health tea by boiling rhizomes with hot water for 20–40 min, and rhizome infusion is usually used to make herbal health wine by soaking rhizomes with Chinese liquor for more than 10 days. Based on the traditional health concept and methods, “*Huangjin*” health tea and “*Huangjin*” health wine were made in this study as follows: a total of 200 g of processed *P. cyrtonema* rhizomes with three replicates was placed in a heating container, boiled with 600 mL of hot water for 3 min, and further maintained at a low boil, lasting for 40 min. After cooling, the extract was collected in a tube labeled as the BW40 group and stored at −80 °C for a short period for further analysis. Similarly, the same weight of processed rhizomes with three replicates was sealed in a glass container and soaked with 600 mL of Chinese liquor (45%, alc/vol). Then, the liquor extract was collected in a tube after 7, 14, and 21 days. The three products were labeled as the W7D, W14D, and W21D groups, respectively. They were stored at −80 °C for a short period for further analysis.

### 2.3. Sample Preparation and Extraction for Widely Targeted Metabolomic Analysis

Sample preparation and extraction were performed based on the methods provided by Metware Biotechnology Co., Ltd. (Wuhan, China). Briefly, the sample was removed from the −80 °C refrigerator, allowed to thaw until no ice remained, and vortexed for 10 s to ensure thorough mixing. A total of 100 µL of the sample was collected and added to the corresponding numbered 1.5 mL centrifuge tube. In addition, 100 µL of 70% methanol with internal standard extraction solution was added. Notably, for volumes less than 100 µL, the extraction solution was added at a ratio of 1:1 (*v*/*v*). Finally, the mixture was vortexed for 15 min at 12,000 r/min at 4 °C and then centrifuged for 3 min. The supernatant was removed, filtered through a microporous membrane (pore size of 0.22 µm), and stored in the injection vial for LC–MS/MS detection.

### 2.4. UPLC Conditions

Sample extracts of the “*Huangjin*” beverage were analyzed using an ultra-performance liquid chromatography–electrospray ionization–tandem mass spectrometry system [UPLC-ESI-MS/MS, UPLC, ExionLC™ AD, https://sciex.com.cn (accessed on 10 October 2023)]. The UPLC analytical conditions were as follows: the column was Agilent SB-C18 (1.8 µm, 2.1 mm × 100 mm); the mobile phase consisted of solvent A composed of pure water with 0.1% formic acid and solvent B comprising acetonitrile with 0.1% formic acid. Sample measurements were performed with a gradient program that employed starting conditions of 95% A and 5% B. Within 9 min, a linear gradient to 5% A and 95% B was programmed, and a composition of 5% A and 95% B was kept for 1 min. Subsequently, a composition of 95% A and 5.0% B was adjusted within 1.1 min and kept for 2.9 min. The flow velocity was set as 0.35 mL per minute, the column oven was set to 40 °C, and the injection volume was 2 μL. The effluent was alternatively connected to an ESI-triple quadrupole-linear ion trap (QTRAP)-MS.

### 2.5. ESI-Q TRAP-MS/MS

The ESI source operation parameters were as follows: source temperature of 500 °C; ion spray voltage (IS) of 5500 V (positive ion mode)/−4500 V (negative ion mode); ion source gas I and II and the curtain gas were set at 50, 60, and 25 psi, respectively; the collision-activated dissociation was high. Triple quadrupole (QQQ) scans were acquired as multiple reaction detection (MRM) experiments with the collision gas (nitrogen) set to medium. The declustering potential (DP) and collision energy (CE) for individual MRM transitions were obtained with further DP and CE optimization. A specific set of MRM transitions was monitored for each period according to the metabolites eluted within this period.

### 2.6. Metabolite Annotation

According to the reference library Metware database MWDB V2.0 (Metware Biotechnology Co., Ltd., Wuhan, China) and publicly available metabolite databases, including MassBank [https://massbank.eu/MassBank/ (accessed on 20 November 2023), HMDB (https://hmdb.ca/ (accessed on accessed on 20 November 2023)), and METLIN (https://metlin.scripps.edu/landing_page.php?pgcontent=mainPage, (accessed on accessed on 20 November 2023)], primary and secondary mass spectrometry data were subjected to qualitative and quantitative analysis by using targeted MRM. In brief, widely target metabolomics used high-resolution mass spectrometry, specifically the AB Sciex TripleTOF 6600, for qualitative detection of the mixed samples. Then, the AB Sciex 6500 QTRAP was utilized for accurate metabolite quantification. This approach combines the advantages of non-targeted and targeted metabolomics by using high-resolution QQQ mass spectrometry with high sensitivity, high specificity, and excellent quantitation capabilities.

During the process of qualitative analysis of the metabolites, repeat signals of K^+^, Na^+^, NH^4+^, and other substances with large molecular weights were eliminated according to the information on the secondary spectrum [22,23]. The quantitative analysis was conducted in the MRM mode. The characteristic ions of each metabolite were screened through the QQQ mass spectrometry to obtain the signal strengths. After obtaining the metabolite profile data of the different samples, integration and correction of the chromatographic peaks were conducted using MultiQuant v3.0.2 (AB Sciex, Concord, ON, Canada). All integral data for the chromatographic peak area were derived, and the relative contents of the corresponding metabolites were calculated as the peak area integrals [24,25].

### 2.7. Multivariate Statistical Analysis

Metabolite data were log_2_-transformed and underwent autoscaling before any statistical analysis. The metabolite data of the 12 sample extracts of the “*Huangjin*” beverages were used for unsupervised principal component analysis (PCA), hierarchical clustering analysis (HCA), and orthogonal partial least squares discriminant analysis (OPLS-DA) using Metware Cloud, which is a free online platform for data analysis [https://cloud.metware.cn (accessed on accessed on 8 December 2023)]. Briefly, unsupervised PCA was performed using the statistics function “prcomp” within R v3.5.1 [base package, www.r-project.org (accessed on 8 December 2023)]. The HCA results for the samples and metabolites were presented as heatmaps with dendrograms, while Pearson’s correlation coefficients (PCC) between samples were calculated using the “cor” function in R v3.5.1 and v4.4.0 (base package; Hmisc) and presented as heatmaps only. Both HCA and PCC were carried out using R v2.8.0 (Complex Heatmap). For the HCA, normalized signal intensities of the metabolites (unit variance scaling, UV scaling) were visualized as a color spectrum. OPLS-DA was performed using R v1.0.1 (MetaboAnalystR). Before OPLS-DA, the data were log_2_-transformed and underwent mean centering, and a permutation test (200 permutations) was performed to avoid overfitting. The OPLS-DA results also contained score and permutation plots [26].

For two-group analysis, the differentially extracted metabolites were determined by their variable importance in project scores (VIP ≥ 1) and a fold change ≥ 2 or a fold change ≤ 0.5, and the VIP values were extracted from the OPLS-DA results. Identified differential metabolites were annotated using the Kyoto Encyclopedia of Genes and Genomes (KEGG) compound database [http://www.kegg.jp/kegg/compound (accessed on 12 December 2023], and annotated metabolites were then mapped to the KEGG pathway database (http://www.kegg.jp/kegg/pathway.html, accessed on 12 December 2023). Pathways with significantly regulated metabolites were then subjected to metabolite set enrichment analysis, and their significance was determined by the hypergeometric test’s *p*-values.

## 3. Results

### 3.1. Metabolomic Profiling of “Huangjin” Tea and “Huangjin” Wine

**Figure 1:**
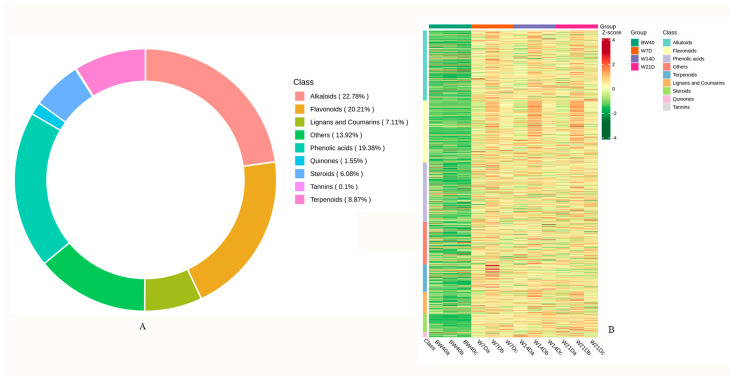
Basic information on secondary metabolites detected in two “*Huangjin*” beverages. (**A**) Quantity and classification of secondary metabolites. (**B**) Clustered heatmap of secondary metabolites in “*Huangjin*” tea samples (BW40 group) and in “*Huangjin*” wine samples (W7D, W14D, and W21D groups). BW40 represents “*Huangjin*” tea samples obtained by rhizome decoction with hot water for 40 min, and W7D, W14D, and W21D represent “*Huangjin*” wine samples obtained by rhizome infusion with Chinese liquor for 70, 14, and 21 days, respectively. The shades of color indicate the quantity of metabolites, with redder shades representing more and greener shades representing fewer.

Based on UPLC–MS/MS and the Metware database and the publicly available metabolite databases, the secondary metabolites in “*Huangjin*” tea (BW group) and in “*Huangjin*” wine (W7D, W14D, and W21D groups) were determined. A total of 970 metabolites were identified from the two “*Huangjin*” beverages, including 221 alkaloids, 188 phenolic acids, 196 flavonoids, 86 terpenoids, 69 lignans and coumarins, 15 quinones, 59 steroids, 1 tannin, and 135 others (Figure 1A, Table S1). This result indicates that the major secondary metabolites in “*Huangjin*” wine and “*Huangjin*” tea were polyphenols, including phenolic acids and flavonoids, which accounted for 39.59% of the total. Alkaloids, which are a large group of nitrogen-containing compounds with a low molecular weight, accounted for 22.78% of the total. The hierarchical clustering heatmap based on UV scaling showed that most of the secondary metabolites in “*Huangjin*” wine (W7D, W14D, and W21D groups) were more abundant than those in “*Huangjin*” tea (BW40 group), which indicates that rhizome infusion with Chinese liquor achieved higher extraction yields of the secondary metabolites than rhizome decoction with hot water (Figure 1B).

**Figure 2:**
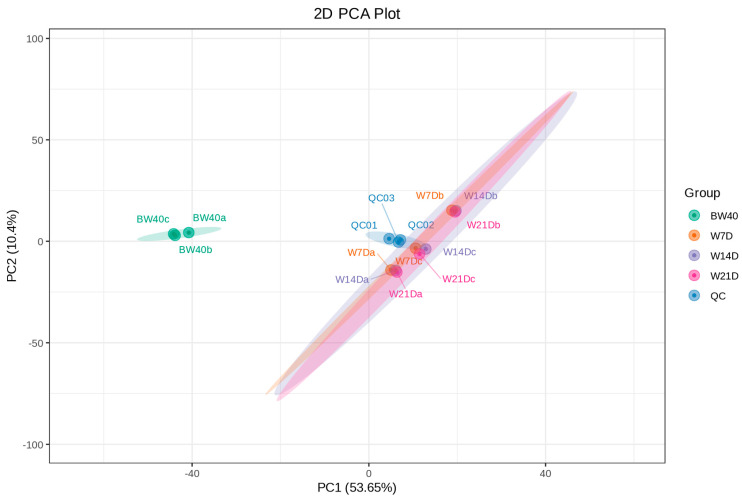
Principal component analysis (PCA) plot of secondary metabolites from 12 “*Huangjin*” beverage samples. The figure in brackets refers to the explained variance ratio. BW40 represents “*Huangjin*” tea samples obtained by rhizome decoction with hot water for 40 min, and W7D, W14D, and W21D represent “*Huangjin*” wine samples obtained by rhizome infusion with Chinese liquor for 7‒, 14‒, and 21 days, respectively. QC represents the mixture of “*Huangjin*” beverages sample extracts for repeatability test.

UV scaling and PCA were performed on 12 samples to gain insights into the differences in the overall metabolites among the four groups of samples and the degree of variability between samples within the same group. The PCA plot showed the explained variance ratios of PC1 and PC2 were 53.65% and 10.4%, respectively. Three mixed samples for QC were grouped together near the center of the PCA plot, which confirms the QC samples had similar metabolic profiles and ensured the stability and repeatability of the entire analysis (Figure 2). In addition, the correlation analysis showed that 12 samples in the same group had PCC values close to 1.0 (Figure S1), which indicates that the data from this metabolome analysis were credible and could be used for further analysis. In the PCA plot, we noticed that three replicates within the W7D group, three replicates within the W14D group, and three replicates within the W21D group were clustered together. They were completely separated from the three replicates within the BW group, which implies that the secondary metabolites within these three groups had no remarkable differences. However, the secondary metabolites between the three groups and the BW group had remarkable differences. The initial findings from PCA indicate that the abundance of secondary metabolites in “*Huangjin*” wine obtained by rhizome infusion lasting from 7 days to 21 days had no substantial changes.

### 3.2. Profiling of Differentially Extracted Secondary Metabolites between “Huangjin” Tea and “Huangjin” Wine

**Figure 3:**
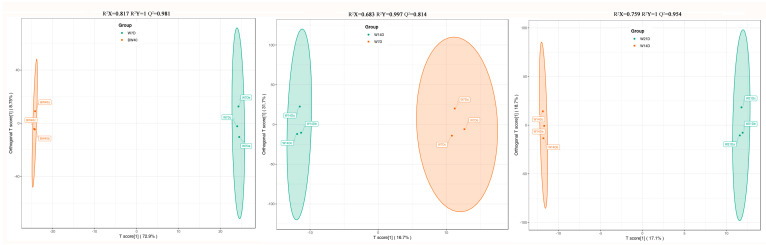
The OPLS-DA score plots of three pairwise comparisons. BW40 represents “*Huangjin*” tea samples obtained by rhizome decoction with hot water for 40 min, and W7D, W14D, and W21D represent “*Huangjin*” wine samples obtained by rhizome infusion with Chinese liquor for 7‒, 14‒, and 21 days, respectively.

OPLS-DA, as a multivariate statistical analysis method with supervised pattern recognition, can solve the insensitivity of PCA to the variables with little correlation. The score plot of each group was generated based on the differential variables to further illustrate the differences between groups. OPLS-DA was performed to reveal the separation among the BW, W7D, W14D, and W21D groups and identify significantly differential secondary metabolites among these groups. The OPLS-DA score plots for the pairwise comparisons of W7D vs. BW40, W14D vs. W7D, and W21D vs. W14D are shown in Figure 3. In the three OPLS-DA models, the values of R^2^Y and Q^2^ for three comparisons were closer to 1.0, except for the comparison of W14D vs. W7D (Q^2^ = 0.814). This result indicates that these models were reliable and suitable for the subsequent screening of differential metabolites.

**Figure 4:**
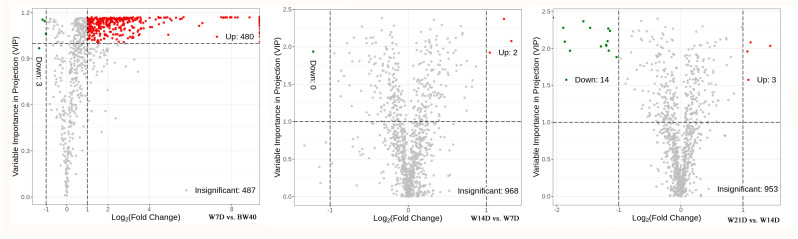
Volcano plots of differentially extracted secondary metabolites (DESMs) from three pairwise comparisons. The dots in plots represent DESMs, with red representing upregulated, green representing downregulated, and gray indicating insignificant. BW40 represents “*Huangjin*” tea samples obtained by rhizome decoction with hot water for 40 min, and W7D, W14D, and W21D represent “*Huangjin*” wine samples obtained by rhizome infusion with Chinese liquor for 7‒, 14‒, and 21 days, respectively.

The significant differential secondary metabolites were identified as differentially extracted secondary metabolites (DESMs) within each pairwise comparison based on both fold change ≥ 2.0 [log_2_ (2) ≥ 1.0] or ≤0.5 [log_2_ (0.5) ≤ −1] and VIP ≥ 1.0 as the screening standards. These DESMs identified from the three comparison groups are displayed in a volcano plot in Figure 4. A total of 483 DESMs (480 up- and 3 downregulated) were identified from W7D vs. BW40, 2 DESMs (2 up- and 0 downregulated) from W14D vs. W7D, and 17 DESMs (3 up- and 14 downregulated) from W21D vs. W14D (Table 1). In addition, of 483 DESMs identified from the W7D vs. BW40 comparison group, 159 of them were found only in the W7D group (Table 2), and detailed information on these compounds is listed in Table S2.

A large quantity of DESMs (483/970, 49.8%) were identified from W7D vs. BW40, while only a very minute quantity was found from W14D vs. W7D and W21D vs. W14D, and most of these identified DESMs were flavonoids and alkaloids. This finding indicates that drastic differences existed in the abundance of secondary metabolites between “*Huangjin*” tea and “*Huangjin*” wine. Meanwhile, continuous rhizome infusion with Chinese liquor for 7 days to 21 days did not lead to further significant changes in the abundance of secondary metabolites in “*Huangjin*” wine. Thus, as for the secondary metabolites, rhizome infusion for a longer period cannot enhance their content and then further promote the health benefits of “*Huangjin*” wine. Moreover, these secondary metabolites, mainly flavonoids and alkaloids, found only in W7D indicate that rhizome infusion with Chinese liquor extracted a greater variety of secondary metabolites than rhizome decoction with hot water. This result from the DESM analysis is consistent with that from PCA and indicates that “*Huangjin*” tea was drastically different from “*Huangjin*” wine not only in its abundance of common secondary metabolites but also in the quantity of secondary metabolites. This is due to the fact that the two extracting solvents used in making “*Huangjin*” beverages led to significant differences in the extraction yields of the secondary metabolites. The greater variety of functional components and their high contents contribute to the superior health benefits of “*Huangjin*” wine compared to “*Huangjin*” tea.

### 3.3. Variation Pattern in Relative Abundance of Differentially Extracted Secondary Metabolites

**Figure 5:**
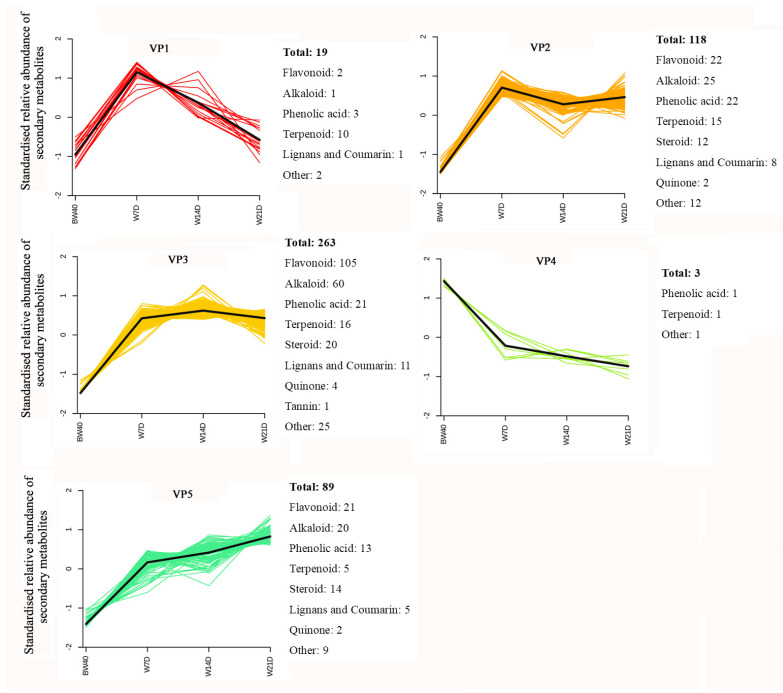
K-means clustering analysis of differentially extracted secondary metabolites (DESMs) from three pairwise comparisons. The number represents the total quantity of DESMs with the same variation pattern. BW40 represents “*Huangjin*” tea samples obtained by rhizome decoction with hot water for 40 min, and W7D, W14D, and W21D represent “*Huangjin*” wine samples obtained by rhizome infusion with Chinese liquor for 7‒, 14‒, and 21 days, respectively.

A total of 492 DESMs were subjected to K-means clustering analysis to understand the variation patterns in their relative abundance. After standardization by UV scaling, these DESMs were clustered into five variation patterns according to the changes in their relative abundance in all 12 samples (Figure 5). The clustering analysis showed that the relative abundance of most of the DESMs (470, 95.5%) clustering into variation patterns 2, 3, and 5 increased in the W7D, W14D, and W21D groups compared with that in the BW40 group. These DESMs mainly consisted of flavonoids, alkaloids, phenolic acids, terpenoids, and steroids. Obviously, variation pattern 3 was the most typical because 263 DESMs (53.46%) were clustered into this pattern, in which flavonoids and alkaloids were the most abundant DESMs. Therefore, the abundance of most of the DESMs in the “*Huangjin*” wine made by rhizome infusion with Chinese liquor for 7 days was significantly higher than that in the “*Huangjin*” tea made by rhizome decoction with hot water for 40 min. However, their abundance was not significantly increased with continuous rhizome infusion for more than 7 days, up to 21 days. In addition, a notable exception was the clustering of 10 terpenoids (diterpenoids) into variation pattern 1, which exhibited continuously decreased trends in relative abundance in “*Huangjin*” wine after rhizome decoction for more than 7 days, up to 21 days (Table S3).

### 3.4. Annotation and Functional Classification of Differentially Extracted Secondary Metabolites

**Figure 6:**
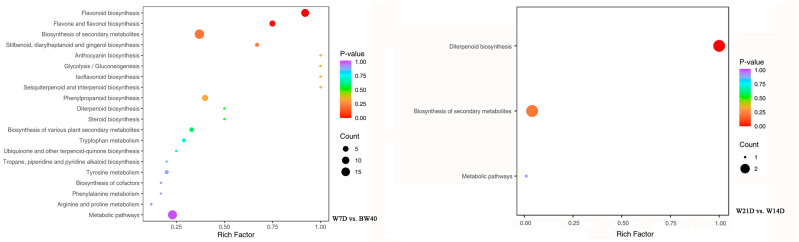
The top 20 significantly enriched KEGG pathways of differentially extracted secondary metabolites from two pairwise comparisons. The color of dots in plots refers to higher or lower *p*-values, with bluer representing higher and redder representing lower. The size of a dot represents more or less secondary metabolites, with larger dots representing more and smaller dots representing fewer. BW40 represents “*Huangjin*” tea samples obtained by rhizome decoction with hot water for 40 min, and W7D, W14D, and W21D represent “*Huangjin*” wine samples obtained by rhizome infusion with Chinese liquor for 7‒, 14‒, and 21 days, respectively.

Enrichment analysis of the KEGG pathways was performed based on all the identified secondary metabolites to understand the enriched pathways of DESMs between “*Huangjin*” tea and “*Huangjin*” wine. Among all 110 annotated secondary metabolites, 35 were DESMs from the W7D vs. BW40 comparison group, 2 were DESMs from W21D vs. W14D, and no annotated secondary metabolites were found from W14D vs. W7D (Figure 6; Table S4). In the top 20 significantly enriched KEGG pathways, all 35 DESMs from W7D vs. BW40 were involved in these pathways and upregulated, including 19 DESMs related to flavonoid biosynthesis, such as flavonoid biosynthesis (ko00941), isoflavonoid biosynthesis (ko00943), and flavone and flavonol biosynthesis (ko00944) (Table 3; Figure S2). The significantly enriched KEGG pathways in W21D vs. W14D were diterpenoid biosynthesis, biosynthesis of secondary metabolites, and metabolic pathways, in which only two downregulated DESMs were annotated. Therefore, flavonoids, as key functional medicinal components in *P. cyrtonema* rhizomes, were the most significantly variable secondary metabolites between “*Huangjin*” tea and “*Huangjin*” wine. Moreover, the flavonoids extracted by rhizome infusion with Chinese liquor are more abundant than those extracted by rhizome decoction with hot water, which contributes to the superior health benefits of “*Huangjin*” wine compared to “*Huangjin*” tea.

## 4. Discussion

The health benefits of “*Huangjin*” tea and “*Huangjin*” wine are derived from the functional components included in water-soluble extracts or alcohol-soluble extracts. According to the data reviewed by Fu et al. (2021) [27], six *Polygonatum* species (including *P. cyrtonema*) in Asparagaceae have been used as herbal teas in China, and the main preparation method used in making “*Huangjin*” tea is rhizome decoction in water. Water and Chinese liquor, as polar molecules and solvents, are popularly used in traditional herb processing and the extraction of polar compounds with biological activity. Water can enhance the efficiency of the extraction process by helping the diffusion of extractable components like polyphenols through plant tissues [28]. Alcohol can dissolve most water-soluble or insoluble substances in herbs; due to its good permeability, it enters plant tissues to promote displacement, diffusion, and dissolution of the phytoconstituents [29]. Solubilizing or extracting many functional components using solely hot water is nearly impossible due to their low solubility in this solvent. Bioactive components such as polyphenols might be poorly soluble in water but have better solubility in organic solvents [30]. In our study, the yield of functional components in “*Huangjin*” tea and “*Huangjin*” wine strongly depended on the extraction solvent: hot water or Chinese liquor (45% alc/vol). Traditional “*Huangjin*” wine is made by rhizome infusion with Chinese liquor (more than 40% alcohol), which means the functional components in “*Huangjin*” wine are extracted using a binary ethanol–water solvent. The addition of ethanol to water is predominantly conducted to reduce its polarity, which allows it to behave more like a medium- or low-polarity solvent. As a result, it can solubilize more non-polar natural products, which leads to higher extraction yields [31]. A mixture of water and ethanol could be a more successful solvent than a single solvent in the extraction of polyphenols [28]. As previously reported, ethanol (95% alc/vol) extracted nearly all the compounds that were present in the hot water extract, and ethanol extraction yielded compounds with favorable physicochemical properties; thus, they are likely to be orally available [9]. Therefore, the many more functional components, especially polyphenols, contained in “*Huangjin*” wine can be attributed to their high solubility in Chinese liquor, which is a binary ethanol–water solvent.

In this study, we found that some identified secondary metabolites, even if they belong to the same class, had different releasing behaviors in hot water and Chinese liquor. For example, among the identified DESMs, 75 flavonoids, 35 alkaloids, and 8 phenolic acids were only present in “*Huangjin*” wine, while the other 75 flavonoids, 70 alkaloids, and 51 phenolic acids were present in “*Huangjin*” tea and “*Huangjin*” wine. This finding suggests significant differences in the extraction yields even among functional components with similar structures, which can be attributed to their differences in polarity and solubility. Similar studies were reported by Ferreira and Pinho (2012) [32] and Plaskova and Mlcek (2023) [28]. More polar flavonoid glycoside and aglycone groups may be extracted with binary mixtures of water–alcohol, but they are unsuitable for less polar flavonoids (isoflavones, flavanones, methylated flavones, and flavonols) [32]. The solubility of tannins and alkaloids is variable; depending on the target molecules, solvents with different polarities like water and ethanol, as well as their aqueous solutions, might be used for extraction [28].

Flavonoids possess many special functions and are one of the most potent nutraceuticals in food, herbs, and phytopharmaceutical products [33,34]. Some flavonoids have been isolated and identified from herbal teas or herbal extracts, such as 16 flavonoids from *Ginkgo biloba* dietary supplement tea [35], 11 from *Cistus incanus* herbal teas [36], and 28 from *Ampelopsis grossedentata* tea [37]. However, only limited information is currently available on the flavonoids in health wine made from edible and medicinal plants, such as three flavonoids, namely rutinum, isoquercitrin, and kaempferol 3-rutinoside, in health wine [38] and two, quercetin and 4′,5,6,7-tetramethoxyflavone, from alcohol extract of “Huainiuxi” (*Radix Achyranthis* Bidentatae) [39]. In this study, we found 150 upregulated flavonoids in “*Huangjin*” wine when compared with “*Huangjin*” tea, among which 75 (mainly flavanones, flavones, flavanols, and homoisoflavonoids) were present only in “*Huangjin*” wine (Table 2). Therefore, “*Huangjin*” wine contained a greater variety of flavonoids and also had a higher flavonoid content than “*Huangjin*” tea. Homoisoflavones (3-benzylidene-4-chromanones) are considered an infrequent flavonoid class in nature and have been reported to show a wide range of biological activities, including antimicrobial, antimutagenic, antioxidant, immunomodulatory, antidiabetic, cytotoxic, antiangiogenic, vasorelaxant, and anti-inflammatory; thus, they can be used as potential clinical medicines [40]. Homoisoflavones are characteristic components and a quality marker of *Polygonatum* plants [41]. Fifteen homoisoflavonoids identified from *P. cyrtonema* rhizomes through extraction with ethyl acetate or petroleum ester were reported by Mottaghipisheh and Stuppner (2021) [42]. In the current study, we initially identified a total of 28 homoisoflavonoids (including isomers); among them, 26 were found only in “*Huangjin*” wine, and the 2 others were significantly differentially extracted from “*Huangjin*” wine, with Log_2_FC values of 6.58 and 5.09, when compared with “*Huangjin*” tea (Table S5). This result suggests that the homoisoflavonoids in *P. cyrtonema* rhizomes are alcohol-soluble and nearly insoluble in hot water. This finding enriches the data on homoisoflavonoids in *P. cyrtonema* rhizomes as characteristic components and quality markers. Moreover, a greater variety of alcohol-soluble homoisoflavonoids and their higher contents contribute to the superior health benefits of “*Huangjin*” wine.

Our present study showed that alkaloids were the most abundant secondary metabolites in “*Huangjin*” beverages. Alkaloids are one of the largest groups of plant secondary metabolites and are present in several economically relevant plant families [43]. Alkaloids are also important components among the active ingredients in Chinese herbal medicines, with diverse and important pharmacological effects on human beings [44]. Extracts from herbal plants containing rich alkaloid contents have been used to treat several ailments, such as fever, snakebites, and insanity [45]. Some alkaloids, such as trigonelline, theophylline, theobromine, and caffeine, have been identified in *Cistus incanus* herbal infusions, with caffeine, which is a purine alkaloid, as the dominating alkaloid [36]. However, no available information on the alkaloids in health wine made from edible and medicinal plants has previously been reported. In this study, we found 105 upregulated alkaloids in “*Huangjin*” wine when compared with “*Huangjin*” tea, among which 35 (mainly alkaloids and amide alkaloids) were present only in “*Huangjin*” wine (Table 2). Amide alkaloids exhibit various bioactivities, such as anti-inflammatory, antiplatelet, and antioxidant activity [46,47,48]. N-trans-feruloyltyramine, which is an amide alkaloid that has been isolated and identified from the blue pigment in Laba garlic (*Allium sativum*), was proven to have significant antiradical properties and selective cytotoxic effects against cancer cells [49]. Among a total of 33 identified amide alkaloids in “*Huangjin*” beverages, 16 of them were found only in “*Huangjin*” wine, and the 17 others were significantly differentially extracted from “*Huangjin*” wine with a Log_2_FC value ≥ 1.06 when compared with “*Huangjin*” tea (Table S5). Therefore, “*Huangjin*” wine contained a greater variety of alkaloids and also had a higher alkaloid content than “*Huangjin*” tea. Amides were the most dominating alkaloids, which contribute to the superior health benefits of “*Huangjin*” wine.

With herbal health wine, the traditional concept is that the quality of the wine is better when it has been stored for longer. Thus, rhizome infusion is usually used to make herbal health wine by soaking rhizomes with Chinese liquor for more than 10 days. Our study indicated that rhizome infusion for a longer period could not further increase the solubilization of the functional components. On the contrary, continuous infusion led to a continuously decreasing terpenoid content in the “*Huangjin*” wine, which suggests that decomposition or structural changes may have occurred in these diterpenoids when the rhizomes were dissolved in Chinese wine for a longer period. Therefore, the health benefits of *“Huangjin”* wine were not enhanced simultaneously with continuous rhizome infusion, which also suggests that the traditional concept that the quality of the wine is better when it has been stored longer might not be true for herbal health wine made by the infusion of Chinese wine with plant materials.

As representative herbal beverages made from *P. cyrtonema* rhizomes, many herbal beverages are potential rich sources of phytochemicals that may help in the management of chronic diseases [27,50]. Although various herbal beverages are widely consumed in China and elsewhere in the world, the chemical and pharmaceutical mechanisms underpinning their claimed health benefits are poorly understood. Therefore, further analytical and clinical research is needed to investigate the functional medicinal components in herbal beverages and verify their medicinal effects in disease risk reduction and health promotion. Meanwhile, comparing *P. cyrtonema* extracts (“*Huangjin*” tea and “*Huangjin*” wine) obtained from crude rhizomes and processed rhizomes to assess their differences and identify the secondary metabolites responsible for their toxic effects might be an interesting direction of future work. In recent years, some important findings have shown that the gut microbiota can metabolize herbal medicines to produce new absorbable active small molecules with active pharmacological effects. Some studies have provided strong evidence on the anti-depression effects of traditional Chinese medicine related to the gut microbiota [51,52]. Thus, this interaction between herbs and the human microbiome should be considered in future studies by employing metabolomics and DNA sequencing technologies.

## 5. Conclusions

The major secondary metabolites in “*Huangjin*” beverages were polyphenols, including phenolic acids and flavonoids, as well as alkaloids. “*Huangjin*” wine has a greater variety of flavonoids and alkaloids than “*Huangjin*” tea, and the functional components in “*Huangjin*” wine were more highly abundant than those in “*Huangjin*” tea. Homoisoflavones and amide alkaloids were the dominating flavonoids and alkaloids in “*Huangjin*” wine, respectively. Continuous rhizome infusion could not increase the content of functional components in “*Huangjin*” wine. From the results obtained, this study not only provides primary evidence to support the claimed health benefits of “*Huangjin*” beverages but also suggests that traditional herbal beverages made by rhizome infusion have superior health benefits to those made by rhizome decoction, which is attributed to the higher yields of functional components extracted using Chinese liquor rather than hot water. Therefore, Chinese liquor shows advantages in terms of its use as a superior binary ethanol–water solvent in making herbal health beverages to enhance the solubility of poorly water-soluble functional components. Moreover, this study also suggests that the traditional concept that the quality of the wine is better when it has been stored for longer might not be true for herbal health wine made through the infusion of Chinese wine with plant materials. Future work should be devoted to analytical and clinical studies investigating the functional components, identifying the secondary metabolites responsible for the toxic effects of herbal beverages, and verifying their medicinal effects in health promotion. The interaction between traditional herbal medicine and the human microbiome should also be considered a meaningful research direction for gaining insights into the role of herbal medicine in treating diseases, which may be related to the gut microbiota.

## Figures and Tables

**Table 1:** The number of differentially extracted secondary metabolites identified from three comparison groups.

Comparison Group	DESM	Total Number	Class								
			Alkaloids	Phenolic Acids	Flavonoids	Terpenoids	Lignans and Coumarins	Quinones	Steroids	Tannins	Others
W7D vs. BW40	Up	483	105	58	150	41	25	8	45		47
	Down	3		1		1					1
W14D vs. W7D	Up	2	1				1				
	Down	0									
W21D vs. W14D	Up	3			1				2		
	Down	14	1	1	1	10					1

**Table 2:** The number of differentially extracted secondary metabolites only present in W7D samples.

Class I	Total Number	Class II
Flavonoids	75	Flavones (8); flavanones (8); flavonols (7); chalcones (4); flavanonols (1); other flavonoids (47, including 26 homoisoflavonoids)
Alkaloids	35	Alkaloids (16); amide (16); isoquinoline alkaloids (1); piperidine alkaloids (1); pyrrole alkaloids (1)
Terpenoids	14	Ditepenoids (7); sesquiterpenoids (3); triterpene (2); triterpene Saponin (2)
Phenolic acids	8	Phenolic acids (8)
Steroids	5	Steroidal saponins (3); steroid (2)
Quinones	4	Anthraquinone (4)
Lignans and coumarins	3	Coumarins (3)
Others	15	Others (14); aldehyde compound (1)

**Table 3:** Significantly enriched KEGG pathways related to flavonoid biosynthesis and upregulated DESMs in the comparison group W7D vs. BW40.

Pathway	Upregulated DESMs		Number of Upregulated DESMs
	Class	Compounds	
ko00941: flavonoid biosynthesis	Flavanones	Naringenin (5,7,4′-Trihydroxyflavanone)	12
	Flavonols	Kaempferol (3,5,7,4′-Tetrahydroxyflavone)	
	Flavanones	Sakuranetin	
	Flavanonols	3,5,7-Trihydroxyflavanone (Pinobanksin)	
	Flavanones	Butin 7,3′,4′-Trihydroxyflavanone	
	Phenolic acids	5-O-p-Coumaroylquinic acid	
	Phenolic acids	Chlorogenic acid (3-O-Caffeoylquinic acid)	
	Flavanones	Pinocembrin (Dihydrochrysin)	
	Flavanonols	Aromadendrin (Dihydrokaempferol)	
	Flavanones	Naringenin-7-O-glucoside (Prunin)	
	Chalcones	Naringenin chalcone 2′,4,4′,6′-Tetrahydroxychalcone	
	Flavonols	Quercetin	
ko00943: Isoflavonoid biosynthesis	Flavanones	Naringenin (5,7,4′-Trihydroxyflavanone)	1
ko00944: flavone and flavonol biosynthesis	Flavonols	Kaempferol (3,5,7,4′-Tetrahydroxyflavone)	6
	Flavones	Vitexin-2″-O-rhamnoside	
	Flavonols	Quercetin-3-O-rhamnoside (Quercitrin)	
	Flavones	Apigenin-7-O-neohesperidoside (Rhoifolin)	
	Flavones	Apigenin-6-C-glucoside (Isovitexin)	
	Flavonols	Quercetin	

## Data Availability

The data are contained within the article and Supplementary Materials.

## Supplementary Materials

The following supporting information can be downloaded at https://www.mdpi.com/article/10.3390/metabo14070376/s1. Figure S1: Correlation analysis of secondary metabolites from 12 “*Huangjin*” beverage samples. The color indicates a higher or lower Pearson’s correlation coefficient (PCC), with redder shades representing higher and greener shades representing lower values; Figure S2: Flavonoid biosynthesis pathways; Table S1: Detailed information on secondary metabolites identified in “*Huangjin*” health beverages; Table S2: Detailed information on DESMs only present in W7D; Table S3: Ten terpenoids clustering into variation pattern 1; Table S4: KEGG pathways and annotated compounds; Table S5: Homoisoflavonoids and amide alkaloids.
