# Supplementary material for: Investigation of the Functional Components in Health Beverages Made from Polygonatum cyrtonema Rhizomes Provides Primary Evidence to Support Their Claimed Health Benefits
Source: Metabolites. 2024 Jul 3;14(7):376. doi: 10.3390/metabo14070376 (PMC11279242; doi:10.3390/metabo14070376)
Supplement: Supplementary file 1 [file metabolites-14-00376-s001.zip › Table S1 Detail information of secondary metabolites identified in í░Huangjiní▒ health beverages.pdf]

Table S1 Detail information of secondary metabolites identified in "*Huangjin*" tea (BW40 sample) and "*Huangjin*" wine (W7D, W14D, and W21D samples)

Index	Formula	Compounds	Class I	Class II	BW40a	BW40b	BW40c	W7Da	W7Db	W7Dc	W14Da	W14Db	W14Dc	W21Da	W21Db	W21Dc
pmp001198	C6H13NO2	6-Deoxyfagomine	Alkaloids	Piperidine alkaloids	1.28E+07	1.19E+07	1.19E+07	1.54E+07	1.98E+07	1.80E+07	1.54E+07	2.04E+07	2.13E+07	1.54E+07	2.22E+07	1.82E+07
Xmbn009362	C15H16N2O	Arenarine B	Alkaloids	Alkaloids	3.10E+06	3.09E+06	3.03E+06	3.94E+06	3.57E+06	3.10E+06	3.19E+06	3.48E+06	3.39E+06	3.39E+06	3.60E+06	3.88E+06
pmf0175	C10H22O	2-Decanol*	Others	Alcohol compounds	7.22E+07	6.62E+07	7.34E+07	8.85E+07	8.61E+07	6.60E+07	7.51E+07	7.53E+07	7.01E+07	7.44E+07	7.43E+07	8.41E+07
pme0376	C15H12O5	Naringenin (5,7,4'-Trihydroxyflavanone)*	Flavonoids	Flavanones	0.00E+00	0.00E+00	0.00E+00	4.20E+04	2.36E+05	1.35E+05	3.72E+04	2.82E+05	1.60E+05	4.86E+04	2.77E+05	1.43E+05
Cmyp007180	C11H16O2	Dihydroactinidiolide	Others	Lactones	1.25E+05	8.46E+04	8.03E+04	5.23E+05	5.12E+05	4.54E+05	4.87E+05	4.09E+05	4.75E+05	5.10E+05	4.57E+05	4.55E+05
Ydhn005639	C22H34O7	Rhodojaponin V	Terpenoids	Ditepenoids	4.88E+04	4.82E+04	3.91E+04	1.12E+05	1.19E+05	9.96E+04	1.27E+05	9.73E+04	8.38E+04	1.10E+05	8.96E+04	8.53E+04
Hmgn001653	C7H6O3	Protocatechualdehyde*	Others	Aldehyde compounds	2.50E+06	2.03E+06	2.22E+06	2.26E+06	2.78E+06	2.27E+06	2.22E+06	2.55E+06	2.19E+06	2.38E+06	2.44E+06	2.22E+06
Wcfp007836	C18H39NO2	Tetradecyldiethanolamine	Alkaloids	Alkaloids	2.01E+07	1.89E+07	2.06E+07	4.03E+07	4.58E+07	3.85E+07	4.23E+07	4.15E+07	4.10E+07	4.01E+07	3.85E+07	3.59E+07
Zmsp000758	C8H15NO2	1-(Hydroxymethyl)hexahydro-1h-pyrrolizin-2-ol	Alkaloids	Pyrrole alkaloids	1.16E+07	1.09E+07	1.16E+07	3.02E+07	3.09E+07	3.21E+07	3.57E+07	3.73E+07	4.68E+07	4.08E+07	4.55E+07	4.33E+07
Lmmp002013	C14H22N2O3	Dihydroferuloylputrescine	Alkaloids	Phenolamine	3.31E+06	3.23E+06	3.09E+06	4.73E+06	6.28E+06	5.90E+06	4.59E+06	6.00E+06	5.30E+06	4.43E+06	5.87E+06	5.49E+06
Xmmn002939	C19H18O6	Methylophiopogonanone A	Flavonoids	Other Flavonoids	7.77E+04	7.17E+04	6.22E+04	1.28E+05	7.94E+04	6.12E+04	9.80E+04	1.16E+05	7.82E+04	8.33E+04	1.20E+05	1.39E+05
Lmgp000796	C8H7NO2	4-Hydroxymandelonitrile	Alkaloids	Phenolamine	4.53E+06	4.47E+06	4.79E+06	6.78E+06	8.50E+06	7.33E+06	6.00E+06	7.01E+06	6.70E+06	4.69E+06	7.08E+06	4.77E+06
Hmhn002787	C21H32O10	(1'R,3R,5R,8'S)-Dihydrophaseic acid-O-β-D-glucoside	Phenolic acids	Phenolic acids	1.16E+05	1.06E+05	7.06E+04	1.61E+05	1.36E+05	1.42E+05	1.83E+05	1.54E+05	1.39E+05	1.73E+05	1.87E+05	1.36E+05
Qahn004265	C24H30O9	Magnolignan A-2-O-glucoside	ignans and Coumarir	Lignans	2.74E+04	1.99E+04	4.98E+04	2.20E+05	7.85E+05	5.97E+05	1.51E+05	8.15E+05	5.49E+05	1.79E+05	7.60E+05	5.88E+05
Zbsn003878	C26H28O14	Vicenin-3	Flavonoids	Flavones	3.08E+05	2.82E+05	2.98E+05	1.34E+06	1.17E+06	1.37E+06	1.36E+06	1.33E+06	1.50E+06	1.51E+06	1.16E+06	1.64E+06
Hmmp001310	C11H9NO2	3-Indoleacrylic acid	Alkaloids	Plumerane	2.27E+07	2.20E+07	2.01E+07	3.41E+07	5.15E+07	3.81E+07	3.12E+07	4.90E+07	3.78E+07	2.70E+07	4.57E+07	3.33E+07
ZbWp001588	C15H19NO6	3-(((S)-1-carboxy-2-phenylethyl)amino)-2,5-dihydroxycyclopentane-1-carboxylic acid	Others	Others	2.46E+07	2.36E+07	1.97E+07	4.80E+07	5.14E+07	4.35E+07	4.56E+07	5.31E+07	4.70E+07	4.92E+07	4.39E+07	4.46E+07
Hmyn000254	C18H18O4	Tababiphenyl C	Others	Chromone	4.93E+05	5.29E+05	5.25E+05	8.02E+05	8.10E+05	7.86E+05	8.48E+05	8.12E+05	7.84E+05	9.21E+05	8.75E+05	8.67E+05
MWSHC2028	C13H14N2O2	(1S,3S)-1-Methyl-1,2,3,4-tetrahydro-β-carboline-3-carboxylic acid*	Alkaloids	Alkaloids	8.38E+06	8.55E+06	8.68E+06	1.55E+07	2.16E+07	1.73E+07	1.71E+07	2.59E+07	1.85E+07	1.70E+07	2.83E+07	2.13E+07
Wcdp005613	C11H8N2O	1,2-dihydro-1-oxo-β-carboline	Alkaloids	Alkaloids	3.02E+05	2.74E+05	2.70E+05	5.84E+05	6.65E+05	6.34E+05	6.80E+05	7.03E+05	6.11E+05	8.10E+05	7.49E+05	7.60E+05
MWSmce582	C6H9NO3	Methyl L-pyroglutamate	Alkaloids	Pyrrole alkaloids	4.22E+06	3.73E+06	3.24E+06	5.70E+06	6.66E+06	6.35E+06	5.70E+06	5.86E+06	6.00E+06	5.20E+06	5.09E+06	5.92E+06
MWSmce632	C10H10O4	Vanillin acetate	Phenolic acids	Phenolic acids	3.46E+05	3.14E+05	3.08E+05	8.72E+05	1.50E+06	1.31E+06	7.85E+05	1.69E+06	1.22E+06	7.78E+05	1.24E+06	1.15E+06
Lhfp112926	C20H22O5	Myricatomentogenin	Others	Others	1.13E+06	8.73E+05	7.61E+05	9.82E+05	8.93E+05	6.04E+05	7.62E+05	6.56E+05	7.86E+05	8.35E+05	1.13E+06	6.49E+05
MWSHY0072	C19H20O5	Methylophiopogonanone B*	Flavonoids	Other Flavonoids	0.00E+00	0.00E+00	0.00E+00	3.38E+05	1.45E+06	4.53E+05	3.53E+05	1.72E+06	5.35E+05	3.29E+05	1.73E+06	4.74E+05
Zjhp082636	C44H68O18	iso-Gentrogenin-Glc-Glc-Xyl*	Steroids	Steroidal saponins	1.31E+05	4.98E+04	9.49E+04	9.67E+04	1.60E+05	1.99E+05	2.14E+05	2.07E+05	2.25E+05	2.44E+05	2.90E+05	2.09E+05
Lmmp001410	C13H20N2O3	Dihydrocaffeoylputrescine	Alkaloids	Phenolamine	3.12E+06	2.93E+06	2.10E+06	4.93E+06	6.02E+06	6.04E+06	5.51E+06	5.93E+06	5.74E+06	5.17E+06	5.37E+06	5.13E+06
Lmmn005168	C20H32O4	Siegesbeckic acid	Terpenoids	Ditepenoids	2.28E+05	4.18E+04	2.12E+05	6.56E+05	1.79E+06	5.27E+04	2.67E+05	5.14E+05	3.65E+05	2.29E+05	5.29E+04	5.05E+04
MWStz070	C9H11NO3	N-(2-Hydroxy-4-methoxyphenyl)acetamide	Alkaloids	Phenolamine	1.10E+07	9.33E+06	9.11E+06	9.21E+06	1.08E+07	9.26E+06	9.24E+06	1.21E+07	1.63E+07	9.24E+06	1.58E+07	1.07E+07
MWSmce248	C9H8O3	3-Hydroxycinnamic Acid*	Phenolic acids	Phenolic acids	7.92E+05	5.07E+05	5.74E+05	9.32E+05	1.13E+06	7.78E+05	8.81E+05	1.03E+06	7.85E+05	9.02E+05	8.78E+05	8.43E+05
MWS1777	C6H6ClN	3-Chloroaniline	Alkaloids	Alkaloids	1.18E+07	1.23E+07	1.27E+07	1.36E+07	1.33E+07	1.30E+07	1.33E+07	1.28E+07	1.18E+07	1.18E+07	1.10E+07	1.14E+07
Lmbn014696	C20H30O2	Pimaric acid*	Terpenoids	Ditepenoids	4.87E+05	6.65E+05	6.70E+05	4.01E+06	1.12E+07	1.90E+06	4.17E+06	3.30E+06	3.00E+06	2.38E+06	1.16E+06	9.95E+05
Lmxp000939	C5H5N5	Zarzissine	Alkaloids	Alkaloids	0.00E+00	0.00E+00	0.00E+00	3.70E+07	3.70E+07	4.16E+07	3.74E+07	3.82E+07	4.31E+07	3.73E+07	4.66E+07	4.25E+07
MWSmce460	C5H9NO	2-Piperidone	Alkaloids	Piperidine alkaloids	4.08E+06	4.41E+06	4.46E+06	4.34E+06	3.98E+06	3.82E+06	3.55E+06	3.79E+06	3.38E+06	3.78E+06	3.89E+06	4.08E+06
Smcp001137	C11H9NO2	naphthisoxazol A	Alkaloids	Alkaloids	2.35E+07	2.13E+07	2.09E+07	3.53E+07	5.32E+07	4.13E+07	3.12E+07	4.89E+07	3.79E+07	3.01E+07	4.59E+07	3.51E+07
Lmhp003426	C13H12N2O2	Aspernigrin A	Alkaloids	Pyridine alkaloids	3.91E+06	3.73E+06	3.68E+06	6.08E+06	8.79E+06	7.45E+06	6.03E+06	9.57E+06	7.93E+06	6.49E+06	1.09E+07	8.05E+06
Zmcp102206	C24H30O4	(Z)-3,8-Dihydro 6,6·;7,3'a-diligustilide	Others	Others	4.07E+06	4.67E+06	4.60E+06	9.69E+06	1.62E+07	4.07E+06	8.81E+06	8.58E+06	8.56E+06	5.87E+06	5.33E+06	4.01E+06
pmp000088	C18H24O10	Regaloside A*	Phenolic acids	Phenolic acids	7.80E+04	9.61E+04	9.47E+04	4.81E+05	8.68E+05	8.17E+05	6.07E+05	1.09E+06	1.07E+06	5.71E+05	1.21E+06	9.90E+05
MWSHC2069	C15H24O3	(2R,5S,8S)-3,4,5,6,7,8-Hexahydro-2,5-dihydroxy-2,5-dimethyl-8-(1-methylethyl)-1(2H)-naphthalenone	Others	Others	1.66E+05	1.67E+05	1.93E+05	1.93E+05	1.88E+05	1.97E+05	2.28E+05	1.94E+05	1.48E+05	2.08E+05	1.64E+05	2.16E+05
Labn003910	C18H26O10	Benzyl B-Primeveroside*	Phenolic acids	Phenolic acids	5.73E+05	5.73E+05	5.88E+05	1.11E+06	1.06E+06	9.18E+05	1.15E+06	1.01E+06	9.85E+05	1.21E+06	1.09E+06	9.80E+05
Wcdp000970	C8H8O	acetophenone	Others	Others	2.82E+06	2.32E+06	2.42E+06	2.30E+06	3.08E+06	3.07E+06	2.91E+06	3.17E+06	5.20E+06	2.42E+06	3.77E+06	3.05E+06
Hmhn007438	C16H14O5	4'-Demethyl-3,9-dihydropunctatin	Flavonoids	Flavones	0.00E+00	0.00E+00	0.00E+00	4.46E+05	5.55E+06	2.44E+06	4.57E+05	6.28E+06	2.54E+06	4.27E+05	6.07E+06	2.32E+06
MWSslk208	C20H30O2	Kaurenoic Acid*	Terpenoids	Ditepenoids	5.13E+05	6.51E+05	7.94E+05	4.42E+06	1.13E+07	1.94E+06	4.90E+06	3.47E+06	3.19E+06	2.53E+06	1.22E+06	9.86E+05
Lmsn004132	C18H18O6	Hamiltone A	Flavonoids	Flavanones	3.94E+05	3.06E+05	3.82E+05	6.86E+05	1.18E+06	7.98E+05	1.04E+06	1.26E+06	1.09E+06	8.24E+05	1.38E+06	9.00E+05
MWSHC20158	C6H6O3	5-Methoxyfurfural*	Others	Aldehyde compounds	6.04E+06	5.45E+06	4.90E+06	6.71E+06	6.01E+06	6.08E+06	5.98E+06	5.31E+06	5.27E+06	6.02E+06	5.34E+06	5.27E+06
pmp001189	C38H40N2O10	(5-8)-Hydroxy-1-(hydroxyldimethoxyphenyl)-N2,N3-bis(4-hydroxyphenethyl)-(5-8)-dimethoxy-1,2-dihydronaphthalene-2,3-dicarboxamide	Alkaloids	Phenolamine	2.54E+04	6.06E+03	8.02E+03	3.52E+05	3.84E+05	2.95E+05	1.93E+05	2.49E+05	2.71E+05	2.79E+05	3.13E+05	3.22E+05
Wbmp004315	C24H29NO9	(1R)-6-hydroxy-1-(4-((3,4,5-trihydroxy-6-(hydroxymethyl)tetrahydro-2H-pyran-2-yl)oxy)benzyl)-1,2,3,4-tetrahydroisoquinolin-7-yl acetate	Alkaloids	Alkaloids	7.48E+04	1.25E+05	6.17E+04	3.92E+05	3.55E+05	2.94E+05	2.52E+05	4.01E+05	2.71E+05	2.49E+05	3.05E+05	3.50E+05
Zjmp102031	C19H20O5	methyl-iso-ophiopogonanone B*	Flavonoids	Other Flavonoids	0.00E+00	0.00E+00	0.00E+00	3.28E+05	1.32E+06	4.38E+05	3.16E+05	1.51E+06	4.91E+05	3.64E+05	1.63E+06	4.62E+05
Zmbp002461	C15H10N2O3	3-(2'-Hydroxyl-phenyl)-4-(3H)-quinazolone	Alkaloids	Alkaloids	2.24E+05	3.12E+05	1.89E+05	6.60E+05	9.47E+05	5.85E+05	8.57E+05	1.23E+06	5.69E+05	5.74E+05	9.28E+05	7.72E+05
Zjjp092610	C18H30O3	Dendronobilin I-iso1	Terpenoids	Sesquiterpenoids	0.00E+00	0.00E+00	0.00E+00	3.76E+05	4.52E+05	4.30E+05	3.90E+05	3.89E+05	3.95E+05	4.17E+05	4.35E+05	3.85E+05
Cmmn013378	C20H30O2	Levopimaric acid*	Terpenoids	Ditepenoids	4.68E+05	5.21E+05	6.06E+05	3.90E+06	9.17E+06	1.85E+06	3.82E+06	2.99E+06	3.13E+06	2.20E+06	1.21E+06	9.82E+05
MWS1962	C10H16O	cis-Citral	Terpenoids	Monoterpenoids	1.39E+06	1.32E+06	1.41E+06	1.85E+06	1.88E+06	1.92E+06	1.78E+06	1.92E+06	1.97E+06	1.76E+06	1.97E+06	1.90E+06
pmf0314	C20H34O	cis-Abienol	Terpenoids	Ditepenoids	5.26E+04	4.75E+04	4.79E+04	4.84E+04	3.61E+04	4.05E+04	5.47E+04	3.75E+04	4.60E+04	4.86E+04	4.13E+04	4.24E+04
Zmgn004894	C8H8O3	Methyl 4-hydroxybenzoate	Phenolic acids	Phenolic acids	9.06E+05	7.15E+05	7.54E+05	8.46E+05	8.41E+05	7.63E+05	7.69E+05	8.33E+05	7.05E+05	8.25E+05	7.82E+05	8.14E+05
mws0052	C21H18O11	Baicalin*	Flavonoids	Flavones	6.50E+06	4.37E+06	4.75E+06	1.68E+07	1.25E+07	1.69E+07	1.82E+07	1.30E+07	2.01E+07	1.96E+07	1.22E+07	1.70E+07
Lmhp003958	C11H18N2O2	Deoxymutaaspergillic acid	Alkaloids	Alkaloids	2.43E+06	2.47E+06	2.63E+06	2.49E+06	3.77E+06	2.89E+06	2.37E+06	3.25E+06	2.55E+06	2.25E+06	2.73E+06	2.44E+06
Zmhn002422	C16H20O9	1-O-Feruloyl-β-D-glucose*	Phenolic acids	Phenolic acids	8.32E+05	6.88E+05	7.70E+05	1.31E+06	1.04E+06	1.04E+06	1.20E+06	1.11E+06	1.13E+06	1.26E+06	1.10E+06	9.93E+05
Lmbn013410	C14H22O	2,4-Di-Tert-Butylphenol*	Phenolic acids	Phenolic acids	2.63E+06	2.53E+06	2.93E+06	9.57E+06	8.11E+06	7.48E+06	7.67E+06	4.32E+06	6.14E+06	7.33E+06	6.20E+06	7.01E+06
MWSmce254	C7H8O3	Ethyl maltol	Phenolic acids	Phenolic acids	0.00E+00	0.00E+00	0.00E+00	1.23E+06	1.21E+06	1.21E+06	1.23E+06	1.17E+06	1.26E+06	9.71E+05	1.11E+06	1.05E+06
Wbmn011269	C22H34O5	Vitexilactone	Terpenoids	Ditepenoids	2.08E+04	3.02E+04	1.94E+04	5.81E+06	4.18E+06	5.30E+06	4.51E+06	6.19E+06	6.14E+06	3.10E+06	6.11E+06	5.49E+06
mws0749	C7H6O3	4-Hydroxybenzoic acid	Phenolic acids	Phenolic acids	2.94E+06	2.32E+06	2.70E+06	2.69E+06	3.49E+06	2.76E+06	2.74E+06	3.14E+06	2.84E+06	2.61E+06	3.08E+06	2.77E+06
Lmrgn03360	C15H20O8	Cryptamygin B	Phenolic acids	Phenolic acids	7.82E+04	9.38E+04	8.88E+04	1.01E+05	1.30E+05	1.18E+05	1.33E+05	1.25E+05	1.36E+05	1.00E+05	1.15E+05	8.77E+04
Zjhp090605	C17H16O5	(3R)-5,7-dihydroxy-8-methyl-3-(4'-hydroxybenzyl)-chroman-4-one	Flavonoids	Other Flavonoids	0.00E+00	0.00E+00	0.00E+00	8.00E+05	5.81E+06	3.03E+06	7.60E+05	6.44E+06	3.23E+06	8.16E+05	6.30E+06	2.62E+06
Zjhp090621	C19H20O6	5,7-dihydroxy-6,8-dimethyl-3-(2'-methoxy-4'-hydroxybenzyl)-chroman-4-one*	Flavonoids	Other Flavonoids	0.00E+00	0.00E+00	0.00E+00	1.15E+06	1.86E+06	1.81E+06	1.15E+06	2.00E+06	1.70E+06	1.20E+06	1.97E+06	1.57E+06
Zahn006118	C16H22O4	1,3-Benzodioxole-5-nonanoic acid	Others	Others	4.53E+04	5.73E+04	5.05E+04	5.76E+04	5.42E+04	5.03E+04	5.63E+04	5.91E+04	4.84E+04	5.22E+04	5.60E+04	5.66E+04
pmb2893	C14H20N4O3	N-p-Coumaroylhydroxyagmatine	Alkaloids	Phenolamine	2.77E+04	3.30E+04	2.83E+04	1.70E+04	6.22E+04	4.29E+04	5.36E+04	7.78E+04	3.14E+04	3.78E+04	7.32E+04	5.89E+04
Wcsn009756	C20H30O4	Epoxyeleganolactone	Others	Others	0.00E+00	0.00E+00	0.00E+00	4.82E+05	4.29E+05	4.49E+05	4.42E+05	3.90E+05	4.24E+05	5.08E+05	5.59E+05	3.51E+05
Lhcp120314	C15H22O2	7β-H-9(10)-ene-11,12-Epoxy-8-oxoeremophilane	Terpenoids	Sesquiterpenoids	6.75E+06	6.92E+06	6.34E+06	7.88E+06	8.81E+06	8.26E+06	8.72E+06	7.53E+06	8.15E+06	7.77E+06	8.24E+06	8.32E+06
Lmcp002933	C16H18N2O3	Pilosine	Alkaloids	Alkaloids	8.56E+06	8.01E+06	8.43E+06	1.39E+07	1.74E+07	1.45E+07	1.58E+07	1.98E+07	1.53E+07	1.46E+07	1.99E+07	1.55E+07
pmn001380	C9H16O4	Eucommiol	Others	Alcohol compounds	1.52E+06	1.29E+06	1.35E+06	2.45E+06	2.35E+06	2.24E+06	2.27E+06	2.23E+06	2.30E+06	2.37E+06	2.23E+06	2.31E+06
HJN025	C7H8O2	4-Hydroxybenzyl Alcohol	Phenolic acids	Phenolic acids	1.06E+06	9.73E+05	8.94E+05	1.07E+06	1.12E+06	1.08E+06	1.03E+06	9.19E+05	1.05E+06	1.09E+06	1.03E+06	1.05E+06
Zmsp001775	C11H11NO5	1-[2-(furan-2-yl)-2-oxoethyl]-5-oxopyrroidine-2-carboxylic acid	Alkaloids	Pyrrole alkaloids	0.00E+00	0.00E+00	0.00E+00	2.02E+06	2.30E+06	2.18E+06	2.56E+06	2.63E+06	2.72E+06	2.32E+06	2.34E+06	2.65E+06
Wcdp005264	C14H31NO2	Tetradecasphinganine	Others	Others	3.38E+07	3.62E+07	4.06E+07	4.32E+07	4.67E+07	3.96E+07	3.87E+07	4.30E+07	3.41E+07	3.83E+07	3.96E+07	4.72E+07
Hmcp002354	C14H14N2O	Crenatine	Alkaloids	Plumerane	1.22E+05	1.13E+05	1.24E+05	1.49E+05	1.54E+05	1.55E+05	1.68E+05	1.53E+05	8.18E+04	1.47E+05	1.27E+05	1.39E+05
ML10174588	C15H22O2	Confertifoline	Terpenoids	Sesquiterpenoids	4.11E+07	4.05E+07	4.01E+07	6.05E+07	5.93E+07	5.61E+07	5.85E+07	5.86E+07	6.02E+07	5.80E+07	5.89E+07	6.72E+07
Zmdp000376	C5H11N3O	4-Guanidinobutanal	Others	Aldehyde compounds	3.98E+06	3.69E+06	4.01E+06	3.37E+06	3.92E+06	3.67E+06	3.74E+06	3.55E+06	4.24E+06	3.49E+06	3.72E+06	3.52E+06
Wcsn010254	C18H30O2	4-[3-(4,8-dimethylnona-3,7-dienyl)-3-methyloxiran-2-yl]butan-2-one	Others	Others	0.00E+00	0.00E+00	0.00E+00	6.11E+06	6.50E+06	6.34E+06	6.82E+06	7.13E+06	7.28E+06	7.56E+06	8.47E+06	6.62E+06
Zmgp004297	C19H20O2	1-phenyl-7-(4-hydroxyphenyl)-4-ene-3-heptanone	Others	Ketone compounds	4.84E+05	4.41E+05	4.53E+05	2.32E+06	3.51E+06	2.43E+06	2.89E+06	2.24E+06	2.74E+06	3.50E+06	1.83E+06	3.57E+06
Lssp210094	C12H21N3O7	2''-hydroxynicotinamide	Alkaloids	Alkaloids	2.31E+05	3.05E+05	2.58E+05	4.79E+05	4.88E+05	3.99E+05	4.26E+05	4.84E+05	5.21E+05	5.00E+05	4.85E+05	4.62E+05
HJAP048	C10H12O5	2,4,5-Trimethoxybenzoic acid	Phenolic acids	Phenolic acids	4.03E+05	3.73E+05	4.71E+05	7.39E+05	6.88E+05	4.96E+05	5.45E+05	6.24E+05	4.29E+05	5.52E+05	5.98E+05	5.80E+05
Zjhp090603	C16H14O5	(3R)-5,7-dihydroxy-3-(4'-hydroxybenzyl)-chroman-4-one	Flavonoids	Other Flavonoids	0.00E+00	0.00E+00	0.00E+00	1.45E+05	2.18E+06	8.03E+05	1.05E+05	2.09E+06	8.53E+05	1.40E+05	2.08E+06	8.98E+05
Zmbp002538	C11H12N2O2	1-Methoxy-indole-3-acetamide	Alkaloids	Plumerane	1.29E+07	1.19E+07	1.13E+07	1.99E+07	2.87E+07	2.25E+07	1.66E+07	2.60E+07	1.80E+07	1.54E+07	2.33E+07	1.68E+07
Zmhn001793	C15H18O9	6-O-Caffeoyl-D-glucose*	Phenolic acids	Phenolic acids	6.93E+04	5.94E+04	6.78E+04	9.64E+04	1.37E+05	1.43E+05	1.36E+05	1.75E+05	1.53E+05	1.36E+05	1.56E+05	1.48E+05
pmp001190	C37H38N2O9	(5-8)-Hydroxy-1-(hydroxylmethoxyphenyl)-N2,N3-bis(4-hydroxyphenethyl)-(5-8)-dimethoxy-1,2-dihydronaphthalene-2,3-dicarboxamide	Alkaloids	Phenolamine	0.00E+00	0.00E+00	0.00E+00	1.81E+05	3.08E+05	3.46E+05	2.78E+05	3.81E+05	3.67E+05	2.76E+05	3.88E+05	3.24E+05
Wbmn003185	C14H22O2	Kobusone	Terpenoids	Sesquiterpenoids	7.69E+06	7.81E+06	7.85E+06	8.62E+06	8.13E+06	8.34E+06	8.23E+06	8.49E+06	8.56E+06	7.80E+06	8.16E+06	8.64E+06
Hmdp001950	C17H18O5	2',7-Dihydroxy-3',4'-dimethoxyisoflavan	Flavonoids	Other Flavonoids	2.17E+07	2.18E+07	2.09E+07	3.87E+07	4.23E+07	3.67E+07	3.98E+07	4.36E+07	3.69E+07	4.10E+07	4.35E+07	3.75E+07
Smbp002161	C9H19NO	8-ethylnorlobelol	Alkaloids	Piperidine alkaloids	0.00E+00	0.00E+00	0.00E+00	2.62E+05	1.70E+05	2.83E+05	4.57E+05	4.14E+05	9.16E+05	4.76E+05	7.46E+05	5.42E+05
MWS1854	C6H7NO	4-Aminophenol	Alkaloids	Phenolamine	0.00E+00	0.00E+00	0.00E+00	8.83E+05	9.33E+05	9.75E+05	8.18E+05	1.06E+06	8.85E+05	9.35E+05	1.16E+06	1.10E+06
Zmzp005934	C18H37NO	Stearamide	Alkaloids	Alkaloids	3.70E+04	9.45E+04	3.67E+04	4.00E+05	3.04E+05	2.72E+05	3.26E+05	3.46E+05	4.22E+05	3.23E+05	3.47E+05	4.45E+05
Wcgn012576	C20H32O4	14-hydroxy-14-(hydroxymethyl)-5,9-dimethyltetracyclo[11.2.1.01,10.04,9]hexadecane-5-carboxylic acid	Terpenoids	Ditepenoids	2.75E+05	3.24E+05	2.91E+05	1.49E+06	2.05E+06	1.46E+06	1.51E+06	1.67E+06	1.48E+06	1.61E+06	2.06E+06	1.46E+06
MWSmce118	C5H6N2O2	Imidazole-4-Acetic Acid*	Alkaloids	Alkaloids	4.69E+06	4.48E+06	5.29E+06	5.69E+06	5.77E+06	4.75E+06	4.57E+06	4.11E+06	4.61E+06	4.74E+06	4.26E+06	4.17E+06
Wcsn010496	C18H30O2	6,10,14-Trimethylpentadeca-5,9-Diene-2,13-Dione	Others	Others	0.00E+00	0.00E+00	0.00E+00	1.25E+05	1.20E+05	1.20E+05	1.23E+05	1.22E+05	1.29E+05	1.56E+05	1.82E+05	1.18E+05
Hmgp002327	C11H9NO2	3-amino-2-naphthoic acid	Alkaloids	Alkaloids	2.41E+07	2.30E+07	1.99E+07	3.47E+07	5.18E+07	4.00E+07	3.25E+07	4.88E+07	3.59E+07	2.90E+07	4.58E+07	3.32E+07
MWS20172	C6H6O3	5-Hydroxymethylfurfural*	Others	Aldehyde compounds	5.78E+06	5.13E+06	5.20E+06	6.63E+06	5.82E+06	5.66E+06	6.07E+06	5.54E+06	5.22E+06	6.60E+06	5.34E+06	5.63E+06
Lmbn005454	C16H14O5	Naringenin-5-methyl ether	Flavonoids	Flavones	0.00E+00	0.00E+00	0.00E+00	2.37E+04	2.36E+05	1.19E+05	1.60E+04	2.70E+05	1.31E+05	1.84E+04	2.91E+05	9.61E+04
Lmmn006306	C18H30O3	Machilusolide D	Others	Lactones	0.00E+00	0.00E+00	0.00E+00	1.68E+06	2.13E+06	1.73E+06	1.70E+06	2.26E+06	1.93E+06	1.60E+06	2.28E+06	1.92E+06
mws0027	C9H10O5	Syringic acid	Phenolic acids	Phenolic acids	1.65E+06	1.24E+06	1.44E+06	1.73E+06	2.22E+06	1.92E+06	1.68E+06	2.02E+06	1.78E+06	1.91E+06	1.95E+06	1.88E+06
Jmyn006493	C18H18O5	(3R)-5,7-Dihydroxy-6,8-dimethyl-3-(4'-hydroxybenzyl)-chromone-4-one	Flavonoids	Other Flavonoids	0.00E+00	0.00E+00	0.00E+00	3.83E+05	2.44E+06	1.21E+06	3.73E+05	2.53E+06	1.20E+06	3.90E+05	2.64E+06	1.09E+06
pme3443	C11H12O4	Sinapinaldehyde	Phenolic acids	Phenolic acids	7.98E+04	4.59E+04	5.09E+04	3.54E+04	3.34E+04	4.99E+04	5.52E+04	4.46E+04	5.52E+04	5.05E+04	6.20E+04	5.24E+04
Lajp006846	C20H30O3	Imbricatusol D	Terpenoids	Ditepenoids	9.76E+04	1.82E+04	7.78E+04	5.49E+05	1.51E+06	6.98E+04	2.27E+05	4.51E+05	2.91E+05	1.57E+05	5.08E+04	5.77E+04
Wafp004334	C24H29NO9	N-Feruloyltyramine 4'-glucoside	Alkaloids	Phenolamine	1.89E+05	2.13E+05	1.13E+05	4.16E+05	2.75E+05	2.37E+05	3.01E+05	3.63E+05	2.79E+05	2.18E+05	2.72E+05	3.26E+05
Zbzn008048	C17H26O4	Embelin	Quinones	Quinones	6.76E+07	7.65E+07	8.18E+07	9.13E+07	9.13E+07	8.19E+07	7.87E+07	9.13E+07	8.54E+07	8.79E+07	8.49E+07	8.82E+07
pmb0782	C5H11N	Piperidine	Alkaloids	Piperidine alkaloids	9.88E+06	8.90E+06	9.38E+06	1.20E+07	1.48E+07	1.42E+07	1.21E+07	1.63E+07	1.67E+07	1.17E+07	1.75E+07	1.44E+07
Yhhn011469	C20H28O2	hemerocallal A	Terpenoids	Ditepenoids	2.93E+06	3.14E+06	3.91E+06	6.66E+06	1.37E+07	3.85E+06	6.19E+06	6.23E+06	5.52E+06	4.18E+06	3.93E+06	3.14E+06
MWSmce709	C9H7N	Isoquinoline	Alkaloids	Isoquinoline alkaloids	3.50E+05	2.86E+05	3.74E+05	4.90E+05	5.00E+05	5.85E+05	5.49E+05	5.31E+05	4.85E+05	4.23E+05	4.91E+05	5.13E+05
Wcsn010224	C18H30O2	14-hydroxy-2,6,10-trimethylpentadeca-2,5,10-trien-4-one	Others	Others	0.00E+00	0.00E+00	0.00E+00	6.60E+06	6.61E+06	6.74E+06	6.96E+06	8.02E+06	7.32E+06	8.02E+06	8.90E+06	6.89E+06
Hmgp002189	C22H22O11	Hispidulin-7-O-glucoside(Homoplantaginin)*	Flavonoids	Flavones	2.84E+04	3.01E+04	3.47E+04	3.89E+05	3.64E+05	3.53E+05	4.95E+05	4.17E+05	4.26E+05	4.06E+05	4.55E+05	5.05E+05
pme1738	C7H9N2O	3-Carbamyl-1-methylpyridinium;(1-Methylnicotinamide)	Alkaloids	Pyridine alkaloids	3.12E+06	3.27E+06	3.20E+06	3.92E+06	3.66E+06	3.84E+06	3.84E+06	3.34E+06	3.80E+06	4.05E+06	3.83E+06	3.65E+06
Cmmn012461	C20H28O2	Dehydroabietic acid	Terpenoids	Ditepenoids	3.27E+06	3.50E+06	3.80E+06	6.48E+06	1.30E+07	2.78E+06	7.01E+06	6.02E+06	6.55E+06	4.61E+06	3.80E+06	2.91E+06
Lmsn002247	C13H16O8	1-O-Salicyloyl-β-D-glucose	Phenolic acids	Phenolic acids	6.71E+05	6.83E+05	5.73E+05	9.31E+05	8.55E+05	1.04E+06	9.89E+05	1.07E+06	9.79E+05	8.96E+05	1.04E+06	8.64E+05
Hmcp003783	C13H10N2O	1-Acetyl-β-carboline	Alkaloids	Plumerane	3.44E+05	3.99E+05	2.59E+05	8.94E+06	1.00E+07	8.77E+06	9.70E+06	1.01E+07	9.52E+06	1.02E+07	1.11E+07	8.92E+06
Labn004625	C19H28O10	2-Phenylethyl beta-primeveroside	Phenolic acids	Phenolic acids	3.69E+05	3.25E+05	3.71E+05	7.15E+05	8.06E+05	9.69E+05	5.98E+05	7.52E+05	8.36E+05	6.57E+05	8.37E+05	9.56E+05
Lmsn003522	C19H20O5	Uvafzelic acid	Others	Others	5.15E+05	5.24E+05	4.65E+05	5.47E+05	5.60E+05	5.52E+05	5.35E+05	5.74E+05	5.25E+05	5.72E+05	5.78E+05	6.24E+05
Lalp007415	C17H16O5	5,7-dihydroxy-6-methyl-3-(4'-hydroxybenzyl)-4-chromanone*	Flavonoids	Other Flavonoids	0.00E+00	0.00E+00	0.00E+00	4.07E+05	7.55E+06	3.34E+06	4.91E+05	8.39E+06	3.39E+06	4.44E+05	8.63E+06	2.83E+06
Wbmp011352	C37H52N2O11	Puberaconitidine	Alkaloids	Alkaloids	0.00E+00	0.00E+00	0.00E+00	6.24E+07	6.25E+07	6.80E+07	6.99E+07	7.03E+07	6.79E+07	6.45E+07	7.37E+07	7.05E+07
Wbkp005691	C15H14N2O3	3-(4-methoxy-9H-pyrido[3,4-b]indol-1-yl)propanoic acid	Alkaloids	Alkaloids	9.60E+05	9.02E+05	9.14E+05	2.09E+06	3.16E+06	2.35E+06	2.18E+06	3.19E+06	2.37E+06	1.99E+06	3.12E+06	2.11E+06
Zjmp102042	C19H20O6	iso-5,7-dihydroxy-6,8-dimethyl-3-(4'-hydroxy-3'-methoxybenzyl)chroman-4-one*	Flavonoids	Other Flavonoids	0.00E+00	0.00E+00	0.00E+00	1.11E+06	1.59E+06	1.66E+06	1.28E+06	2.06E+06	1.83E+06	1.21E+06	2.09E+06	1.60E+06
MWSmce548	C5H11NO2	Betaine	Alkaloids	Alkaloids	6.64E+06	7.19E+06	7.24E+06	9.10E+06	1.06E+07	1.10E+07	8.16E+06	1.01E+07	1.14E+07	8.03E+06	9.81E+06	9.77E+06
HJN016	C36H36N2O8	Cannabisin D	Alkaloids	Alkaloids	0.00E+00	0.00E+00	0.00E+00	5.07E+05	6.44E+05	6.70E+05	5.79E+05	7.74E+05	7.52E+05	6.06E+05	6.97E+05	7.24E+05
pmb1754	C5H15NO4P+	O-Phosphocholine	Alkaloids	Alkaloids	3.13E+06	3.47E+06	3.02E+06	2.37E+06	2.67E+06	2.47E+06	2.04E+06	2.40E+06	2.84E+06	2.23E+06	2.25E+06	2.24E+06
MWSHC2062	C16H20O9	Ferulic β-glucoside*	Phenolic acids	Phenolic acids	8.01E+05	5.31E+05	6.74E+05	9.45E+05	1.04E+06	1.11E+06	1.06E+06	1.02E+06	1.06E+06	1.04E+06	9.81E+05	9.72E+05
Lhfp110832	C15H22O	Furanoeremophilane	Terpenoids	Sesquiterpenoids	3.85E+06	3.91E+06	3.55E+06	3.32E+06	3.22E+06	2.79E+06	3.40E+06	2.46E+06	2.56E+06	2.63E+06	2.22E+06	2.43E+06
zjbp120812	C22H32O8	Scutebarbolide C	Terpenoids	Ditepenoids	2.13E+07	2.27E+07	2.31E+07	2.19E+07	2.15E+07	2.30E+07	2.27E+07	2.09E+07	2.09E+07	2.06E+07	2.14E+07	2.23E+07
Lahp001956	C11H9O2N	Pyran[3,4-b]indole-2-ketone	Alkaloids	Plumerane	2.63E+07	2.16E+07	2.22E+07	3.65E+07	5.31E+07	4.31E+07	3.10E+07	5.08E+07	3.66E+07	2.83E+07	5.01E+07	3.29E+07
Zblp002750	C10H12O3	Methyl-3-(3-hydroxyphenyl)Propionate	Phenolic acids	Phenolic acids	2.89E+06	2.64E+06	3.00E+06	3.66E+06	4.05E+06	3.71E+06	3.56E+06	4.10E+06	3.72E+06	3.48E+06	4.32E+06	3.75E+06
Zmhn002227	C17H22O10	4-O-Glucosyl-sinapate	Phenolic acids	Phenolic acids	2.39E+05	2.22E+05	2.62E+05	2.85E+05	4.26E+05	3.85E+05	3.04E+05	3.68E+05	3.39E+05	3.56E+05	3.51E+05	4.30E+05
Jmbn003306	C25H32O11	Clemastanin A	ignans and Coumarir	Lignans	1.74E+05	2.38E+05	1.93E+05	3.01E+05	3.58E+05	3.71E+05	3.29E+05	3.36E+05	3.81E+05	3.75E+05	3.66E+05	3.08E+05
Jmwn002494	C15H18O8	(2E)-3-[4-(β-D-glucopyranoside)-phenylacrylic]-acid	Phenolic acids	Phenolic acids	3.63E+04	3.39E+04	4.06E+04	5.52E+04	8.27E+04	5.11E+04	5.64E+04	5.82E+04	5.76E+04	4.42E+04	4.51E+04	4.35E+04
Labn002833	C18H26O11	2-(Hydroxymethyl)Phenyl 6-O-Beta-D-Xylopyranosyl-Beta-D-Glucopyranoside	Phenolic acids	Phenolic acids	1.06E+06	8.84E+05	9.11E+05	2.31E+06	2.24E+06	2.08E+06	2.42E+06	2.29E+06	2.03E+06	2.24E+06	2.28E+06	2.11E+06
Zbfn001230	C19H26O14	3'-Gallic acyl sucrose	Phenolic acids	Phenolic acids	3.87E+05	3.27E+05	3.88E+05	1.90E+05	3.00E+05	2.09E+05	1.83E+05	3.77E+05	2.22E+05	2.19E+05	3.22E+05	1.88E+05
Layp000971	C14H21NO8	Hydroxymenisdaurin D	Alkaloids	Alkaloids	5.15E+05	8.42E+05	3.76E+05	1.42E+06	1.67E+06	1.63E+06	1.44E+06	1.66E+06	1.45E+06	1.57E+06	1.54E+06	1.53E+06
Hmtp000776	C8H9NO3	4,5,6-Trihydroxy-2-cyclohexen-1-ylideneacetonitrile	Alkaloids	Alkaloids	8.09E+05	7.42E+05	6.41E+05	1.08E+06	1.30E+06	1.12E+06	1.00E+06	1.30E+06	1.06E+06	8.98E+05	1.25E+06	1.08E+06
mws0628	C7H6O2	4-Hydroxybenzaldehyde	Others	Aldehyde compounds	1.67E+07	1.29E+07	1.40E+07	1.66E+07	1.86E+07	1.66E+07	1.72E+07	1.82E+07	1.76E+07	1.80E+07	1.98E+07	1.86E+07
mws1350	C9H10O4	Syringaldehyde; 4-Hydroxy-3,5-Dimethoxybenzaldehyde	Others	Aldehyde compounds	1.43E+06	1.18E+06	1.30E+06	1.12E+06	1.16E+06	1.11E+06	1.10E+06	1.15E+06	1.08E+06	1.16E+06	1.17E+06	1.15E+06
Zahp006974	C17H17NO2	Cinnamoyltyramine*	Alkaloids	Phenolamine	0.00E+00	0.00E+00	0.00E+00	3.35E+04	2.44E+05	1.06E+05	4.07E+04	2.67E+05	1.19E+05	4.31E+04	2.51E+05	9.65E+04
Wcdp011414	C22H43NO	Erucamide	Others	Others	3.22E+06	2.52E+05	4.34E+05	2.38E+05	4.16E+06	8.19E+06	2.29E+06	4.04E+06	5.86E+06	3.44E+06	3.06E+06	6.02E+06
Lamp008287	C17H16O5	3-Hydroxy-9,10-dimethoxypterocarpan	Flavonoids	Other Flavonoids	0.00E+00	0.00E+00	0.00E+00	3.39E+04	1.64E+05	7.50E+04	2.66E+04	2.07E+05	9.44E+04	2.80E+04	1.70E+05	8.60E+04
Wbmp003991	C24H40NO7+	11-Ethyl-6,18-dimethoxy-13-(methoxymethyl)-11-azoniahexacyclo[7.7.2.12,5.01,10.03,8.013,17]nonadecane-4,8,9,16-tetrol	Alkaloids	Alkaloids	1.16E+07	1.02E+07	9.73E+06	8.08E+07	6.70E+07	7.44E+07	1.04E+08	1.01E+08	9.44E+07	1.06E+08	1.05E+08	1.07E+08
mws1068	C15H10O6	Kaempferol (3,5,7,4'-Tetrahydroxyflavone)	Flavonoids	Flavonols	0.00E+00	0.00E+00	0.00E+00	1.54E+04	1.90E+05	9.72E+04	1.13E+04	2.35E+05	8.71E+04	1.62E+04	2.27E+05	8.46E+04
Lchp000221	C11H10O4	(Z)-3-Ethylidene-7-hydroxy-6-methoxyphthalide	Others	Others	3.33E+05	3.68E+05	2.27E+05	4.09E+05	3.31E+05	3.58E+05	2.90E+05	3.28E+05	2.72E+05	2.35E+05	3.55E+05	3.14E+05
Wafn002827	C16H22O9	Dihydroferulic acid glucoside	Phenolic acids	Phenolic acids	4.82E+05	4.69E+05	5.28E+05	5.81E+05	8.02E+05	6.68E+05	6.64E+05	6.16E+05	5.92E+05	5.38E+05	7.61E+05	6.66E+05
pmb0764	C6H9NOS	4-Methyl-5-thiazoleethanol	Others	Others	3.48E+06	2.98E+06	3.10E+06	4.54E+06	4.93E+06	4.57E+06	4.48E+06	4.69E+06	4.53E+06	4.49E+06	4.70E+06	4.54E+06
pmb0774	C10H12N2O	N-Hydroxytryptamine	Alkaloids	Plumerane	3.68E+05	3.46E+05	2.34E+05	2.64E+05	3.08E+05	2.72E+05	2.31E+05	3.24E+05	2.78E+05	2.97E+05	4.15E+05	2.56E+05
Wcfp001744	C5H9NO	N-Methylpyrrolidone	Others	Others	9.44E+06	9.56E+06	1.02E+07	9.10E+06	9.32E+06	8.66E+06	8.07E+06	8.10E+06	7.69E+06	8.10E+06	8.20E+06	9.02E+06
Wcgp011192	C16H24O2	5-Hydroxy-8,8-dimethyl-1,3,4,4a,4b,5,6,7,8a,9-decahydrophenanthren-2-one	Others	Others	1.17E+07	1.03E+07	1.13E+07	1.77E+07	1.64E+07	1.57E+07	1.50E+07	1.48E+07	1.36E+07	1.42E+07	1.51E+07	1.72E+07
Lhmp122205	C18H20O4	5-O-Methyllatifolin	Others	Others	2.07E+05	1.42E+05	1.84E+05	1.80E+05	1.80E+05	1.79E+05	1.67E+05	1.79E+05	2.34E+05	1.87E+05	1.76E+05	1.38E+05
Zjhn090628	C17H16O6	(3R)-5,7-Dihydroxy-8-methyl-3-(2',4'-dihydroxybenzyl)-chroman-4-one*	Flavonoids	Other Flavonoids	7.09E+03	4.07E+03	7.57E+03	1.17E+05	2.79E+05	2.40E+05	1.28E+05	2.96E+05	2.42E+05	1.43E+05	3.13E+05	2.01E+05
Zjhp090607	C16H14O6	Disporopsin/5,7-dihydroxy-3-(2',4'-dihydroxybenzyl)-chroman-4-one	Flavonoids	Other Flavonoids	0.00E+00	0.00E+00	0.00E+00	8.95E+04	3.82E+06	1.35E+06	1.00E+05	3.98E+06	1.32E+06	8.80E+04	3.38E+06	9.18E+05
Yacn003968	C11H13NO3	Cantleyine	Alkaloids	Alkaloids	4.86E+04	2.79E+04	2.55E+04	5.02E+05	7.22E+05	8.08E+05	5.12E+05	6.99E+05	7.01E+05	4.51E+05	7.30E+05	7.90E+05
Lmfn006411	C16H14O4	Cryptostrobin (8-C-Methyl-5,7-Dihydroxyflavanone)	Flavonoids	Flavanones	0.00E+00	0.00E+00	0.00E+00	8.44E+03	5.07E+05	8.95E+04	8.50E+03	5.81E+05	1.08E+05	4.81E+04	6.67E+05	7.95E+04
pmb3142	C13H16O8	Salicylic acid-2-O-glucoside	Phenolic acids	Phenolic acids	1.60E+06	1.54E+06	1.50E+06	2.06E+06	2.12E+06	2.17E+06	2.12E+06	2.23E+06	2.34E+06	2.10E+06	2.40E+06	2.21E+06
Wbjp002870	C13H16N2O3	1,3-Dimethyl-2-oxo-5-methoxyindoline-3-acetamide	Alkaloids	Alkaloids	1.78E+05	2.02E+05	1.50E+05	3.39E+05	6.33E+05	4.89E+05	3.06E+05	6.27E+05	4.57E+05	4.16E+05	5.99E+05	6.41E+05
Zjhp090615	C17H16O6	5,7-dihydroxy-8-methyl-3-(2',4'-dihydroxybenzyl)-chroman-4-one*	Flavonoids	Other Flavonoids	0.00E+00	0.00E+00	0.00E+00	1.25E+04	7.25E+05	2.01E+05	2.13E+04	8.24E+05	2.29E+05	3.06E+04	7.93E+05	2.20E+05
Zjhp082629	C39H62O13	iso-Diosgenin-Gal-Glc	Steroids	Steroidal saponins	3.08E+05	5.83E+05	4.54E+05	8.28E+05	8.85E+05	8.44E+05	6.85E+05	1.19E+06	1.30E+06	1.07E+06	8.73E+05	2.92E+05
Wcdp009119	C18H30O2	Elaidolinolenic acid	Phenolic acids	Phenolic acids	3.78E+04	2.71E+04	3.40E+04	5.86E+06	5.95E+06	5.98E+06	5.51E+06	5.89E+06	5.92E+06	5.71E+06	5.80E+06	5.48E+06
Wcfn008271	C14H8O4	Danthron; 1,8-Dihydroxyanthraquinone	Quinones	Anthraquinone	1.89E+07	1.89E+07	1.94E+07	1.79E+07	1.59E+07	1.57E+07	1.60E+07	1.53E+07	1.52E+07	1.42E+07	1.43E+07	1.41E+07
Wcdp006247	C26H48NO7P	1-18:3-LysoPC	Others	Others	4.52E+05	3.23E+05	4.34E+05	8.29E+06	9.89E+06	1.03E+07	9.13E+06	1.20E+07	1.08E+07	1.12E+07	1.34E+07	1.22E+07
Zjhp082615	C33H52O8	iso-Diosgenin-Gal	Steroids	Steroidal saponins	3.24E+04	1.18E+04	1.94E+04	3.09E+05	2.73E+05	3.24E+05	2.75E+05	3.24E+05	4.68E+05	4.18E+05	3.95E+05	4.21E+05
Lmtp002642	C26H28O14	Apigenin-8-C-(2''-glucosyl)arabinoside	Flavonoids	Flavones	3.10E+05	2.18E+05	2.18E+05	1.10E+06	1.01E+06	1.09E+06	1.35E+06	9.75E+05	1.03E+06	9.45E+05	1.11E+06	1.21E+06
Hmln003529	C18H26O10	Benzyl β-	Phenolic acids	Phenolic acids	5.52E+05	4.44E+05	4.96E+05	1.06E+06	1.06E+06	8.30E+05	9.62E+05	8.13E+05	8.22E+05	8.86E+05	1.00E+06	8.78E+05
Hmdp000626	C9H11NO2	Polygonatine A	Alkaloids	Alkaloids	5.16E+07	4.55E+07	4.52E+07	2.79E+07	2.26E+07	2.62E+07	2.76E+07	2.03E+07	4.62E+07	2.45E+07	2.88E+07	2.65E+07
Jmyp005575	C19H20O6	5,7-Dihydroxy-6,8-dimethyl-3-(3'-hydroxy-4'-methoxybenzyl)-chroma4-one*	Flavonoids	Other Flavonoids	0.00E+00	0.00E+00	0.00E+00	1.21E+06	2.33E+06	2.06E+06	1.44E+06	2.33E+06	2.15E+06	1.36E+06	2.26E+06	1.63E+06
Jmyn006479	C18H18O6	3,5,7-Trihydroxy-6,8-dimethyl-3-(4'-hydroxybenzyl)-chroman-4-one (Polygonatone C)	Flavonoids	Other Flavonoids	0.00E+00	0.00E+00	0.00E+00	2.66E+06	4.45E+06	4.14E+06	2.81E+06	4.63E+06	4.21E+06	2.84E+06	4.84E+06	3.55E+06
MWSmce466	C8H8O2	4-Hydroxyacetophenone	Others	Ketone compounds	7.24E+05	6.70E+05	5.36E+05	6.65E+05	7.59E+05	6.04E+05	8.64E+05	6.97E+05	7.54E+05	5.05E+05	6.32E+05	5.04E+05
pmb2871	C13H16O9	1-O-Gentisoyl-β-D-glucoside*	Phenolic acids	Phenolic acids	1.68E+06	1.59E+06	1.59E+06	3.55E+06	2.92E+06	2.70E+06	3.62E+06	3.30E+06	2.59E+06	3.60E+06	3.17E+06	2.88E+06
Zahp012577	C18H33NO	Octadecadienamide	Alkaloids	Alkaloids	2.78E+05	5.42E+05	3.61E+05	1.21E+06	8.51E+05	9.18E+05	9.51E+05	1.24E+06	1.42E+06	1.10E+06	1.06E+06	1.54E+06
Zjhp082616	C33H52O8	Diosgenin-Gal	Steroids	Steroidal saponins	6.05E+06	4.68E+06	4.85E+06	1.74E+07	1.88E+07	1.83E+07	1.84E+07	1.58E+07	2.20E+07	2.03E+07	1.98E+07	1.97E+07

Wdlp003530	C19H24N2O2	19(S),20(R)-dihydroperaksine	Alkaloids	Plumerane	1.38E+05	1.54E+05	2.21E+05	8.56E+05	9.44E+05	1.03E+06	1.16E+06	1.56E+06	1.31E+06	1.42E+06	1.28E+06	1.26E+06
mws0458	C8H8O3	Vanillin; 4-Hydroxy-3-Methoxybenzaldehyde	Others	Aldehyde compounds	5.22E+06	4.16E+06	4.71E+06	4.98E+06	5.06E+06	4.57E+06	5.01E+06	4.99E+06	4.40E+06	4.63E+06	4.62E+06	4.35E+06
pmp000966	C7H13NO2	Stachydrine	Alkaloids	Pyrrole alkaloids	9.67E+06	1.03E+07	1.06E+07	1.32E+07	1.42E+07	1.36E+07	1.29E+07	1.45E+07	1.48E+07	1.29E+07	1.41E+07	1.44E+07
Wcdn001824	C13H16O9	2-Hydroxy-5-[3,4,5-Trihydroxy-6-(Hydroxymethyl)Oxan-2-Yl]Oxybenzoic Acid	Others	Others	2.01E+06	1.99E+06	2.33E+06	4.51E+06	4.06E+06	3.92E+06	4.03E+06	4.13E+06	3.87E+06	4.88E+06	3.94E+06	3.62E+06
Zjhp090609	C18H16O5	(E)-5,7-dihydroxy-6,8-dimethyl-3-(4'-hydroxybenzylidene)-chroman-4-one	Flavonoids	Other Flavonoids	0.00E+00	0.00E+00	0.00E+00	1.39E+06	3.14E+06	1.53E+06	1.75E+06	3.52E+06	1.74E+06	1.72E+06	3.37E+06	1.46E+06
Hahn000897	C20H28O14	picrorhizin	Others	Others	2.85E+05	2.23E+05	2.40E+05	8.66E+05	7.80E+05	8.63E+05	8.55E+05	8.52E+05	8.26E+05	8.79E+05	9.15E+05	7.23E+05
mws0097	C20H22O6	Pinoresinol*	ignans and Coumarir	Lignans	1.20E+05	8.58E+04	6.87E+04	1.47E+05	2.44E+05	2.51E+05	1.65E+05	2.51E+05	2.14E+05	1.38E+05	2.47E+05	2.27E+05
Ladp002110	C10H7NO4	4-hydroxy-2-oxo-1,2-dihydroquinoline-3-carboxylic acid	Alkaloids	Quinoline alkaloids	2.95E+06	3.10E+06	2.45E+06	4.59E+06	4.88E+06	2.97E+06	4.43E+06	4.49E+06	4.19E+06	4.10E+06	3.85E+06	3.89E+06
Lmxp000429	C9H15N3O2	Hercynine	Alkaloids	Alkaloids	1.19E+06	1.03E+06	1.16E+06	9.68E+05	1.32E+06	1.23E+06	9.01E+05	1.35E+06	1.08E+06	1.06E+06	1.59E+06	1.02E+06
Cmdp004421	C39H60O13	Gentrogenin-3-O-glucosyl(1→4)fucoside (Kingianoside B)	Steroids	Steroidal saponins	2.26E+06	2.15E+06	1.94E+06	3.53E+06	3.02E+06	4.51E+06	5.99E+06	4.96E+06	5.02E+06	3.64E+06	2.83E+06	5.45E+06
MWS1830	C9H10O3	Ethylsalicylate	Phenolic acids	Phenolic acids	6.06E+04	9.62E+04	7.91E+04	1.93E+05	7.67E+04	1.11E+05	1.58E+05	1.03E+05	1.47E+05	1.16E+05	9.54E+04	1.20E+05
Wbjp001317	C11H10N2O2	vasicinone	Alkaloids	Alkaloids	6.70E+05	5.97E+05	5.26E+05	5.00E+05	8.40E+05	6.47E+05	5.13E+05	8.35E+05	6.20E+05	5.43E+05	7.74E+05	5.81E+05
Hmgp006095	C28H29O7N	Lyciumamide C	Alkaloids	Phenolamine	0.00E+00	0.00E+00	0.00E+00	7.38E+05	1.28E+06	1.19E+06	8.35E+05	1.47E+06	1.16E+06	8.06E+05	1.20E+06	1.17E+06
mws0011	C17H24O9	Syringin	Phenolic acids	Phenolic acids	1.23E+06	1.17E+06	1.22E+06	1.49E+06	1.56E+06	1.22E+06	1.43E+06	1.49E+06	1.21E+06	1.40E+06	1.43E+06	1.24E+06
Ysjp000315	C6H11NO2	Pipecolic acid	Alkaloids	Piperidine alkaloids	6.41E+05	6.38E+05	5.60E+05	9.02E+05	8.80E+05	9.62E+05	9.35E+05	8.73E+05	1.02E+06	9.63E+05	7.56E+05	9.43E+05
HJN003	C17H22O10	1-O-Sinapoyl-β-D-glucose	Phenolic acids	Phenolic acids	8.19E+05	6.52E+05	6.33E+05	6.92E+05	7.12E+05	7.87E+05	6.36E+05	6.55E+05	6.16E+05	6.43E+05	6.65E+05	8.42E+05
pmf0174	C10H22O	1-Decanol*	Others	Alcohol compounds	6.77E+07	6.38E+07	7.40E+07	8.70E+07	8.57E+07	7.05E+07	7.30E+07	7.67E+07	7.30E+07	7.16E+07	7.99E+07	8.75E+07
Lazn003727	C26H34O13	(1S,2R)-1-(3-Methoxy-4-Hydroxyphenyl)-2-[2-(β-D-Glucopyranosyloxy)-4-[(E)-3-Hydroxy-1-Propenyl]-6-Methoxyphenoxy]-1,3-Propanediol	ignans and Coumarir	Lignans	2.41E+06	1.97E+06	2.08E+06	2.88E+06	3.03E+06	2.69E+06	2.96E+06	3.14E+06	2.52E+06	2.81E+06	2.98E+06	2.43E+06
MWS0435	C8H9NO2	Acetaminophen	Alkaloids	Phenolamine	6.41E+06	5.36E+06	6.23E+06	6.77E+06	8.96E+06	9.33E+06	6.97E+06	7.62E+06	8.88E+06	7.10E+06	9.26E+06	8.02E+06
Lmyp003371	C7H8O2	3,5-Dihydroxytoluene	Phenolic acids	Phenolic acids	6.15E+06	5.56E+06	5.00E+06	7.85E+06	7.86E+06	6.45E+06	7.74E+06	8.09E+06	6.42E+06	7.48E+06	7.12E+06	6.57E+06
Hmcn001415	C17H24O10	Majoroside	Terpenoids	Monoterpenoids	9.73E+05	9.82E+05	1.04E+06	6.30E+05	9.28E+05	8.14E+05	6.63E+05	8.85E+05	7.49E+05	7.04E+05	8.39E+05	6.90E+05
Lmdn009099	C20H20O6	Coniferyl ferulate	Phenolic acids	Phenolic acids	0.00E+00	0.00E+00	0.00E+00	8.52E+04	4.11E+05	2.28E+05	8.85E+04	4.88E+05	2.56E+05	8.65E+04	4.97E+05	2.45E+05
Lskp211279	C10H16N2O4	Longitubanine A	Alkaloids	Alkaloids	1.61E+07	1.36E+07	1.42E+07	1.24E+07	1.53E+07	1.33E+07	1.24E+07	1.48E+07	1.33E+07	1.31E+07	1.46E+07	1.33E+07
MWSHC2027	C13H14N2O2	(1R,3S)-1-Methyl-1,2,3,4-tetrahydro-β-carboline-3-carboxylic acid*	Alkaloids	Alkaloids	8.78E+06	8.22E+06	8.64E+06	1.58E+07	2.30E+07	1.67E+07	1.73E+07	2.58E+07	1.87E+07	1.70E+07	2.79E+07	2.04E+07
Zjbp081703	C33H52O8	3-hydroxy-5,20-diene-26 furostanol-Glc	Steroids	Steroidal saponins	1.88E+06	1.47E+06	1.39E+06	1.40E+07	1.14E+07	1.23E+07	1.18E+07	1.23E+07	1.42E+07	1.18E+07	1.26E+07	1.12E+07
Labn003679	C15H20O8	(E)-caffeyl alcohol 4-O-β-D-glucopyranoside	Phenolic acids	Phenolic acids	5.38E+05	4.41E+05	4.47E+05	4.64E+05	5.40E+05	5.69E+05	5.26E+05	4.89E+05	5.33E+05	5.14E+05	5.16E+05	5.45E+05
Lmtn002233	C15H20O8	Androsin	Phenolic acids	Phenolic acids	2.06E+05	1.84E+05	2.18E+05	2.32E+05	2.85E+05	2.51E+05	2.56E+05	3.07E+05	2.31E+05	2.53E+05	2.92E+05	2.58E+05
Jmyn006245	C17H16O6	(3R)-5,7-Dihydroxy-8-methyl-3-(2',4'-dihydroxybenzyl)-chroma-4-one*	Flavonoids	Other Flavonoids	9.14E+03	6.29E+03	6.03E+03	1.89E+05	4.30E+05	3.48E+05	1.90E+05	4.53E+05	3.82E+05	2.03E+05	4.42E+05	3.00E+05
pmn001367	C13H16O9	Protocatechuic acid-4-O-glucoside*	Phenolic acids	Phenolic acids	2.27E+06	2.27E+06	2.48E+06	4.65E+06	3.91E+06	3.61E+06	4.65E+06	4.05E+06	3.74E+06	4.92E+06	4.35E+06	3.74E+06
Zjxp110301	C18H30O3	3-Hydroxy-3,7,11-trimethyldodeca-1,6E,10-trien-9-yl isobutyrate	Terpenoids	Sesquiterpenoids	0.00E+00	0.00E+00	0.00E+00	4.30E+05	5.50E+05	4.58E+05	3.78E+05	4.61E+05	3.77E+05	4.39E+05	4.76E+05	4.13E+05
Smhn003633	C20H28O13	Pyrafortunoside A	Phenolic acids	Phenolic acids	6.79E+04	5.74E+04	5.40E+04	1.13E+05	9.12E+04	1.01E+05	8.75E+04	6.69E+04	8.38E+04	7.80E+04	7.23E+04	7.90E+04
Wasn002329	C16H17NO9	Xanthurenic Acid 8-O-Glucoside	Alkaloids	Quinoline alkaloids	9.65E+05	9.19E+05	8.00E+05	1.84E+06	1.74E+06	1.71E+06	2.03E+06	1.75E+06	1.96E+06	2.16E+06	1.89E+06	1.79E+06
MWSmce501	C8H8O4	Protocatechuic Acid Methyl Ester	Phenolic acids	Phenolic acids	4.37E+04	3.29E+04	4.03E+04	4.43E+04	6.14E+04	6.10E+04	8.44E+04	6.69E+04	7.06E+04	8.30E+04	3.93E+04	7.56E+04
Hahp000801	C5H11NO2	alanine betaine	Alkaloids	Alkaloids	2.64E+07	2.40E+07	2.40E+07	2.55E+07	2.94E+07	2.75E+07	2.40E+07	3.17E+07	2.80E+07	2.29E+07	3.09E+07	2.41E+07
Wagp009406	C18H18O5	2'-Hydroxy-4,4',6'-Trimethoxychalcone; Flavokawain A	Flavonoids	Chalcones	0.00E+00	0.00E+00	0.00E+00	2.80E+05	1.99E+06	8.61E+05	2.51E+05	2.42E+06	1.05E+06	2.81E+05	2.27E+06	9.19E+05
Hmcn001884	C21H20O12	6-Hydroxyluteolin 5-glucoside*	Flavonoids	Flavones	3.01E+04	1.74E+04	2.27E+04	9.75E+04	2.76E+05	2.77E+05	1.15E+05	3.42E+05	2.66E+05	1.16E+05	3.35E+05	2.35E+05
Hmln007772	C17H26O4	Nordihydrocapsiate	Phenolic acids	Phenolic acids	6.68E+07	7.37E+07	7.88E+07	8.81E+07	8.64E+07	8.03E+07	7.61E+07	8.61E+07	8.42E+07	8.26E+07	8.52E+07	9.12E+07
Hmhp006830	C18H18O6	6,8-Dihydroxy-2-(2-hydroxy-4-methoxybenzyl)-7-methyl-3,4-dihydronaphthalen-1(2H)-one*	Flavonoids	Other Flavonoids	0.00E+00	0.00E+00	0.00E+00	1.09E+06	2.08E+06	1.88E+06	1.17E+06	2.12E+06	1.94E+06	1.10E+06	1.86E+06	1.41E+06
Hmhn006723	C16H14O6	Disporopsin	Flavonoids	Flavones	0.00E+00	0.00E+00	0.00E+00	1.40E+05	4.04E+06	1.71E+06	1.04E+05	4.61E+06	1.75E+06	1.41E+05	5.26E+06	1.43E+06
HJN012	C20H26O7	4,7,9,9'-Tetrahydroxy-3,3'-dimethoxy-8-O-4'-neolignan	Phenolic acids	Phenolic acids	5.95E+04	4.37E+04	4.95E+04	9.62E+04	7.36E+04	7.66E+04	9.65E+04	6.05E+04	6.49E+04	9.42E+04	8.50E+04	8.55E+04
Lamp007163	C22H22O10	4'-Demethyleucomin glucoside	Flavonoids	Other Flavonoids	0.00E+00	0.00E+00	0.00E+00	5.28E+05	4.74E+06	3.65E+06	9.32E+05	4.62E+06	3.86E+06	9.04E+05	5.99E+06	3.49E+06
Wagp004981	C23H24O13	Limocitrin 3-Glucoside*	Flavonoids	Flavones	7.57E+04	3.84E+04	4.46E+04	2.02E+05	2.66E+05	2.02E+05	3.25E+05	1.91E+05	1.59E+05	1.83E+05	2.53E+05	1.42E+05
pme3200	C2H7N3	1-Methylguanidine	Alkaloids	Alkaloids	2.70E+05	2.83E+05	2.80E+05	2.29E+05	1.73E+05	2.04E+05	2.22E+05	2.43E+05	1.93E+05	2.20E+05	2.35E+05	2.03E+05
Lmjn013497	C20H30O4	Agathic acid	Terpenoids	Ditepenoids	0.00E+00	0.00E+00	0.00E+00	4.46E+05	3.98E+05	3.97E+05	5.06E+05	4.23E+05	4.25E+05	4.33E+05	4.72E+05	3.92E+05
Wcdp004275	C14H31NO	Lauramine oxide	Others	Others	5.72E+06	5.43E+06	5.27E+06	6.35E+06	7.85E+06	6.45E+06	6.11E+06	6.47E+06	7.15E+06	5.60E+06	6.05E+06	7.29E+06
MWSmce573	C9H10O2	3,4-Dimethylbenzoic acid	Phenolic acids	Phenolic acids	5.08E+04	5.43E+04	4.26E+04	5.85E+04	6.49E+04	5.81E+04	6.67E+04	5.05E+04	4.83E+04	5.31E+04	5.74E+04	6.57E+04
Wcdp007560	C26H52NO7P	1-Vaccenoyl-Glycero-3-Phosphocholine	Others	Others	0.00E+00	0.00E+00	0.00E+00	3.60E+07	3.64E+07	4.75E+07	3.82E+07	4.33E+07	5.19E+07	3.95E+07	4.89E+07	5.00E+07
Lcyn000106	C26H32O13	Dichotomoside A	ignans and Coumarir	Lignans	1.01E+06	9.24E+05	9.13E+05	8.96E+05	1.06E+06	8.36E+05	8.85E+05	1.14E+06	9.09E+05	1.07E+06	1.11E+06	9.05E+05
Lmlp004754	C14H17NO7	Ehretioside B	Alkaloids	Phenolamine	0.00E+00	0.00E+00	0.00E+00	1.81E+05	1.65E+05	1.23E+05	1.01E+05	1.75E+05	1.03E+05	1.17E+05	1.05E+05	1.01E+05
Wchn003892	C16H20O9	Juglanoside E	Others	Others	7.61E+05	6.75E+05	7.79E+05	1.30E+06	1.05E+06	1.16E+06	1.34E+06	1.10E+06	1.20E+06	1.33E+06	1.12E+06	1.02E+06
Hmqn005002	C21H32O3	12-Hydroxymethylabietate	Terpenoids	Ditepenoids	1.07E+04	1.30E+03	1.99E+04	2.88E+04	8.31E+04	5.01E+03	1.36E+04	1.86E+04	1.43E+04	2.09E+04	3.55E+03	3.34E+03
Wayn000370	C10H19NO7	N-(1-Deoxy-1-fructosyl)Piperidic acid	Alkaloids	Alkaloids	2.36E+06	2.90E+06	2.44E+06	3.19E+06	3.41E+06	2.98E+06	2.92E+06	3.35E+06	3.08E+06	3.37E+06	3.28E+06	4.03E+06
Lahp002608	C11H9NO2	3,5-Dihydro-2H-Furo[3,2-C]Quinolin-4-One	Alkaloids	Quinoline alkaloids	2.33E+07	2.20E+07	2.01E+07	3.74E+07	5.59E+07	4.22E+07	3.37E+07	5.05E+07	3.68E+07	2.93E+07	4.58E+07	3.49E+07
mws0028	C8H8O4	Vanillic acid	Phenolic acids	Phenolic acids	8.37E+05	6.31E+05	7.33E+05	7.91E+05	9.39E+05	8.23E+05	8.12E+05	9.38E+05	8.41E+05	8.19E+05	8.74E+05	7.46E+05
Zbzp011453	C16H22O4	de-O-methyllasiodiplodin	Quinones	Quinones	3.71E+07	4.00E+07	3.09E+07	3.08E+07	2.60E+07	3.33E+07	3.15E+07	3.63E+07	3.07E+07	3.14E+07	3.12E+07	3.44E+07
Zjhp090635	C19H20O5	5,7-dihydroxy-6,8-dimethyl-3-(4'-methoxybenzyl)-chroman-4-one*	Flavonoids	Other Flavonoids	0.00E+00	0.00E+00	0.00E+00	2.87E+05	1.24E+06	4.21E+05	2.76E+05	1.50E+06	4.44E+05	3.02E+05	1.45E+06	3.43E+05
mws0444	C7H7NO3	3-Aminosalicylic acid	Phenolic acids	Phenolic acids	5.42E+05	4.70E+05	4.96E+05	6.92E+05	1.40E+06	9.43E+05	6.71E+05	1.08E+06	1.01E+06	7.01E+05	1.05E+06	1.12E+06
Wccp002870	C14H18N2O5	2-Amino-5-[(1-Carboxy-2-Phenylethyl)Amino]-5-Oxopentanoic acid	Alkaloids	Alkaloids	1.10E+06	9.19E+05	9.18E+05	1.79E+06	2.61E+06	2.22E+06	1.73E+06	2.91E+06	2.09E+06	1.70E+06	2.61E+06	2.03E+06
MWSmce089	C17H17NO3	p-Coumaroyltyramine*	Alkaloids	Phenolamine	0.00E+00	0.00E+00	0.00E+00	2.82E+07	4.02E+07	4.21E+07	2.63E+07	4.12E+07	4.38E+07	2.51E+07	3.59E+07	4.15E+07
pmp000970	C20H30O3	Hispanolone	Terpenoids	Ditepenoids	0.00E+00	0.00E+00	0.00E+00	2.71E+05	2.50E+05	4.15E+05	2.52E+05	2.42E+05	2.39E+05	2.68E+05	3.42E+05	2.80E+05
pme2693	C6H14N2O	N-Acetylputrescine	Alkaloids	Alkaloids	9.05E+06	7.79E+06	7.38E+06	1.38E+07	1.96E+07	1.90E+07	1.46E+07	2.13E+07	1.81E+07	1.46E+07	2.32E+07	1.80E+07
MWSmce448	C5H6N2O2	Imidazol-1-yl-acetic acid*	Alkaloids	Alkaloids	4.91E+06	4.75E+06	5.12E+06	5.43E+06	4.85E+06	5.11E+06	4.13E+06	4.43E+06	5.03E+06	4.86E+06	4.35E+06	4.30E+06
Sayp010368	C20H26O3	phyllanflexoid A	Terpenoids	Ditepenoids	1.47E+06	9.06E+05	1.59E+06	7.34E+06	1.98E+07	1.61E+06	6.38E+06	6.73E+06	5.08E+06	2.41E+06	1.12E+06	1.35E+06
pmf0085	C30H50O	9,19-Cyclolanost-24-en-3-ol (Cycloartenol)	Terpenoids	Triterpene	1.85E+06	1.79E+06	1.97E+06	1.65E+06	1.54E+06	1.55E+06	1.68E+06	1.63E+06	1.65E+06	1.65E+06	1.70E+06	1.60E+06
Wasn002449	C19H26O13	Protocatechuic acid 1-O-Rutinoside	Phenolic acids	Phenolic acids	2.04E+06	1.82E+06	1.87E+06	1.98E+06	2.62E+06	2.73E+06	1.87E+06	2.55E+06	2.57E+06	1.85E+06	2.72E+06	3.01E+06
zjbp111026	C20H22O5	tetrahydrochromone M	Others	Chromone	1.02E+06	9.25E+05	7.99E+05	8.87E+05	6.73E+05	1.06E+06	4.88E+05	7.29E+05	9.16E+05	7.82E+05	7.15E+05	7.86E+05
Lssp210092	C18H19NO4	N-trans-ferulic tyramine*	Alkaloids	Phenolamine	1.74E+05	1.94E+05	1.97E+05	3.59E+07	5.18E+07	4.89E+07	3.89E+07	4.79E+07	4.87E+07	3.80E+07	5.37E+07	4.21E+07
MW0139629	C16H14O5	Sakuranetin	Flavonoids	Flavanones	0.00E+00	0.00E+00	0.00E+00	2.58E+05	3.25E+06	1.38E+06	2.33E+05	3.49E+06	1.31E+06	2.15E+05	3.74E+06	1.05E+06
Hmcp002207	C22H22O12	Isorhamnetin-7-O-glucoside (Brassicin)*	Flavonoids	Flavonols	1.09E+05	3.07E+04	2.05E+04	5.54E+05	6.44E+05	9.22E+05	5.26E+05	8.64E+05	1.16E+06	4.80E+05	9.24E+05	7.48E+05
MWStz038	C36H36N2O8	Grossamide	Alkaloids	Phenolamine	0.00E+00	0.00E+00	0.00E+00	2.94E+06	3.69E+06	3.89E+06	3.20E+06	4.00E+06	4.20E+06	3.25E+06	4.05E+06	3.64E+06
Lssp210412	C13H24N2O	Cuscohygrine	Alkaloids	Pyrrole alkaloids	8.04E+05	7.94E+05	8.46E+05	7.50E+05	7.25E+05	7.08E+05	8.00E+05	7.55E+05	6.82E+05	7.20E+05	6.99E+05	6.55E+05
pmp001311	C27H30O17	6-Hydroxykaempferol-7,6-O-Diglucoside	Flavonoids	Flavonols	3.57E+05	3.54E+05	4.14E+05	1.22E+06	9.59E+05	1.17E+06	1.25E+06	1.05E+06	1.00E+06	9.74E+05	1.07E+06	1.18E+06
MWSHC20168	C7H6O4	Methyl cumalate	Phenolic acids	Phenolic acids	3.48E+05	2.03E+05	2.31E+05	3.26E+05	4.54E+05	3.63E+05	3.24E+05	3.36E+05	3.51E+05	3.21E+05	4.61E+05	3.88E+05
mws1014	C10H8O5	Fraxetin (7,8-Dihydroxy-6-methoxycoumarin)	ignans and Coumarir	Coumarins	2.51E+06	2.33E+06	2.73E+06	2.50E+06	3.46E+06	3.27E+06	2.42E+06	3.51E+06	3.46E+06	2.20E+06	3.51E+06	2.88E+06
MWSslk183	C15H10O4	7,8-Dihydroxy-4-phenylcoumarin	ignans and Coumarir	Coumarins	3.81E+04	3.77E+04	3.64E+04	1.78E+06	2.01E+06	1.90E+06	1.83E+06	2.28E+06	2.07E+06	1.91E+06	2.31E+06	1.82E+06
pmn001583	C27H30O16	Quercetin-3-O-robinobioside	Flavonoids	Flavonols	1.89E+04	1.87E+04	1.33E+04	2.69E+04	3.00E+04	2.52E+04	3.54E+04	4.04E+04	2.06E+04	2.83E+04	3.32E+04	1.49E+04
Lmyn006922	C22H24O10	Sakuranin	Flavonoids	Flavanones	3.93E+04	4.22E+04	4.66E+04	1.70E+05	2.11E+05	1.93E+05	1.92E+05	1.84E+05	1.95E+05	2.18E+05	1.91E+05	1.82E+05
Zdcp003644	C13H18N2O3	N-Caffeoylputrescine	Alkaloids	Phenolamine	5.75E+05	5.64E+05	5.37E+05	9.40E+05	1.01E+06	9.55E+05	9.17E+05	1.05E+06	1.11E+06	1.07E+06	1.18E+06	1.04E+06
Wccp005403	C18H19NO4	Acetyl-Caranine	Alkaloids	Alkaloids	2.00E+05	2.03E+05	1.58E+05	3.74E+07	5.62E+07	4.86E+07	3.96E+07	5.06E+07	4.97E+07	3.91E+07	4.97E+07	4.48E+07
Lmlp003161	C14H20N2O3	N-Feruloylputrescine	Alkaloids	Phenolamine	1.37E+06	1.29E+06	1.28E+06	1.76E+06	2.52E+06	1.59E+06	1.49E+06	2.75E+06	1.81E+06	1.54E+06	2.61E+06	1.29E+06
Zjhp090619	C18H18O6	(3R)-5,7-dihydroxy-8-methyl-3-(2'-hydroxy-4'-methoxybenzyl)-chroman-4-one	Flavonoids	Other Flavonoids	0.00E+00	0.00E+00	0.00E+00	8.22E+04	1.80E+06	3.55E+05	8.70E+04	1.92E+06	4.26E+05	8.31E+04	1.92E+06	3.00E+05
pme2433	C4H11NO2	Diethanolamine	Alkaloids	Alkaloids	2.16E+05	2.57E+05	2.04E+05	1.56E+05	1.42E+05	1.32E+05	1.23E+05	1.01E+05	1.40E+05	1.28E+05	1.25E+05	1.61E+05
Wbkp012886	C22H32O8	(1S,3R,3a1S,8R,10S,11aS)-1,5,10-trihydroxy-3,3a1,8,11a-tetramethyl-2,11-dioxohexadecahydrodibenzo[de,g]chromen-4-yl acetate	Terpenoids	Ditepenoids	2.29E+07	2.24E+07	2.35E+07	2.24E+07	2.34E+07	2.24E+07	2.43E+07	2.23E+07	2.23E+07	2.17E+07	2.23E+07	2.16E+07
pme2828	C6H5NO3	4-Nitrophenol	Phenolic acids	Phenolic acids	2.41E+07	2.18E+07	2.04E+07	2.08E+07	1.74E+07	1.58E+07	1.94E+07	1.65E+07	1.54E+07	1.83E+07	1.54E+07	1.42E+07
HJN055	C21H24O10	Dihydrocharcone-4'-O-glucoside	Flavonoids	Chalcones	1.04E+05	9.89E+04	1.19E+05	4.16E+05	3.66E+05	3.30E+05	3.81E+05	3.90E+05	4.22E+05	4.20E+05	2.64E+05	3.76E+05
Hmtn001288	C9H10O4	Methyl 2,4-dihydroxyphenylacetate	Phenolic acids	Phenolic acids	4.87E+04	5.34E+04	2.37E+04	3.40E+05	3.10E+05	3.26E+05	2.45E+05	2.75E+05	3.06E+05	2.99E+05	3.21E+05	3.15E+05
Wasn002933	C16H22O10	1-O-(3,4,5-Trimethoxybenzoyl)-B-D-Glucopyranoside	Phenolic acids	Phenolic acids	1.00E+06	7.59E+05	8.67E+05	2.19E+06	1.77E+06	1.93E+06	1.95E+06	1.73E+06	1.82E+06	1.97E+06	1.86E+06	1.62E+06
Lmlp001436	C9H10O4	Dihydrocaffeic acid	Phenolic acids	Phenolic acids	5.03E+04	2.23E+04	4.02E+04	2.37E+04	7.08E+04	1.36E+05	7.95E+04	1.65E+04	8.91E+04	5.71E+04	4.24E+04	8.50E+04
pmp000089	C18H24O10	Regaloside H*	Phenolic acids	Phenolic acids	7.59E+04	8.92E+04	7.35E+04	4.51E+05	9.40E+05	8.11E+05	5.14E+05	9.64E+05	9.89E+05	5.42E+05	9.35E+05	1.03E+06
mws5028	C7H5NO2	2-Benzoxazolinone	Alkaloids	Alkaloids	3.22E+05	2.89E+05	2.66E+05	2.56E+05	4.12E+05	3.52E+05	4.54E+05	3.43E+05	4.25E+05	3.72E+05	3.65E+05	3.65E+05
Lmlp005236	C21H22O11	Dihydrokaempferol-3-O-glucoside	Flavonoids	Flavanonols	2.08E+05	1.57E+05	2.04E+05	2.70E+05	2.61E+05	2.36E+05	2.85E+05	2.91E+05	3.31E+05	3.02E+05	3.84E+05	3.77E+05
Lhmp110110	C22H24O7	Pyramidatin F(iso-01)	Others	Others	4.06E+05	3.45E+05	3.28E+05	9.80E+05	9.15E+05	1.05E+06	9.44E+05	1.06E+06	1.01E+06	9.12E+05	9.24E+05	9.96E+05
pmp001004	C16H12N2O2	Perlolyrine	Alkaloids	Plumerane	5.69E+05	7.31E+05	6.55E+05	1.88E+07	2.00E+07	1.88E+07	1.97E+07	2.13E+07	1.92E+07	1.89E+07	1.98E+07	1.66E+07
Wbjp001169	C11H12N2O3	o-Carboxy-5-hydroxytryptamine	Alkaloids	Alkaloids	6.14E+05	4.99E+05	5.03E+05	5.35E+05	7.62E+05	5.11E+05	4.64E+05	6.93E+05	6.09E+05	4.95E+05	7.85E+05	5.43E+05
Lmbn002648	C9H8O3	α-Hydroxycinnamic Acid*	Phenolic acids	Phenolic acids	7.78E+05	4.87E+05	5.66E+05	8.40E+05	1.01E+06	8.04E+05	8.65E+05	1.01E+06	6.97E+05	8.20E+05	9.37E+05	9.49E+05
Yacp000453	C5H9NO2	3-hydroxy-1-methylpyrrolidin-2-one	Alkaloids	Pyrrole alkaloids	3.77E+06	3.67E+06	3.98E+06	2.55E+06	3.33E+06	2.82E+06	2.36E+06	2.71E+06	2.78E+06	2.18E+06	2.70E+06	2.28E+06
Zbdn001947	C13H18O8	Isotachioside*	Phenolic acids	Phenolic acids	1.92E+05	1.89E+05	1.88E+05	2.57E+05	2.42E+05	2.82E+05	2.53E+05	2.64E+05	2.98E+05	2.94E+05	2.71E+05	2.56E+05
pmf0348	C10H20O3	2,6-Dimethyl-7-octene-2,3,6-triol	Others	Alcohol compounds	1.92E+06	1.36E+06	1.28E+06	3.03E+06	2.98E+06	2.90E+06	2.89E+06	2.44E+06	2.69E+06	2.92E+06	2.81E+06	2.83E+06
Wcgn012724	C16H26O4	6-Hydroxyphomodiol	Others	Others	3.01E+06	3.59E+06	3.05E+06	1.38E+07	1.38E+07	1.48E+07	1.54E+07	1.17E+07	1.69E+07	1.37E+07	1.44E+07	1.20E+07
Xmqp004074	C20H26O10	Peucedanol 3'-O-glucoside	ignans and Coumarir	Coumarins	4.52E+05	6.00E+05	4.43E+05	2.61E+06	2.66E+06	2.68E+06	3.16E+06	2.50E+06	2.78E+06	3.04E+06	2.71E+06	2.04E+06
Zahp013600	C18H35NO	Octadec-2-enamide	Alkaloids	Alkaloids	1.12E+06	2.47E+06	1.37E+06	5.12E+06	2.70E+06	3.77E+06	4.18E+06	5.09E+06	6.24E+06	4.81E+06	4.30E+06	6.75E+06
Lmmn001294	C14H20O9	Koaburaside	Phenolic acids	Phenolic acids	2.77E+05	3.19E+05	2.95E+05	3.25E+05	4.64E+05	4.22E+05	3.21E+05	4.47E+05	3.99E+05	3.14E+05	3.92E+05	4.09E+05
Wcdn004141	C26H36O12	2-[3-[4-[1,3-dihydroxy-1-(4-hydroxy-3-methoxyphenyl)propan-2-yl]oxy-3-methoxyphenyl]propoxy]-6-(hydroxymethyl)oxane-3,4,5-triol	Others	Others	6.15E+05	6.53E+05	5.68E+05	1.44E+06	1.24E+06	1.14E+06	1.45E+06	1.19E+06	1.26E+06	1.48E+06	1.20E+06	1.18E+06
Zmln000899	C15H20O9	Syringaldehyde-4-O-glucoside	Phenolic acids	Phenolic acids	4.41E+04	3.90E+04	6.12E+04	2.21E+05	2.13E+05	2.04E+05	1.71E+05	2.13E+05	2.18E+05	1.92E+05	2.23E+05	2.58E+05
Hmcp002187	C23H24O13	Limocitrin-3-O-galactoside*	Flavonoids	Flavonols	9.86E+04	8.29E+04	1.09E+05	4.48E+05	3.46E+05	2.24E+05	2.94E+05	1.56E+05	1.13E+05	4.66E+05	3.71E+05	3.46E+05
Zmbp002117	C10H9NO2	1-Methoxy-3-indolealdehyde	Alkaloids	Plumerane	2.94E+05	2.64E+05	2.44E+05	3.62E+05	3.82E+05	4.54E+05	3.11E+05	4.21E+05	5.13E+05	4.05E+05	4.35E+05	4.68E+05
Smln008909	C15H14O4	Aloebarbendol	Phenolic acids	Phenolic acids	7.10E+04	6.62E+04	7.23E+04	1.02E+05	1.10E+05	1.08E+05	1.15E+05	1.13E+05	1.15E+05	1.09E+05	1.14E+05	1.18E+05
Lhzp102630	C33H52O8	3-Epidiosgenin-3-O-glucoside*	Steroids	Steroidal saponins	1.67E+06	1.30E+06	1.20E+06	1.12E+07	1.05E+07	1.17E+07	8.67E+06	1.19E+07	1.02E+07	1.08E+07	8.42E+06	9.54E+06
Lmfp005064	C56H92O29	Digitogenin-3-O-glucosyl(1→3)galactosyl(1→2)[xylosyl(1→3)]glucosyl(1→4)galactoside (Digitonin)	Steroids	Steroidal saponins	5.22E+06	3.30E+06	5.01E+06	1.13E+07	1.10E+07	9.74E+06	1.46E+07	9.91E+06	1.43E+07	1.33E+07	1.20E+07	1.04E+07
Zbfn002396	C15H20O9	Caffeoyl-O-mannitol	Phenolic acids	Phenolic acids	2.18E+05	1.90E+05	1.83E+05	6.81E+05	5.32E+05	6.60E+05	6.28E+05	6.08E+05	6.01E+05	6.77E+05	5.30E+05	6.61E+05
Lmhn002437	C7H6O4	Terreic acid	Others	Ketone compounds	2.42E+05	2.08E+05	1.77E+05	3.04E+05	4.47E+05	3.55E+05	3.33E+05	3.83E+05	3.67E+05	3.42E+05	4.38E+05	3.50E+05
Zjzp081813	C39H64O13	Timosaponin A-II	Steroids	Steroidal saponins	1.72E+05	2.09E+05	1.99E+05	1.59E+06	1.09E+06	1.17E+06	1.24E+06	1.29E+06	1.07E+06	8.91E+05	7.81E+05	1.23E+06
Wcdp006912	C16H35NO2	Lauryldiethanolamine	Others	Others	1.06E+07	1.15E+07	1.18E+07	1.33E+07	1.46E+07	1.37E+07	1.36E+07	1.39E+07	1.36E+07	1.36E+07	1.36E+07	1.53E+07
Zasn010433	C20H36O2	9,12-Octadecadienoic acid,ethyl ester	Others	Others	0.00E+00	0.00E+00	0.00E+00	2.88E+05	1.86E+05	1.76E+05	2.58E+05	2.92E+05	2.68E+05	2.93E+05	2.40E+05	2.20E+05
Qayp008402	C35H56O14	Sileneoside H	Steroids	Steroidal saponins	0.00E+00	0.00E+00	0.00E+00	2.85E+05	3.64E+05	3.06E+05	3.87E+05	3.73E+05	4.08E+05	3.34E+05	4.05E+05	3.93E+05
pmn001375	C26H32O12	1-Hydroxypinoresinol-1-O-Glucoside	ignans and Coumarir	Lignans	1.74E+05	1.51E+05	1.75E+05	1.56E+05	2.16E+05	1.66E+05	1.63E+05	2.14E+05	1.50E+05	1.47E+05	2.54E+05	1.86E+05
Lafp003256	C11H14O5	3,4'-Dihydroxy-3',5'-dimethoxypropiophenone	Others	Ketone compounds	1.60E+06	1.69E+06	1.71E+06	2.13E+06	3.56E+06	2.31E+06	2.40E+06	4.19E+06	2.49E+06	2.08E+06	4.13E+06	2.29E+06
Wcsn010794	C20H32O2	(3E,5E,8Z,11Z)-7,10,15-trimethylheptadeca-3,5,8,11-tetraenoic acid	Others	Others	9.52E+03	2.12E+04	2.27E+04	9.50E+04	2.40E+05	4.52E+04	9.78E+04	7.24E+04	6.29E+04	5.19E+04	2.68E+04	2.28E+04
Wbmn010531	C22H34O5	Previtexilactone	Terpenoids	Ditepenoids	9.74E+03	5.70E+03	7.57E+03	1.68E+06	1.60E+06	1.51E+06	1.33E+06	1.61E+06	1.55E+06	1.22E+06	1.23E+06	1.43E+06
pmb2795	C10H10O3	4-Methoxycinnamic acid	Phenolic acids	Phenolic acids	7.80E+04	4.41E+04	7.47E+04	2.71E+05	2.86E+05	3.00E+05	3.91E+05	3.20E+05	3.22E+05	4.05E+05	4.41E+05	4.48E+05
Hmqp002567	C17H17NO3	4-Hydroxycinnamic acid p-hydroxyphenethylamin	Phenolic acids	Phenolic acids	0.00E+00	0.00E+00	0.00E+00	2.80E+07	4.11E+07	4.08E+07	2.47E+07	3.21E+07	3.62E+07	2.38E+07	3.42E+07	3.05E+07
Latp004059	C13H14O5	4-(Trans)-acetul-3,6,8-trihydroxy-3-methyl-dihydronapht halenone	Others	Chromone	2.80E+05	2.90E+05	3.50E+05	5.69E+05	4.43E+05	3.76E+05	3.14E+05	3.78E+05	2.89E+05	4.69E+05	4.95E+05	3.91E+05
pme0066	C3H7N3O2	Guanidinoacetate	Alkaloids	Alkaloids	3.34E+04	3.46E+04	4.41E+04	3.50E+04	3.32E+04	3.28E+04	5.22E+04	3.20E+04	3.43E+04	3.82E+04	2.77E+04	3.24E+04
pmn001498	C33H44N2O3	Isodendrocrepine*	Alkaloids	Alkaloids	0.00E+00	0.00E+00	0.00E+00	5.38E+05	9.38E+05	5.31E+05	6.52E+05	8.96E+05	7.31E+05	5.26E+05	8.34E+05	6.88E+05
Wasn003547	C7H16O5S	Heptane-1,7-diol sulfate	Others	Others	1.70E+05	1.18E+05	7.15E+04	3.43E+05	2.17E+05	2.02E+05	3.42E+05	2.07E+05	3.27E+05	3.50E+05	1.78E+05	2.93E+05
Wcdp000839	C6H6O2	5-methylfurfural	Others	Others	7.09E+05	6.08E+05	6.35E+05	2.59E+05	3.05E+05	3.00E+05	1.74E+05	3.03E+05	4.94E+05	2.15E+05	2.94E+05	2.75E+05
Hmdn006855	C30H48O3	3,4-Cycloopen-cycloaltin-4(29),25-diene-24(R)-hydroxy-3-carboxylic acid	Terpenoids	Triterpene	4.04E+03	1.92E+03	2.14E+03	5.11E+03	7.96E+03	1.02E+04	4.37E+03	8.15E+03	7.83E+03	7.59E+03	6.91E+03	7.11E+03
Zmxp004480	C28H32O16	Chrysoeriol-7-O-gentiobioside	Flavonoids	Flavones	3.70E+04	5.02E+04	3.59E+04	3.67E+05	3.53E+05	3.82E+05	3.58E+05	6.70E+05	4.39E+05	4.48E+05	4.22E+05	3.52E+05
Zmsp000237	C11H21NO8	1,4-Dideoxy-1,4-imino-(2-O-β-D-glucosyl)-D-arabinitol	Alkaloids	Pyrrole alkaloids	2.15E+06	2.36E+06	2.36E+06	1.76E+06	1.57E+06	1.71E+06	1.49E+06	1.33E+06	1.75E+06	1.42E+06	1.33E+06	1.33E+06
pme0422	C10H10O4	Isoferulic Acid*	Phenolic acids	Phenolic acids	2.46E+05	1.86E+05	1.76E+05	3.45E+05	4.53E+05	4.57E+05	3.66E+05	4.89E+05	3.58E+05	3.58E+05	4.17E+05	4.60E+05
Lhkp101510	C15H16O5	Hamaudol	Others	Chromone	2.09E+05	2.18E+05	1.95E+05	6.39E+05	1.71E+06	1.36E+06	7.20E+05	2.07E+06	1.44E+06	6.70E+05	1.74E+06	1.41E+06
Zjmp102043	C19H20O6	2,5,7-trihydroxy-6,8-dimethyl-3-(4'-methoxybenzyl)chroman-4-one	Flavonoids	Other Flavonoids	0.00E+00	0.00E+00	0.00E+00	3.51E+04	9.82E+04	3.50E+04	4.48E+04	9.33E+04	2.83E+04	4.35E+04	7.43E+04	3.13E+04
Lmmp003517	C13H20O3	Solatuberenol A	Others	Others	2.48E+05	1.60E+05	1.68E+05	2.12E+05	1.89E+05	2.06E+05	2.25E+05	2.50E+05	1.68E+05	2.35E+05	1.69E+05	2.37E+05
Lmgp004959	C29H34O16	Tricin-7-O-neohesperidoside	Flavonoids	Flavones	8.53E+04	1.13E+05	8.04E+04	1.16E+05	6.70E+04	7.06E+04	8.86E+04	6.18E+04	8.63E+04	1.13E+05	1.41E+05	8.68E+04
Zmbp000490	C8H15NO2	Valerine	Alkaloids	Alkaloids	2.79E+06	2.36E+06	2.22E+06	2.36E+06	2.59E+06	2.58E+06	1.76E+06	1.36E+06	2.29E+06	1.83E+06	2.10E+06	1.66E+06
Wbtn006715	C15H10O7	1,2,4,5,8-pentahydroxy-6-methylanthracene-9,10-dione*	Flavonoids	Other Flavonoids	0.00E+00	0.00E+00	0.00E+00	3.86E+04	1.55E+05	5.14E+04	4.36E+04	1.77E+05	6.55E+04	4.68E+04	1.67E+05	4.22E+04
pmb0818	C11H11NO3	Methoxyindoleacetic acid	Alkaloids	Plumerane	3.30E+05	2.75E+05	2.46E+05	4.58E+05	8.01E+05	5.11E+05	4.94E+05	6.18E+05	5.56E+05	4.38E+05	5.86E+05	4.34E+05
Lmxn008846	C11H18O3	Hydroxydihydrobovolide	Others	Lactones	2.15E+04	1.96E+04	1.22E+04	4.02E+04	3.86E+04	2.91E+04	3.41E+04	4.72E+04	3.81E+04	2.88E+04	4.13E+04	4.42E+04
Hmsn002272	C15H20O8	Demethyl coniferin	Phenolic acids	Phenolic acids	6.00E+05	5.65E+05	6.27E+05	1.16E+06	1.22E+06	1.28E+06	1.44E+06	1.21E+06	1.37E+06	1.24E+06	1.35E+06	1.39E+06
Wmtn012065	C15H30N2O	Piperazirum	Alkaloids	Alkaloids	0.00E+00	0.00E+00	0.00E+00	9.07E+05	1.09E+06	9.13E+05	8.84E+05	1.11E+06	1.02E+06	8.42E+05	1.16E+06	9.64E+05
Jmmn008945	C29H46O6	2 α,3 α,19 α,23-tetrahydroxyursolane-12-en-28-oic acid	Terpenoids	Triterpene	0.00E+00	0.00E+00	0.00E+00	1.12E+04	1.41E+04	1.02E+04	1.18E+04	7.89E+03	7.33E+03	8.68E+03	1.09E+04	9.56E+03
Zjhp082617	C33H50O9	Gentrogenin-Glc	Steroids	Steroidal saponins	2.09E+07	1.71E+07	3.30E+07	7.37E+07	7.46E+07	6.08E+07	7.24E+07	5.59E+07	5.75E+07	6.45E+07	5.11E+07	9.80E+07
HX1356	C22H24O7	(7R,8S)-9-acetyl-dehydrodiconiferyl alcohol	ignans and Coumarir	Lignans	1.48E+05	1.63E+05	1.31E+05	1.42E+05	3.22E+05	1.93E+05	1.94E+05	2.63E+05	1.87E+05	1.62E+05	2.82E+05	2.50E+05
MWSHC2022	C15H20O10	Glucosyringic acid	Phenolic acids	Phenolic acids	2.94E+06	2.74E+06	3.02E+06	3.86E+06	4.30E+06	3.77E+06	3.22E+06	4.07E+06	3.56E+06	3.24E+06	3.95E+06	3.45E+06
Yamp002097	C10H11NO3	1-[2-(Furan-2-yl)-2-oxoethyl]pyrrolidin-2-One	Alkaloids	Alkaloids	5.33E+06	5.03E+06	4.97E+06	5.83E+06	6.24E+06	5.45E+06	5.19E+06	5.16E+06	4.85E+06	4.96E+06	5.00E+06	5.05E+06
Zjzp081815	C39H64O13	Sarsasapogenin-3-O-glucosyl(1→2)mannoside (Timosaponin A-IV)	Steroids	Steroidal saponins	6.91E+05	5.47E+05	6.33E+05	1.36E+06	1.57E+06	1.49E+06	1.53E+06	1.43E+06	1.80E+06	1.94E+06	2.08E+06	1.61E+06
Qmgp102028	C33H53NO7	Isorubijervosine	Alkaloids	Steroid alkaloids	5.87E+04	6.69E+04	6.28E+04	2.70E+05	2.58E+05	3.31E+05	2.91E+05	3.45E+05	2.37E+05	1.39E+05	2.30E+05	2.75E+05
Wdbp008550	C18H20O4	Stemanthrene C	Others	Others	1.98E+05	1.57E+05	1.89E+05	1.83E+05	1.95E+05	1.52E+05	2.08E+05	1.97E+05	2.59E+05	2.06E+05	1.72E+05	1.95E+05
Wbwn009791	C24H30O8	Marlignan E	ignans and Coumarir	Lignans	2.38E+05	2.30E+05	2.47E+05	3.68E+05	4.19E+05	3.27E+05	3.17E+05	4.55E+05	3.90E+05	4.08E+05	3.52E+05	3.08E+05
Lmbn002644	C8H8O	4-Methylbenzaldehyde	Others	Aldehyde compounds	8.06E+04	6.74E+04	7.58E+04	1.04E+05	1.09E+05	7.34E+04	8.83E+04	1.32E+05	6.04E+04	9.50E+04	7.88E+04	8.69E+04
Wdhp006324	C17H17NO3	(2e)-3-(4-hydroxyphenyl)-n-[2-(4-hydroxyphenyl)ethyl]prop-2-enimidic acid	Alkaloids	Isoquinoline alkaloids	0.00E+00	0.00E+00	0.00E+00	1.30E+07	1.74E+07	1.73E+07	1.08E+07	1.66E+07	1.80E+07	1.02E+07	1.61E+07	1.76E+07
MWSslk106	C8H11N	2-Phenylethylamine	Alkaloids	lphenylethylamine alk	8.47E+05	6.79E+05	7.40E+05	1.18E+06	1.68E+06	1.48E+06	1.08E+06	1.19E+06	1.20E+06	1.24E+06	1.42E+06	1.22E+06
Wbsp001938	C9H17NO5	Casuarine Analogue	Alkaloids	Alkaloids	8.42E+05	7.99E+05	7.37E+05	9.18E+05	1.00E+06	8.30E+05	8.90E+05	9.27E+05	8.95E+05	9.86E+05	9.57E+05	8.15E+05
MWStz040	C5H9NO2	Pterolactam	Alkaloids	Pyrrole alkaloids	3.75E+06	3.48E+06	3.54E+06	2.61E+06	3.08E+06	2.82E+06	2.15E+06	2.68E+06	2.64E+06	2.16E+06	2.78E+06	2.41E+06
Zjmp102044	C17H16O6	5,7-dihydroxy-6-methyl-3(R)-(2,4-dihydroxybenzyl)chroman-4-one*	Flavonoids	Other Flavonoids	0.00E+00	0.00E+00	0.00E+00	2.74E+04	6.14E+05	2.51E+05	2.60E+04	8.99E+05	2.61E+05	3.53E+04	8.78E+05	2.56E+05
Lmmn002260	C27H36O12	5'-Methoxyisolariciresinol-9'-O-glucoside	ignans and Coumarir	Lignans	3.99E+05	3.93E+05	3.78E+05	3.83E+05	4.82E+05	4.06E+05	3.46E+05	5.32E+05	3.94E+05	3.62E+05	5.53E+05	3.87E+05
Zjhp082628	C39H62O13	Diosgenin-Gal-Glc	Steroids	Steroidal saponins	2.91E+06	3.57E+06	3.04E+06	1.72E+07	2.06E+07	2.04E+07	2.28E+07	2.58E+07	2.23E+07	1.52E+07	1.83E+07	1.51E+07
Wcsn010375	C18H30O2	Methyl (4E,8E,11E)-5,9,13-trimethyltetradeca-4,8,11-trienoate	Others	Others	0.00E+00	0.00E+00	0.00E+00	1.95E+05	1.92E+05	1.73E+05	1.96E+05	2.17E+05	1.98E+05	2.22E+05	2.40E+05	1.84E+05
MWSslk051	C8H13NO2	Scopine	Alkaloids	Tropan alkaloids	1.70E+06	1.70E+06	1.79E+06	2.35E+06	2.41E+06	2.47E+06	2.12E+06	2.47E+06	2.40E+06	2.10E+06	2.31E+06	2.29E+06
Wagp010326	C22H20O9	3,5-Diacetyltambulin	Flavonoids	Flavones	3.13E+06	3.90E+06	2.17E+06	7.61E+06	8.04E+06	7.99E+06	7.40E+06	8.57E+06	6.53E+06	7.62E+06	8.14E+06	9.14E+06
Wafp004987	C18H17NO4	N-Feruloylphenylacetamide	Alkaloids	Phenolamine	8.04E+05	6.27E+05	5.89E+05	1.16E+07	9.09E+06	1.19E+07	8.62E+06	8.69E+06	1.06E+07	9.09E+06	9.47E+06	8.71E+06
Wbmn002185	C20H14O7	6-Hydroxy-4-(4-hydroxy-3-methoxyphenyl)-7-methoxy-naphtho[2,3-c]furan-1,3-dione-1(ISO-1)	ignans and Coumarir	Lignans	9.28E+04	5.90E+04	8.97E+04	1.25E+05	1.10E+05	1.49E+05	1.44E+05	1.27E+05	1.21E+05	1.34E+05	1.08E+05	1.10E+05
Wbmp003697	C13H20O2	(R)-3-(3'-Hydroxybutyl)-2,4,4-trimethylcyclohexa-2,5-dienone	Terpenoids	Sesquiterpenoids	6.82E+04	7.29E+04	8.02E+04	1.18E+05	1.08E+05	9.13E+04	8.93E+04	1.14E+05	1.32E+05	9.54E+04	1.13E+05	1.27E+05
MWStz221	C18H19NO4	N-Cis-Feruloyltyramine*	Alkaloids	Phenolamine	1.68E+05	2.23E+05	1.78E+05	3.86E+07	5.12E+07	5.11E+07	4.16E+07	5.22E+07	4.91E+07	3.87E+07	5.04E+07	4.40E+07
Wcfp003695	C13H18O2	Oleracone B	Others	Others	5.68E+05	4.50E+05	5.63E+05	7.80E+05	7.37E+05	5.94E+05	5.91E+05	6.37E+05	6.45E+05	5.85E+05	6.14E+05	7.18E+05
Hmln003169	C21H24O7	Trachelogenin	ignans and Coumarir	Lignans	1.27E+05	9.98E+04	9.61E+04	3.48E+05	7.11E+05	5.41E+05	3.75E+05	6.40E+05	5.37E+05	3.97E+05	6.97E+05	6.03E+05
Wasn001446	C14H26O10	Glucosyl 6-methylhept-6-ene-1,2,3,4,5-pentaol	Others	Others	1.89E+06	1.98E+06	1.83E+06	1.24E+06	1.39E+06	1.11E+06	1.04E+06	1.10E+06	9.57E+05	1.23E+06	8.75E+05	1.47E+06
pmn001573	C23H26O10	Desmethylagrimonolide-6-O-glucoside*	Phenolic acids	Phenolic acids	0.00E+00	0.00E+00	0.00E+00	6.08E+04	4.70E+05	3.02E+05	6.83E+04	4.94E+05	3.57E+05	3.79E+04	5.58E+05	3.08E+05
pma6298	C5H5NO	3-Hydroxypyridine	Alkaloids	Pyridine alkaloids	5.26E+05	5.03E+05	3.79E+05	5.36E+05	4.82E+05	5.21E+05	6.53E+05	4.74E+05	4.98E+05	5.11E+05	5.01E+05	5.01E+05
Wmxp009647	C20H26O3	Momilactone A	Terpenoids	Ditepenoids	1.10E+05	9.21E+04	1.24E+05	8.22E+05	2.18E+06	1.59E+05	5.21E+05	6.05E+05	4.80E+05	2.13E+05	1.55E+05	1.72E+05
Lmyp004963	C27H30O14	Apigenin-6-C-(2''-rhamnosyl)glucoside	Flavonoids	Flavones	4.58E+04	2.42E+04	3.65E+04	7.14E+04	8.43E+04	1.47E+05	1.05E+05	1.44E+05	8.49E+04	1.13E+05	8.27E+04	1.16E+05
Zjmp101909	C39H62O13	isodiosgenin-Glc-Glc	Steroids	Steroidal saponins	4.83E+05	4.83E+05	3.70E+05	1.77E+06	1.89E+06	1.95E+06	1.99E+06	1.45E+06	2.27E+06	2.38E+06	1.93E+06	1.65E+06
Wafp004641	C24H29NO9	N-Feruloyltyramine 4-glucoside	Alkaloids	Phenolamine	9.53E+05	9.33E+05	1.16E+06	3.55E+06	5.67E+06	4.46E+06	2.83E+06	5.54E+06	4.21E+06	2.77E+06	4.93E+06	4.33E+06
MWS20147	C21H20O11	Luteolin-3'-O-	Flavonoids	Flavones	1.53E+04	3.18E+03	2.34E+03	3.58E+04	7.81E+04	6.04E+04	7.11E+04	8.66E+04	8.61E+04	2.05E+04	3.71E+04	6.13E+04
Zjhp090618	C18H18O6	5,7-Dihydroxy-6,8-dimethyl-3-(2',4'-hydroxybenzyl)chroman-4-One*	Flavonoids	Other Flavonoids	0.00E+00	0.00E+00	0.00E+00	8.47E+05	1.69E+06	1.56E+06	9.72E+05	1.57E+06	1.55E+06	9.66E+05	1.68E+06	1.32E+06
Jmwn002117	C14H20O9	2-(3,4-dihydroxyphenyl)ethanediol 1-O-β-D-	Phenolic acids	Phenolic acids	3.26E+05	3.31E+05	3.01E+05	3.81E+05	4.18E+05	4.88E+05	3.87E+05	4.42E+05	4.48E+05	3.75E+05	4.79E+05	4.29E+05

		glucopyranoside												
pmb0758	C24H26O11	Anisic acid-O-feruloyl glucoside	Phenolic acids	Phenolic acids	3.24E+05	3.11E+05	3.05E+05	7.51E+05	5.31E+05	6.80E+05	7.09E+05	5.11E+05	7.39E+05	6.40E+05	5.17E+05	5.68E+05
Zjmp101931	C39H62O14	25(R)-ruscogenin-Glc-Glc	Steroids	Steroidal saponins	4.04E+04	2.40E+04	3.16E+04	1.62E+05	2.30E+05	2.48E+05	2.72E+05	2.18E+05	2.95E+05	3.12E+05	2.60E+05	2.84E+05
mws0914	C15H12O5	3,5,7-Trihydroxyflavanone (Pinobanksin)*	Flavonoids	Flavanonols	0.00E+00	0.00E+00	0.00E+00	6.41E+04	3.22E+05	1.83E+05	5.72E+04	3.31E+05	2.11E+05	5.52E+04	3.47E+05	1.47E+05
mws0025	C6H6O3	Pyrogallol	Phenolic acids	Phenolic acids	3.82E+05	3.58E+05	3.82E+05	5.99E+05	6.77E+05	7.48E+05	6.23E+05	6.66E+05	6.79E+05	6.06E+05	6.40E+05	6.46E+05
MWS5206	C9H10O4	4-Hydroxyphenyllactic Acid	Phenolic acids	Phenolic acids	4.91E+04	4.51E+04	3.03E+04	2.55E+05	2.87E+05	3.02E+05	2.87E+05	2.70E+05	2.63E+05	2.85E+05	2.63E+05	2.67E+05
Zjhp082688	C51H82O23	Spirost-5-en-3-ol-3-O-glucosyl(1→2)[glucosyl(1→3)]glucosyl(1→4) galactoside	Steroids	Steroidal saponins	1.56E+05	1.38E+05	1.58E+05	5.43E+05	5.77E+05	9.02E+05	4.73E+05	5.23E+05	9.99E+05	7.83E+05	8.32E+05	1.22E+06
Wmmn000125	C24H30O13	mudanpioside E	Others	Others	5.42E+04	5.30E+04	3.73E+04	1.16E+05	1.22E+05	9.26E+04	1.30E+05	7.63E+04	1.33E+05	1.08E+05	1.27E+05	7.57E+04
MWSslk035	C13H18O7	Orcinol glucoside	Phenolic acids	Phenolic acids	2.19E+06	1.61E+06	1.52E+06	3.19E+06	4.62E+06	2.26E+06	4.37E+06	4.43E+06	2.50E+06	3.22E+06	4.14E+06	2.48E+06
Qagp006800	C36H36N2O8	Lyciumamide B	Alkaloids	Phenolamine	0.00E+00	0.00E+00	0.00E+00	1.59E+06	2.22E+06	2.10E+06	1.78E+06	2.28E+06	2.32E+06	1.89E+06	2.32E+06	2.05E+06
Wcdp006741	C26H50NO7P	1-(9Z,12Z-Octadecadienoyl)-Sn-Glycero-3-Phosphocholine	Others	Others	2.60E+04	2.34E+04	4.63E+04	5.60E+06	6.19E+06	6.74E+06	5.99E+06	6.46E+06	6.02E+06	5.92E+06	6.84E+06	6.95E+06
Lmnn102603	C18H28O9	Nuezhengalaside	Terpenoids	Monoterpenoids	9.39E+04	7.67E+04	6.21E+04	1.96E+05	1.17E+05	1.15E+05	1.58E+05	1.31E+05	1.46E+05	1.34E+05	1.40E+05	1.64E+05
pme2292	C4H12N2	Putrescine	Alkaloids	Alkaloids	3.00E+05	2.57E+05	2.92E+05	3.57E+05	5.26E+05	4.44E+05	3.29E+05	5.40E+05	4.60E+05	3.57E+05	5.62E+05	4.02E+05
mws0018	C10H26N4	Spermine	Alkaloids	Alkaloids	4.83E+05	4.44E+05	4.99E+05	5.45E+05	6.60E+05	6.28E+05	5.03E+05	6.85E+05	6.02E+05	6.61E+05	7.63E+05	6.24E+05
Wabn003563	C17H22O10	3'-O-Beta-D-Glucopyranosyl plumbagic acid	Phenolic acids	Phenolic acids	1.46E+05	1.14E+05	1.01E+05	1.49E+05	9.99E+04	1.06E+05	1.14E+05	9.82E+04	1.41E+05	1.23E+05	1.23E+05	1.29E+05
Hahn002315	C28H36O13	syringaresinol-4-O-β-D-glucopyranoside*	ignans and Coumarir	Lignans	3.32E+05	2.45E+05	5.83E+05	4.71E+05	6.53E+05	6.05E+05	5.36E+05	9.92E+05	3.62E+05	5.56E+05	7.50E+05	6.02E+05
Lmrn003000	C9H10O3	2-Hydroxy-3-phenylpropanoic acid	Phenolic acids	Phenolic acids	1.70E+04	1.07E+04	2.24E+04	9.62E+04	1.54E+05	1.37E+05	1.16E+05	1.61E+05	1.29E+05	1.03E+05	1.33E+05	1.35E+05
Qadp004840	C56H90O27	Typaspidoside L	Steroids	Steroidal saponins	1.28E+04	4.52E+04	5.28E+04	3.30E+05	4.05E+05	1.31E+05	2.89E+05	4.41E+05	2.33E+05	3.76E+05	3.86E+05	3.71E+05
Zbzp000519	C8H13NO2	Retronecine	Alkaloids	Pyrrole alkaloids	4.28E+06	4.58E+06	4.59E+06	3.08E+06	3.30E+06	2.91E+06	2.88E+06	3.09E+06	2.17E+06	2.63E+06	3.05E+06	2.45E+06
mws4113	C21H22O5	3'-Hydroxy-4'-O-methylglabridin	Flavonoids	Other Flavonoids	0.00E+00	0.00E+00	0.00E+00	3.70E+04	3.75E+04	4.38E+04	4.50E+04	4.33E+04	4.93E+04	2.96E+04	4.30E+04	4.43E+04
Lazn004796	C20H24O8	Demethyl-erythro-Guaiacylglycerol β-Sinapyl Ether	ignans and Coumarir	Lignans	1.25E+05	1.16E+05	1.12E+05	1.60E+05	1.55E+05	1.63E+05	1.69E+05	1.80E+05	1.45E+05	1.62E+05	1.54E+05	1.52E+05
Layn004158	C18H26O10	Purpuroside	Phenolic acids	Phenolic acids	2.34E+05	2.35E+05	2.44E+05	5.60E+05	5.60E+05	5.40E+05	6.24E+05	5.59E+05	5.30E+05	5.75E+05	4.46E+05	5.80E+05
Wcsn011125	C22H34O4	Ethyl (4E,6E,8E,10E,13Z)-15,16-dihydroxy-5,9-dimethyloctadeca-4,6,8,10,13-pentaenoate	Others	Others	1.50E+05	1.17E+05	8.47E+04	2.49E+07	2.25E+07	2.10E+07	2.30E+07	2.21E+07	2.16E+07	2.43E+07	2.40E+07	2.29E+07
Zjmp102003	C18H18O5	iso-ophiopogonanone B*	Flavonoids	Other Flavonoids	0.00E+00	0.00E+00	0.00E+00	2.37E+05	3.50E+05	2.88E+05	2.61E+05	3.84E+05	3.26E+05	2.63E+05	3.69E+05	3.03E+05
HJN011	C17H17NO4	Tataramide A	Alkaloids	Phenolamine	3.50E+04	2.50E+04	2.06E+04	8.39E+05	1.63E+06	1.37E+06	9.00E+05	1.61E+06	1.18E+06	8.86E+05	1.39E+06	1.17E+06
Lmjn102210	C23H26O10	Desmethylagrimonolide-6-O-β-D-glucopyranoside*	ignans and Coumarir	Coumarins	0.00E+00	0.00E+00	0.00E+00	4.90E+04	3.91E+05	3.08E+05	5.50E+04	3.88E+05	3.16E+05	5.13E+04	4.96E+05	2.15E+05
MW0112082	C8H9NO	2-Aminoacetophenone	Others	Ketone compounds	5.75E+05	8.98E+05	7.22E+05	5.82E+06	7.93E+06	6.06E+06	6.27E+06	8.24E+06	6.25E+06	6.60E+06	9.28E+06	5.80E+06
Zbsp007084	C15H12O5	Butin; 7,3',4'-Trihydroxyflavanone	Flavonoids	Flavanones	0.00E+00	0.00E+00	0.00E+00	8.81E+04	3.80E+05	2.18E+05	6.86E+04	4.04E+05	2.65E+05	7.38E+04	3.84E+05	2.31E+05
MWSHC20164	C16H22O4	Dibutyl phthalate*	Phenolic acids	Phenolic acids	5.79E+06	6.04E+06	7.19E+06	5.03E+06	6.95E+06	6.92E+06	5.43E+06	5.03E+06	6.93E+06	5.16E+06	6.53E+06	5.96E+06
Zahn007990	C11H18O3	5-hydroxy-3,4-dimethyl-5-pentylfuran-2(5H)-one	Others	Others	2.13E+04	1.91E+04	2.47E+04	4.23E+04	5.03E+04	4.61E+04	3.95E+04	4.47E+04	4.34E+04	4.73E+04	4.70E+04	4.53E+04
pmn001681	C10H14O2	1-(4-Methoxyphenyl)-1-propanol	Phenolic acids	Phenolic acids	1.90E+05	1.92E+05	1.67E+05	1.50E+05	1.65E+05	1.66E+05	1.63E+05	1.36E+05	1.43E+05	1.41E+05	1.15E+05	1.16E+05
Lacp001431	C14H21NO4	N-(3-hydroxy-4-methoxyphenethyl)-4-hydroxybutanamide	Alkaloids	Phenolamine	8.09E+06	6.73E+06	6.75E+06	1.15E+07	1.48E+07	1.46E+07	1.36E+07	1.80E+07	1.73E+07	1.50E+07	1.80E+07	1.83E+07
Lmjn010508	C17H26O3	Fusarester B	Others	Ketone compounds	1.46E+06	1.50E+06	1.57E+06	1.27E+06	1.29E+06	1.33E+06	1.37E+06	1.29E+06	1.26E+06	1.28E+06	1.29E+06	1.32E+06
Hmjp005556	C17H17NO3	(2E)-3-(4-Hydroxyphenyl)-N-[2-(4-hydroxyphenyl)ethyl]-2-propenamide	Alkaloids	Alkaloids	2.23E+06	2.39E+06	2.32E+06	1.06E+07	8.76E+06	1.14E+07	8.20E+06	9.17E+06	9.52E+06	6.75E+06	6.60E+06	9.87E+06
Hmcn002162	C26H40O11	Shinjuglycoside E	Terpenoids	Ditepenoids	1.54E+05	9.19E+04	1.00E+05	4.46E+05	3.49E+05	4.03E+05	4.93E+05	3.60E+05	4.34E+05	4.27E+05	3.44E+05	3.59E+05
mws1346	C6H11NO4	DL-2-Aminoadipic	Alkaloids	Alkaloids	8.72E+05	8.72E+05	9.28E+05	6.18E+05	5.60E+05	5.76E+05	5.86E+05	5.35E+05	5.80E+05	7.18E+05	7.59E+05	6.91E+05
Lmrn001951	C9H10O4	(S)-2-Hydroxy-3-(4-Hydroxyphenyl)Propanoic Acid	Phenolic acids	Phenolic acids	3.24E+04	3.91E+04	2.44E+04	2.56E+05	2.54E+05	2.84E+05	2.27E+05	2.70E+05	2.71E+05	2.64E+05	2.61E+05	2.40E+05
Lakn000379	C28H42O18	2,5-Dihydroxyphenylacetate ethyl triglucoside	Phenolic acids	Phenolic acids	1.20E+05	1.48E+05	1.06E+05	2.62E+05	2.21E+05	1.88E+05	1.82E+05	1.89E+05	1.99E+05	2.13E+05	1.86E+05	2.17E+05
pma0675	C8H8O3	o-Anisic acid	Phenolic acids	Phenolic acids	1.99E+05	1.56E+05	2.24E+05	2.54E+05	2.12E+05	1.87E+05	1.57E+05	2.16E+05	1.84E+05	1.82E+05	1.63E+05	1.75E+05
Zaxn006988	C20H30O2	Isopimaric acid*	Terpenoids	Ditepenoids	3.65E+05	6.32E+05	6.22E+05	3.62E+06	9.41E+06	1.78E+06	4.04E+06	3.08E+06	2.79E+06	2.41E+06	1.13E+06	8.95E+05
Zjhp082614	C33H52O8	iso-Diosgenin-Glc*	Steroids	Steroidal saponins	2.93E+06	2.67E+06	2.22E+06	2.09E+07	2.23E+07	2.35E+07	1.99E+07	2.02E+07	2.00E+07	1.64E+07	1.64E+07	1.78E+07
Qmgp102003	C29H41NO4	O-Acetyljervine	Alkaloids	Steroid alkaloids	6.10E+04	6.14E+04	8.37E+04	1.89E+06	1.75E+06	1.83E+06	1.80E+06	1.96E+06	2.00E+06	1.70E+06	2.13E+06	1.86E+06
pmp001230	C16H22O4	Senkyunolide M	Others	Lactones	3.26E+07	3.94E+07	5.58E+07	3.42E+07	4.48E+07	3.25E+07	4.17E+07	4.31E+07	4.11E+07	3.60E+07	4.21E+07	5.32E+07
mws0639	C7H6O4	2,3-Dihydroxybenzoic Acid*	Phenolic acids	Phenolic acids	6.50E+05	5.06E+05	5.77E+05	7.69E+05	1.08E+06	9.73E+05	8.34E+05	9.68E+05	1.01E+06	8.89E+05	9.99E+05	9.32E+05
Li512115	C25H24O12	Isochlorogenic acid B	Phenolic acids	Phenolic acids	4.68E+04	2.07E+04	2.83E+04	7.48E+04	1.02E+05	6.56E+04	7.99E+04	8.15E+04	4.92E+04	6.66E+04	1.04E+05	6.66E+04
Lazn004665	C15H21N5O4	Triacanthinapioside	Alkaloids	Alkaloids	2.03E+05	1.43E+05	1.34E+05	9.93E+05	6.41E+05	9.59E+05	9.64E+05	6.98E+05	9.49E+05	9.03E+05	8.05E+05	9.68E+05
Wbtn014060	C23H22O5	Nigrolineaxanthone K	Flavonoids	Other Flavonoids	0.00E+00	0.00E+00	0.00E+00	5.24E+04	4.44E+04	5.17E+04	7.09E+04	6.04E+04	5.88E+04	5.40E+04	6.01E+04	5.50E+04
Wbkn004126	C26H36O11	Javanicolide C	Terpenoids	Ditepenoids	3.02E+05	3.46E+05	2.97E+05	6.80E+05	7.71E+05	5.03E+05	5.73E+05	6.09E+05	5.53E+05	6.84E+05	4.61E+05	6.07E+05
pmb2999	C22H22O11	Chrysoeriol-5-O-glucoside	Flavonoids	Flavones	4.09E+04	5.19E+04	4.14E+04	2.00E+05	8.70E+05	5.46E+05	1.23E+05	8.27E+05	4.97E+05	1.51E+05	7.32E+05	5.05E+05
Lazn006513	C33H40O13	(1R,2S)-2-[4-[(3R,3aR,6R,6aS)-3-(4-Hydroxy-3,5-dimethoxyphenyl)-1,3,3a,4,6,6a-hexahydrofuro[3,4-c]furan-6-yl]-2,6-dimethoxyphenoxy]-1-(4-hydroxy-3,5-dimethoxyphenyl)propane-1,3-diol	ignans and Coumarir	Lignans	5.11E+04	3.95E+04	3.73E+04	9.68E+04	8.51E+04	9.22E+04	9.98E+04	8.55E+04	1.02E+05	9.45E+04	1.11E+05	1.18E+05
pmb0492	C34H37N3O6	N',N'',N'''-p-Coumaroyl-cinnamoyl-caffeoyl spermidine	Alkaloids	Phenolamine	1.85E+05	2.09E+05	1.53E+05	4.53E+05	6.13E+05	4.87E+05	3.97E+05	5.64E+05	5.76E+05	4.39E+05	6.19E+05	4.54E+05
mws0145	C8H8O3	O-Anisic acid (2-Methoxybenzoic acid)	Phenolic acids	Phenolic acids	1.27E+05	1.03E+05	1.32E+05	1.16E+05	1.74E+05	1.66E+05	1.16E+05	1.70E+05	1.33E+05	1.36E+05	2.13E+05	1.64E+05
pme0282	C8H6O4	Phthalic acid	Phenolic acids	Phenolic acids	7.19E+04	7.29E+04	7.52E+04	1.11E+05	1.56E+05	7.94E+04	9.32E+04	1.32E+05	1.12E+05	9.41E+04	9.55E+04	9.53E+04
Hmlp003068	C27H30O17	Meratin*	Flavonoids	Flavones	3.72E+05	4.10E+05	3.43E+05	1.26E+06	1.67E+06	1.38E+06	1.19E+06	1.23E+06	1.20E+06	1.22E+06	1.27E+06	1.00E+06
Lcsn000191	C24H30O13	isatioxyneolignoside A	ignans and Coumarir	Lignans	6.07E+05	5.28E+05	4.66E+05	1.32E+06	1.15E+06	1.31E+06	1.38E+06	1.11E+06	1.29E+06	1.40E+06	1.13E+06	1.20E+06
Zahp005843	C19H21NO5	N-Trans-Sinapoyltyramine	Alkaloids	Phenolamine	6.51E+04	6.93E+04	6.37E+04	1.92E+06	2.95E+06	2.93E+06	2.04E+06	3.00E+06	2.79E+06	2.17E+06	2.76E+06	2.37E+06
mws1433	C18H19NO4	N-Feruloyltyramine; Moupinamide*	Alkaloids	Phenolamine	1.44E+05	1.36E+05	2.06E+05	3.79E+07	5.22E+07	4.95E+07	3.90E+07	5.04E+07	4.72E+07	4.13E+07	5.08E+07	4.47E+07
mws0103	C9H7NO	Indole-3-carboxaldehyde	Alkaloids	Plumerane	8.58E+05	9.08E+05	9.56E+05	1.34E+06	1.22E+06	1.35E+06	1.33E+06	1.27E+06	1.45E+06	1.43E+06	1.51E+06	1.41E+06
MWSprf075	C16H22O7	Raspberryketone glucoside	Phenolic acids	Phenolic acids	8.93E+04	8.19E+04	5.67E+04	2.23E+05	3.80E+05	3.18E+05	2.36E+05	4.26E+05	3.46E+05	2.80E+05	4.89E+05	2.35E+05
Hmcp001579	C29H34O18	Limocitrin-3-O-sophoroside*	Flavonoids	Flavonols	7.92E+04	7.97E+04	8.87E+04	1.94E+05	1.74E+05	1.81E+05	2.03E+05	2.57E+05	1.93E+05	2.15E+05	1.77E+05	2.55E+05
Yamn004462	C26H36O11	6-((4-(3-hydroxy-2-(4-(3-hydroxypropyl)-2-methylphenoxy)propoxy)-2-methoxyphenoxy)methyl)tetrahydro-2H-pyran-2,3,4,5-tetraol	ignans and Coumarir	Lignans	3.34E+05	3.61E+05	3.79E+05	1.01E+06	6.14E+05	7.97E+05	6.40E+05	7.23E+05	9.46E+05	8.52E+05	6.46E+05	7.41E+05
MWSprf162	C27H38O4	(25R)-Spirost-4-ene-3,12-dione	Steroids	Steroidal saponins	0.00E+00	0.00E+00	0.00E+00	6.26E+04	5.66E+04	4.91E+04	5.72E+04	5.46E+04	4.87E+04	6.92E+04	5.72E+04	3.94E+04
Wcfp005519	C9H6O3	5-hydroxyindene-1,3-dione	Terpenoids	Sesquiterpenoids	7.72E+06	7.95E+06	7.68E+06	6.66E+06	5.80E+06	5.78E+06	5.89E+06	5.07E+06	5.43E+06	5.26E+06	4.79E+06	4.95E+06
Hmln002100	C26H32O12	Nortrachelogenin-4-O-glucoside	ignans and Coumarir	Lignans	1.17E+05	8.27E+04	7.88E+04	9.34E+04	1.37E+05	1.09E+05	1.17E+05	1.52E+05	1.21E+05	7.07E+04	1.28E+05	1.05E+05
MWSslk097	C10H10O4	Dimethyl phthalate	Phenolic acids	Phenolic acids	5.96E+06	5.73E+06	6.67E+06	5.85E+06	4.92E+06	5.21E+06	5.44E+06	5.75E+06	5.47E+06	5.71E+06	5.93E+06	6.14E+06
Lamn006238	C23H26O11	3,9-dihydroeucomnalin glucoside	Flavonoids	Other Flavonoids	2.43E+04	4.92E+04	3.36E+04	1.26E+05	1.53E+05	1.52E+05	1.58E+05	1.34E+05	1.95E+05	1.38E+05	2.16E+05	1.90E+05
Zaxn005783	C21H32O2	Methyl neoabietate	Terpenoids	Ditepenoids	2.51E+04	2.10E+04	1.61E+04	1.73E+05	4.44E+05	1.95E+05	1.55E+05	1.69E+05	1.36E+05	0.00E+00	0.00E+00	0.00E+00
Zasp102439	C8H7N	m-Aminophenylacetylene	Alkaloids	Alkaloids	2.28E+06	2.54E+06	2.32E+06	2.34E+06	2.88E+06	2.52E+06	2.43E+06	2.77E+06	2.55E+06	2.25E+06	2.61E+06	2.35E+06
Hmhp006887	C18H18O5	6,8-Dihydroxy-2-(4-hydroxybenzyl)-5,7-dimethyl-3,4-dihydronaphthalen-1(2H)-one*	Flavonoids	Other Flavonoids	0.00E+00	0.00E+00	0.00E+00	2.54E+05	2.18E+06	1.02E+06	2.70E+05	2.52E+06	1.09E+06	3.08E+05	2.44E+06	9.33E+05
pmb3074	C16H18O8	5-O-p-Coumaroylquinic acid	Phenolic acids	Phenolic acids	2.49E+04	2.27E+04	1.64E+04	5.07E+04	4.55E+04	3.90E+04	4.61E+04	6.01E+04	4.02E+04	3.76E+04	5.38E+04	3.41E+04
Zbsp003407	C27H30O15	Apigenin-6,8-di-C-glucoside (Vicenin-2)	Flavonoids	Flavones	6.54E+04	3.84E+04	3.89E+04	1.89E+05	1.18E+05	1.43E+05	1.63E+05	1.58E+05	1.40E+05	1.36E+05	1.09E+05	1.44E+05
Hajp009554	C16H14O4	2',4'-Dihydroxy-4-methoxychalcone; (E)-Cardamonin	Flavonoids	Chalcones	0.00E+00	0.00E+00	0.00E+00	1.89E+04	1.16E+06	2.08E+05	1.90E+04	1.46E+06	2.52E+05	1.50E+04	1.36E+06	2.12E+05
MWS20177	C15H8O5	5-hydroxy-anthraquinone-2-carboxylic acid	Quinones	Anthraquinone	0.00E+00	0.00E+00	0.00E+00	5.99E+04	8.28E+04	7.59E+04	7.22E+04	1.00E+05	8.19E+04	5.93E+04	9.89E+04	5.32E+04
pmb2497	C9H10O5	4-Hydroxy-3-methoxymandelate	Phenolic acids	Phenolic acids	4.91E+04	3.12E+04	3.09E+04	7.60E+04	1.22E+05	1.01E+05	7.40E+04	1.14E+05	1.11E+05	1.14E+05	1.14E+05	9.69E+04
Lakn003294	C15H20O9	2-β-D-Glucopyranosyloxy-5-hydroxyphenylacetic acidmethylester	Phenolic acids	Phenolic acids	8.96E+05	6.13E+05	6.51E+05	7.49E+05	1.16E+06	1.07E+06	8.18E+05	1.23E+06	9.32E+05	7.82E+05	1.09E+06	1.26E+06
MWSmce040	C8H8O3	Isovanillin	Others	Aldehyde compounds	2.17E+06	1.74E+06	2.10E+06	1.93E+06	1.78E+06	1.68E+06	1.77E+06	1.82E+06	1.63E+06	1.54E+06	1.66E+06	1.78E+06
Wcjp002598	C9H7NO	8-hydroxyquinoline	Alkaloids	Quinoline alkaloids	9.74E+05	9.61E+05	6.79E+05	1.17E+06	2.24E+06	1.85E+06	1.34E+06	2.26E+06	1.50E+06	1.37E+06	2.02E+06	1.53E+06
Zmjn003003	C9H10O4	3,4-Dimethoxybenzoic acid; Veratric acid	Phenolic acids	Phenolic acids	4.98E+04	4.92E+04	5.31E+04	1.11E+05	1.40E+05	1.18E+05	1.02E+05	1.06E+05	9.52E+04	1.06E+05	1.00E+05	1.15E+05
Lmjp003295	C22H22O12	6-Methoxykaempferol-3-O-glucoside	Flavonoids	Flavonols	3.36E+04	6.77E+04	6.76E+04	4.94E+05	6.89E+05	7.78E+05	3.42E+05	7.43E+05	9.49E+05	4.73E+05	8.71E+05	8.31E+05
Wbmn010746	C22H34O5	Negundoin A	Terpenoids	Ditepenoids	0.00E+00	0.00E+00	0.00E+00	5.69E+06	5.44E+06	6.07E+06	5.11E+06	4.31E+06	5.60E+06	4.42E+06	5.10E+06	5.25E+06
Lmmn004685	C20H24O5	Nectandrin B	ignans and Coumarir	Lignans	2.51E+04	1.77E+04	1.95E+04	1.17E+05	1.34E+05	1.13E+05	1.01E+05	1.26E+05	1.15E+05	1.02E+05	1.47E+05	1.27E+05
pme1002	C8H11NO	L-Tyramine	Alkaloids	Alkaloids	1.95E+06	1.53E+06	1.61E+06	1.86E+06	2.33E+06	2.50E+06	2.22E+06	2.12E+06	3.02E+06	1.93E+06	2.87E+06	2.77E+06
Zmwp005562	C17H17NO3	cis-N-p-Coumaroyltyramine*	Alkaloids	Phenolamine	0.00E+00	0.00E+00	0.00E+00	2.63E+07	3.64E+07	4.06E+07	2.36E+07	3.35E+07	3.44E+07	1.99E+07	2.97E+07	2.97E+07
Wahp002067	C13H18N2O2	p-Coumaroylputrescine	Alkaloids	Phenolamine	1.79E+05	8.98E+04	5.22E+04	2.05E+05	2.01E+05	1.99E+05	1.68E+05	1.57E+05	1.54E+05	1.29E+05	2.78E+05	2.20E+05
pmb0069	C7H7NO	Benzamide	Alkaloids	Alkaloids	1.00E+06	9.78E+05	8.58E+05	1.15E+06	1.65E+06	1.24E+06	1.13E+06	1.37E+06	1.34E+06	1.05E+06	1.26E+06	1.27E+06
Zjhp082605	C27H42O3	Diosgenin	Steroids	Steroid	0.00E+00	0.00E+00	0.00E+00	1.15E+06	1.29E+06	1.66E+06	1.21E+06	1.55E+06	1.78E+06	1.26E+06	1.34E+06	1.53E+06
Wdtp010604	C20H30O7	3,4,5-trihydroxy-6-(4-octylphenoxy)oxane-2-carboxylic acid	Phenolic acids	Phenolic acids	2.33E+05	2.24E+05	1.97E+05	2.30E+05	1.99E+05	2.64E+05	2.53E+05	2.02E+05	2.07E+05	1.97E+05	2.09E+05	1.81E+05
pmp000579	C22H22O11	Diosmetin-7-O-galactoside*	Flavonoids	Flavones	0.00E+00	0.00E+00	0.00E+00	5.34E+05	3.37E+05	4.60E+05	4.77E+05	3.12E+05	5.33E+05	6.01E+05	4.51E+05	5.22E+05
Zasn010461	C17H34O2	Pentadecanoic acid,14-methyl-,methyl ester	Others	Others	1.44E+05	1.47E+05	1.72E+05	2.28E+05	2.09E+05	1.73E+05	2.23E+05	1.75E+05	2.71E+05	2.12E+05	1.89E+05	2.37E+05
FDATN00282	C28H36O15	Neohesperidin dihydrochalcone	Flavonoids	Chalcones	8.00E+04	9.18E+04	8.46E+04	7.12E+04	8.39E+04	8.14E+04	1.01E+05	8.18E+04	1.01E+05	9.62E+04	8.93E+04	8.54E+04
Zmdn006929	C15H12O4	Aloe-Emodin-9-Anthrone	Quinones	Anthraquinone	0.00E+00	0.00E+00	0.00E+00	3.32E+03	1.12E+05	2.63E+04	4.13E+03	1.46E+05	2.71E+04	4.17E+03	1.25E+05	2.86E+04
Lmwp102713	C14H16O5	(S)-Peucedanol	ignans and Coumarir	Coumarins	1.21E+05	9.61E+04	1.73E+05	1.46E+05	1.42E+05	1.79E+05	1.71E+05	1.38E+05	1.02E+05	1.09E+05	1.04E+05	1.41E+05
HJN090	C21H22O10	Butin-7-O-glucoside	Flavonoids	Flavanones	7.07E+03	1.13E+04	7.66E+03	2.23E+04	8.29E+04	5.53E+04	2.30E+04	1.61E+05	4.95E+04	2.57E+04	8.71E+04	6.48E+04
pmb0142	C9H8O3	Caffeic aldehyde	Phenolic acids	Phenolic acids	0.00E+00	0.00E+00	0.00E+00	4.49E+05	4.77E+05	5.04E+05	4.69E+05	3.31E+05	4.20E+05	3.84E+05	3.98E+05	3.50E+05
pmn001587	C18H24O12	Asperulosidic acid	Terpenoids	Monoterpenoids	4.28E+05	3.43E+05	3.91E+05	1.68E+05	1.87E+05	1.98E+05	1.33E+05	2.42E+05	1.68E+05	1.73E+05	2.13E+05	1.86E+05
MWSmce461	C4H7NO2	L-Azetidine-2-carboxylic acid*	Alkaloids	Alkaloids	1.29E+07	1.28E+07	1.36E+07	8.03E+06	7.40E+06	7.16E+06	6.68E+06	6.29E+06	7.21E+06	6.14E+06	5.94E+06	5.66E+06
Zjzp081862	C51H82O23	Diosgenin-Glc-Glc-Glc-Glc	Steroids	Steroidal saponins	8.81E+04	8.19E+04	6.29E+04	3.74E+05	4.88E+05	4.24E+05	3.48E+05	4.34E+05	5.72E+05	4.66E+05	4.14E+05	6.09E+05
MWSHY0031	C21H18O11	Apigenin-7-O-glucuronide*	Flavonoids	Flavones	6.07E+06	5.16E+06	4.94E+06	1.85E+07	1.32E+07	1.85E+07	2.10E+07	1.42E+07	1.66E+07	2.13E+07	1.59E+07	1.61E+07
MWSHY0190	C28H32O15	Diosmetin-7-O-rutinoside (Diosmin)*	Flavonoids	Flavones	2.47E+04	1.23E+05	3.99E+04	1.14E+05	1.67E+05	1.87E+05	2.74E+05	2.19E+05	1.74E+05	1.87E+05	2.47E+05	1.95E+05
Xmbn003152	C17H24N2O7	N-(1-deoxy-α-D fructos-1-yl)-L-tryptophan	Alkaloids	Alkaloids	1.21E+05	1.44E+05	1.16E+05	2.59E+05	2.49E+05	2.70E+05	2.25E+05	2.11E+05	2.43E+05	3.02E+05	2.45E+05	2.23E+05
Lmsn003409	C22H22O12	1-O-Galloyl-6-O-p-Coumaroyl-β-D-glucose	Phenolic acids	Phenolic acids	1.39E+04	1.67E+04	1.51E+04	1.64E+04	2.17E+04	2.12E+04	2.11E+04	2.54E+04	3.17E+04	2.71E+04	2.39E+04	2.24E+04
Lmhp002153	C13H18N2O3	N-p-Coumaroylhydroxyputrescine	Alkaloids	Phenolamine	1.42E+06	1.05E+06	8.18E+05	2.12E+06	1.81E+06	2.13E+06	1.85E+06	2.00E+06	2.15E+06	1.91E+06	1.91E+06	2.26E+06
Lahp004442	C15H12O5	3,4-Dihydro-4-(4'-hydroxyphenyl)-5,7-dihydroxycoumarin	ignans and Coumarir	Coumarins	0.00E+00	0.00E+00	0.00E+00	7.89E+04	3.72E+05	2.97E+05	7.91E+04	3.91E+05	2.52E+05	6.14E+04	4.12E+05	1.98E+05
Cmjn004337	C18H26O10	Benzyl-(2''-O-xylosyl)glucoside*	Phenolic acids	Phenolic acids	6.21E+05	5.85E+05	5.83E+05	1.00E+06	9.63E+05	9.39E+05	1.00E+06	9.90E+05	9.01E+05	1.03E+06	9.90E+05	9.00E+05
Zahp011689	C12H12O3	2,2-dimethylchromene-6-carboxylic acid	Others	Others	2.38E+07	2.19E+07	2.12E+07	1.89E+07	1.61E+07	2.07E+07	2.54E+07	1.85E+07	2.24E+07	2.47E+07	2.18E+07	2.55E+07
Lmyn000160	C8H14O8	Mucic acid Dimethyl Ester	Phenolic acids	Phenolic acids	1.97E+05	1.75E+05	1.88E+05	1.18E+05	1.00E+05	1.03E+05	1.23E+05	1.23E+05	1.15E+05	1.23E+05	1.16E+05	9.29E+04
Wdbn005328	C22H26O8	3,3'-Bis(3,4-dihydro-4-hydroxy-6,8-dimethoxy-2H-1-benzopyran)	Others	Others	1.02E+06	8.69E+05	6.37E+05	9.28E+05	1.76E+06	1.51E+06	1.39E+06	1.74E+06	1.23E+06	1.01E+06	1.94E+06	1.54E+06
mws0183	C7H6O4	3,4-Dihydroxybenzoic acid (Protocatechuic acid)*	Phenolic acids	Phenolic acids	4.62E+05	3.70E+05	3.77E+05	5.13E+05	6.57E+05	5.06E+05	6.24E+05	7.13E+05	5.74E+05	6.34E+05	7.26E+05	7.29E+05
Wafn003070	C16H20O10	Hydroxyferulic acid glucoside	Phenolic acids	Phenolic acids	7.31E+05	6.42E+05	6.45E+05	7.38E+05	8.21E+05	7.12E+05	7.14E+05	7.77E+05	6.85E+05	7.75E+05	8.51E+05	6.84E+05
Wcfp003475	C15H20N2O6	6,7-dimethyl-4-(2,3,4,5-tetrahydroxypentyl)-1H-quinoxaline-2,3-dione	Others	Others	1.85E+06	1.76E+06	1.74E+06	2.24E+06	2.49E+06	2.21E+06	2.05E+06	2.50E+06	2.17E+06	2.12E+06	2.61E+06	2.51E+06
Lalp010122	C17H18O4	7,3'-Dihydroxy-4'-methoxy-8-methylflavan	Flavonoids	Other Flavonoids	0.00E+00	0.00E+00	0.00E+00	4.13E+04	3.26E+05	1.51E+05	4.04E+04	4.51E+05	1.53E+05	2.55E+04	3.23E+05	1.15E+05
Cmbn003996	C19H26O11	Regaloside G	Phenolic acids	Phenolic acids	4.59E+05	3.84E+05	4.23E+05	3.85E+05	4.67E+05	4.13E+05	4.62E+05	4.85E+05	4.52E+05	3.72E+05	4.66E+05	4.59E+05
MWSmce331	C4H7NO2	Azetidine-2-carboxylic acid*	Alkaloids	Alkaloids	1.30E+07	1.27E+07	1.35E+07	8.04E+06	7.56E+06	7.21E+06	6.53E+06	6.14E+06	6.75E+06	6.04E+06	6.05E+06	5.91E+06
Wbsp000107	C17H24O6	(2s)-2-[(5s)-5-hydroxyhexa-1,3-dien-1-yl]-5-methoxy-4-[(3r)-3-methoxybutanoyl]-2-methylfuran-3-one	Others	Ketone compounds	2.02E+05	1.65E+05	1.81E+05	2.34E+05	2.39E+05	1.57E+05	1.16E+05	2.87E+05	1.35E+05	1.29E+05	2.28E+05	2.18E+05
Lmjp003044	C22H22O12	Isorhamnetin-3-O-Glucoside*	Flavonoids	Flavonols	0.00E+00	0.00E+00	0.00E+00	4.70E+05	8.40E+05	9.64E+05	5.52E+05	9.22E+05	9.82E+05	7.10E+05	9.85E+05	8.78E+05
Wcgn012055	C20H30O4	Araucarolone	Terpenoids	Ditepenoids	0.00E+00	0.00E+00	0.00E+00	4.99E+05	4.21E+05	4.39E+05	4.85E+05	4.65E+05	4.44E+05	5.10E+05	4.57E+05	3.85E+05
Wbkp008793	C38H48N6O7	(3S,3'S)-N,N'-((1R,2R)-4-((3,4,5-trihydroxy-6-methyltetrahydro-2H-pyran-2-yl)oxy)cyclohexane-1,2-diyl)bis(2-methyl-2,3,4,9-tetrahydro-1H-pyrido[3,4-b]indole-3-carboxamide)	Alkaloids	Alkaloids	0.00E+00	0.00E+00	0.00E+00	2.75E+05	2.64E+05	3.28E+05	3.53E+05	3.00E+05	3.66E+05	3.44E+05	3.73E+05	3.46E+05
pmp001253	C36H36N2O8	Cannabisin F	Alkaloids	Phenolamine	0.00E+00	0.00E+00	0.00E+00	1.45E+06	1.73E+06	1.59E+06	1.26E+06	1.80E+06	1.59E+06	1.25E+06	1.45E+06	1.51E+06
MWS20145	C21H22O11	Eriodictyol-7-O-glucoside	Flavonoids	Flavanones	1.96E+05	2.12E+05	2.15E+05	4.52E+05	1.92E+05	3.23E+05	3.49E+05	2.62E+05	4.22E+05	2.40E+05	3.49E+05	3.05E+05
Lmsn002288	C21H28O14	1-O-Caffeoyl-(6-O-glucosyl)-β-D-glucose	Phenolic acids	Phenolic acids	7.87E+04	6.56E+04	6.10E+04	5.37E+04	5.18E+04	8.59E+04	9.78E+04	8.63E+04	7.56E+04	5.17E+04	7.94E+04	5.71E+04
Waln006120	C19H36O10	Octyl 6-O-Alpha-L-Arabinopyranosyl-Beta-D-Glucopyranoside	Others	Others	9.98E+04	7.74E+04	7.95E+04	1.64E+05	1.33E+05	1.56E+05	1.47E+05	1.81E+05	1.55E+05	1.33E+05	1.74E+05	1.40E+05
Lamp009532	C16H14O5	4'-demethyl-3,9-dihydroeucomin	Flavonoids	Other Flavonoids	0.00E+00	0.00E+00	0.00E+00	6.72E+04	9.31E+05	3.83E+05	4.58E+04	9.95E+05	3.40E+05	6.24E+04	1.09E+06	3.73E+05
Qmxn081806	C22H32O6	Nervonin J(ISO)	Terpenoids	Ditepenoids	8.09E+03	5.92E+03	3.73E+03	1.32E+04	2.35E+04	1.71E+04	2.17E+04	1.67E+04	2.16E+04	2.67E+04	2.64E+04	1.54E+04
Lmbn005172	C9H10O3	2,6-Dimethoxybenzaldehyde	Others	Aldehyde compounds	2.54E+04	1.92E+04	2.29E+04	9.98E+04	1.39E+05	1.07E+05	1.24E+05	1.26E+05	1.06E+05	7.66E+04	1.29E+05	9.29E+04
pmb1096	C8H7N	Indole	Alkaloids	Plumerane	1.20E+06	1.62E+06	1.72E+06	1.26E+06	1.18E+06	1.24E+06	1.29E+06	1.17E+06	1.17E+06	1.21E+06	1.40E+06	1.23E+06
mws0178	C16H18O9	Chlorogenic acid (3-O-Caffeoylquinic acid)	Phenolic acids	Phenolic acids	8.51E+04	7.91E+04	7.93E+04	1.64E+05	1.98E+05	2.19E+05	1.60E+05	1.31E+05	1.95E+05	1.57E+05	1.13E+05	1.43E+05
Yajn003045	C21H32O10	Ebuloside	Terpenoids	Monoterpenoids	5.73E+04	6.96E+04	6.00E+04	9.15E+04	7.49E+04	7.29E+04	8.33E+04	8.41E+04	7.81E+04	8.28E+04	8.43E+04	6.59E+04
pmp001073	C9H13NO2	Synephrine; 4-[1-Hydroxy-2-(methylamino)ethyl]phenol	Alkaloids	Phenolamine	0.00E+00	0.00E+00	0.00E+00	1.44E+06	1.62E+06	2.09E+06	1.61E+06	1.85E+06	1.67E+06	1.93E+06	1.60E+06	2.26E+06
Wbmp002283	C9H7NO	A-hydroxyquinoline	Alkaloids	Alkaloids	4.86E+05	5.98E+05	4.06E+05	8.70E+05	1.17E+06	9.27E+05	7.58E+05	1.30E+06	7.99E+05	7.67E+05	1.08E+06	8.31E+05
mws2218	C8H10N4O2	Caffeine	Alkaloids	Alkaloids	3.25E+04	3.77E+04	1.63E+04	5.01E+04	1.10E+05	8.06E+04	6.81E+04	1.20E+05	8.54E+04	8.27E+04	1.13E+05	7.43E+04
Wagp004803	C21H30O9	Abscisic acid D-glucopyranosyl ester	Others	Others	9.56E+05	7.22E+05	8.46E+05	4.28E+06	3.88E+06	3.78E+06	3.82E+06	3.80E+06	3.99E+06	4.22E+06	3.65E+06	3.73E+06
mws0906	C16H22O8	Coniferin	Phenolic acids	Phenolic acids	8.07E+04	7.90E+04	7.55E+04	1.77E+05	1.20E+05	1.96E+05	2.03E+05	2.04E+05	1.71E+05	2.23E+05	1.79E+05	2.21E+05
Jmyn006217	C18H18O7	Polygonatone B	Others	Others	0.00E+00	0.00E+00	0.00E+00	2.20E+04	4.10E+04	9.82E+03	3.05E+03	4.57E+04	1.48E+04	2.80E+04	4.70E+04	1.03E+04
pmn001420	C15H18O9	1-O-Caffeoyl-β-D-glucose*	Phenolic acids	Phenolic acids	1.53E+05	9.29E+04	6.71E+04	9.16E+04	1.81E+05	2.21E+05	1.61E+05	1.12E+05	2.01E+05	1.91E+05	1.33E+05	1.72E+05
Zamn006847	C12H20O4	glycoric acid	Others	Others	5.67E+04	4.83E+04	4.96E+04	1.75E+05	9.56E+04	9.50E+04	1.44E+05	1.07E+05	1.21E+05	1.29E+05	8.59E+04	1.42E+05
MWS3020	C7H16N2O	N-Acetylcadaverine	Alkaloids	Alkaloids	3.21E+05	2.75E+05	3.06E+05	2.79E+05	3.56E+05	3.14E+05	3.19E+05	3.56E+05	3.16E+05	3.16E+05	4.33E+05	3.65E+05
Lmbn001981	C7H6O3	2,5-Dihydroxybenzaldehyde*	Others	Aldehyde compounds	2.37E+06	2.14E+06	1.83E+06	2.00E+06	2.52E+06	2.00E+06	2.09E+06	2.50E+06	1.93E+06	2.06E+06	2.52E+06	1.97E+06
Lmcp000282	C7H15NO3	L-Carnitine	Alkaloids	Alkaloids	2.76E+06	3.17E+06	2.64E+06	1.96E+06	2.22E+06	1.66E+06	1.97E+06	1.90E+06	1.96E+06	2.03E+06	2.24E+06	1.82E+06
Wbmn013660	C20H32O4	3,9-Dihydroxy-13(14)-labden-16,15-olide	Terpenoids	Ditepenoids	2.86E+05	3.75E+05	3.34E+05	1.76E+06	2.04E+06	1.57E+06	1.80E+06	1.84E+06	1.83E+06	1.69E+06	2.09E+06	1.59E+06
pmn001727	C24H29NO9	N-(4-O-(Glucosyl)-E-feruloyl)-tyramine	Alkaloids	Phenolamine	2.45E+05	4.16E+05	2.71E+05	9.99E+05	1.62E+06	1.31E+06	1.03E+06	1.79E+06	1.51E+06	1.01E+06	1.49E+06	1.40E+06
mws1397	C22H18O10	Epicatechin gallate*	Flavonoids	Flavanols	4.09E+04	4.23E+04	5.33E+04	4.01E+04	4.18E+04	4.20E+04	4.81E+04	2.91E+04	3.62E+04	4.38E+04	3.36E+04	2.70E+04
MWSHC2025	C12H12N2O2	(R)-1,2,3,4-Tetrahydro-3-carboxy-2-carboline	Alkaloids	Alkaloids	2.82E+06	2.73E+06	2.67E+06	5.37E+06	5.55E+06	5.18E+06	5.35E+06	6.47E+06	5.83E+06	6.40E+06	7.55E+06	6.57E+06
Hmgp006701	C36H36O8N2	Lyciumamide A	Alkaloids	Phenolamine	0.00E+00	0.00E+00	0.00E+00	1.75E+06	2.13E+06	1.96E+06	1.65E+06	2.08E+06	1.93E+06	1.60E+06	1.95E+06	1.62E+06
Yaap003029	C11H14O5	3,4,5-trimethoxyphenyl acetate	Others	Others	1.26E+06	1.24E+06	1.18E+06	7.13E+05	8.30E+05	8.22E+05	6.36E+05	8.43E+05	8.04E+05	7.27E+05	8.57E+05	7.39E+05
Lmmn006246	C20H34O4	7β-Hydroxydarutigenol	Terpenoids	Ditepenoids	2.24E+06	1.14E+06	2.22E+06	2.24E+06	3.35E+06	1.84E+06	2.49E+06	1.98E+06	2.11E+06	2.28E+06	2.62E+06	2.87E+06
pmf0070	C29H50O	Stigmast-5-en-3-ol (β-Sitosterol)	Steroids	Steroid	1.20E+06	9.84E+05	9.40E+05	5.67E+06	9.04E+06	7.56E+06	8.84E+06	7.24E+06	9.25E+06	1.06E+07	5.52E+06	1.27E+07
Hmjp005089	C18H19NO5	(2Z)-N-[2-(3,4-Dihydroxyphenyl)-2-hydroxyethyl]-3-(4-methoxyphenyl)-2-propenamide*	Alkaloids	Phenolamine	1.53E+03	6.16E+03	8.00E+03	8.03E+04	7.95E+04	1.15E+05	9.00E+04	9.96E+04	6.40E+04	1.58E+05	1.31E+05	7.44E+04
Wbmp006300	C25H33NO4	1,2,9,10-tetraethoxy-6-methyl-5,6,6a,7-tetrahydro-4H-dibenzo[de,g]quinoline	Alkaloids	Alkaloids	2.93E+06	3.01E+06	2.87E+06	7.08E+06	8.10E+06	6.43E+06	6.38E+06	8.83E+06	7.63E+06	6.54E+06	9.47E+06	7.70E+06
Zahp011321	C14H29NO	N-Isobutyl Decanamide	Alkaloids	Alkaloids	1.41E+05	1.17E+05	6.39E+04	3.06E+05	3.26E+05	3.15E+05	2.70E+05	3.45E+05	3.32E+05	3.71E+05	3.67E+05	4.00E+05
Lmyn003028	C19H28O11	Benzyl-β-gentiobioside*	Phenolic acids	Phenolic acids	2.87E+05	2.71E+05	2.18E+05	1.46E+05	1.44E+05	9.57E+04	1.58E+05	1.39E+05	1.40E+05	1.34E+05	1.54E+05	9.15E+04
MWSmce446	C22H28O11	Cimifugin-7-O-glucoside	Others	Chromone	4.93E+05	4.30E+05	3.25E+05	2.39E+05	4.26E+05	3.56E+05	6.31E+05	4.71E+05	3.15E+05	2.95E+05	4.47E+05	2.63E+05
Zjhp082613	C33H52O8	Diosgenin-Glc	Steroids	Steroidal saponins	4.39E+06	3.85E+06	3.53E+06	1.24E+07	1.35E+07	1.30E+07	1.34E+07	1.38E+07	1.75E+07	1.25E+07	1.42E+07	1.37E+07
Lmtn002565	C14H18O9	1-O-Vanilloyl-D-Glucose	Phenolic acids	Phenolic acids	4.63E+05	5.37E+05	4.33E+05	8.70E+05	1.21E+06	1.01E+06	8.49E+05	1.44E+06	1.10E+06	9.28E+05	1.65E+06	1.06E+06
Hmbp003234	C12H14O6	1-O-Caffeoylglycerol	Phenolic acids	Phenolic acids	1.97E+05	1.52E+05	1.78E+05	2.40E+05	1.88E+05	1.82E+05	1.63E+05	1.58E+05	2.09E+05	2.10E+05	1.54E+05	1.62E+05
Wbtn002952	C13H19NO7	1-(β-D-Glucopyranosyl)-3-ethyl-4-methyl-1H-pyrrole-2,5-dione	Others	Ketone compounds	1.55E+05	1.79E+05	1.77E+05	1.68E+05	1.76E+05	1.43E+05	1.60E+05	1.65E+05	1.38E+05	1.40E+05	1.65E+05	1.44E+05
pmn001381	C15H26O9	Eucommioside	Terpenoids	Terpene	1.17E+05	5.66E+04	2.17E+04	3.47E+05	3.70E+05	4.32E+05	2.76E+05	2.50E+05	2.36E+05	1.96E+05	3.01E+05	2.09E+05
Zmhn002301	C15H18O8	p-Coumaric acid-4-O-glucoside	Phenolic acids	Phenolic acids	2.76E+05	2.86E+05	1.94E+05	1.92E+05	2.55E+05	2.01E+05	2.12E+05	1.79E+05	2.83E+05	1.83E+05	2.13E+05	1.64E+05
MWSHC20105	C20H20O7	4-ketopinoresinol	ignans and Coumarir	Lignans	7.95E+04	6.66E+04	6.81E+04	2.49E+05	2.47E+05	2.60E+05	2.01E+05	2.41E+05	2.46E+05	2.74E+05	2.56E+05	2.55E+05
Lmxp011770	C16H22O4	Diisobutyl phthalate*	Phenolic acids	Phenolic acids	7.80E+06	7.14E+06	6.59E+06	6.33E+06	5.93E+06	5.80E+06	6.50E+06	5.08E+06	4.56E+06	6.12E+06	8.40E+06	6.75E+06
Zjhp082622	C38H58O13	Gentrogenin-Glc-Xyl	Steroids	Steroidal saponins	1.05E+05	1.24E+05	9.89E+04	3.12E+05	2.82E+05	3.12E+05	2.64E+05	2.53E+05	2.93E+05	4.33E+05	4.41E+05	3.21E+05
Hmlp008487	C13H22O2	Blumenol C	Terpenoids	Monoterpenoids	1.41E+05	1.18E+05	1.59E+05	1.88E+05	1.58E+05	1.54E+05	1.48E+05	9.75E+04	1.30E+05	1.22E+05	1.50E+05	1.21E+05
Lmcn004052	C33H40O13	Buddlenol D	Phenolic acids	Phenolic acids	1.82E+04	1.28E+04	1.74E+04	6.14E+04	4.78E+04	7.28E+04	4.85E+04	6.90E+04	4.78E+04	5.48E+04	5.03E+04	5.26E+04
Lacn005311	C26H30O12	Phellatin	Flavonoids	Flavonols	3.54E+05	2.43E+05	2.56E+05	3.34E+05	5.24E+05	3.55E+05	3.57E+05	3.95E+05	3.98E+05	3.51E+05	4.93E+05	4.28E+05
Lamp009984	C16H12O5	4'-Demethyleucomin	Flavonoids	Other Flavonoids	0.00E+00	0.00E+00	0.00E+00	2.95E+04	1.73E+05	7.85E+04	1.93E+04	2.11E+05	9.98E+04	1.67E+04	2.40E+05	5.41E+04
Qabp005310	C45H72O18	Dehydroxy Epiruscogenin-glucose-glucose-glucoside	Steroids	Steroidal saponins	1.52E+04	9.43E+04	8.53E+04	3.22E+05	2.74E+05	4.78E+05	3.28E+05	4.27E+05	1.87E+05	2.29E+05	2.53E+05	3.04E+05
Sazp011041	C20H22O5	Phyllnirurin	ignans and Coumarir	Lignans	9.22E+05	9.92E+05	9.35E+05	9.85E+05	1.18E+06	8.90E+05	1.06E+06	1.14E+06	9.75E+05	9.30E+05	1.50E+06	1.21E+06
Sadp007533	C17H16O3	5-formyl-2,6-dihydroxy-1,7-dimethyl-9,10-dihydrophenanthrene	Terpenoids	Ditepenoids	2.03E+04	2.13E+04	3.84E+04	2.61E+05	2.89E+05	2.51E+05	2.39E+05	2.66E+05	1.97E+05	2.89E+05	2.94E+05	2.64E+05
Hmlp013407	C15H26O	β-Eudesmol	Terpenoids	Sesquiterpenoids	3.12E+04	2.88E+04	2.85E+04	2.42E+04	3.68E+04	2.58E+04	2.47E+04	3.07E+04	2.45E+04	2.58E+04	3.11E+04	2.70E+04
Zmhn002334	C16H20O9	6-O-Feruloyl-β-D-glucose	Phenolic acids	Phenolic acids	2.36E+05	1.94E+05	1.85E+05	2.70E+05	2.36E+05	2.53E+05	3.16E+05	2.04E+05	2.64E+05	2.41E+05	2.65E+05	2.72E+05
Sadp008019	C17H16O3	5-formyl-2,7-dihydroxy-1,6-dimethyl-9,10-	Terpenoids	Ditepenoids	0.00E+00	0.00E+00	0.00E+00	3.02E+05	4.04E+05	4.01E+05	3.06E+05	4.89E+05	3.95E+05	3.08E+05	4.17E+05	3.21E+05

		dihydrophenanthrene														
Smln004290	C17H12O6	Aloesaponarin I	Quinones	Anthraquinone	6.84E+03	5.50E+03	7.01E+03	6.71E+04	6.44E+04	7.51E+04	7.64E+04	8.35E+04	8.11E+04	8.03E+04	1.04E+05	7.54E+04
Wbjp006632	C36H36N2O8	Tribulusamide A	Alkaloids	Alkaloids	0.00E+00	0.00E+00	0.00E+00	1.94E+06	2.32E+06	2.39E+06	1.79E+06	2.30E+06	2.24E+06	1.54E+06	2.21E+06	2.17E+06
Wcdp010708	C18H30O	Octadeca-2,9,12,15-tetraen-1-ol	Others	Others	0.00E+00	0.00E+00	0.00E+00	2.53E+06	2.18E+06	2.22E+06	2.52E+06	2.58E+06	2.11E+06	2.36E+06	2.41E+06	2.30E+06
Wbmp006077	C26H36NO4+	1,10-dimethoxy-6,6-dimethyl-2,9-di(propan-2-yloxy)-5,6,6a,7-tetrahydro-4H-dibenzo[de,g]quinolin-6-ium	Alkaloids	Alkaloids	5.63E+06	4.74E+06	4.53E+06	1.51E+07	1.56E+07	1.48E+07	1.58E+07	1.57E+07	1.42E+07	1.58E+07	1.52E+07	1.47E+07
Wcfn003299	C17H20O6	(2S)-3,3-bis-(4-hydroxy-3-methoxyphenyl)-propane-1,2-diol	Others	Others	6.53E+04	9.65E+04	9.07E+04	1.09E+05	9.16E+04	9.49E+04	1.06E+05	1.02E+05	1.05E+05	1.05E+05	1.04E+05	9.76E+04
Lmjp004581	C36H54O7	Integracide H	Terpenoids	Triterpene	2.61E+05	3.46E+05	3.31E+05	2.09E+06	1.95E+06	1.55E+06	1.75E+06	2.10E+06	1.51E+06	1.66E+06	1.73E+06	2.04E+06
Wchn006110	C20H24O6	Tetrahydro-2-(4-Hydroxy-3-Methoxyphenyl)-6-(Alpha,4-Dihydroxyphenethyl)-4H-Pyran-4-Ol	Others	Others	3.23E+04	2.70E+04	2.34E+04	7.17E+04	5.38E+04	4.59E+04	5.43E+04	4.49E+04	5.64E+04	7.49E+04	4.10E+04	6.57E+04
pma0149	C15H16O9	Sinapoyl malate	Phenolic acids	Phenolic acids	1.50E+05	1.40E+05	1.18E+05	3.19E+05	2.43E+05	2.94E+05	2.33E+05	2.38E+05	3.62E+05	3.02E+05	2.42E+05	3.28E+05
Wcdp010864	C16H33NO	Hexadecanamide	Alkaloids	Alkaloids	3.10E+05	5.24E+05	2.84E+05	1.24E+06	1.23E+06	1.05E+06	1.32E+06	1.46E+06	1.52E+06	1.12E+06	1.21E+06	1.69E+06
Zjbp081720	C45H72O18	Spirost-5-ene-3,27-diol-3-O-rhamnosyl(1→2)O-[glucosyl(l→4)]glucoside	Steroids	Steroidal saponins	1.19E+05	9.62E+04	8.98E+04	5.84E+05	2.74E+05	4.26E+05	5.81E+05	5.72E+05	3.57E+05	4.97E+05	3.78E+05	5.15E+05
Lskp211267	C10H10O3	3-Ethyl-7-hydroxyphthalide	Others	Others	4.07E+05	4.13E+05	3.90E+05	3.99E+05	4.32E+05	4.00E+05	4.98E+05	4.52E+05	5.33E+05	4.09E+05	5.03E+05	5.96E+05
Lakn004098	C15H22O5	Dihydrophaseic acid	Terpenoids	Sesquiterpenoids	1.67E+04	2.23E+04	2.36E+04	3.48E+04	3.09E+04	2.66E+04	2.63E+04	3.08E+04	4.46E+04	4.45E+04	2.66E+04	3.14E+04
Wcdp003967	C12H27NO2	Deacetylclavaminol H	Others	Others	6.96E+06	6.68E+06	7.99E+06	8.49E+06	9.63E+06	9.06E+06	7.86E+06	7.56E+06	7.95E+06	8.22E+06	8.36E+06	9.20E+06
Lmwp003442	C21H20O11	Amoenin	Flavonoids	Flavonols	1.61E+05	2.29E+05	2.09E+05	3.99E+05	3.69E+05	2.65E+05	3.49E+05	4.69E+05	2.53E+05	4.01E+05	3.72E+05	3.43E+05
Zjhp090636	C16H12O5	5,7-dihydroxy-3-(4'-hydroxybenzylidene)-chroman-4-one	Flavonoids	Other Flavonoids	0.00E+00	0.00E+00	0.00E+00	1.93E+04	1.70E+05	8.84E+04	2.79E+04	2.23E+05	9.72E+04	1.67E+04	2.30E+05	5.76E+04
Zjmp102002	C18H18O5	ophiopogonanone B*	Flavonoids	Other Flavonoids	0.00E+00	0.00E+00	0.00E+00	2.78E+05	2.15E+06	9.31E+05	2.47E+05	2.50E+06	1.06E+06	2.74E+05	2.39E+06	9.07E+05
Zmtn001624	C10H7NO3	N-Acetylisatin	Alkaloids	Plumerane	1.87E+05	1.32E+05	2.16E+05	1.94E+05	2.76E+05	2.57E+05	2.38E+05	2.73E+05	3.07E+05	2.26E+05	3.33E+05	2.63E+05
MWSmce341	C16H22O4	Butyl isobutyl phthalate*	Phenolic acids	Phenolic acids	3.00E+07	3.29E+07	2.90E+07	3.15E+07	3.08E+07	2.28E+07	2.10E+07	1.68E+07	1.48E+07	3.03E+07	2.41E+07	3.29E+07
Qmgp102132	C27H45NO3	Vertaline B	Alkaloids	Steroid alkaloids	6.16E+04	4.53E+04	4.48E+04	1.93E+05	4.72E+05	3.62E+05	4.16E+05	3.44E+05	3.88E+05	3.52E+05	2.27E+05	3.77E+05
pme2768	C14H6N2O8	Pyrroloquinoline quinone	Alkaloids	Quinoline alkaloids	3.04E+05	2.71E+05	2.55E+05	4.04E+05	4.16E+05	4.44E+05	4.00E+05	4.23E+05	4.40E+05	4.06E+05	3.75E+05	4.39E+05
pmp001187	C36H36N2O8	1-(Dihydroxyphenyl)-N2,N3-bis(4-hydroxyphenethyl)-(5-8)-dimethoxy-1,2dihydronaphthalene-2.3-dicarboxamide	Alkaloids	Phenolamine	0.00E+00	0.00E+00	0.00E+00	2.25E+06	2.73E+06	2.95E+06	2.43E+06	2.90E+06	3.20E+06	2.65E+06	2.84E+06	2.96E+06
Zjzp081818	C39H62O14	iso-2-Hydroxy diosgenin-Glc-Glc	Steroids	Steroidal saponins	6.04E+05	7.90E+05	9.48E+05	2.07E+06	3.27E+06	1.98E+06	3.20E+06	2.56E+06	1.43E+06	1.93E+06	3.43E+06	2.38E+06
pmp001269	C18H39NO	Hexadecyl ethanolamine	Alkaloids	Alkaloids	0.00E+00	0.00E+00	0.00E+00	3.79E+05	7.35E+05	4.21E+05	6.74E+05	5.38E+05	4.50E+05	3.34E+05	5.39E+05	5.18E+05
Zblp005777	C18H19NO4	N-trans-ferulicacidacylp-hydroxyphenylethylamine*	Alkaloids	Phenolamine	1.66E+05	1.34E+05	1.69E+05	3.77E+07	5.65E+07	5.21E+07	4.05E+07	5.43E+07	5.21E+07	3.86E+07	5.30E+07	4.50E+07
Zmsn003194	C16H22O10	Secologaoside	Others	Others	9.32E+04	6.54E+04	6.81E+04	2.58E+05	1.94E+05	1.80E+05	2.41E+05	1.39E+05	1.33E+05	2.61E+05	1.52E+05	2.08E+05
mws0856	C21H20O12	Quercetin-4'-O-glucoside (Spiraeoside)*	Flavonoids	Flavonols	1.75E+04	2.45E+04	1.12E+04	3.95E+04	1.66E+05	1.01E+05	2.22E+04	1.39E+05	1.07E+05	4.38E+04	2.24E+05	1.72E+05
Lszp210010	C15H20N2O6	Tatarine	Alkaloids	Alkaloids	2.46E+05	2.27E+05	2.16E+05	1.71E+05	1.89E+05	1.57E+05	1.67E+05	1.72E+05	1.88E+05	2.38E+05	2.13E+05	1.76E+05
WaYn006596	C32H38O12	Buddlenol C*	ignans and Coumarir	Lignans	1.24E+04	8.18E+03	8.17E+03	3.57E+04	3.13E+04	3.10E+04	2.92E+04	2.76E+04	3.16E+04	3.94E+04	3.39E+04	3.14E+04
Hmln008119	C11H18O3	Robinlin	Terpenoids	Terpene	2.64E+04	2.25E+04	2.08E+04	3.75E+04	3.83E+04	2.83E+04	3.39E+04	4.02E+04	4.07E+04	3.59E+04	3.57E+04	3.03E+04
Hmmn002544	C16H20O9	Ferulic acid-4-O-glucoside	Phenolic acids	Phenolic acids	6.29E+04	4.27E+04	3.28E+04	1.17E+05	7.18E+04	6.31E+04	9.07E+04	4.76E+04	7.61E+04	6.22E+04	7.11E+04	6.99E+04
mws0355	C22H18O10	Catechin gallate*	Flavonoids	Flavanols	2.80E+04	2.96E+04	3.22E+04	3.04E+04	3.99E+04	2.33E+04	2.39E+04	2.76E+04	2.77E+04	2.86E+04	1.65E+04	2.02E+04
Zjhp090620	C19H20O6	5,7-dihydroxy-6,8-dimethyl-3(R,S)-(3'-hydroxy-4'-methoxybenzyl)-chroman-4-one*	Flavonoids	Other Flavonoids	0.00E+00	0.00E+00	0.00E+00	4.27E+04	3.63E+05	6.85E+04	2.90E+04	4.54E+05	1.00E+05	3.03E+04	4.16E+05	7.55E+04
Lmsp102219	C15H10O5	Aloe emodin	Quinones	Anthraquinone	5.63E+04	5.05E+04	6.14E+04	8.02E+04	1.86E+05	1.06E+05	8.67E+04	1.27E+05	9.87E+04	9.59E+04	1.25E+05	7.04E+04
Lskp211278	C10H16N2O5	Oxypinnatanine A	Alkaloids	Alkaloids	6.45E+05	6.39E+05	6.42E+05	2.75E+05	6.61E+05	4.14E+05	4.06E+05	4.80E+05	5.74E+05	2.89E+05	6.32E+05	4.01E+05
pme3083	C8H7NO3	2-(Formylamino)benzoic acid	Phenolic acids	Phenolic acids	1.22E+05	1.10E+05	1.20E+05	3.18E+05	3.05E+05	2.88E+05	3.04E+05	2.56E+05	3.04E+05	2.50E+05	2.35E+05	2.21E+05
Wbsp000037	C14H21NO2	1-[3-(4-methyl-6-oxocyclohex-1-en-1-yl)propyl]pyrrolidin-2-one	Others	Ketone compounds	2.95E+06	2.29E+06	2.61E+06	2.81E+06	2.54E+06	2.82E+06	2.45E+06	2.12E+06	2.93E+06	2.71E+06	2.50E+06	2.61E+06
Hmyp002656	C11H11NO4	Methyl dioxindole-3-acetate	Alkaloids	Plumerane	2.52E+05	2.05E+05	2.52E+05	4.03E+05	3.44E+05	3.31E+05	3.28E+05	3.34E+05	3.32E+05	2.88E+05	3.32E+05	2.79E+05
Saln000462	C25H30O12	7-O-coumaroyl-loganic acid	Terpenoids	Monoterpenoids	8.25E+05	9.05E+05	7.25E+05	2.10E+06	1.60E+06	1.74E+06	1.75E+06	1.64E+06	1.43E+06	1.74E+06	1.81E+06	1.89E+06
Yajn003638	C16H26O10	Lamiol	Terpenoids	Monoterpenoids	1.05E+05	6.53E+04	5.28E+04	1.03E+05	9.33E+04	1.17E+05	1.22E+05	1.19E+05	1.09E+05	9.08E+04	1.32E+05	1.25E+05
MWSmce352	C27H43NO3	Sipeimine	Alkaloids	Steroid alkaloids	2.70E+06	2.34E+06	2.88E+06	7.24E+06	6.31E+06	5.94E+06	5.92E+06	5.70E+06	7.27E+06	6.80E+06	5.21E+06	5.86E+06
MWS1839	C9H10O3	Ethylparaben	Phenolic acids	Phenolic acids	1.37E+04	1.36E+04	1.46E+04	1.08E+05	6.84E+04	8.40E+04	1.14E+05	7.96E+04	1.10E+05	1.91E+05	2.04E+05	2.04E+05
Jmwn004371	C31H40O16	4-Hydroxyphenethoxy-8-O-β-D-[6-O-(4-O-β-D-glucopyranosyl)-sinapoyl]glucopyranoside	Phenolic acids	Phenolic acids	1.68E+04	4.72E+04	3.23E+04	6.17E+04	1.27E+05	7.17E+04	5.22E+04	2.11E+05	1.24E+05	4.58E+04	1.73E+05	1.09E+05
Yaan003788	C28H40O14	[(2R,3R,4S,5S)-4-hydroxy-5-(hydroxymethyl)-2-[[(2R,3S,4S,5R,6S)-3,4,5-trihydroxy-6-[2-[(1R)-4-methylcyclohex-3-en-1-yl]propan-2-yloxy]oxan-2-yl]methoxy]oxolan-3-yl]3,4,5-trihydroxybenzoate	Others	Others	6.72E+04	4.99E+04	8.39E+04	1.70E+05	1.13E+05	2.27E+05	1.37E+05	1.28E+05	1.93E+05	1.34E+05	1.13E+05	2.81E+05
WaYn006121	C23H26O10	Salicin 7-O-Ferulate	Phenolic acids	Phenolic acids	0.00E+00	0.00E+00	0.00E+00	5.30E+03	6.61E+04	5.80E+04	3.49E+03	5.18E+04	4.69E+04	4.63E+03	6.64E+04	4.74E+04
Zjhp090606	C17H16O5	(3R)-5,7-dihydroxyl-6-methyl-3-(4'-hydroxylbenzyl)-chroman-4-one*	Flavonoids	Other Flavonoids	0.00E+00	0.00E+00	0.00E+00	4.31E+05	6.28E+06	2.83E+06	4.34E+05	7.42E+06	3.17E+06	4.48E+05	7.60E+06	2.54E+06
Zjmp102025	C17H14O5	Desmethyl-ophiopogonone B*	Flavonoids	Other Flavonoids	0.00E+00	0.00E+00	0.00E+00	1.17E+05	1.82E+05	8.94E+04	1.53E+05	2.05E+05	1.01E+05	1.39E+05	1.78E+05	7.84E+04
Lmmn001552	C14H20O9	Leonuriside A	Phenolic acids	Phenolic acids	9.35E+04	7.84E+04	7.48E+04	1.22E+05	1.60E+05	1.40E+05	1.67E+05	1.10E+05	1.26E+05	1.03E+05	1.44E+05	1.79E+05
Wmgp004939	C19H22O2N2	N-Desmethoxyrankinidine	Alkaloids	Plumerane	1.26E+05	7.88E+04	9.95E+04	5.48E+05	3.42E+05	2.19E+05	2.69E+05	3.81E+05	1.63E+05	1.83E+05	2.52E+05	4.14E+05
Wbjp010933	C27H38O4	5',6a,8a,9-tetramethyl-1,3',4',5',6,6a,6b,6',7,8a,8b,9,11a,12,12a,12b-hexadecahydrospiro[naphtho[2',1':4,5]indeno[2,1-b]furan-10,2'-pyran]-4,8(2H,5H)-dione	Alkaloids	Alkaloids	7.74E+03	6.83E+03	7.18E+03	1.06E+05	7.00E+04	5.59E+04	5.33E+04	1.04E+05	8.24E+04	8.34E+04	1.07E+05	8.39E+04
Hmln003729	C18H34O11	Capsoside B	Others	Others	8.19E+04	6.99E+04	1.21E+05	1.38E+05	1.40E+05	1.04E+05	1.31E+05	1.23E+05	1.50E+05	1.40E+05	7.60E+04	1.39E+05
MWS20194	C9H8O2	Cinnamic acid	Phenolic acids	Phenolic acids	4.01E+05	3.86E+05	1.58E+05	5.17E+05	5.36E+05	2.70E+05	3.71E+05	3.23E+05	3.78E+05	3.91E+05	3.11E+05	4.26E+05
Zmhp002730	C27H30O17	6-Hydroxykaempferol-6,7-O-Diglucoside*	Flavonoids	Flavonols	3.47E+04	6.27E+04	4.47E+04	1.25E+05	7.28E+04	8.55E+04	8.92E+04	9.15E+04	1.43E+05	1.04E+05	8.95E+04	9.10E+04
Hasp010605	C21H36O4	(S)-2,3-dihydroxypropyl (9Z,12Z,15Z)-octadeca-9,12,15-trienoate	Others	Others	0.00E+00	0.00E+00	0.00E+00	2.86E+06	2.81E+06	2.91E+06	2.75E+06	2.72E+06	2.37E+06	2.27E+06	2.32E+06	2.49E+06
MWSslk108	C9H17NO4	O-Acetyl-L-carnitine	Alkaloids	Alkaloids	9.29E+05	1.07E+06	7.73E+05	6.10E+05	7.19E+05	7.64E+05	7.30E+05	5.27E+05	5.37E+05	7.23E+05	7.35E+05	7.32E+05
Lmyp008454	C15H10O3	2-(Hydroxymethyl)Anthraquinone	Quinones	Anthraquinone	0.00E+00	0.00E+00	0.00E+00	6.00E+04	6.44E+04	8.49E+04	7.59E+04	7.84E+04	9.92E+04	1.48E+05	6.38E+04	5.32E+04
Wafn004433	C17H32O10	1-Hexanol arabinosylglucoside	Others	Others	1.06E+04	1.61E+04	2.75E+04	2.57E+04	2.91E+04	3.62E+04	2.01E+04	3.12E+04	1.67E+04	2.01E+04	1.48E+04	2.98E+04
MWSmce064	C16H14O6	Hematoxylin	Phenolic acids	Phenolic acids	9.51E+04	7.29E+04	8.41E+04	1.79E+05	1.43E+05	1.46E+05	1.17E+05	1.68E+05	1.73E+05	1.61E+05	1.93E+05	1.69E+05
mws0789	C15H12O4	Pinocembrin (Dihydrochrysin)	Flavonoids	Flavanones	0.00E+00	0.00E+00	0.00E+00	5.59E+03	8.41E+04	2.28E+04	2.82E+04	1.16E+05	1.83E+04	2.86E+04	1.15E+05	2.36E+04
pmb0819	C10H8N2	3-Indoleacetonitrile	Alkaloids	Plumerane	9.96E+04	1.27E+05	1.38E+05	1.48E+05	2.18E+05	1.56E+05	1.38E+05	1.85E+05	1.50E+05	1.18E+05	1.26E+05	1.26E+05
Lazn004429	C28H38O13	(2S,3R,4S,6S)-2-[4-[(1R,2R)-1,3-Dihydroxy-2-[4-[(E)-3-hydroxyprop-1-enyl]-2,6-dimethoxyphenoxy]propyl]-2,6-dimethoxyphenoxy]-6-(hydroxymethyl)oxane-3,4-diol	ignans and Coumarir	Lignans	1.56E+05	1.11E+05	1.43E+05	1.90E+05	1.86E+05	1.97E+05	1.76E+05	1.83E+05	1.66E+05	2.28E+05	2.78E+05	1.80E+05
MWSmce177	C12H14O4	Ethyl ferulate	Phenolic acids	Phenolic acids	9.19E+03	8.80E+03	1.65E+04	4.25E+04	4.48E+04	4.25E+04	4.05E+04	3.06E+04	4.18E+04	3.13E+04	3.84E+04	3.77E+04
MWS20152	C22H26O8	Syringaresinol	ignans and Coumarir	Lignans	4.34E+04	1.50E+04	3.77E+04	7.32E+04	9.55E+04	1.02E+05	7.48E+04	1.00E+05	7.19E+04	9.36E+04	8.41E+04	7.33E+04
Lacp001298	C9H13NO2	4-Hydroxy-3-methoxy-β-phenethylamine	Alkaloids	Phenolamine	1.00E+05	1.07E+05	1.46E+05	7.66E+04	8.88E+04	8.91E+04	7.11E+04	7.22E+04	1.19E+05	6.48E+04	1.11E+05	9.47E+04
Lmqn006260	C15H10O7	2',3',4',5,7-Pentahydroxyflavone*	Flavonoids	Flavones	0.00E+00	0.00E+00	0.00E+00	6.00E+04	1.78E+05	4.94E+04	6.00E+04	1.83E+05	5.84E+04	6.51E+04	1.78E+05	5.42E+04
Smcp000882	C15H21NO7	N-benzoyl-2-aminoethyl-β-D-glucopyranoside	Alkaloids	Alkaloids	9.74E+06	9.19E+06	8.73E+06	1.81E+07	1.88E+07	1.87E+07	1.84E+07	1.80E+07	1.86E+07	1.83E+07	1.66E+07	1.87E+07
mws1708	C13H18O7	Gastrodin	Phenolic acids	Phenolic acids	1.69E+05	1.89E+05	1.24E+05	2.55E+05	2.62E+05	3.14E+05	3.40E+05	2.32E+05	3.68E+05	3.05E+05	2.54E+05	2.37E+05
pmn001497	C33H44N2O3	Dendrocrepine*	Alkaloids	Alkaloids	0.00E+00	0.00E+00	0.00E+00	4.40E+05	7.19E+05	2.92E+05	5.24E+05	5.68E+05	5.76E+05	4.26E+05	9.96E+05	6.52E+05
Zmcp002666	C27H30O17	Quercetin-3,7-Di-O-glucoside*	Flavonoids	Flavonols	3.79E+05	4.75E+05	2.95E+05	1.26E+06	1.32E+06	1.28E+06	1.29E+06	1.17E+06	1.35E+06	1.24E+06	1.21E+06	1.42E+06
Wcfn003941	C12H18O7S	Sulfo jasmonate	Others	Others	8.93E+04	9.70E+04	9.56E+04	2.37E+05	2.03E+05	3.22E+05	1.69E+05	2.23E+05	2.79E+05	1.79E+05	3.19E+05	2.80E+05
MWSprf097	C18H25NO6	USaramine	Alkaloids	Alkaloids	1.04E+05	1.06E+05	1.18E+05	3.23E+05	2.16E+05	2.78E+05	2.55E+05	2.66E+05	2.22E+05	2.12E+05	2.91E+05	2.72E+05
MWS2058	C8H8O3	3-Methylsalicylic Acid	Phenolic acids	Phenolic acids	4.91E+04	3.53E+04	3.90E+04	1.00E+05	1.26E+05	1.45E+05	1.76E+05	1.74E+05	2.15E+05	2.69E+05	2.07E+05	2.54E+05
Hmdp003288	C51H82O23	Trillin-6'-O-sophorotrioside	Steroids	Steroidal saponins	1.18E+05	8.62E+04	1.18E+05	4.70E+05	2.91E+05	4.70E+05	2.69E+05	4.87E+05	6.10E+05	4.85E+05	6.53E+05	3.76E+05
mws2212	C9H8O4	Caffeic acid	Phenolic acids	Phenolic acids	1.92E+05	1.34E+05	2.32E+05	1.29E+05	2.80E+05	2.76E+05	1.89E+05	2.75E+05	2.49E+05	1.87E+05	2.43E+05	2.95E+05
Wcsn011332	C18H32O2	Ethyl (10Z,13Z)-hexadeca-10,13-dienoate	Others	Others	0.00E+00	0.00E+00	0.00E+00	1.52E+06	1.46E+06	1.41E+06	1.79E+06	1.90E+06	1.82E+06	1.74E+06	1.85E+06	1.71E+06
Zjzp081805	C33H54O8	Sarsasapogenin-3-O-galactoside (Timosaponin A-I)	Steroids	Steroidal saponins	1.36E+05	1.19E+05	1.11E+05	9.50E+05	8.28E+05	9.81E+05	9.51E+05	1.21E+06	1.23E+06	6.66E+05	8.28E+05	1.18E+06
Ladn005852	C20H20O7	Graminone A	ignans and Coumarir	Lignans	5.01E+04	5.98E+04	5.22E+04	2.57E+05	2.50E+05	2.83E+05	2.62E+05	2.23E+05	2.82E+05	2.95E+05	2.35E+05	2.32E+05
mws1383	C12H10N4O2	Lumichrome	Alkaloids	Alkaloids	1.89E+05	1.64E+05	1.56E+05	2.41E+05	2.94E+05	2.97E+05	2.27E+05	3.46E+05	3.16E+05	2.24E+05	3.11E+05	2.50E+05
Zjhp082607	C27H40O4	3-Hydroxyspirost-5-en-12-one (Gentrogenin)	Steroids	Steroid	7.09E+05	5.44E+05	7.45E+05	3.28E+06	3.85E+06	4.20E+06	3.98E+06	3.79E+06	4.90E+06	3.94E+06	3.92E+06	4.64E+06
Wbkp008219	C38H46N6O7	(3S,3'S)-N,N'-((1R,2R)-5-((3,4,5-trihydroxy-6-methyltetrahydro-2H-pyran-2-yl)oxy)cyclohex-3-ene-1,2-diyl)bis(2-methyl-2,3,4,9-tetrahydro-1H-pyrido[3,4-b]indole-3-carboxamide)	Alkaloids	Alkaloids	4.15E+05	4.34E+05	4.58E+05	9.77E+06	9.45E+06	9.25E+06	8.26E+06	9.27E+06	9.81E+06	8.72E+06	9.80E+06	9.58E+06
Lazn005469	C38H48O17	Buddlenol F glucoside	ignans and Coumarir	Lignans	5.90E+04	8.86E+04	8.47E+04	1.29E+05	1.21E+05	1.36E+05	1.30E+05	1.09E+05	1.27E+05	1.26E+05	1.08E+05	1.48E+05
Lmjp005170	C15H14O5	4,9-Dihydroxy-α-Lapachone	Quinones	Quinones	2.49E+05	3.43E+05	2.56E+05	3.74E+05	3.02E+05	2.59E+05	5.57E+05	3.46E+05	2.78E+05	3.28E+05	2.68E+05	2.83E+05
pme3227	C27H30O14	Vitexin-2''-O-rhamnoside	Flavonoids	Flavones	6.18E+04	2.42E+04	6.59E+04	1.90E+05	2.19E+05	2.63E+05	2.58E+05	1.67E+05	2.61E+05	2.40E+05	2.67E+05	2.18E+05
Wbjp004911	C33H50O9	Hecogenone-rhamnose	Alkaloids	Alkaloids	6.71E+05	5.22E+05	6.22E+05	3.49E+06	3.45E+06	3.69E+06	2.28E+06	3.20E+06	4.13E+06	5.69E+06	3.70E+06	2.67E+06
Lamn004483	C30H38O17	Scillavone B diglucoside	Flavonoids	Other Flavonoids	4.45E+04	2.58E+04	2.32E+04	9.48E+04	6.91E+04	1.23E+05	9.74E+04	1.36E+05	8.50E+04	1.29E+05	1.01E+05	1.40E+05
pme2836	C10H11NO2	5-Hydroxytryptophol	Alkaloids	Plumerane	9.66E+05	1.11E+06	1.14E+06	1.13E+06	1.28E+06	1.11E+06	1.16E+06	1.34E+06	1.17E+06	1.40E+06	1.13E+06	1.22E+06
Lmmn001643	C9H8O3	2-Hydroxycinnamic acid*	Phenolic acids	Phenolic acids	5.28E+05	4.58E+05	5.72E+05	6.24E+05	6.00E+05	5.60E+05	5.09E+05	5.98E+05	4.64E+05	7.38E+05	5.83E+05	3.29E+05
Wccp001353	C16H19NO2	(1S,12S,14R)-4-methyl-11-oxa-4-azatetracyclo[8.6.1.01,12.06,17]heptadeca-6(17),7,9,15-tetraen-14-ol	Alkaloids	Alkaloids	4.86E+05	3.79E+05	4.04E+05	4.76E+05	5.50E+05	4.76E+05	4.20E+05	5.33E+05	5.19E+05	4.13E+05	5.39E+05	4.78E+05
pmb0501	C5H14N4	Agmatine	Alkaloids	Alkaloids	8.57E+05	1.65E+06	1.57E+06	1.83E+06	3.68E+06	2.42E+06	2.00E+06	4.14E+06	2.38E+06	2.16E+06	3.33E+06	2.19E+06
Jmwn004308	C30H38O15	4-Dihydroxyphenethoxy-8-O-β-D-[6-O-(4-O-β-D-glucopyranosyl)-feruloyl]glucopyranoside	Phenolic acids	Phenolic acids	4.68E+04	4.42E+04	4.19E+04	3.85E+04	1.04E+05	7.18E+04	5.04E+04	1.05E+05	6.35E+04	6.33E+04	1.44E+05	8.83E+04
Wbwn009733	C24H30O8	Isodunnianin	ignans and Coumarir	Lignans	4.29E+05	3.00E+05	5.01E+05	6.92E+05	7.67E+05	6.00E+05	5.24E+05	7.64E+05	5.30E+05	6.67E+05	6.78E+05	5.22E+05
Lmyp003951	C11H14O5	3-Hydroxy-1-(4-hydroxy-3,5-dimethoxyphenyl)propan-1-one	Others	Ketone compounds	4.93E+05	5.80E+05	5.73E+05	9.79E+05	1.04E+06	6.59E+05	6.54E+05	1.24E+06	8.09E+05	6.56E+05	1.03E+06	9.64E+05
Wtmp005173	C27H30O14	5,7-dihydroxy-2-(4-hydroxyphenyl)-6-[(2S,4R,5S)-3,4,5-trihydroxy-6-[[(2R,3S,5R)-3,4,5-trihydroxy-6-methyloxan-2-yl]oxymethyl]oxan-2-yl]chromen-4-one	Flavonoids	Isoflavones	1.93E+04	3.94E+04	5.04E+04	1.38E+05	4.62E+04	9.16E+04	1.27E+05	2.19E+05	1.57E+05	2.14E+05	1.08E+05	1.58E+05
HJAP050	C19H21NO5	N-Feruloyl-3-methoxytyramine	Alkaloids	Phenolamine	0.00E+00	0.00E+00	0.00E+00	1.37E+05	1.51E+05	1.13E+05	1.25E+05	1.00E+05	1.25E+05	1.07E+05	1.19E+05	1.19E+05
Lamn005190	C29H38O15	Glucosyl isomucronulatol-7-O-glucoside	Flavonoids	Other Flavonoids	7.38E+04	8.21E+04	8.40E+04	1.18E+05	1.09E+05	1.49E+05	1.43E+05	1.24E+05	1.17E+05	1.47E+05	1.37E+05	1.07E+05
mws0704	C2H8NO4P	O-Phosphorylethanolamine	Alkaloids	Alkaloids	5.43E+04	8.10E+04	7.80E+04	6.72E+04	7.40E+04	6.55E+04	6.36E+04	7.11E+04	6.62E+04	5.86E+04	7.27E+04	6.35E+04
Hmbp001276	C8H8O4	Gallacetophenone	Phenolic acids	Phenolic acids	3.10E+04	2.07E+04	3.65E+04	3.85E+04	5.00E+04	4.46E+04	3.98E+04	4.42E+04	5.89E+04	1.87E+04	2.50E+04	1.98E+04
Zmsp001834	C8H8O4	2,6-Dimethoxy-1,4-benzoquinone*	Quinones	Quinones	3.40E+05	4.25E+05	1.58E+05	3.71E+05	4.02E+05	4.04E+05	5.51E+05	5.04E+05	3.97E+05	5.26E+05	3.84E+05	3.24E+05
Lmmn003663	C14H18O9	5-Glucosyloxy-2-Hydroxybenzoic acid methyl ester	Phenolic acids	Phenolic acids	4.61E+05	4.14E+05	4.60E+05	8.61E+05	1.21E+06	1.04E+06	9.63E+05	1.16E+06	9.85E+05	9.18E+05	1.27E+06	1.22E+06
Smdp007779	C16H14O5	8-Demethylfarrerol	Flavonoids	Flavanones	0.00E+00	0.00E+00	0.00E+00	4.42E+04	3.58E+05	1.60E+05	2.78E+04	4.49E+05	1.45E+05	2.57E+04	3.95E+05	1.36E+05
Lajp005753	C23H24O12	5,3'-dihydroxy-6,7,4'-trimethoxyflavone-8-O-β-D-glucoside	Flavonoids	Flavones	1.14E+04	3.82E+04	3.81E+04	2.22E+05	3.42E+05	2.75E+05	2.97E+05	3.30E+05	2.54E+05	3.02E+05	2.74E+05	3.03E+05
Hmgp002036	C22H22O12	Nepetin-7-O-glucoside(Nepitrin)	Flavonoids	Flavones	0.00E+00	0.00E+00	0.00E+00	3.55E+05	7.87E+05	8.81E+05	4.41E+05	6.86E+05	1.09E+06	4.48E+05	7.38E+05	7.39E+05
Wbmp010544	C37H50N2O11	10-Hydroxymethyllycaconitine	Alkaloids	Alkaloids	5.85E+05	4.43E+05	6.96E+05	1.21E+07	1.29E+07	1.35E+07	1.19E+07	1.40E+07	1.48E+07	1.43E+07	1.55E+07	1.53E+07
Labn004865	C21H22O10	Hydroxy isoliquiritigenin glucoside	Flavonoids	Chalcones	0.00E+00	0.00E+00	0.00E+00	2.62E+04	1.05E+05	7.94E+04	4.12E+04	1.41E+05	9.87E+04	3.05E+04	1.60E+05	7.09E+04
mws0853	C11H14O4	Sinapyl alcohol	Phenolic acids	Phenolic acids	6.43E+04	3.87E+04	5.18E+04	3.02E+04	3.24E+04	4.69E+04	3.76E+04	4.47E+04	3.15E+04	1.53E+04	3.73E+04	3.54E+04
MW0133705	C16H12O3	4'-Methoxyflavone	Flavonoids	Flavones	7.92E+03	6.07E+03	6.45E+03	4.85E+04	6.14E+04	4.97E+04	4.26E+04	5.41E+04	4.98E+04	5.85E+04	5.86E+04	4.48E+04
Qabp004989	C51H82O23	Dehydroxy Epiruscogenin-glucose-glucose-glucose-glucoside	Steroids	Steroidal saponins	2.05E+05	1.40E+05	1.44E+05	7.74E+05	7.73E+05	6.60E+05	6.01E+05	7.46E+05	1.26E+06	6.88E+05	9.14E+05	9.75E+05
Wagp007639	C17H17NO2	N-(4-hydroxyphenethyl)cinnamamide*	Alkaloids	Alkaloids	0.00E+00	0.00E+00	0.00E+00	3.08E+04	2.18E+05	1.09E+05	2.32E+04	2.29E+05	9.32E+04	4.02E+04	2.37E+05	8.44E+04
Zjhp082619	C33H52O9	Pennogenin-Glc	Steroids	Steroidal saponins	1.12E+06	1.23E+06	1.45E+06	3.28E+06	4.42E+06	3.18E+06	3.87E+06	1.45E+06	2.63E+06	3.09E+06	3.91E+06	4.46E+06
Saln004355	C26H32O12	Luzonoside C	Terpenoids	Monoterpenoids	5.48E+05	4.43E+05	4.16E+05	5.16E+05	6.42E+05	4.75E+05	4.63E+05	6.50E+05	5.16E+05	5.14E+05	6.41E+05	5.55E+05
pme2122	C5H9N3	Histamine	Alkaloids	Alkaloids	5.80E+04	9.76E+04	9.56E+04	7.10E+04	1.11E+05	8.96E+04	5.65E+04	1.08E+05	7.27E+04	9.18E+04	1.06E+05	6.08E+04
Wcsn010349	C22H32O4	Moniliferanone D	Others	Others	0.00E+00	0.00E+00	0.00E+00	1.18E+07	1.42E+07	1.11E+07	1.18E+07	1.51E+07	1.29E+07	1.48E+07	1.62E+07	1.24E+07
Hmcp005535	C30H48O	α-Amyrenone	Terpenoids	Triterpene	6.78E+04	5.46E+04	5.60E+04	8.56E+04	6.90E+04	5.60E+04	7.71E+04	7.26E+04	7.76E+04	8.42E+04	6.69E+04	6.58E+04
MWSHY0163	C15H12O6	Aromadendrin (Dihydrokaempferol)	Flavonoids	Flavanonols	8.01E+04	7.87E+04	6.08E+04	2.33E+05	3.19E+05	3.12E+05	1.83E+05	2.12E+05	2.02E+05	2.09E+05	2.25E+05	1.62E+05
Wcfp002357	C15H20N6O8	N6-Threonylcarbamoyladenosine	Others	Others	3.17E+05	2.89E+05	2.78E+05	3.50E+05	4.40E+05	3.38E+05	3.92E+05	4.11E+05	4.13E+05	3.89E+05	4.33E+05	4.63E+05
Wbmp005699	C22H22O10	Oxoflaccidin-glucose	Quinones	PhenAnthraquinones	7.16E+04	2.24E+04	4.12E+04	9.08E+05	6.52E+06	4.17E+06	1.10E+06	5.52E+06	3.86E+06	9.36E+05	7.72E+06	3.74E+06
Hmsn002598	C13H18O8	Salirepin	Phenolic acids	Phenolic acids	5.87E+04	6.47E+04	6.97E+04	1.02E+05	7.19E+04	8.65E+04	1.03E+05	9.87E+04	8.69E+04	9.81E+04	9.28E+04	7.54E+04
Lmgp004152	C27H30O15	Diosmetin-8-C-(2''-O-arabinosyl)glucoside	Flavonoids	Flavones	4.68E+04	5.31E+04	6.20E+04	1.43E+05	2.01E+05	1.70E+05	1.64E+05	2.32E+05	1.85E+05	1.52E+05	1.45E+05	1.72E+05
Zjmp102024	C17H14O5	Desmethyl-iso-ophiopogonone B*	Flavonoids	Other Flavonoids	0.00E+00	0.00E+00	0.00E+00	1.23E+05	1.71E+05	1.02E+05	1.21E+05	1.80E+05	1.13E+05	1.14E+05	1.54E+05	8.61E+04
pmb0475	C29H34O18	Gallic acid-4-O-(6'''-feruloyl)sophoroside	Phenolic acids	Phenolic acids	8.86E+04	9.70E+04	7.71E+04	2.53E+05	2.12E+05	2.91E+05	2.72E+05	2.20E+05	3.01E+05	3.30E+05	2.47E+05	2.94E+05
Hmgn004693	C20H20O7	8-Hydroxy-α-conidendrin	ignans and Coumarir	Lignans	1.49E+04	2.09E+04	2.01E+04	8.38E+04	8.21E+04	8.12E+04	7.28E+04	6.20E+04	7.72E+04	8.88E+04	6.08E+04	5.52E+04
mws4085	C11H12O5	Sinapic acid	Phenolic acids	Phenolic acids	8.15E+04	7.48E+04	5.23E+04	9.86E+04	1.04E+05	1.02E+05	1.08E+05	1.00E+05	7.92E+04	1.01E+05	8.61E+04	8.55E+04
Zjhp082643	C45H70O18	Gentrogenin-Glc-Glc-Rha	Steroids	Steroidal saponins	6.33E+04	5.24E+04	8.06E+04	8.99E+04	1.51E+05	2.86E+04	8.38E+04	7.84E+04	1.31E+05	1.27E+05	1.20E+05	1.48E+05
mws1358	C6H6O2	Pyrocatechol	Phenolic acids	Phenolic acids	1.78E+05	1.60E+05	1.08E+05	1.91E+05	2.04E+05	1.88E+05	1.57E+05	1.21E+05	1.61E+05	1.60E+05	1.84E+05	1.30E+05
Lazn006152	C20H22O5	Isoshonanin	ignans and Coumarir	Lignans	4.13E+05	6.30E+05	6.37E+05	5.16E+05	5.97E+05	4.97E+05	4.85E+05	5.85E+05	5.46E+05	5.59E+05	6.61E+05	4.37E+05
Lmgn001670	C7H6O3	Salicylic acid	Phenolic acids	Phenolic acids	3.17E+05	2.84E+05	3.36E+05	4.26E+05	5.12E+05	4.29E+05	4.63E+05	3.94E+05	4.16E+05	4.76E+05	4.83E+05	5.49E+05
WaYn002313	C16H22O10	Homosyringic Acid 4'-O-Glucoside	Phenolic acids	Phenolic acids	2.23E+05	1.80E+05	1.65E+05	4.26E+05	4.54E+05	4.53E+05	4.39E+05	3.93E+05	4.36E+05	4.39E+05	3.62E+05	4.22E+05
Lazp004399	C51H80O24	Tupichinin B	Steroids	Steroidal saponins	1.58E+06	9.02E+05	7.36E+05	4.98E+06	2.97E+06	3.36E+06	3.94E+06	2.42E+06	2.55E+06	4.66E+06	2.66E+06	4.09E+06
Lmmn009932	C31H50O3	3-Hydroxy-24-methylene-9,19-cyclolanostan-26-oic acid (Ambolic acid)	Terpenoids	Triterpene	5.68E+03	4.85E+03	4.25E+03	1.75E+04	1.06E+04	1.60E+04	1.32E+04	1.44E+04	1.86E+04	1.67E+04	1.50E+04	1.66E+04
Lazn002951	C17H20O6	Guaiacylglycerol-β-Guaiacyl Ether	ignans and Coumarir	Lignans	4.44E+04	5.19E+04	1.79E+04	7.06E+04	7.13E+04	6.69E+04	7.19E+04	7.00E+04	5.30E+04	6.62E+04	6.95E+04	4.62E+04
Wcdn004423	C28H38O13	(2S,3R,4R)-3a-[(Beta-D-Glucopyranosyl)Oxy]Lyoniresinol	ignans and Coumarir	Lignans	8.36E+04	3.39E+04	5.10E+04	5.11E+04	1.00E+05	6.82E+04	1.06E+05	8.08E+04	6.93E+04	8.69E+04	4.80E+04	1.09E+05
Lamp008416	C16H14O6	3-(4-hydroxy)-5,7-dihydroxy-9-hydroxychroman-4-one	Flavonoids	Other Flavonoids	0.00E+00	0.00E+00	0.00E+00	1.78E+05	4.16E+06	1.57E+06	1.10E+05	4.57E+06	1.68E+06	1.01E+05	4.56E+06	1.34E+06
Wcsn011122	C18H32O2	(5Z,10E,14S)-14-hydroxy-2,6,10-trimethylpentadeca-5,10-dien-4-one	Others	Others	2.32E+05	1.57E+05	1.28E+05	8.43E+07	8.17E+07	7.48E+07	8.46E+07	9.30E+07	8.79E+07	8.83E+07	9.52E+07	8.21E+07
pmn001687	C13H12O8	Cis-Coutaric acid	Phenolic acids	Phenolic acids	2.53E+05	2.59E+05	2.55E+05	3.42E+05	4.03E+05	3.90E+05	3.05E+05	3.99E+05	3.60E+05	2.76E+05	4.21E+05	3.67E+05
Jmyn004445	C56H92O29	Furost-5-ene-3,22,26-triol-3-O-glucosyl(1→2)[xylosyl(1→3)]glucosyl(1→4)galactoside-26-O-glucoside (Timosaponin H1)	Steroids	Steroidal saponins	7.90E+04	3.75E+04	1.57E+04	1.89E+05	1.20E+05	1.07E+05	1.18E+05	2.00E+05	1.53E+05	1.60E+05	2.39E+05	1.25E+05
Lmmn003748	C20H24O6	Isolariciresinol	ignans and Coumarir	Lignans	1.76E+04	1.18E+04	2.06E+04	3.43E+04	3.55E+04	3.86E+04	4.08E+04	2.00E+04	3.04E+04	4.35E+04	3.09E+04	5.60E+04
mws0017	C7H19N3	Spermidine	Alkaloids	Alkaloids	4.09E+05	4.16E+05	3.82E+05	5.48E+05	5.78E+05	5.83E+05	4.88E+05	5.50E+05	6.60E+05	3.74E+05	5.08E+05	5.00E+05
Wbjp004916	C17H12N2O4	Cephalanthrin A	Alkaloids	Alkaloids	0.00E+00	0.00E+00	0.00E+00	9.36E+06	1.26E+07	1.05E+07	9.58E+06	1.24E+07	1.09E+07	8.43E+06	1.24E+07	8.03E+06
Zjhp090623	C20H22O6	5-hydroxy-7-methoxy-6,8-dimethyl-3-(2'-hydroxy-4'-methoxybenzyl)-chroman-4-one	Flavonoids	Other Flavonoids	0.00E+00	0.00E+00	0.00E+00	4.90E+04	9.65E+04	1.58E+05	6.22E+04	1.09E+05	1.33E+05	8.22E+04	1.08E+05	1.25E+05
Wbmp008021	C26H39NO7	(14-Ethyl-4,6,19-trimethoxy-9,11-dioxa-14-azaheptacyclo[10.7.2.12,5.01,13.03,8.08,12.016,20]docosan-21-yl)acetate	Alkaloids	Alkaloids	0.00E+00	0.00E+00	0.00E+00	3.76E+06	4.05E+06	3.85E+06	4.04E+06	4.42E+06	4.93E+06	4.86E+06	4.89E+06	5.04E+06
Hmhp006479	C17H16O6	2-(2,4-dihydroxybenzyl)-6,8-dihydroxy-7-methyl-3,4-dihydronaphthalen-1(2H)-one	Flavonoids	Other Flavonoids	0.00E+00	0.00E+00	0.00E+00	1.09E+05	1.44E+05	1.41E+05	7.80E+04	1.97E+05	1.38E+05	1.17E+05	1.99E+05	1.39E+05
Lcsp002966	C7H10O2	2-Methylcyclohexane-1,3-dione	Others	Ketone compounds	3.10E+06	2.79E+06	6.30E+06	3.42E+06	2.65E+06	2.70E+06	3.03E+06	2.46E+06	3.08E+06	2.67E+06	3.49E+06	2.90E+06
Zmxp005324	C20H24O6	Dihydrodehydrodiconiferyl alcohol	ignans and Coumarir	Lignans	1.15E+05	4.78E+04	3.58E+04	1.58E+05	1.23E+05	9.00E+04	1.74E+05	1.84E+05	1.73E+05	1.32E+05	1.11E+05	1.48E+05
pmn001384	C25H24O12	Isochlorogenic acid C	Phenolic acids	Phenolic acids	2.73E+04	2.75E+04	2.56E+04	6.53E+04	9.84E+04	7.17E+04	5.53E+04	8.38E+04	7.26E+04	8.25E+04	5.43E+04	1.02E+05
pmp000509	C6H6O3	Maltol	Others	Ketone compounds	4.75E+06	2.92E+06	7.52E+06	5.62E+06	4.34E+06	5.71E+06	5.36E+06	5.78E+06	6.65E+06	4.33E+06	5.78E+06	5.87E+06
Cmlp002704	C24H30N4	Folicanthine	Alkaloids	Plumerane	6.95E+04	3.28E+04	1.00E+05	1.49E+05	1.76E+05	1.29E+05	1.08E+05	2.26E+05	2.22E+05	8.55E+04	1.12E+05	8.58E+04
MWSmce712	C10H12O2	Ethyl phenylacetate	Phenolic acids	Phenolic acids	1.76E+06	1.90E+06	2.19E+06	1.55E+06	1.88E+06	1.94E+06	1.70E+06	1.71E+06	2.18E+06	1.88E+06	2.10E+06	2.02E+06
Lmwp011196	C24H38O4	Bis(2-ethylhexyl)phthalate*	Phenolic acids	Phenolic acids	7.88E+05	2.49E+05	3.83E+05	1.87E+05	1.23E+06	9.91E+05	7.25E+05	1.19E+06	1.13E+06	9.97E+05	7.20E+05	1.61E+06
		(3S)-5,7-dihydroxy-3-[(4-[illegible]														

Zjhp090611	C18H18O5	[illegible]methoxyphenyl)methyl]-6-methyl-2,3-dihydrochromen-4-one*	Flavonoids	Other Flavonoids	0.00E+00	0.00E+00	0.00E+00	2.83E+05	3.54E+05	2.91E+05	3.28E+05	4.42E+05	3.34E+05	2.78E+05	4.11E+05	3.30E+05
mws1292	C26H28O14	Isoschaftoside	Flavonoids	Flavones	1.46E+05	1.61E+05	1.57E+05	6.84E+05	5.27E+05	7.17E+05	7.07E+05	7.79E+05	6.51E+05	5.11E+05	5.67E+05	6.82E+05
Hahn001170	C15H22O9	Aucubin	Terpenoids	Sesquiterpenoids	3.53E+05	2.91E+05	3.11E+05	4.69E+05	5.14E+05	4.76E+05	5.02E+05	5.14E+05	4.82E+05	5.21E+05	5.12E+05	5.14E+05
Smnp003415	C20H24O9	7-O-Methylaloesin	Flavonoids	Other Flavonoids	1.63E+05	1.69E+05	1.68E+05	1.56E+05	7.13E+04	1.08E+05	9.41E+04	1.17E+05	3.24E+04	9.33E+04	8.81E+04	4.34E+04
Lcdn000131	C15H20O7	sachaliside	Others	Others	2.32E+05	2.82E+05	1.84E+05	3.51E+05	4.02E+05	3.57E+05	3.11E+05	3.56E+05	2.62E+05	2.15E+05	2.59E+05	2.39E+05
Lajp004422	C28H32O15	Hirsutissimiside C	Flavonoids	Isoflavones	2.41E+04	1.89E+04	1.90E+04	6.98E+04	3.69E+05	2.80E+05	8.77E+04	3.72E+05	2.78E+05	5.12E+04	4.59E+05	1.63E+05
pmn001690	C16H24O7	3-Hydroxy-4-isopropylbenzylalcohol-3-O-glucoside	Phenolic acids	Phenolic acids	1.05E+05	8.36E+04	1.12E+05	1.14E+05	1.51E+05	1.20E+05	1.37E+05	1.26E+05	1.05E+05	1.24E+05	1.05E+05	1.25E+05
mws0102	C9H7NO2	Indole-5-carboxylic acid	Alkaloids	Plumerane	2.06E+04	1.48E+04	2.53E+04	2.77E+04	3.26E+04	1.89E+04	1.77E+04	2.27E+04	3.03E+04	5.23E+04	3.30E+04	2.20E+04
Zjhp090634	C18H16O6	(E)-3-(3',4'-dihydroxybenzylidene)-5,7-dihydroxy-6,8-dimethylchroman-4-	Flavonoids	Other Flavonoids	0.00E+00	0.00E+00	0.00E+00	6.78E+04	4.37E+04	5.50E+04	4.94E+04	6.22E+04	6.71E+04	7.39E+04	5.24E+04	6.92E+04
Zjhp090604	C16H14O5	5,7-dihydroxy-3-(4'-hydroxybenzyl)-chroman-4-one	Flavonoids	Other Flavonoids	0.00E+00	0.00E+00	0.00E+00	3.22E+04	3.29E+05	1.52E+05	2.77E+04	4.08E+05	1.36E+05	3.77E+04	3.98E+05	1.44E+05
pme1292	C8H8O4	Homogentisic acid*	Phenolic acids	Phenolic acids	8.34E+05	6.39E+05	6.11E+05	1.25E+06	1.82E+06	1.42E+06	1.22E+06	1.41E+06	1.30E+06	1.47E+06	1.53E+06	1.48E+06
MWSmce265	C14H14O4	Decursinol	ignans and Coumarir	Coumarins	1.96E+06	2.20E+06	2.08E+06	2.08E+06	1.93E+06	1.95E+06	1.76E+06	2.20E+06	1.46E+06	1.60E+06	1.85E+06	2.12E+06
Zjzp081816	C39H64O13	Smilagenin-3-O-glucosyl(1→2)mannoside (Smilageninoside)	Steroids	Steroidal saponins	2.58E+05	3.29E+05	2.85E+05	1.17E+06	2.47E+06	2.22E+06	1.74E+06	2.64E+06	2.10E+06	1.02E+06	1.67E+06	1.59E+06
Hmdp001676	C17H17NO4	N-p-Coumaroyloctopamine	Alkaloids	Phenolamine	6.59E+05	6.91E+05	6.68E+05	7.59E+05	5.05E+05	7.13E+05	7.25E+05	8.83E+05	5.74E+05	8.42E+05	8.80E+05	7.48E+05
Zjzp081803	C27H44O3	3-hydroxy-20-ene-Furostanol	Steroids	Steroid	9.52E+04	4.67E+04	7.44E+04	2.20E+05	2.52E+05	2.64E+05	1.76E+05	2.09E+05	2.60E+05	1.30E+05	2.27E+05	1.97E+05
MWS3149	C8H8O3	3-Methoxybenzoic acid	Phenolic acids	Phenolic acids	6.72E+04	2.87E+04	2.56E+04	7.16E+04	8.31E+04	1.48E+05	7.44E+04	9.89E+04	1.01E+05	1.22E+05	1.25E+05	1.52E+05
pme1261	C9H19NO4	D-Panthenol	Others	Alcohol compounds	2.06E+05	3.18E+05	1.78E+04	1.52E+05	1.77E+05	1.12E+05	2.61E+05	9.44E+04	1.96E+05	7.80E+04	8.65E+04	1.33E+05
Zjhp090617	C18H18O6	5-hydroxy-3-(2-hydroxy-4-methoxybenzyl)-7-methoxychroman-4-one	Flavonoids	Other Flavonoids	0.00E+00	0.00E+00	0.00E+00	2.23E+04	6.94E+05	1.09E+05	2.96E+04	9.48E+05	2.01E+05	3.46E+04	9.13E+05	1.35E+05
MWSmce338	C6H11NO3	N-Hydroxypipecolic acid	Alkaloids	Piperidine alkaloids	9.86E+05	8.45E+05	8.55E+05	1.16E+06	9.73E+05	9.43E+05	1.10E+06	1.12E+06	1.27E+06	1.05E+06	1.41E+06	1.09E+06
Ymsp002308	C16H19NO3	4'-O-Methylnorbelladine	Alkaloids	Alkaloids	1.07E+06	1.01E+06	9.01E+05	2.06E+06	2.05E+06	2.30E+06	2.02E+06	1.94E+06	1.91E+06	3.33E+06	1.59E+06	1.78E+06
pmn001338	C21H18O11	Rhein-8-O-glucoside	Quinones	Anthraquinone	1.42E+04	2.61E+04	5.10E+04	1.08E+05	1.35E+05	1.18E+05	6.97E+04	1.35E+05	6.88E+04	9.65E+04	1.46E+05	1.17E+05
Lmcp005645	C17H14O7	Quercetin-3,4'-Dimethyl Ether	Flavonoids	Flavonols	1.50E+04	1.69E+04	9.13E+03	3.44E+05	5.37E+05	4.44E+05	5.15E+05	6.58E+05	5.78E+05	4.78E+05	6.41E+05	4.96E+05
Zjzp081806	C33H54O8	Sarsasapogenin-Glc	Steroids	Steroidal saponins	7.49E+04	7.24E+04	7.70E+04	3.14E+05	2.65E+05	4.83E+05	3.09E+05	2.59E+05	5.31E+05	2.72E+05	3.98E+05	3.94E+05
WaYn006850	C32H38O12	Buddlenol F*	ignans and Coumarir	Lignans	1.59E+04	1.74E+04	1.29E+04	3.38E+04	3.18E+04	3.81E+04	4.11E+04	3.06E+04	4.77E+04	3.93E+04	5.24E+04	4.22E+04
Lmzn001582	C18H28O9	5'-Glucosyloxyjasmanic	Phenolic acids	Phenolic acids	7.07E+04	4.62E+04	4.26E+04	1.74E+05	1.54E+05	1.08E+05	1.48E+05	1.43E+05	1.57E+05	1.73E+05	1.43E+05	8.94E+04
pmb1912	C20H23N7O7	10-Formyltetrahydrofolic Acid	Alkaloids	Alkaloids	6.05E+04	6.20E+04	5.77E+04	1.28E+05	8.60E+04	1.31E+05	7.32E+04	8.65E+04	1.35E+05	9.15E+04	6.96E+04	7.42E+04
Qmgp102504	C20H17N3O2	Angustoline	Alkaloids	Plumerane	4.11E+05	3.62E+05	4.13E+05	1.38E+06	1.61E+06	1.53E+06	1.59E+06	1.59E+06	1.51E+06	1.45E+06	1.53E+06	1.47E+06
pmf0557	:16H11N2NaO4	Orange I	Alkaloids	Alkaloids	0.00E+00	0.00E+00	0.00E+00	2.79E+04	2.21E+05	1.34E+05	3.26E+04	1.79E+05	9.72E+04	4.67E+04	2.44E+05	7.72E+04
mws0043	C21H22O8	Nobiletin (5,6,7,8,3',4'-Hexamethoxyflavone)	Flavonoids	Flavones	1.54E+04	1.56E+04	2.29E+04	6.49E+04	5.81E+04	4.67E+04	6.13E+04	6.82E+04	5.11E+04	4.52E+04	5.04E+04	4.66E+04
pme2268	C7H7NO2	Trigonelline	Alkaloids	Pyridine alkaloids	2.74E+05	2.69E+05	3.06E+05	3.70E+05	4.43E+05	4.53E+05	4.10E+05	4.38E+05	4.30E+05	4.28E+05	3.96E+05	4.13E+05
Wchn006138	C20H22O5	4,9-Dihydroxy-17-methoxy-2-oxatricyclo[13.2.2.13,7]icoSa-1(17),3,5,7(20),15,18-hexaen-10-one	Others	Others	9.14E+04	7.88E+04	5.41E+04	1.36E+05	1.42E+05	1.37E+05	2.68E+05	1.76E+05	2.25E+05	1.44E+05	1.87E+05	1.28E+05
MWSHY0132	C21H20O11	Quercetin-3-O-rhamnoside(Quercitrin)	Flavonoids	Flavonols	9.41E+04	6.25E+04	5.64E+04	1.71E+05	2.32E+05	2.27E+05	1.91E+05	2.28E+05	1.96E+05	2.26E+05	2.30E+05	2.92E+05
Lmtp002474	C26H28O14	Apigenin-6-C-(2''-glucosyl)arabinoside	Flavonoids	Flavones	1.72E+05	1.37E+05	1.55E+05	8.37E+05	1.01E+06	7.39E+05	7.17E+05	7.35E+05	7.11E+05	7.60E+05	8.65E+05	9.58E+05
mws0014	C10H10O4	Ferulic acid*	Phenolic acids	Phenolic acids	1.65E+05	1.73E+05	1.35E+05	2.92E+05	3.68E+05	3.05E+05	2.44E+05	3.76E+05	2.85E+05	2.62E+05	2.74E+05	3.29E+05
Zmpn003044	C9H6O3	7-Hydroxycoumarin;Umbelliferone	ignans and Coumarir	Coumarins	1.29E+04	1.21E+04	2.21E+04	6.59E+04	8.17E+04	8.97E+04	6.89E+04	6.50E+04	1.06E+05	8.80E+04	7.58E+04	3.69E+04
mws1375	C12H21N3O6	Nicotianamine	Alkaloids	Alkaloids	3.75E+05	4.50E+05	3.64E+05	3.77E+05	2.83E+05	3.82E+05	2.79E+05	3.69E+05	5.00E+05	3.11E+05	3.18E+05	3.09E+05
Hmdn001667	C20H16O13	Ellagic acid-4-O-glucoside	Tannins	Tannin	1.40E+04	1.56E+04	2.11E+04	6.21E+04	2.44E+05	2.44E+05	8.30E+04	2.96E+05	2.12E+05	4.69E+04	2.96E+05	2.30E+05
mws0066	C16H12O7	Isorhamnetin; 3'-Methoxy-3,4',5,7-Tetrahydroxyflavone	Flavonoids	Flavonols	0.00E+00	0.00E+00	0.00E+00	2.78E+04	8.05E+04	1.99E+04	3.39E+04	9.24E+04	3.17E+04	8.21E+03	8.81E+04	2.07E+04
Labn002291	C19H26O13	Rhamnosyl-gentisic acid-5-O-β-D-	Phenolic acids	Phenolic acids	1.27E+05	6.92E+04	6.21E+04	6.27E+04	1.25E+05	1.10E+05	5.67E+04	1.15E+05	1.26E+05	5.08E+04	8.54E+04	1.49E+05
MWSHY0136	C21H20O11	Kaempferol-3-O-glucoside (Astragalin)	Flavonoids	Flavonols	2.02E+04	9.91E+03	3.21E+04	1.01E+05	3.86E+04	1.01E+05	9.29E+04	7.74E+04	3.12E+04	6.36E+04	6.59E+04	1.50E+05
Hmgp002950	C25H33N3O6	N1-Caffeoyl-N10-dihydrocaffeoylspermidine	Alkaloids	Phenolamine	9.29E+04	7.28E+04	8.10E+04	1.83E+05	1.47E+05	1.57E+05	2.13E+05	1.68E+05	1.80E+05	1.65E+05	1.27E+05	2.36E+05
HJAP056	C23H24O11	Dihydroxy-dimethoxyflavone-7-O-glucoside*	Flavonoids	Flavones	2.70E+04	2.94E+04	2.01E+04	1.17E+05	1.47E+05	1.95E+05	1.05E+05	1.51E+05	1.60E+05	1.26E+05	2.11E+05	1.22E+05
Lmtn002324	C19H28O11	Benzyl-(2''-O-glucosyl)glucoside*	Phenolic acids	Phenolic acids	2.77E+05	3.15E+05	2.41E+05	1.49E+05	1.69E+05	1.26E+05	1.75E+05	1.08E+05	1.08E+05	1.12E+05	1.42E+05	1.13E+05
Hmqn000843	C13H18O8	Tachioside*	Phenolic acids	Phenolic acids	1.95E+05	1.86E+05	1.47E+05	2.27E+05	2.51E+05	2.77E+05	2.13E+05	2.73E+05	2.50E+05	2.65E+05	2.50E+05	2.23E+05
MWSHC2029	C13H10N2O2	Harman-3-carboxylic acid	Alkaloids	Plumerane	2.78E+04	2.55E+04	5.22E+04	1.24E+05	1.56E+05	1.36E+05	1.57E+05	2.23E+05	1.69E+05	1.67E+05	1.66E+05	1.30E+05
Zjhp082686	C51H80O23	iso-14-Dehydroxy-neoprazerigenin A-Glc-Glc-Glc-Glc	Steroids	Steroidal saponins	6.01E+04	1.05E+04	5.26E+04	1.63E+05	2.84E+05	3.90E+05	2.05E+05	1.83E+05	2.62E+05	2.15E+05	2.76E+05	3.07E+05
Zjhp090625	C22H22O10	(E)-7-O-β-D-glucopyranoside-5-hydroxy-3-(4'-hydroxybenzylidene)-chroman-4-one	Flavonoids	Other Flavonoids	3.21E+04	3.68E+04	2.42E+04	7.88E+05	4.68E+06	3.42E+06	1.13E+06	5.25E+06	3.89E+06	9.24E+05	6.18E+06	3.49E+06
Yaap009385	C24H30O6	Myrtucommulone B	Others	Others	1.00E+06	1.14E+06	9.33E+05	6.04E+06	8.87E+06	6.98E+06	7.19E+06	6.02E+06	6.90E+06	8.84E+06	4.63E+06	8.54E+06
pmn001671	C23H32O15	Furanofructosyl-α-D-(6-mustard acyl)glucoside	Phenolic acids	Phenolic acids	3.36E+04	2.16E+04	1.42E+04	4.03E+04	3.29E+04	2.62E+04	3.19E+04	3.85E+04	3.12E+04	4.17E+04	3.22E+04	3.47E+04
WaYn003084	C24H30O13	Guaiacylglycerol 8-O-(1''-O-Glucosyl)Vanillic Acid	Phenolic acids	Phenolic acids	6.21E+04	3.92E+04	4.45E+04	1.25E+05	9.58E+04	1.33E+05	1.42E+05	1.07E+05	9.83E+04	1.27E+05	1.17E+05	1.22E+05
Zmcp002839	C33H41O22+	Delphinidin-3,5,3'-Tri-O-glucoside	Flavonoids	Anthocyanidins	4.60E+04	5.38E+04	4.05E+04	1.84E+05	1.93E+05	2.15E+05	1.66E+05	2.02E+05	2.28E+05	1.91E+05	2.06E+05	2.22E+05
pmf0440	C10H10O2	4-MethoxycinnaMaldehyde	Others	Aldehyde compounds	2.15E+06	2.41E+06	2.51E+06	2.25E+06	2.29E+06	2.38E+06	2.14E+06	2.42E+06	2.16E+06	2.39E+06	2.45E+06	2.68E+06
MWSHY0046	C21H20O12	Quercetin-3-O-glucoside (Isoquercitrin)*	Flavonoids	Flavonols	1.73E+05	2.18E+05	1.03E+05	2.10E+05	4.53E+05	4.25E+05	3.01E+05	4.61E+05	3.79E+05	2.73E+05	5.19E+05	2.33E+05
Lmyp005841	C21H20O9	Chrysin-7-O-glucoside	Flavonoids	Flavones	1.19E+04	1.82E+04	2.20E+04	9.12E+04	9.99E+04	8.58E+04	3.76E+04	5.28E+04	7.08E+04	5.66E+04	1.23E+05	6.00E+04
pmp001285	C8H4O3	Phthalic anhydride	Phenolic acids	Phenolic acids	2.37E+07	2.96E+07	3.00E+07	2.18E+07	2.93E+07	2.22E+07	2.05E+07	1.94E+07	2.32E+07	2.67E+07	2.72E+07	2.39E+07
pmp000087	C13H16O6	2-Feruloyl-sn-glycerol	Phenolic acids	Phenolic acids	1.82E+05	1.12E+05	1.95E+05	1.73E+05	1.44E+05	1.18E+05	9.76E+04	1.45E+05	1.35E+05	1.68E+05	1.26E+05	1.58E+05
Lmgp004474	C27H30O14	Genistein-7-O-galactoside-rhamnose	Flavonoids	Isoflavones	5.61E+04	4.77E+04	5.01E+04	1.73E+05	2.21E+05	2.86E+05	1.59E+05	1.45E+05	2.63E+05	1.94E+05	1.89E+05	1.84E+05
mws0467	C9H10O3	3-(4-Hydroxyphenyl)-propionic acid	Phenolic acids	Phenolic acids	3.03E+04	2.13E+04	2.12E+04	1.24E+05	1.78E+05	1.70E+05	1.54E+05	2.00E+05	1.34E+05	1.35E+05	1.77E+05	1.50E+05
Wayp003244	C9H9NO3	3-Succinoylpyridine	Alkaloids	Pyridine alkaloids	7.80E+05	7.64E+05	8.42E+05	6.75E+05	5.55E+05	4.89E+05	4.18E+05	4.46E+05	5.16E+05	3.11E+05	3.22E+05	3.68E+05
mws1179	C21H22O10	Naringenin-7-O-glucoside (Prunin)	Flavonoids	Flavanones	0.00E+00	0.00E+00	0.00E+00	2.74E+04	1.02E+05	9.19E+04	3.33E+04	9.54E+04	6.95E+04	3.90E+04	1.01E+05	8.96E+04
pmb0382	C19H14O6	O-Feruloyl 4-hydroxycoumarin	ignans and Coumarir	Coumarins	0.00E+00	0.00E+00	0.00E+00	1.77E+05	1.57E+05	2.21E+05	3.71E+05	4.71E+05	4.58E+05	4.84E+05	5.15E+05	4.96E+05
Jmwn002172	C14H20O8	3,4-dihydroxyphenylethanol-β-D-glucopyranoside	Phenolic acids	Phenolic acids	4.46E+04	4.46E+04	5.89E+04	9.04E+04	8.20E+04	9.47E+04	9.18E+04	7.19E+04	7.41E+04	1.22E+05	1.04E+05	8.35E+04
HJN037	C8H8O5	3,5-Dihydroxy-4-methoxybenzoic acid; 4-O-Methylgallic Acid	Phenolic acids	Phenolic acids	5.14E+04	3.26E+04	4.53E+04	2.87E+04	4.88E+04	2.73E+04	1.71E+04	1.92E+04	2.75E+04	2.87E+04	2.80E+04	3.53E+04
mws0046	C27H32O14	Naringenin-7-O-Neohesperidoside(Naringin)	Flavonoids	Flavanones	9.56E+03	1.03E+04	1.16E+04	2.51E+04	1.37E+04	1.45E+04	2.97E+04	2.27E+04	8.56E+03	1.89E+04	2.25E+04	1.09E+04
pmn001511	C13H18O7	3-Hydroxy-5-Methylphenol-1-O-Glucoside	Phenolic acids	Phenolic acids	1.48E+06	9.08E+05	9.51E+05	2.68E+06	1.88E+06	2.56E+06	2.93E+06	3.51E+06	2.97E+06	2.42E+06	3.52E+06	3.01E+06
Wcfn003850	C19H28O9	Corchoionoside B	Others	Others	3.61E+04	5.87E+04	3.85E+04	1.09E+05	8.97E+04	1.31E+05	1.48E+05	1.66E+05	1.35E+05	1.41E+05	1.20E+05	1.59E+05
Ymjm000154	C13H18O7	Salicin	Phenolic acids	Phenolic acids	1.72E+06	1.44E+06	9.28E+05	3.47E+06	2.47E+06	2.72E+06	4.46E+06	3.56E+06	2.39E+06	3.36E+06	4.52E+06	3.88E+06
pmp000436	C30H50O	13-Methyl-27-norolean-14-en-3-ol (Taraxerol)	Terpenoids	Triterpene	0.00E+00	0.00E+00	0.00E+00	8.19E+04	7.99E+04	6.78E+04	7.59E+04	7.03E+04	5.64E+04	1.05E+05	6.64E+04	6.07E+04
Yaln008277	C22H34O6	Trijugin A	Terpenoids	Sesquiterpenoids	7.11E+04	4.79E+04	3.80E+04	2.82E+05	3.61E+05	2.32E+05	4.85E+05	3.45E+05	3.86E+05	3.29E+05	3.09E+05	3.16E+05
pmn001692	C18H19NO5	3-O-Acetylhamayne	Alkaloids	Isoquinoline alkaloids	2.12E+04	1.85E+04	1.79E+04	1.73E+05	1.86E+05	1.77E+05	1.84E+05	1.68E+05	1.91E+05	1.49E+05	1.40E+05	1.53E+05
Qabp004961	C57H92O28	Dehydroxy Epiruscogenin-glucose-glucose-glucose-glucose-glucoside	Steroids	Steroidal saponins	7.56E+05	1.00E+06	7.38E+05	1.43E+06	5.09E+06	3.33E+06	3.86E+06	4.08E+06	4.45E+06	4.22E+06	5.57E+06	5.20E+06
Lmtp002822	C25H26O13	Apigenin-6-C-arabinoside-8-C-xyloside	Flavonoids	Flavones	2.46E+05	2.15E+05	1.60E+05	5.20E+05	3.79E+05	4.74E+05	6.87E+05	4.92E+05	4.35E+05	5.25E+05	2.80E+05	4.33E+05
Hmjp005906	C19H21NO5	N-Trans-Feruloyl-3'-O-methyldopamine	Alkaloids	Phenolamine	0.00E+00	0.00E+00	0.00E+00	1.09E+05	1.40E+05	1.53E+05	1.40E+05	1.42E+05	1.59E+05	1.29E+05	1.36E+05	1.49E+05
Zjhp090614	C17H16O6	5,7-dihydroxy-6-methyl-3-(2',4'-dihydroxybenzyl)-chroman-4-one	Flavonoids	Other Flavonoids	0.00E+00	0.00E+00	0.00E+00	1.01E+05	2.68E+06	7.52E+05	9.43E+04	2.99E+06	7.79E+05	7.86E+04	2.98E+06	6.65E+05
Zjhp090622	C19H20O6	(3R)-5,7-dihydroxy-6,8-dimethyl-3-(2'-hydroxy-4'-methoxybenzyl)-chroman-4-one*	Flavonoids	Other Flavonoids	0.00E+00	0.00E+00	0.00E+00	4.07E+05	4.26E+05	4.28E+05	4.04E+05	3.78E+05	5.69E+05	3.73E+05	3.76E+05	4.62E+05
pmp001309	C21H20O12	6-Hydroxykaempferol-7-O-glucoside*	Flavonoids	Flavonols	4.45E+04	4.97E+04	5.79E+04	1.90E+05	7.39E+05	3.42E+05	2.09E+05	8.26E+05	4.55E+05	2.11E+05	1.02E+06	3.27E+05
Zmcn006960	C54H88O23	Aralia saponin XVI*	Terpenoids	Triterpene Saponin	2.90E+04	2.70E+04	2.06E+04	1.11E+05	1.33E+05	1.46E+05	1.18E+05	1.43E+05	1.77E+05	1.38E+05	1.75E+05	1.71E+05
Zmcp102201	C12H12O3	Senkyunolide B	Others	Lactones	3.03E+07	3.86E+07	3.56E+07	2.85E+07	3.65E+07	2.87E+07	3.78E+07	2.92E+07	2.58E+07	2.68E+07	3.26E+07	3.10E+07
MWSHC20189	C20H22O6	Epipinoresinol*	ignans and Coumarir	Lignans	9.51E+04	8.52E+04	7.57E+04	1.98E+05	2.66E+05	2.87E+05	2.24E+05	2.63E+05	2.36E+05	1.73E+05	3.11E+05	2.38E+05
Smhp004476	C26H28O14	Isovitexin-2''-O-xyloside*	Flavonoids	Flavones	6.51E+04	9.92E+04	4.58E+04	2.48E+05	2.56E+05	2.20E+05	2.28E+05	2.13E+05	2.53E+05	1.79E+05	2.28E+05	2.19E+05
pmp000127	C28H32O16	Chrysoeriol-6,8-di-C-glucoside	Flavonoids	Flavones	1.07E+05	1.05E+05	1.48E+05	4.25E+05	4.48E+05	5.52E+05	5.53E+05	3.56E+05	4.54E+05	4.57E+05	4.12E+05	5.26E+05
Lazn002030	C23H30O11	Guaiacylglycerol-β-Guaiacyl Ether glucoside	ignans and Coumarir	Lignans	5.58E+04	2.77E+04	4.90E+04	7.49E+04	5.57E+04	5.60E+04	5.28E+04	5.83E+04	4.97E+04	5.57E+04	5.06E+04	5.21E+04
zjbp110819	C19H18O4	6,7-dimethoxy-2-[2-phenylethyl]chromone	Others	Chromone	4.43E+04	5.68E+04	1.08E+05	3.99E+05	4.42E+05	2.40E+05	3.92E+05	5.37E+05	2.47E+05	3.14E+05	3.44E+05	4.46E+05
Hahn002372	C9H10O3	apocynin	Others	Others	2.04E+05	1.31E+05	1.88E+05	9.55E+04	2.23E+05	1.26E+05	1.28E+05	1.99E+05	1.29E+05	1.34E+05	2.24E+05	1.23E+05
Zjhp082693	C51H80O24	Spirost-5-en-12-one-3-O-glucosyl(1→2)[glucosyl(1→3)]glucosyl(1→4)galactoside	Steroids	Steroidal saponins	8.28E+05	9.40E+05	7.26E+05	3.09E+06	3.41E+06	5.05E+06	3.10E+06	2.35E+06	4.52E+06	1.49E+06	2.45E+06	3.64E+06
Lmmp002463	C28H32O16	Sexangularetin-3-O-glucoside-7-O-rhamnoside	Flavonoids	Flavonols	9.98E+03	6.11E+03	1.62E+04	1.20E+05	1.68E+05	9.87E+04	8.62E+04	1.66E+05	1.40E+05	6.11E+04	6.05E+04	1.54E+05
Wmzn000627	C22H36O4	Ajugalaevigatic acid	Terpenoids	Ditepenoids	1.69E+05	1.32E+05	1.71E+05	1.69E+06	1.82E+06	1.68E+06	1.86E+06	2.43E+06	2.48E+06	2.22E+06	2.70E+06	2.30E+06
Saln000499	C31H40O17	7-O-(4''-O-glucosyl)coumaroyl-loganic acid	Terpenoids	Monoterpenoids	7.15E+05	7.83E+05	6.26E+05	1.23E+06	1.10E+06	1.07E+06	7.37E+05	9.96E+05	7.41E+05	8.55E+05	1.00E+06	8.92E+05
Lmdn003756	C10H10O4	Methyl caffeate	Phenolic acids	Phenolic acids	4.59E+04	6.63E+04	1.15E+04	1.01E+05	1.37E+05	1.39E+05	1.17E+05	6.42E+04	8.56E+04	7.22E+04	1.71E+05	8.68E+04
Lchp000218	C11H12O4	(R)-3-ethyl-7-hydroxy-6-methoxyphthalide	Others	Others	1.53E+05	1.48E+05	1.08E+05	1.55E+05	1.48E+05	4.23E+04	9.13E+04	1.52E+05	1.72E+05	1.19E+05	1.34E+05	1.45E+05
Lmbn004790	C11H14O4	Methyl 3-(3-hydroxy-4-methoxyphenyl)propanoate	Phenolic acids	Phenolic acids	8.84E+04	7.99E+04	5.67E+04	5.29E+04	5.61E+04	4.54E+04	4.04E+04	4.03E+04	3.23E+04	4.07E+04	1.63E+04	3.06E+04
Wcgp003250	C10H10O2	2-Methyl-3-hydroxyindan-1-one	Others	Others	3.21E+05	1.55E+05	2.91E+05	2.36E+05	3.96E+05	4.08E+05	1.90E+05	3.79E+05	3.62E+05	2.66E+05	4.19E+05	2.53E+05
mws0180	C7H6O4	2,5-Dihydroxybenzoic acid; Gentisic Acid*	Phenolic acids	Phenolic acids	3.10E+05	2.90E+05	2.59E+05	3.77E+05	4.35E+05	3.84E+05	3.18E+05	4.64E+05	3.92E+05	4.27E+05	4.49E+05	4.56E+05
pmb2654	C19H27NO12	Anthranilate-1-O-Sophoroside	Phenolic acids	Phenolic acids	9.58E+05	9.27E+05	7.98E+05	8.44E+05	6.83E+05	9.04E+05	7.53E+05	1.11E+06	1.18E+06	1.14E+06	9.20E+05	8.15E+05
pmb0500	C23H26N2O5	N-p-Coumaroyl-N'-feruloylputrescine	Alkaloids	Phenolamine	3.62E+04	5.92E+04	6.72E+04	2.01E+05	1.53E+05	3.43E+05	2.36E+05	1.19E+05	2.78E+05	2.04E+05	1.85E+05	1.73E+05
mws2103	C14H12O4	Piceatannol	Others	Stilbene	4.02E+04	4.50E+04	6.14E+04	6.96E+04	4.92E+04	6.86E+04	8.59E+04	5.08E+04	6.02E+04	4.69E+04	4.70E+04	6.51E+04
MWS20182	C10H12O4	Dihydroferulic Acid	Phenolic acids	Phenolic acids	1.22E+05	8.78E+04	1.20E+05	1.19E+05	1.43E+05	1.07E+05	7.83E+04	8.93E+04	9.64E+04	1.31E+05	1.32E+05	7.02E+04
Lmdp003286	C21H20O12	Isohyperoside*	Flavonoids	Flavonols	2.04E+05	1.74E+05	1.53E+05	2.28E+05	2.76E+05	3.80E+05	3.95E+05	4.41E+05	4.21E+05	2.03E+05	5.58E+05	3.36E+05
Lmdn003662	C19H34O8	Rehmaionoside A	Terpenoids	Monoterpenoids	6.90E+04	5.00E+04	4.65E+04	7.57E+04	1.16E+05	6.81E+04	7.10E+04	9.56E+04	8.37E+04	6.45E+04	7.52E+04	8.54E+04
MWSmce249	C7H5NO5	Chelidamic acid	Alkaloids	Pyridine alkaloids	4.48E+05	4.09E+05	4.30E+05	2.70E+06	5.63E+06	3.87E+06	2.67E+06	5.32E+06	3.94E+06	3.04E+06	5.60E+06	3.49E+06
Lcgn000239	C26H34O11	dihydrodehydrodiconiferyl alcohol-9-O-β-D-glucopyranoside	ignans and Coumarir	Lignans	1.79E+05	1.06E+05	1.27E+05	3.94E+05	2.63E+05	3.03E+05	3.84E+05	2.92E+05	2.98E+05	3.31E+05	2.39E+05	2.72E+05
Lmzn005381	C53H86O22	Jujuboside I	Terpenoids	Triterpene Saponin	0.00E+00	0.00E+00	0.00E+00	1.22E+04	9.45E+03	7.63E+03	1.33E+04	7.35E+03	1.33E+04	1.47E+04	6.00E+03	1.34E+04
Smgp004575	C21H20O12	Quercetin-5-O-β-D-glucoside*	Flavonoids	Flavonols	2.39E+05	1.62E+05	1.37E+05	1.47E+05	5.05E+05	3.80E+05	2.66E+05	5.61E+05	4.33E+05	1.45E+05	5.53E+05	3.41E+05
MWSslk135	C10H8O4	7,8-Dihydroxy-4-methylcoumarin	ignans and Coumarir	Coumarins	2.75E+05	2.72E+05	2.33E+05	3.35E+05	2.09E+05	1.93E+05	2.21E+05	1.97E+05	1.64E+05	2.37E+05	1.63E+05	1.12E+05
Zmjp004852	C23H24O13	5,6,3',4'-Tetrahydroxy-3,7-dimethoxyflavone-6-O-glucoside	Flavonoids	Flavones	5.73E+04	2.83E+04	1.60E+04	1.25E+05	1.02E+05	1.30E+05	2.39E+05	9.28E+04	2.58E+05	1.93E+05	2.00E+05	3.00E+05
Yajn003986	C21H34O10	Dihydropenstemide	Terpenoids	Monoterpenoids	1.35E+05	1.10E+05	1.15E+05	1.57E+05	1.61E+05	1.34E+05	1.23E+05	1.68E+05	1.17E+05	1.17E+05	1.77E+05	1.49E+05
WaYn005387	C22H24O10	Populoside	Phenolic acids	Phenolic acids	2.30E+03	3.60E+03	1.44E+03	1.34E+04	1.32E+05	7.72E+04	1.55E+04	1.16E+05	6.14E+04	1.28E+04	1.21E+05	7.36E+04
mws0983	C20H39NO2	N-Oleoylethanolamine	Alkaloids	Alkaloids	0.00E+00	0.00E+00	0.00E+00	8.40E+04	1.11E+05	8.87E+04	1.11E+05	1.34E+05	1.00E+05	8.94E+04	1.28E+05	8.83E+04
Wbwp010459	C20H22O5	5-[(2r,3s,4s,5s)-5-(2h-1,3-benzodioxol-5-yl)-3,4-dimethyloxolan-2-yl]-2-methoxyphenol	ignans and Coumarir	Lignans	7.85E+05	8.61E+05	1.12E+06	9.11E+05	1.05E+06	8.22E+05	9.53E+05	1.11E+06	1.18E+06	8.22E+05	8.84E+05	9.84E+05
pme1598	C22H24O11	Hesperetin-5-O-glucoside	Flavonoids	Flavanones	9.75E+03	4.39E+04	2.59E+04	3.69E+04	1.48E+05	9.34E+04	1.88E+04	1.46E+05	9.89E+04	1.26E+04	1.23E+05	1.17E+05
pmp000241	C27H28O17	Luteolin-6-C-(2''-glucuronyl)glucoside	Flavonoids	Flavones	6.81E+04	1.25E+04	3.62E+04	1.25E+05	1.58E+05	1.12E+05	2.00E+05	1.55E+05	1.01E+05	1.09E+05	8.97E+04	1.67E+05
MWStz208	C18H19NO5	N-Feruloyloctopamine*	Alkaloids	Phenolamine	4.30E+04	7.38E+04	5.14E+04	2.77E+05	4.39E+05	1.14E+06	5.99E+05	3.88E+05	7.10E+05	5.62E+05	4.83E+05	4.21E+05
pmp000607	C22H22O12	1-Demethyl orange blunt-leaf casein-2-O-β-D-glucoside	Quinones	Anthraquinone	3.01E+04	8.63E+04	5.49E+04	1.71E+05	2.03E+05	2.74E+05	3.42E+05	2.92E+05	1.97E+05	2.55E+05	1.71E+05	2.60E+05
Lazn003560	C22H30O11	Methoxy-erythro-Guaiacyl glycerol β-threo-syringyl glycerol ether	ignans and Coumarir	Lignans	2.12E+04	2.28E+04	3.00E+04	2.92E+04	3.07E+04	3.67E+04	2.11E+04	5.24E+04	3.73E+04	2.33E+04	3.00E+04	2.53E+04
Lmnp102580	C26H28O14	Apigenin-6-C-(2''-xylosyl)glucoside*	Flavonoids	Flavones	2.18E+04	4.49E+04	1.61E+04	2.07E+05	1.60E+05	1.42E+05	1.95E+05	1.45E+05	1.09E+05	1.37E+05	1.38E+05	1.93E+05
Lmjp003655	C22H22O11	6-C-MethylKaempferol-3-glucoside*	Flavonoids	Flavones	4.92E+04	6.02E+04	4.15E+04	3.62E+05	3.43E+05	4.85E+05	4.65E+05	3.46E+05	3.37E+05	6.18E+05	3.80E+05	5.04E+05
Zjhp082647	C45H70O19	Spirost-5-en-12-one-3-O-glucosyl(1→2)glucosyl(1→4)galactoside (Pratioside D1)	Steroids	Steroidal saponins	2.00E+06	1.12E+06	6.86E+05	3.07E+06	4.89E+06	5.40E+06	3.85E+06	2.70E+06	3.37E+06	2.50E+06	4.24E+06	5.10E+06
MWSmce283	C9H10O2	4'-Hydroxypropiophenone	Others	Ketone compounds	3.74E+04	4.43E+04	3.54E+04	5.42E+04	8.59E+04	4.92E+04	5.88E+04	5.94E+04	6.73E+04	6.39E+04	7.05E+04	8.47E+04
Cmrn003275	C16H28O8	Kankanoside E	Terpenoids	Monoterpenoids	6.99E+04	5.49E+04	6.49E+04	8.06E+04	8.12E+04	7.81E+04	5.30E+04	7.82E+04	8.48E+04	5.87E+04	8.70E+04	7.65E+04
MWSHY0149	C15H10O3	5-Hydroxyflavone	Flavonoids	Flavones	2.19E+05	1.87E+05	1.18E+05	8.28E+04	1.52E+05	2.47E+05	8.37E+04	9.51E+04	8.06E+04	1.22E+05	5.15E+04	4.55E+04
MWS1888	C12H16O6	Phenyl-beta-D-glucoside	Phenolic acids	Phenolic acids	0.00E+00	0.00E+00	0.00E+00	7.43E+04	5.30E+04	6.16E+04	9.94E+04	1.06E+05	9.61E+04	1.03E+05	7.33E+04	1.11E+05
Hahp004959	C29H34O17	Sesuvioside C	Flavonoids	Isoflavones	7.72E+03	3.51E+04	1.47E+04	5.81E+05	3.23E+05	3.73E+05	5.98E+05	2.99E+05	5.28E+05	5.42E+05	3.53E+05	4.99E+05
WaYn004638	C21H28O10	Grandidentoside	Phenolic acids	Phenolic acids	7.15E+05	7.02E+05	7.40E+05	9.49E+05	1.11E+06	5.58E+05	1.44E+06	8.55E+05	6.77E+05	1.31E+06	8.10E+05	6.33E+05
Lmbp010382	C18H16O3	6-Methoxy-2-(2-phenylethyl)chromone	Others	Chromone	6.31E+03	1.28E+04	1.62E+04	6.31E+04	4.83E+04	7.14E+04	7.15E+04	7.05E+04	6.01E+04	6.53E+04	4.94E+04	9.19E+04
Wdmp007253	C21H35NO	1-(1-aminoethyl)-9a,11a-dimethyl-tetradecahydrocyclopenta[a]phenanthren-7-one	Alkaloids	Plumerane	6.60E+05	4.96E+05	5.39E+05	3.91E+05	5.73E+05	3.23E+05	5.11E+05	4.63E+05	3.91E+05	4.32E+05	3.76E+05	3.71E+05
Lafp004228	C19H30O7	(6R,9R)-3-Oxo-α-ionol-β-D-glucoside	Terpenoids	Sesquiterpenoids	3.08E+04	1.76E+04	2.99E+04	5.83E+05	2.18E+05	1.51E+05	1.95E+05	2.20E+05	1.93E+05	2.09E+05	1.79E+05	3.69E+05
Lahp005848	C10H10O4	pithecellobiumol B	Others	Chromone	2.79E+06	3.03E+06	2.61E+06	3.23E+06	3.01E+06	2.29E+06	3.03E+06	2.86E+06	3.00E+06	2.66E+06	2.32E+06	2.34E+06
Lmsp004301	C21H22O11	3',5,5',7-Tetrahydroxyflavanone-7-O-glucoside	Flavonoids	Flavanones	1.49E+05	2.21E+05	1.45E+05	2.96E+05	2.45E+05	2.63E+05	2.46E+05	1.85E+05	2.31E+05	3.93E+05	3.38E+05	2.96E+05
Wbjp006749	C54H53N3O12	N-(4-Hydroxyphenethyl)-2-(3-methoxy-4-hydroxyphenyl)-5-[(Z)-2-[2-methoxy-4-[(E)-2-[(4-hydroxyphenethyl)carbamoyl]ethenyl]phenoxy]-2-[(4-hydroxyphenethyl)carbamoyl]ethenyl]-7-methoxy-2,3-dihydrobenzofuran-3-	Alkaloids	Alkaloids	0.00E+00	0.00E+00	0.00E+00	2.29E+05	3.46E+05	2.67E+05	1.76E+05	3.05E+05	2.49E+05	1.99E+05	3.54E+05	2.39E+05
Lmmn000774	C15H20O9	Dihydrocaffeoylglucos	Phenolic acids	Phenolic acids	1.01E+05	1.12E+05	1.01E+05	1.33E+05	2.07E+05	1.57E+05	1.83E+05	1.57E+05	2.52E+05	1.81E+05	1.66E+05	1.58E+05
Zjxp101205	C39H62O14	Spirost-25(27)ene-2,3-diol-3-O-glucosyl(1→2)galactosi	Steroids	Steroidal saponins	1.49E+06	1.14E+06	2.34E+06	4.73E+06	2.47E+06	5.37E+06	5.65E+06	3.50E+06	4.18E+06	7.91E+06	6.43E+06	3.95E+06
pmp001233	C12H14O2	Butylphthalide	Others	Lactones	1.64E+05	1.88E+05	1.26E+05	2.92E+05	2.98E+05	1.92E+05	2.83E+05	1.51E+05	1.83E+05	2.50E+05	1.68E+05	3.25E+05
Lmpp003958	C32H31O14+	Malvidin-3-O-(6''-O-p-coumaroyl)glucoside	Flavonoids	Anthocyanidins	8.29E+04	8.11E+04	5.25E+04	7.86E+04	6.91E+04	6.69E+04	1.40E+05	1.11E+05	9.72E+04	7.38E+04	8.18E+04	6.98E+04
MWSmce602	C27H43NO3	Peiminine	Alkaloids	Steroid alkaloids	4.24E+06	3.19E+06	3.23E+06	1.37E+07	1.24E+07	1.48E+07	1.48E+07	1.10E+07	1.31E+07	1.41E+07	1.53E+07	9.85E+06
Cmyp002064	C22H22O11	Yuanhuanin	Flavonoids	Flavones	2.97E+04	8.12E+04	2.35E+04	2.28E+05	3.83E+05	4.99E+05	3.72E+05	3.12E+05	4.62E+05	4.81E+05	3.33E+05	5.09E+05
Wagp001908	C20H30N2O8	N-Feruloylputrescine N-glucoside	Alkaloids	Phenolamine	6.91E+04	1.05E+05	1.98E+04	1.01E+05	5.68E+04	8.12E+04	6.66E+04	9.86E+04	1.17E+05	9.09E+04	1.36E+05	1.10E+05
Zmgp004436	C23H24O13	Okanin-4'-(6''-O-acetyl)glucoside	Flavonoids	Chalcones	8.88E+04	1.08E+05	4.40E+04	3.65E+05	3.53E+05	2.96E+05	3.37E+05	3.76E+05	3.06E+05	3.86E+05	2.76E+05	3.33E+05
Lalp005356	C17H16O5	(2R)-4',7-dihydroxy-5-methoxy-8-methylflavanone	Flavonoids	Flavanones	3.56E+04	3.53E+04	3.58E+04	1.06E+05	1.70E+05	1.26E+05	1.12E+05	1.69E+05	8.35E+04	1.04E+05	1.83E+05	1.29E+05
Qadp004838	C50H80O22	Aspidistrin	Steroids	Steroidal saponins	1.78E+03	1.19E+04	1.75E+03	3.53E+04	9.51E+03	1.46E+04	2.05E+04	8.81E+03	3.51E+04	2.86E+04	6.07E+04	2.72E+04
MWS1848	C8H8O2	Phenyl acetate	Phenolic acids	Phenolic acids	4.63E+04	2.47E+04	2.32E+04	4.26E+04	3.79E+04	3.13E+04	4.41E+04	3.09E+04	2.39E+04	9.98E+03	4.85E+04	2.91E+04
Lmmp010562	C24H38O4	Diisooctyl Phthalate*	Phenolic acids	Phenolic acids	7.87E+05	3.73E+05	2.27E+05	3.14E+05	1.38E+06	1.08E+06	7.27E+05	5.85E+05	6.47E+05	7.80E+05	9.26E+05	1.57E+06
Zbsn012113	C21H32O6	Diacetoxy-[6]-gingerdiol	Phenolic acids	Phenolic acids	0.00E+00	0.00E+00	0.00E+00	2.34E+04	2.86E+04	2.54E+04	2.43E+04	3.33E+04	2.85E+04	2.89E+04	2.53E+04	2.55E+04
mws0047	C27H30O14	Apigenin-7-O-neohesperidoside (Rhoifolin)	Flavonoids	Flavones	7.03E+04	1.72E+05	1.39E+05	3.55E+05	4.06E+05	4.03E+05	3.66E+05	4.39E+05	3.78E+05	3.51E+05	3.86E+05	4.02E+05
Zmpp000906	C8H11NO3	Norepinephrine	Alkaloids	Alkaloids	6.85E+04	9.84E+04	7.89E+04	1.29E+05	1.26E+05	8.18E+04	1.81E+05	1.37E+05	2.50E+05	1.96E+05	2.55E+05	2.27E+05
Lhyp112511	C28H38O13	Rumphioside B	Terpenoids	Ditepenoids	1.38E+05	1.53E+05	1.69E+05	2.80E+05	2.49E+05	1.34E+05	1.55E+05	2.29E+05	2.48E+05	1.05E+05	2.41E+05	3.06E+05
Ybnn006457	C16H14O6	Artocarpanone	Flavonoids	Flavanones	0.00E+00	0.00E+00	0.00E+00	3.61E+04	1.39E+05	7.18E+04	9.75E+03	1.82E+05	6.58E+04	3.08E+04	2.06E+05	5.42E+04
pme2960	C15H12O5	Naringenin chalcone; 2',4,4',6'-Tetrahydroxychalcone	Flavonoids	Chalcones	0.00E+00	0.00E+00	0.00E+00	4.97E+04	2.94E+05	1.42E+05	5.10E+04	2.67E+05	1.63E+05	5.05E+04	2.00E+05	1.21E+05
mws0005	C10H12N2	Tryptamine	Alkaloids	Plumerane	6.86E+04	1.30E+05	9.91E+04	1.31E+05	2.11E+05	1.52E+05	1.24E+05	2.00E+05	1.71E+05	1.58E+05	1.47E+05	1.30E+05
Zjmp102023	C19H18O5	iso-methyl-ophiopogonone B	Flavonoids	Other Flavonoids	0.00E+00	0.00E+00	0.00E+00	2.94E+04	4.45E+04	3.09E+04	3.20E+04	5.01E+04	2.92E+04	3.34E+04	4.92E+04	2.60E+04
Qatp010618	C20H22O5	Lignans Machilin F	ignans and Coumarir	Lignans	6.38E+05	9.84E+05	6.37E+05	7.55E+05	1.27E+06	5.69E+05	7.50E+05	6.51E+05	5.81E+05	7.42E+05	8.83E+05	5.64E+05
Zjbn102528	C16H14O3	7-hydroxy-2,4-dimethoxyphenanthren	Others	Others	1.07E+04	8.92E+03	9.31E+03	9.98E+03	9.30E+03	8.06E+03	1.06E+04	9.82E+03	1.32E+04	1.18E+04	8.64E+03	1.28E+04
mws2368	C8H10O2	Tyrosol; 4-Hydroxyphenylethanol	Phenolic acids	Phenolic acids	9.11E+04	8.35E+04	4.16E+04	5.78E+04	7.24E+04	7.42E+04	8.81E+04	6.55E+04	6.06E+04	9.52E+04	9.57E+04	8.30E+04
HJAP026	C28H32O16	Chrysoeriol-6-C-glucoside-4'-O-glucoside	Flavonoids	Flavones	2.82E+05	3.45E+05	3.29E+05	6.57E+05	6.85E+05	7.47E+05	8.29E+05	8.12E+05	5.84E+05	8.56E+05	5.39E+05	6.98E+05
Lamn006359	C22H24O10	4'-demethyl-3,9-dihydroeucomin glucoside	Flavonoids	Other Flavonoids	0.00E+00	0.00E+00	0.00E+00	1.92E+04	2.08E+05	1.38E+05	2.19E+04	2.08E+05	1.52E+05	2.12E+04	2.85E+05	1.28E+05
Wbjp005343	C33H48O9	(25R)-Spirost-4-ene-3,12-dione-rhamnose	Alkaloids	Alkaloids	1.90E+05	2.26E+05	1.97E+05	9.63E+05	7.37E+05	7.87E+05	7.83E+05	7.64E+05	1.02E+06	8.27E+05	7.51E+05	9.50E+05
MWSHC2041	C27H30O14	sophorabioside	Flavonoids	Isoflavones	3.43E+04	3.44E+04	4.98E+04	1.25E+05	1.25E+05	1.54E+05	1.56E+05	1.74E+05	2.90E+05	1.17E+05	1.15E+05	1.23E+05
pmb0603	C28H32O16	Chrysoeriol-7,4'-di-O-glucoside	Flavonoids	Flavones	1.18E+05	4.92E+04	5.70E+03	3.20E+05	4.00E+05	1.46E+05	1.98E+05	1.93E+05	2.06E+05	1.24E+05	2.09E+05	2.56E+05
mws0055	C20H20O7	Tangeretin (4',5,6,7,8-Pentamethoxyflavone)	Flavonoids	Flavones	0.00E+00	0.00E+00	0.00E+00	5.29E+04	4.12E+04	2.59E+04	3.72E+04	5.80E+04	4.48E+04	4.72E+04	4.38E+04	4.28E+04
Jmzn003632	C26H34O11	Manglieside D	ignans and Coumarir	Lignans	9.60E+04	7.19E+04	8.44E+04	8.49E+04	1.22E+05	9.60E+04	1.31E+05	7.73E+04	1.07E+05	1.17E+05	1.65E+05	1.71E+05
Hmlp004943	C28H32O16	Nitensoside B	ignans and Coumarir	Coumarins	8.90E+03	2.25E+03	5.91E+03	2.49E+04	6.55E+04	1.02E+05	8.38E+04	1.29E+05	1.09E+05	7.03E+04	1.24E+05	1.30E+05
Wdbn009958	C21H28O7	21-hydroperoxy-8-hydroxy-5,19-dimethyl-15,18,20-trioxapentacyclo[14.5.1.04,13.05,10.019,22]docosa-1,10-dien-14-one	Steroids	Steroidal saponins	0.00E+00	0.00E+00	0.00E+00	6.88E+04	7.38E+04	7.06E+04	4.93E+04	5.33E+04	1.29E+05	1.11E+05	6.74E+04	5.70E+04
Wadn003423	C18H18O9	Salvianic acid C	Phenolic acids	Phenolic acids	4.42E+04	5.28E+04	5.66E+04	6.90E+04	6.93E+04	6.92E+04	8.02E+04	8.76E+04	7.35E+04	1.02E+05	7.64E+04	6.74E+04
MWSHC2016	C22H18O11	Epigallocatechin-3-O-gallate	Flavonoids	Flavanols	6.45E+04	8.55E+04	5.99E+04	6.26E+04	5.14E+04	6.32E+04	4.10E+04	5.86E+04	6.09E+04	4.29E+04	5.87E+04	7.36E+04
Wafn003429	C16H30O10	3-Methylbutyl 6-O-(alpha-L-arabinopyranosyl)-beta-D-glucopyranoside	Others	Others	2.33E+05	4.51E+05	4.85E+05	8.93E+05	6.94E+05	6.73E+05	1.63E+06	1.69E+06	6.60E+05	9.41E+05	8.84E+05	7.49E+05
[illegible]	[illegible]	4-Hydroxy-7-[illegible]	[illegible]	[illegible]	[illegible]	[illegible]	[illegible]	[illegible]	[illegible]	[illegible]	[illegible]	[illegible]	[illegible]	[illegible]	[illegible]	[illegible]

pmb4777	C16H18O8	methoxycoumarin-p-rhamnoside	ignans and Coumarir	Coumarins	1.28E+04	1.00E+04	8.52E+03	4.63E+04	3.68E+04	3.95E+04	3.47E+04	3.21E+04	2.43E+04	3.88E+04	4.16E+04	2.31E+04
Qmzp101807	C20H22O5	Avicennol	ignans and Coumarir	Coumarins	6.15E+05	6.23E+05	6.44E+05	9.15E+05	1.02E+06	8.44E+05	9.80E+05	1.01E+06	7.57E+05	1.19E+06	1.13E+06	5.86E+05
HJN088	C18H24O12	Licoagroside B	Others	Ketone compounds	1.71E+05	1.20E+05	1.11E+05	1.61E+05	1.41E+05	1.51E+05	1.25E+05	1.62E+05	1.08E+05	1.67E+05	1.38E+05	1.59E+05
Zjmp102041	C19H20O6	5,7-dihydroxy-6,8-dimethyl-3-(4'-hydroxy-3'-methoxybenzyl)chroman-4-one*	Flavonoids	Other Flavonoids	0.00E+00	0.00E+00	0.00E+00	3.67E+05	4.07E+05	5.20E+05	4.02E+05	4.70E+05	5.65E+05	3.91E+05	4.19E+05	5.05E+05
pma2987	C6H11N3O	Histidinol	Alkaloids	Alkaloids	9.94E+06	9.66E+06	1.09E+07	1.19E+07	1.16E+07	1.14E+07	1.22E+07	1.12E+07	1.25E+07	1.19E+07	1.25E+07	1.07E+07
Lmmn003020	C22H28O8	Lyoniresinol	ignans and Coumarir	Lignans	2.73E+04	3.10E+04	3.28E+04	5.74E+04	4.13E+04	4.69E+04	4.61E+04	6.06E+04	4.15E+04	4.21E+04	4.00E+04	3.16E+04
Lmmp003091	C27H30O16	Quercetin-3-O-(4''-O-glucosyl)rhamnoside	Flavonoids	Flavonols	2.78E+04	1.46E+04	1.83E+04	4.71E+04	6.57E+04	1.08E+05	5.05E+04	3.54E+04	7.02E+04	6.21E+04	2.73E+04	3.21E+04
Lmpp003930	C30H26O12	Apigenin-7-O-(6''-p-Coumaryl)glucoside	Flavonoids	Flavones	4.80E+04	2.36E+04	2.22E+04	1.79E+05	8.39E+04	2.23E+05	1.73E+05	1.85E+05	1.26E+05	1.36E+05	1.68E+05	1.83E+05
MWSmce300	C7H13NO	Nortropine	Alkaloids	Tropan alkaloids	1.20E+05	1.21E+05	9.43E+04	1.13E+05	1.45E+05	1.67E+05	1.30E+05	1.29E+05	1.50E+05	1.41E+05	1.89E+05	1.15E+05
Lmzn001925	C12H16O5	3,4'-Dihydroxy-3'-methoxybenzenepentanoic acid	Phenolic acids	Phenolic acids	4.95E+04	4.36E+04	6.59E+04	7.18E+04	8.83E+04	5.98E+04	8.45E+04	7.63E+04	7.53E+04	8.09E+04	9.96E+04	5.13E+04
pmp001022	C27H45NO2	Tortifoline	Alkaloids	Steroid alkaloids	6.74E+05	1.34E+06	6.94E+05	6.11E+06	8.27E+06	8.19E+06	5.30E+06	4.15E+06	6.50E+06	5.22E+06	5.70E+06	7.82E+06
pme2598	C8H8O4	3,4-Dihydroxybenzeneacetic acid*	Phenolic acids	Phenolic acids	1.08E+05	8.46E+04	9.61E+04	1.68E+05	1.30E+05	2.09E+05	1.72E+05	1.78E+05	1.84E+05	1.15E+05	1.06E+05	1.22E+05
zjbp111502	C22H26O9	Ciwujiatone	ignans and Coumarir	Lignans	4.57E+04	9.52E+04	6.54E+04	7.88E+04	1.50E+05	1.65E+05	1.43E+05	1.60E+05	1.62E+05	1.34E+05	1.42E+05	1.48E+05
Lamp006064	C29H34O16	3-[(3,4-dihydroxyphenyl)methylidene]-5,7-dihydroxy-6-methoxy-2h-1-benzopyran-4-one glucosyl rhamnoside	Flavonoids	Other Flavonoids	1.15E+05	1.15E+05	9.97E+04	6.24E+04	1.13E+05	8.74E+04	8.76E+04	1.17E+05	1.01E+05	1.29E+05	1.02E+05	8.09E+04
MWSHY0064	C28H32O16	Isorhamnetin-3-O-neohesperidoside*	Flavonoids	Flavonols	0.00E+00	0.00E+00	0.00E+00	1.32E+05	6.33E+04	6.19E+04	1.18E+05	1.54E+05	1.21E+05	9.01E+04	1.22E+05	9.11E+04
Jmzn003343	C26H34O11	Saikolignanoside A	ignans and Coumarir	Lignans	9.20E+04	7.13E+04	8.37E+04	1.32E+05	1.47E+05	1.41E+05	1.17E+05	1.01E+05	1.14E+05	1.32E+05	1.86E+05	9.48E+04
MWS2070	C10H12O3	Propyl 4-hydroxybenzoate	Phenolic acids	Phenolic acids	2.89E+04	3.10E+04	3.10E+04	4.56E+04	4.54E+04	3.90E+04	3.83E+04	4.83E+04	4.65E+04	3.42E+04	4.63E+04	4.72E+04
HJAP011	C22H22O11	Chrysoeriol-8-C-glucoside (Scoparin)*	Flavonoids	Flavones	3.23E+04	2.20E+04	4.14E+04	5.07E+05	3.73E+05	2.65E+05	1.93E+05	2.68E+05	1.34E+05	1.37E+05	1.91E+05	2.43E+05
Wbhp008068	C20H32O5	(1R,4aS,4bR,7R,8R,8aR,9S,10aR)-7-ethenyl-8,8a,9-trihydroxy-1,4a,7-trimethyl-2,3,4,4b,5,6,8,9,10,10a-decahydrophenanthrene-1-carboxylic acid	Terpenoids	Terpene	4.01E+05	2.50E+05	3.63E+05	1.10E+06	1.70E+06	1.46E+06	1.66E+06	2.11E+06	1.81E+06	1.63E+06	1.39E+06	1.43E+06
Zjbp081711	C39H62O13	3-Hydroxyspirost-6-one-3-O-rhamnosyl-(1→2)-glucoside	Steroids	Steroidal saponins	2.27E+04	2.62E+04	8.42E+03	1.15E+05	1.81E+05	1.01E+05	1.57E+05	1.58E+05	2.00E+05	9.39E+04	1.09E+05	1.27E+05
Lmap001661	C17H24O9	Grevilloside C	Phenolic acids	Phenolic acids	6.11E+04	5.79E+04	4.16E+04	7.22E+04	9.24E+04	9.97E+04	1.01E+05	7.08E+04	6.98E+04	9.40E+04	1.11E+05	9.52E+04
pmp001265	C24H49NO8	2-Amino-4-dihydroxy octadecyl galactoside	Alkaloids	Alkaloids	0.00E+00	0.00E+00	0.00E+00	4.01E+05	3.12E+05	3.00E+05	3.93E+05	3.40E+05	4.42E+05	2.80E+05	3.81E+05	3.20E+05
Lmlp002205	C11H16O3	Isololiolide	Terpenoids	Terpene	4.87E+04	5.25E+04	8.26E+04	4.89E+04	9.15E+04	4.12E+04	6.14E+04	5.01E+04	7.66E+04	5.17E+04	4.00E+04	2.31E+04
Zmhp102730	C27H30O17	6-Hydroxykaempferol-3,6-O-Diglucoside*	Flavonoids	Flavonols	5.28E+04	2.18E+04	2.74E+04	9.14E+04	6.68E+04	5.73E+04	6.41E+04	8.38E+04	1.01E+05	9.54E+04	7.79E+04	1.04E+05
Sayp011213	C20H28O3	cleistanthol	Terpenoids	Ditepenoids	6.11E+04	4.68E+04	5.04E+04	1.81E+05	4.06E+05	3.98E+04	8.44E+04	1.15E+05	1.02E+05	4.83E+04	3.33E+04	2.78E+04
Zmcn006748	C54H88O23	Aralia saponin XIV*	Terpenoids	Triterpene Saponin	1.75E+04	1.27E+04	9.93E+03	9.92E+04	8.15E+04	7.69E+04	8.79E+04	6.71E+04	8.03E+04	1.14E+05	1.01E+05	1.01E+05
pmb0496	C15H22N4O3	N-Feruloylagmatine	Alkaloids	Phenolamine	6.01E+04	6.81E+04	8.47E+04	3.47E+05	3.66E+05	2.55E+05	2.51E+05	2.83E+05	2.38E+05	1.93E+05	3.29E+05	2.04E+05
MWS1852	C8H8O2	4-Methoxybenzaldehyde	Others	Aldehyde compounds	0.00E+00	0.00E+00	0.00E+00	2.24E+05	2.75E+05	2.29E+05	2.68E+05	2.55E+05	2.07E+05	2.12E+05	1.70E+05	1.73E+05
Qmjp110218	C18H19NO5	N-Feruloyl dopamine*	Alkaloids	Phenolamine	1.49E+04	9.74E+03	4.02E+03	7.64E+04	5.39E+04	4.87E+04	7.41E+04	9.51E+04	4.25E+04	3.32E+04	6.88E+04	4.72E+04
Hamp010390	C16H22O4	cytosporone C	Others	Others	6.64E+06	5.86E+06	6.94E+06	6.93E+06	6.45E+06	5.43E+06	6.36E+06	5.59E+06	5.05E+06	6.40E+06	6.72E+06	5.84E+06
Wdbp005281	C16H16O3	9,10-Dihydro-5-methoxy-8-methyl-2,7-phenanthrenediol	Others	Others	3.01E+05	4.49E+05	4.49E+05	2.65E+05	3.45E+05	4.20E+05	2.82E+05	3.11E+05	3.24E+05	3.31E+05	4.12E+05	3.61E+05
pmn001506	C36H58O8	Oleanolic acid-3-O-glucoside	Terpenoids	Triterpene Saponin	0.00E+00	0.00E+00	0.00E+00	1.85E+04	1.46E+04	2.16E+04	1.80E+04	2.08E+04	1.96E+04	1.83E+04	1.59E+04	1.92E+04
HJAP010	C25H26O14	Luteolin-6,8-di-C-arabinoside	Flavonoids	Flavones	4.91E+04	2.63E+04	3.49E+04	1.28E+05	8.23E+04	1.86E+05	1.64E+05	7.31E+04	8.01E+04	6.65E+04	1.06E+05	1.51E+05
Wbjp002330	C11H12N2O3	(3S)-1,3-dihydroxy-2,3,3a,4-tetrahydropyrrolo[2,1-b]quinazolin-9(1H)-one	Alkaloids	Alkaloids	3.30E+05	3.91E+05	2.79E+05	5.78E+05	5.20E+05	6.14E+05	5.12E+05	5.33E+05	3.99E+05	5.88E+05	5.22E+05	5.74E+05
pme0368	C27H30O14	Apigenin-7-O-rutinoside	Flavonoids	Flavones	5.22E+04	5.05E+04	1.08E+04	2.14E+05	2.17E+05	1.78E+05	9.65E+04	1.22E+05	2.20E+05	1.60E+05	1.85E+05	2.03E+05
mws0160	C9H10O4	3,4-Dihydroxybenzoic Acid Ethyl Ester (Protocatechuic acid ethyl ester)	Phenolic acids	Phenolic acids	1.58E+04	1.42E+04	1.18E+04	4.01E+04	5.46E+04	4.77E+04	6.03E+04	4.35E+04	5.06E+04	6.71E+04	4.59E+04	6.25E+04
pmb3000	C24H24O12	Chrysoeriol-7-O-(6''-acetyl)glucoside	Flavonoids	Flavones	1.57E+04	5.41E+03	3.85E+04	3.75E+04	4.33E+04	2.32E+04	6.04E+04	4.43E+04	3.24E+04	3.20E+04	3.04E+04	2.47E+04
Lhmp112929	C22H26N2O3	16-Methoxytabersonine	Alkaloids	Plumerane	1.13E+05	7.42E+04	9.86E+04	1.17E+05	1.15E+05	1.05E+05	9.27E+04	6.15E+04	1.07E+05	5.31E+04	1.28E+05	9.48E+04
pmp001020	C27H43NO2	Korseveridine	Alkaloids	Steroid alkaloids	1.16E+05	2.55E+04	1.03E+05	5.88E+05	4.37E+05	5.85E+05	4.47E+05	5.30E+05	6.46E+05	3.55E+05	5.28E+05	4.01E+05
mws0987	C10H8O2	6-MethylCoumarin	ignans and Coumarir	Coumarins	1.78E+05	2.08E+05	1.90E+05	1.49E+05	2.13E+05	1.65E+05	1.60E+05	1.44E+05	1.40E+05	1.10E+05	1.56E+05	1.81E+05
Lman002731	C15H18O9	Grevilloside F	Phenolic acids	Phenolic acids	4.01E+04	3.79E+04	4.54E+04	5.74E+04	1.01E+05	5.43E+04	7.63E+04	8.99E+04	7.47E+04	5.12E+04	1.03E+05	7.79E+04
Wbsp000191	C12H23N3O	3,7,11-trimethyl-2-oxa-6,10,13-triazatricyclo[7.3.1.05,13]tridecane	Alkaloids	Alkaloids	3.01E+05	2.48E+05	2.53E+05	2.93E+05	3.51E+05	2.00E+05	2.27E+05	1.73E+05	1.48E+05	1.52E+05	1.57E+05	1.67E+05
Lssp210093	C18H19NO5	7-hydroxy-N-trans-ferulic tyramine*	Alkaloids	Phenolamine	0.00E+00	0.00E+00	0.00E+00	3.15E+05	5.38E+05	3.20E+05	4.28E+05	2.88E+05	4.17E+05	5.17E+05	7.13E+05	1.80E+05
Hmcp001628	C29H34O18	Limocitrin-3,7-di-O-glucoside*	Flavonoids	Flavonols	4.23E+04	5.13E+04	1.52E+04	5.82E+04	6.51E+04	4.21E+04	1.01E+05	4.36E+04	2.15E+04	6.71E+04	2.06E+04	8.16E+04
zjgp122326	C12H15NO7	Pyridine-4-formyl-O-β-D-glucopyranoside	Others	Others	2.36E+05	1.90E+05	1.30E+05	4.04E+05	4.46E+05	4.15E+05	4.96E+05	4.20E+05	5.56E+05	8.58E+05	3.26E+05	2.90E+05
Zbfn004680	C28H34O16	2-(3,4-Dihydroxyphenethoxy)-6-(3,4,5-trihydroxycaffeoyl)-O-arabinopyranosyl-D-glucose	Phenolic acids	Phenolic acids	1.95E+04	7.81E+03	1.85E+04	8.57E+04	1.58E+05	1.63E+05	6.96E+04	1.46E+05	1.41E+05	3.74E+04	1.78E+05	1.37E+05
Sazp005659	C21H22O7	Comazaphilone D	Others	Others	5.84E+04	1.01E+05	9.65E+04	1.52E+05	1.79E+05	2.22E+05	1.19E+05	2.08E+05	1.65E+05	1.83E+05	1.01E+05	1.00E+05
Zjbn102547	C28H30O9	2,7-Dihydroxy-1-(p-hydroxybenzyl)-4-methoxy-9,10-dihydrophenanthrene-4'-O-glucose	Others	Others	1.40E+04	1.85E+04	1.10E+04	2.99E+04	5.27E+04	2.90E+04	3.62E+04	3.76E+04	3.54E+04	4.05E+04	4.50E+04	4.42E+04
Lmdn001501	C16H22O10	Gardoside	Terpenoids	Monoterpenoids	3.20E+04	2.07E+04	1.20E+04	3.65E+04	3.75E+04	3.67E+04	3.80E+04	3.20E+04	5.33E+04	4.80E+04	2.98E+04	4.17E+04
Lmsn010558	C35H62O6	Squamocin K	Others	Others	0.00E+00	0.00E+00	0.00E+00	8.88E+03	1.32E+04	1.13E+04	9.63E+03	2.53E+04	1.10E+04	1.22E+04	1.55E+04	1.33E+04
pmb3041	C23H22O14	Tricin-7-O-saccharic acid	Flavonoids	Flavones	3.77E+04	6.97E+04	1.05E+05	1.06E+05	1.04E+05	7.72E+04	7.23E+04	1.40E+05	1.24E+05	5.97E+04	1.28E+05	1.38E+05
Zbzp007397	C11H16O2	5,6,7,7a-tetrahydro-4,4,7a-trimethyl-2(4H)-benzofuranone	Others	Others	8.64E+04	1.06E+05	9.01E+04	2.93E+05	3.42E+05	3.54E+05	3.03E+05	2.18E+05	2.68E+05	2.18E+05	2.63E+05	2.67E+05
mws1434	C21H20O10	Apigenin-6-C-glucoside (Isovitexin)	Flavonoids	Flavones	5.85E+03	6.08E+03	6.01E+03	4.66E+04	4.05E+04	3.48E+04	4.38E+04	1.65E+04	4.49E+04	3.61E+04	3.51E+04	5.19E+04
Wbmn014253	C20H28O3	Hardwickiic acid	Terpenoids	Ditepenoids	6.80E+05	1.19E+06	9.23E+05	1.47E+06	1.21E+06	1.21E+06	1.22E+06	1.53E+06	1.20E+06	1.40E+06	1.03E+06	1.49E+06
Zmhp003322	C36H36O17	Isovitexin-2''-O-(6'''-p-coumaroyl)glucoside	Flavonoids	Flavones	2.47E+04	2.86E+04	1.55E+04	2.24E+04	8.88E+04	7.67E+04	5.76E+04	8.28E+04	6.31E+04	1.06E+05	8.59E+04	2.63E+04
HJAP155	C21H20O12	Laricitrin-3-O-xyloside	Flavonoids	Flavonols	1.02E+05	9.16E+04	7.80E+04	1.46E+05	1.16E+05	6.22E+04	8.76E+04	1.29E+05	4.75E+04	8.07E+04	8.07E+04	5.70E+04
Zbnp004766	C23H24O13	Limocitrin-7-O-glucoside*	Flavonoids	Flavonols	7.23E+04	4.46E+04	8.61E+04	5.19E+05	5.68E+05	3.09E+05	6.01E+05	2.29E+05	2.67E+05	5.37E+05	3.03E+05	4.94E+05
Zmxp004573	C10H10O4	3-methoxy-4,5-methylenedioxy-acetophenone	Phenolic acids	Phenolic acids	1.02E+04	1.23E+04	1.58E+04	1.48E+05	1.22E+05	1.15E+05	1.26E+05	8.91E+04	8.60E+04	9.98E+04	1.04E+05	7.38E+04
Yshs000041	C26H36O7	Vibsanin J	Terpenoids	Ditepenoids	0.00E+00	0.00E+00	0.00E+00	8.20E+05	4.83E+05	9.09E+05	8.31E+05	6.09E+05	6.13E+05	9.33E+05	7.20E+05	8.56E+05
pmn001370	C32H42O16	Pinoresinol-4,4'-O-diglucoside	ignans and Coumarir	Lignans	7.41E+04	3.96E+04	1.23E+05	6.89E+04	4.40E+04	2.09E+04	7.50E+04	3.65E+04	6.44E+04	7.92E+04	8.38E+04	7.73E+04
MWSprf094	C27H34O12	Tracheloside	ignans and Coumarir	Lignans	1.84E+04	1.34E+04	2.17E+04	2.49E+04	3.11E+04	3.91E+04	3.71E+04	1.62E+04	2.48E+04	2.70E+04	2.58E+04	1.60E+04
Lazn003996	C20H26O7	Erythro-Guaiacylglycerol-β-dihydroconiferyl Ether	ignans and Coumarir	Lignans	3.48E+04	2.82E+04	2.73E+04	3.50E+04	5.42E+04	4.09E+04	5.23E+04	4.42E+04	4.96E+04	3.44E+04	4.19E+04	4.52E+04
Latp009986	C16H22O4	6β-Hydroxy-8α-methoxyeremophila-1(10),7(11)-dien-12,8β-olide	Terpenoids	Sesquiterpenoids	3.37E+07	2.21E+07	2.30E+07	2.82E+07	2.61E+07	2.83E+07	2.88E+07	2.27E+07	3.98E+07	3.10E+07	2.35E+07	2.59E+07
mws1584	C25H24O12	1,3-O-Dicaffeoylquinic Acid (Cynarin)	Phenolic acids	Phenolic acids	3.86E+04	4.16E+04	4.21E+04	7.34E+04	6.82E+04	8.53E+04	6.85E+04	4.48E+04	9.10E+04	6.72E+04	5.05E+04	3.68E+04
Lmpp005784	C21H20O12	6-Methoxyquercetin-3-O-Xyloside	Flavonoids	Flavonols	5.59E+04	5.67E+04	1.03E+05	1.39E+05	1.53E+05	1.01E+05	9.20E+04	5.27E+04	8.90E+04	1.17E+05	1.08E+05	7.69E+04
Hahn000968	C32H36O15	picroroside B	Others	Others	1.37E+04	1.87E+04	1.72E+04	8.99E+04	7.80E+04	7.72E+04	7.61E+04	5.12E+04	7.80E+04	6.64E+04	9.23E+04	5.42E+04
Smnp005251	C15H12O4	furoaloesone	Others	Aldehyde compounds	1.69E+05	1.22E+05	1.57E+05	2.91E+05	3.86E+05	2.10E+05	2.87E+05	3.08E+05	2.19E+05	3.54E+05	2.49E+05	2.06E+05
Lmpp003929	C31H29O13+	Peonidin-3-O-(6''-O-p-coumaroyl)glucoside	Flavonoids	Anthocyanidins	1.01E+04	6.40E+03	5.60E+03	9.02E+04	9.96E+04	7.59E+04	1.23E+05	9.93E+04	8.83E+04	1.42E+05	1.13E+05	9.30E+04
Qmyp101334	C18H28O3	JiangxiBaiyingsu I	Terpenoids	Sesquiterpenoids	0.00E+00	0.00E+00	0.00E+00	7.82E+04	1.02E+05	1.04E+05	6.16E+04	9.63E+04	8.66E+04	5.84E+04	7.52E+04	7.84E+04
Sacn003282	C17H24O11	10-hydroxymajoroside	Phenolic acids	Phenolic acids	1.41E+05	1.11E+05	1.52E+05	2.13E+05	2.25E+05	2.17E+05	2.34E+05	2.81E+05	2.89E+05	1.86E+05	2.49E+05	2.47E+05
pmp000479	C16H29NO	N-Isobutyl-2E,4E-dodedienamide	Alkaloids	Alkaloids	4.86E+04	6.94E+04	5.66E+04	1.32E+05	1.54E+05	9.19E+04	1.22E+05	1.15E+05	1.37E+05	1.04E+05	1.00E+05	9.73E+04
Qmgp102027	C27H43NO3	Rubivirine	Alkaloids	Steroid alkaloids	3.77E+06	3.37E+06	3.25E+06	1.66E+07	1.41E+07	1.54E+07	1.40E+07	8.61E+06	1.14E+07	1.53E+07	1.23E+07	1.22E+07
Wbjp002336	C16H18N2O5	N,N-diethyl-5-hydroxytryptamineoxalate	Alkaloids	Alkaloids	6.72E+05	7.01E+05	6.52E+05	8.37E+05	1.58E+06	1.26E+06	1.28E+06	7.82E+05	9.69E+05	9.87E+05	1.29E+06	1.51E+06
Wbmn010845	C20H30O4	Pentandranoic acid C	Terpenoids	Ditepenoids	0.00E+00	0.00E+00	0.00E+00	8.46E+04	8.38E+04	7.71E+04	8.26E+04	9.35E+04	6.68E+04	7.25E+04	8.75E+04	6.80E+04
Wchn003447	C16H22O9	(S)-4-(3-Hydroxyphenyl)-4-(beta-D-glucopyranosyloxy)butyric acid	Others	Others	3.48E+04	3.25E+04	3.74E+04	1.91E+04	4.43E+04	2.65E+04	1.80E+04	5.02E+04	3.11E+04	4.03E+04	2.21E+04	3.02E+04
Zjhp082646	C45H72O18	Spirost-5-ene-3,27-diol-27-O-glucoside-3-O-[rhamnosyl(1→4)]glucoside (Polygonatoside D)	Steroids	Steroidal saponins	9.60E+04	9.23E+04	1.61E+05	3.70E+05	3.39E+05	3.26E+05	4.25E+05	4.96E+05	4.15E+05	5.04E+05	4.72E+05	6.44E+05
HX1372	C20H24O7	guaiacylglycerol-β-coniferyl ether	ignans and Coumarir	Lignans	1.17E+04	1.67E+04	1.22E+04	4.32E+04	1.75E+04	1.74E+04	2.20E+04	1.34E+04	3.56E+04	4.23E+04	1.59E+04	1.50E+04
Wmqn000820	C11H14O3	Dehydrololiolide	Terpenoids	Terpene	4.12E+04	5.75E+04	4.52E+04	8.16E+04	1.26E+05	1.04E+05	8.16E+04	1.12E+05	1.43E+05	1.22E+05	8.82E+04	8.33E+04
Zahn005590	C20H24O6	(7'S,8R,8'R)-3,5'-dimethoxy-3',4,9'-trihydroxy-7',9-epoxy-8,8'-lignan	ignans and Coumarir	Lignans	3.22E+04	1.96E+04	3.28E+04	6.79E+04	6.29E+04	7.76E+04	6.49E+04	5.87E+04	6.52E+04	3.97E+04	8.90E+04	4.43E+04
pmn001525	C23H26O11	3,5-Digalloylshikimic acid	Phenolic acids	Phenolic acids	1.53E+04	1.21E+04	1.92E+04	1.67E+04	1.98E+04	2.45E+04	1.89E+04	1.33E+04	2.29E+04	1.89E+04	1.66E+04	1.98E+04
Wafp003720	C24H29NO10	N-Feruloyloctopamine glucoside	Alkaloids	Phenolamine	3.90E+04	9.71E+04	3.75E+04	7.49E+04	8.02E+04	1.20E+05	1.19E+05	1.22E+05	9.37E+04	4.87E+04	6.36E+04	3.95E+04
MWSslk066	C8H8O4	3-Hydroxy-4-methoxybenzoic acid; Isovanillic Acid	Phenolic acids	Phenolic acids	2.70E+05	1.63E+05	1.22E+05	1.79E+05	1.69E+05	1.04E+05	1.27E+05	1.84E+05	1.04E+05	1.47E+05	1.25E+05	1.31E+05
Xmsn004568	C16H24O8	Mudanpioside F	Terpenoids	Monoterpenoids	4.70E+04	4.83E+04	5.48E+04	2.97E+04	1.15E+05	8.89E+04	4.60E+04	1.12E+05	6.25E+04	4.97E+04	6.61E+04	5.39E+04
Hmln001931	C32H42O16	Matairesinol-4,4'-O-diglucoside	ignans and Coumarir	Lignans	2.74E+04	1.53E+04	2.66E+04	7.23E+04	4.76E+04	5.26E+04	4.34E+04	3.69E+04	6.16E+04	4.24E+04	4.67E+04	2.62E+04
mws0566	C7H8O2	4-Methylcatechol	Phenolic acids	Phenolic acids	5.02E+04	2.70E+04	3.01E+04	3.20E+04	5.50E+04	2.99E+04	3.51E+04	3.74E+04	5.34E+04	5.20E+04	5.80E+04	3.21E+04
Hmgp001902	C23H24O11	Pectolinarigenin-7-O-glucoside	Flavonoids	Flavones	2.82E+05	2.86E+05	2.72E+05	5.07E+05	8.86E+05	8.83E+05	6.38E+05	9.16E+05	9.80E+05	5.37E+05	1.03E+06	7.94E+05
NK10264324	C6H6O3	Phloroglucinol; 1,3,5-Benzenetriol	Phenolic acids	Phenolic acids	3.43E+06	3.17E+06	2.93E+06	3.71E+06	4.23E+06	4.28E+06	5.15E+06	4.58E+06	5.01E+06	5.39E+06	4.66E+06	5.05E+06
pmp001287	C8H9N	N-Benzylmethylene isomethylamine	Alkaloids	Alkaloids	1.85E+06	1.94E+06	1.91E+06	2.74E+06	4.28E+06	3.64E+06	3.50E+06	4.60E+06	3.72E+06	3.66E+06	4.44E+06	3.81E+06
Zbjn005343	C28H32O15	Acacetin-7-O-(6''-O-glucoside)glucoside	Flavonoids	Flavones	1.14E+04	2.32E+04	1.10E+04	1.26E+05	7.10E+04	1.00E+05	1.62E+05	6.29E+04	6.99E+04	1.21E+05	7.04E+04	1.06E+05
Hagn003556	C23H16O6	7-Benzyloxy-5-hydroxy-3',4'-methylenedioxyflavonoid	Flavonoids	Flavones	6.31E+04	7.24E+04	7.26E+04	1.76E+05	1.21E+05	1.88E+05	1.12E+05	1.54E+05	1.71E+05	1.82E+05	1.41E+05	1.98E+05
pmp000608	C23H24O12	Aurantio-obtusin-6-O-Glucoside	Quinones	Anthraquinone	0.00E+00	0.00E+00	0.00E+00	2.56E+05	3.20E+05	3.46E+05	4.01E+05	3.44E+05	3.37E+05	2.94E+05	4.03E+05	2.55E+05
pmb2970	C28H34O16	Hesperetin-5,7-di-O-glucoside	Flavonoids	Flavanones	1.31E+05	9.07E+04	7.95E+04	5.54E+04	7.21E+04	1.10E+05	6.72E+04	1.04E+05	4.67E+04	7.57E+04	9.48E+04	7.85E+04
Hmjp002999	C28H32O16	Isorhamnetin-3-O-rutinoside (Narcissin)*	Flavonoids	Flavonols	0.00E+00	0.00E+00	0.00E+00	1.26E+05	1.16E+05	1.29E+05	1.14E+05	1.15E+05	9.52E+04	1.00E+05	7.74E+04	9.76E+04
pme2954	C15H10O7	Quercetin	Flavonoids	Flavonols	0.00E+00	0.00E+00	0.00E+00	6.31E+04	1.31E+05	4.40E+04	5.86E+04	1.81E+05	4.77E+04	6.26E+04	1.18E+05	4.40E+04
Zjhp083025	C57H90O29	Furost-5,20(22)-dien-12-one-3-O-glucosyl(1-2)[glucosyl(1-3)]glucosyl(1-4)galactoside-26-O-glucoside (Kingianoside Z)	Steroids	Steroidal saponins	7.58E+06	3.43E+06	1.67E+06	7.45E+06	1.64E+07	1.53E+07	1.26E+07	1.00E+07	9.95E+06	2.61E+07	5.06E+06	9.50E+06
Zmlp003063	C33H40O19	Luteolin-7-O-(2''-O-rhamnosyl)rutinoside*	Flavonoids	Flavones	4.43E+04	5.00E+04	5.18E+04	3.18E+04	5.21E+04	3.95E+04	3.68E+04	3.63E+04	6.11E+04	3.43E+04	2.05E+04	5.52E+04
pme1439	C9H8O3	p-Coumaric acid	Phenolic acids	Phenolic acids	5.35E+04	4.49E+04	9.51E+04	5.71E+04	8.87E+04	5.34E+04	4.39E+04	1.14E+05	5.76E+04	1.29E+05	5.02E+04	3.52E+04
Zjxp101225	C45H72O19	26-O-Glucosyl-furost-20(22)-25(27)-diene-3,12,26-triol-3-O-glucosyl(1→2)-galactoside	Steroids	Steroidal saponins	9.14E+04	9.31E+04	7.23E+04	1.30E+05	3.10E+05	2.75E+05	2.04E+05	2.30E+05	2.41E+05	7.67E+04	3.58E+05	1.31E+05
Lazn005606	C37H46O16	[(2S)-2α-[3,5-Dimethoxy-4-[[(αS,βR)-α-(Hydroxymethyl)-β,4-Dihydroxy-3-Methoxyphenethyl]Oxy]Phenyl]-5-(3-Hydroxy-1-Propenyl)-7-Methoxy-2,3-Dihydrobenzofuran]-3β-Ylmethylβ-D-Glucopyranoside	ignans and Coumarir	Lignans	1.59E+04	1.57E+04	3.48E+03	3.48E+04	5.60E+04	3.22E+04	3.44E+04	3.11E+04	4.29E+04	3.46E+04	5.43E+04	2.21E+04
Yajn004424	C17H28O10	2-((6-hydroxy-4-(hydroxy(methoxy)methyl)-7-methyl-1,4a,5,6,7,7a-hexahydrocyclopenta[c]pyran-1-yl)oxy)-6-(hydroxymethyl)tetrahydro-2H-pyran-3,4,5-triol	Terpenoids	Monoterpenoids	2.17E+04	1.46E+04	1.47E+04	7.18E+04	2.80E+04	4.89E+04	7.48E+04	5.35E+04	3.78E+04	4.78E+04	2.97E+04	5.72E+04
mws1329	C21H20O12	Quercetin-7-O-glucoside	Flavonoids	Flavonols	2.92E+04	9.63E+04	1.74E+04	2.47E+04	1.30E+05	9.72E+04	3.01E+04	1.85E+05	5.92E+04	3.15E+04	1.33E+05	7.80E+04
Wafp003258	C24H27NO9	N-Feruloylphenylacetamide glucoside	Alkaloids	Phenolamine	6.90E+04	7.43E+04	5.76E+04	1.42E+05	9.85E+04	1.24E+05	1.02E+05	1.05E+05	1.44E+05	1.19E+05	1.34E+05	2.09E+05
Zmcn003729	C26H32O12	1-Hydroxypinoresinol-4'-O-Glucoside	ignans and Coumarir	Lignans	6.28E+04	3.10E+04	4.42E+04	3.95E+04	5.02E+04	6.36E+04	6.42E+04	8.50E+04	8.44E+04	1.75E+04	7.11E+04	4.04E+04
pmp000878	C22H22O10	Swertisin	Flavonoids	Flavones	1.19E+05	2.59E+04	4.72E+04	2.47E+05	1.15E+05	3.14E+05	2.12E+05	2.02E+05	1.96E+05	2.88E+05	1.96E+05	2.94E+05
Lssp210056	C21H20O12	Quercetin3-O-galactoside*	Flavonoids	Other Flavonoids	3.82E+04	8.87E+04	4.39E+04	1.47E+05	2.41E+05	1.09E+05	1.39E+05	1.82E+05	2.03E+05	1.20E+05	2.65E+05	1.94E+05
Lmfn002465	C22H28O13	1,2,4-Trihydroxynaphthalene-1,4-di-glucoside (Lawsoniaside)	Others	Others	2.85E+05	1.93E+05	1.83E+05	1.59E+05	2.39E+05	2.41E+05	2.85E+05	3.69E+05	3.00E+05	2.55E+05	1.90E+05	3.57E+05
Lmdp003994	C23H24O10	6,4'-Dimethoxyisoflavone-7-O-glucoside (Wistin)	Flavonoids	Isoflavones	1.61E+04	1.99E+04	1.32E+04	7.29E+04	6.31E+04	1.70E+05	1.20E+05	1.31E+05	1.51E+05	1.13E+05	1.06E+05	1.03E+05
Qabp004038	C57H90O29	3-Epineoruscogenin-glucose-glucose-glucose-glucose-glucoside	Steroids	Steroidal saponins	1.11E+07	2.55E+06	2.71E+06	7.10E+06	1.47E+07	2.38E+07	9.32E+06	6.33E+06	9.38E+06	1.29E+07	2.45E+07	1.71E+07
Zjhp082685	C51H80O23	3-Hydroxy-cholest-5,14,16-trien-22-one-3-O-glucosyl(1→2)glucosyl(1→4)galactoside-26-O-glucoside (Polygonatumoside C)	Steroids	Steroidal saponins	2.95E+04	1.46E+04	2.28E+04	1.31E+05	8.70E+04	1.50E+05	1.04E+05	1.39E+05	1.31E+05	1.56E+05	2.04E+05	1.52E+05
Zjhp082618	C33H52O9	Isochiapagenin-Glc	Steroids	Steroidal saponins	1.90E+05	6.68E+04	1.55E+05	2.28E+05	2.43E+05	8.72E+05	4.11E+05	2.64E+05	1.98E+05	2.25E+05	2.63E+05	1.90E+05
MWSmce169	C21H28O2	Pregna-4,17(20)-diene-3,16-dione (Guggulsterone)	Steroids	Steroid	0.00E+00	0.00E+00	0.00E+00	1.04E+05	1.68E+05	9.88E+04	1.26E+05	1.63E+05	2.22E+05	1.35E+05	1.20E+05	1.64E+05
pmp001018	C27H43NO2	Puqiedinone	Alkaloids	Steroid alkaloids	5.30E+04	6.90E+04	6.56E+04	3.77E+05	5.70E+05	4.58E+05	4.03E+05	6.25E+05	8.19E+05	7.15E+05	5.88E+05	7.14E+05
pmp001080	C28H32O15	Diosmetin-7-O-Neohesperidoside (Neodiosmin)*	Flavonoids	Flavones	0.00E+00	0.00E+00	0.00E+00	5.97E+04	3.97E+04	4.33E+04	7.57E+04	5.46E+04	4.95E+04	4.62E+04	6.65E+04	3.51E+04
Wcsn008814	C16H26O3	(3,7-dimethyl-11-oxododeca-2,6-dienyl)acetate	Others	Others	7.91E+05	8.95E+05	1.10E+06	1.17E+06	1.30E+06	1.14E+06	8.15E+05	2.18E+06	2.39E+06	2.07E+06	1.37E+06	1.92E+06
Wmhp000055	C8H8O4	2,5-Dimethoxybenzoquinone*	Quinones	Quinones	3.16E+05	3.37E+05	2.27E+05	5.01E+05	6.02E+05	4.75E+05	7.20E+05	4.91E+05	2.72E+05	3.79E+05	4.78E+05	2.87E+05
Zjzp081871	C56H90O27	Diosgenin-Glc-Glc-Glc-Glc-Xyl	Steroids	Steroidal saponins	4.30E+04	5.22E+04	2.22E+04	2.48E+05	1.36E+05	1.74E+05	5.30E+04	1.35E+05	1.44E+05	2.76E+05	2.55E+05	3.69E+05
Lmnp002584	C28H32O16	Hispidulin-8-C-(2''-O-glucosyl)glucoside	Flavonoids	Flavones	1.88E+04	6.87E+04	4.29E+04	2.77E+05	3.53E+05	2.86E+05	3.62E+05	2.80E+05	2.85E+05	3.04E+05	2.56E+05	2.99E+05
Zjhp082696	C51H82O24	Pennogenin-Glc-Glc-Glc-Glc	Steroids	Steroidal saponins	1.54E+05	5.10E+04	1.57E+05	2.28E+05	2.25E+05	1.99E+05	2.52E+05	1.83E+05	2.92E+05	2.98E+05	5.41E+05	4.33E+05
Lmsp004721	C28H32O16	Tamarixetin-3-O-glucoside-7-O-rhamnoside	Flavonoids	Flavonols	0.00E+00	0.00E+00	0.00E+00	1.46E+05	1.27E+05	9.73E+04	1.66E+05	7.73E+04	8.01E+04	1.04E+05	5.87E+04	1.16E+05
MWS1877	C14H22O	4-tert-Octylphenol	Phenolic acids	Phenolic acids	8.33E+03	1.11E+04	1.58E+04	3.81E+04	2.37E+04	1.70E+04	1.30E+04	1.96E+04	1.93E+04	2.86E+04	2.98E+04	2.77E+04
Wmjn000315	C13H8O6	1,3,6,7-Tetrahydroxyxanthone	Flavonoids	Flavones	3.61E+04	5.34E+04	3.80E+04	8.76E+04	1.05E+05	1.51E+05	1.21E+05	1.47E+05	1.36E+05	1.66E+05	1.12E+05	1.34E+05
Zjhp082635	C44H68O18	Gentrogenin-Glc-Glc-Xyl*	Steroids	Steroidal saponins	6.80E+04	3.11E+04	2.82E+04	1.03E+05	1.85E+05	1.12E+05	7.77E+04	1.98E+05	4.74E+04	1.84E+05	1.07E+05	1.50E+05
ZINC4349710	C23H24O13	Syringetin-3-o-glucoside	Flavonoids	Flavonols	5.76E+04	8.51E+04	1.22E+05	2.50E+05	2.37E+05	1.11E+05	2.45E+05	2.83E+05	1.75E+05	1.91E+05	1.56E+05	1.60E+05
MWSmce698	C10H12O	2',4'-Dimethylacetophenone	Others	Ketone compounds	1.53E+06	1.64E+06	1.55E+06	2.14E+06	2.09E+06	2.01E+06	1.52E+06	2.21E+06	1.75E+06	1.72E+06	2.41E+06	1.80E+06
Lmsp003852	C29H24O16	1-O-p-Coumaroyl-4,6-(S)-HHDP-β-D-glucose	Phenolic acids	Phenolic acids	1.48E+05	7.45E+04	8.65E+04	9.82E+04	1.34E+05	1.94E+05	1.23E+05	8.00E+04	1.68E+05	5.87E+04	8.87E+04	3.17E+05
pmn001459	C23H32O15	Sibiricose A6	Phenolic acids	Phenolic acids	4.28E+03	5.65E+03	5.10E+03	3.33E+04	1.48E+04	7.36E+03	2.92E+04	1.15E+04	1.39E+04	2.38E+04	1.64E+04	2.70E+04
pmp000172	C23H24O11	5,2'-Dihydroxy-7,8-dimethoxyflavone glycoside*	Flavonoids	Flavones	2.84E+04	2.07E+04	7.14E+03	5.41E+04	1.23E+05	4.80E+04	2.53E+03	1.28E+05	8.60E+04	5.06E+04	2.59E+05	4.26E+04
Lmdn003868	C21H36O9	Frehmaglutoside G	Terpenoids	Monoterpenoids	1.61E+04	3.70E+03	1.95E+04	1.01E+04	1.21E+04	1.88E+04	2.22E+04	1.95E+04	8.66E+03	1.15E+04	1.81E+04	8.97E+03
Qaxn004283	C34H46O18	Acanthoside D	ignans and Coumarir	Lignans	7.47E+03	3.26E+03	2.03E+04	3.90E+04	5.69E+04	2.61E+04	3.61E+04	6.54E+04	5.86E+04	2.44E+04	5.41E+04	2.61E+04
Ladp003428	C57H92O27	dioscoreanoside I	Steroids	Steroidal saponins	4.90E+04	5.30E+03	1.52E+04	5.65E+04	1.48E+05	6.68E+04	3.31E+04	1.73E+05	7.61E+04	1.64E+05	1.21E+05	1.41E+05
pmn001741	C16H22O11	Oleoside	Terpenoids	Monoterpenoids	1.60E+04	1.45E+04	1.97E+04	2.40E+04	2.50E+04	1.24E+04	3.03E+04	2.94E+04	2.37E+04	1.89E+04	2.28E+04	2.29E+04
pme0241	C7H6O2	Benzoic acid	Phenolic acids	Phenolic acids	4.13E+05	4.13E+05	3.96E+05	1.50E+05	3.84E+05	3.03E+05	4.15E+05	7.05E+05	3.65E+05	5.77E+05	3.54E+05	9.47E+04
Lmdp002901	C15H22O8	2,6-Dihydroxy-4-isopropylphenyl-1-O-β-D-glucoside	Phenolic acids	Phenolic acids	7.41E+04	9.30E+04	6.67E+04	8.04E+04	1.22E+05	7.60E+04	1.13E+05	9.10E+04	1.05E+05	6.47E+04	7.09E+04	1.36E+05
Yarn005295	C28H36O13	Tortoside A	ignans and Coumarir	Lignans	6.40E+05	3.04E+05	7.57E+05	3.38E+05	4.38E+05	4.25E+05	2.93E+05	1.00E+06	7.22E+05	6.66E+05	4.10E+05	8.20E+05
mws1035	C33H40O19	Kaempferol-3-O-robinoside-7-O-rhamnoside (Robinin)*	Flavonoids	Flavonols	6.52E+04	7.57E+04	3.78E+04	4.43E+03	5.36E+04	4.92E+04	2.64E+04	3.84E+04	1.84E+04	3.31E+04	5.38E+04	3.59E+04
Qmhp090808	C29H28N2O4	18-Demethylparaensidimerin C	Alkaloids	Alkaloids	2.97E+05	2.84E+05	2.63E+05	7.69E+05	4.56E+05	5.97E+05	3.92E+05	4.63E+05	6.71E+05	3.79E+05	5.36E+05	8.47E+05
Lazn003564	C28H38O14	Picraquassioside C	ignans and Coumarir	Lignans	2.96E+04	3.15E+04	1.16E+04	3.15E+04	5.06E+04	2.90E+04	4.11E+04	3.95E+04	2.24E+04	2.28E+04	1.91E+04	2.97E+04
Hmhn003067	C15H18O8	Phenylpropionic acid-O-β-D-glucopyranoside	Phenolic acids	Phenolic acids	1.54E+04	1.55E+04	1.81E+04	4.57E+04	4.05E+04	5.30E+04	5.22E+04	5.61E+04	2.69E+04	1.57E+04	3.93E+04	3.37E+04
Lcyn000684	C21H30O13	Prustomentoside B	Others	Others	1.12E+05	1.22E+05	6.37E+04	4.24E+04	8.35E+04	8.78E+04	4.50E+04	6.48E+04	6.41E+04	1.08E+05	8.00E+04	4.60E+04
Lmln010063	C14H22O	2,6-Di-tert-butylphenol*	Phenolic acids	Phenolic acids	2.84E+06	1.92E+06	3.28E+06	7.44E+06	5.20E+06	6.22E+06	3.84E+06	4.65E+06	6.25E+06	6.80E+06	5.94E+06	3.84E+06
Zbsp004060	C25H26O13	Apigenin-6-C-xyloside-8-C-arabinoside	Flavonoids	Flavones	3.03E+04	7.81E+04	5.35E+04	1.14E+05	1.38E+05	3.56E+05	1.95E+05	2.78E+05	2.21E+05	1.97E+05	1.56E+05	1.72E+05
pmb0631	C26H28O15	Orientin-6-C-arabinoside	Flavonoids	Flavones	3.51E+04	4.99E+04	6.98E+04	9.06E+04	1.23E+05	8.11E+04	9.96E+04	1.57E+05	1.29E+05	1.54E+05	7.86E+04	9.53E+04
Zjxp101242	C57H94O28	Furost-20(22)-ene-3,26-diol-3-O-glucosyl(1→2)[glucosyl(1→3)]glucosyl(1→4)galactoside-26-O-glucoside (Macrostemonoside E)(iso-1)	Steroids	Steroidal saponins	7.83E+04	5.64E+04	2.73E+04	2.51E+05	1.89E+05	4.49E+05	9.94E+04	1.75E+05	4.51E+05	6.18E+05	3.58E+05	2.57E+05
Hmtn001302	C13H16O8	Glucosyloxybenzoic acid	Phenolic acids	Phenolic acids	3.58E+04	3.57E+04	3.41E+04	7.02E+04	5.23E+04	4.22E+04	5.38E+04	6.98E+04	7.89E+04	6.09E+04	6.54E+04	6.26E+04
Zjhp082695	C51H82O24	Spirost-5-ene-3,14-diol-3-O-glucosyl(1→2)[glucosyl(1→3)]glucosyl(1→4)galactoside	Steroids	Steroidal saponins	3.12E+04	1.43E+04	3.95E+04	1.26E+05	7.53E+04	1.93E+05	1.50E+05	8.03E+04	1.42E+05	2.11E+05	1.52E+05	1.62E+05
Yamn004631	C27H36O12	viburfordosides G	ignans and Coumarir	Lignans	3.95E+04	8.40E+04	9.60E+04	5.00E+04	3.90E+04	5.13E+04	6.01E+04	5.65E+04	1.08E+05	1.36E+05	5.66E+04	6.98E+04
Lmmn005092	C47H72O20	Medicagenic acid-3-O-glucuronide-28-O-rhamnosyl(1,2)-arabinoside	Terpenoids	Triterpene Saponin	3.47E+04	3.39E+04	2.67E+04	1.95E+04	9.36E+04	1.07E+05	4.81E+04	1.17E+05	1.08E+05	5.67E+04	1.36E+05	9.71E+04
Lmnp002845	C22H22O11	Hispidulin-8-C-glucoside*	Flavonoids	Flavones	8.16E+04	6.06E+04	5.47E+04	4.25E+05	3.68E+05	1.46E+05	2.46E+05	2.31E+05	9.23E+04	2.42E+05	1.61E+05	4.41E+05
