# Supplementary material for: Investigation of the Functional Components in Health Beverages Made from Polygonatum cyrtonema Rhizomes Provides Primary Evidence to Support Their Claimed Health Benefits
Source: Metabolites. 2024 Jul 3;14(7):376. doi: 10.3390/metabo14070376 (PMC11279242; doi:10.3390/metabo14070376)
Supplement: Supplementary file 1 [file metabolites-14-00376-s001.zip › Table S2 Detail information of DESMs only present in W7D.pdf]

Table S2 Detail information of identified DESMs only present in W7D smples

Index	Compounds	Class I	Class II	Formula	Level	CAS	W7Da	W7Db	W7Dc	BW40a	BW40b	BW40c	VIP	P-value	Type
pme0376	Naringenin (5,7,4'-Trihydroxyflavanone)	Flavonoids	Flavanones	C15H12O5	1	480-41-1	4.20E+04	2.36E+05	1.35E+05	0.00E+00	0.00E+00	0.00E+00	1.15E+00	1.33E-01	up
MWSHY0072	Methylophiopogonanone B	Flavonoids	Other Flavonoids	C19H20O5	1	74805-91-7	3.38E+05	1.45E+06	4.53E+05	0.00E+00	0.00E+00	0.00E+00	1.16E+00	1.68E-01	up
Lmxp000939	Zarzissine	Alkaloids	Alkaloids	C5H5N5	3	160568-14-9	3.70E+07	3.70E+07	4.16E+07	0.00E+00	0.00E+00	0.00E+00	1.17E+00	1.60E-03	up
Hmhn007438	4'-Demethyl-3,9-dihydropunctatin	Flavonoids	Flavones	C16H14O5	1	96911-00-1	4.46E+05	5.55E+06	2.44E+06	0.00E+00	0.00E+00	0.00E+00	1.15E+00	1.99E-01	up
Zjmp102031	methyl-iso-ophiopogonanone B	Flavonoids	Other Flavonoids	C19H20O5	1	-	3.28E+05	1.32E+06	4.38E+05	0.00E+00	0.00E+00	0.00E+00	1.16E+00	1.57E-01	up
Zjjp092610	Dendronobilin I-iso1	Terpenoids	Sesquiterpenoids	C18H30O3	2	-	3.76E+05	4.52E+05	4.30E+05	0.00E+00	0.00E+00	0.00E+00	1.17E+00	2.89E-03	up
MWSmce254	Ethyl maltol	Phenolic acids	Phenolic acids	C7H8O3	3	4940-11-8	1.23E+06	1.21E+06	1.21E+06	0.00E+00	0.00E+00	0.00E+00	1.17E+00	3.28E-05	up
Zjhp090605	(3R)-5,7-dihydroxy-8-methyl-3-(4'-hydroxybenzyl)-chroman-4-one	Flavonoids	Other Flavonoids	C17H16O5	2	-	8.00E+05	5.81E+06	3.03E+06	0.00E+00	0.00E+00	0.00E+00	1.16E+00	1.57E-01	up
Zjhp090621	5,7-dihydroxy-6,8-dimethyl-3-(2'-methoxy-4'-hydroxybenzyl)-chroman-4-one	Flavonoids	Other Flavonoids	C19H20O6	1	-	1.15E+06	1.86E+06	1.81E+06	0.00E+00	0.00E+00	0.00E+00	1.17E+00	1.97E-02	up
Wcsn009756	Epoxyeleganolactone	Others	Others	C20H30O4	3	-	4.82E+05	4.29E+05	4.49E+05	0.00E+00	0.00E+00	0.00E+00	1.17E+00	1.15E-03	up
Zmsp001775	1-[2-(furan-2-yl)-2-oxoethyl]-5-oxopyrroidine-2-carboxylic acid	Alkaloids	Pyrrole alkaloids	C11H11NO5	2	-	2.02E+06	2.30E+06	2.18E+06	0.00E+00	0.00E+00	0.00E+00	1.17E+00	1.39E-03	up
Wcsn010254	4-[3-(4,8-dimethylnona-3,7-dienyl)-3-methyloxiran-2-yl]butan-2-one	Others	Others	C18H30O2	1	-	6.11E+06	6.50E+06	6.34E+06	0.00E+00	0.00E+00	0.00E+00	1.17E+00	3.27E-04	up
Zjhp090603	(3R)-5,7-dihydroxy-3-(4'-hydroxybenzyl)-chroman-4-one	Flavonoids	Other Flavonoids	C16H14O5	1	-	1.45E+05	2.18E+06	8.03E+05	0.00E+00	0.00E+00	0.00E+00	1.14E+00	2.24E-01	up
pmp001190	(5-8)-Hydroxy-1-(hydroxylmethoxyphenyl)-N2,N3-bis(4-hydroxyphenethyl)-(5-8)-dimethoxy-1,2-dihydronaphthalene-2,3-dicarboxamide	Alkaloids	Amide	C37H38N2O9	3	-	1.81E+05	3.08E+05	3.46E+05	0.00E+00	0.00E+00	0.00E+00	1.17E+00	3.07E-02	up
Smbp002161	8-ethylnorlobelol	Alkaloids	Piperidine alkaloids	C9H19NO	2	-	2.62E+05	1.70E+05	2.83E+05	0.00E+00	0.00E+00	0.00E+00	1.17E+00	2.04E-02	up
MWS1854	4-Aminophenol	Alkaloids	Amide	C6H7NO	2	123-30-8	8.83E+05	9.33E+05	9.75E+05	0.00E+00	0.00E+00	0.00E+00	1.17E+00	8.22E-04	up
Wcsn010496	6,10,14-Trimethylpentadeca-5,9-Diene-2,13-Dione	Others	Others	C18H30O2	3	-	1.25E+05	1.20E+05	1.20E+05	0.00E+00	0.00E+00	0.00E+00	1.17E+00	1.89E-04	up
Lmbn005454	Naringenin-5-methyl ether	Flavonoids	Flavones	C16H14O5	1	61823-56-1	2.37E+04	2.36E+05	1.19E+05	0.00E+00	0.00E+00	0.00E+00	1.13E+00	1.75E-01	up
Lmmn006306	Machilusolide D	Others	Lactones	C18H30O3	1	-	1.68E+06	2.13E+06	1.73E+06	0.00E+00	0.00E+00	0.00E+00	1.17E+00	5.81E-03	up
Jmyn006493	(3R)-5,7-Dihydroxy-6,8-dimethyl-3-(4'-hydroxybenzyl)-chromone-4-one	Flavonoids	Other Flavonoids	C18H18O5	1	-	3.83E+05	2.44E+06	1.21E+06	0.00E+00	0.00E+00	0.00E+00	1.16E+00	1.53E-01	up
Wcsn010224	14-hydroxy-2,6,10-trimethylpentadeca-2,5,10-trien-4-one	Others	Others	C18H30O2	3	-	6.60E+06	6.61E+06	6.74E+06	0.00E+00	0.00E+00	0.00E+00	1.17E+00	4.67E-05	up
Lalp007415	5,7-dihydroxy-6-methyl-3-(4'-hydroxybenzyl)-4-chromanone	Flavonoids	Other Flavonoids	C17H16O5	1	-	4.07E+05	7.55E+06	3.34E+06	0.00E+00	0.00E+00	0.00E+00	1.14E+00	2.11E-01	up
Wbmp011352	Puberaconitidine	Alkaloids	Alkaloids	C37H52N2O11	1	83685-21-6	6.24E+07	6.25E+07	6.80E+07	0.00E+00	0.00E+00	0.00E+00	1.17E+00	8.41E-04	up
Zjmp102042	iso-5,7-dihydroxy-6,8-dimethyl-3-(4'-hydroxy-3'-methoxybenzyl)chroman-4-one	Flavonoids	Other Flavonoids	C19H20O6	1	-	1.11E+06	1.59E+06	1.66E+06	0.00E+00	0.00E+00	0.00E+00	1.17E+00	1.39E-02	up
HJN016	Cannabisin D	Alkaloids	Alkaloids	C36H36N2O8	2	-	5.07E+05	6.44E+05	6.70E+05	0.00E+00	0.00E+00	0.00E+00	1.17E+00	6.85E-03	up
Zahp006974	Cinnamoyltyramine	Alkaloids	Amide	C17H17NO2	3	20384-14-9	3.35E+04	2.44E+05	1.06E+05	0.00E+00	0.00E+00	0.00E+00	1.14E+00	1.74E-01	up
Lamp008287	3-Hydroxy-9,10-dimethoxypterocarpan	Flavonoids	Other Flavonoids	C17H16O5	3	-	3.39E+04	1.64E+05	7.50E+04	0.00E+00	0.00E+00	0.00E+00	1.15E+00	1.41E-01	up
mws1068	Kaempferol (3,5,7,4'-Tetrahydroxyflavone)	Flavonoids	Flavonols	C15H10O6	3	520-18-3	1.54E+04	1.90E+05	9.72E+04	0.00E+00	0.00E+00	0.00E+00	1.11E+00	1.84E-01	up
Zjhp090607	Disporopsin/5,7-dihydroxy-3-(2',4'-dihydroxybenzyl)-chroman-4-one	Flavonoids	Other Flavonoids	C16H14O6	1	-	8.95E+04	3.82E+06	1.35E+06	0.00E+00	0.00E+00	0.00E+00	1.12E+00	2.51E-01	up
Lmfn006411	Cryptostrobin (8-C-Methyl-5,7-Dihydroxyflavanone)	Flavonoids	Flavanones	C16H14O4	2	-	8.44E+03	5.07E+05	8.95E+04	0.00E+00	0.00E+00	0.00E+00	1.04E+00	3.22E-01	up
Zjhp090615	5,7-dihydroxy-8-methyl-3-(2',4'-dihydroxybenzyl)-chroman-4-one	Flavonoids	Other Flavonoids	C17H16O6	3	-	1.25E+04	7.25E+05	2.01E+05	0.00E+00	0.00E+00	0.00E+00	1.06E+00	2.80E-01	up
Jmyp005575	5,7-Dihydroxy-6,8-dimethyl-3-(3'-hydroxy-4'-methoxybenzyl)-chroma4-one	Flavonoids	Other Flavonoids	C19H20O6	1	-	1.21E+06	2.33E+06	2.06E+06	0.00E+00	0.00E+00	0.00E+00	1.17E+00	3.13E-02	up
Jmyn006479	3,5,7-Trihydroxy-6,8-dimethyl-3-(4'-hydroxybenzyl)-chroman-4-one (Polygonatone C)	Flavonoids	Other Flavonoids	C18H18O6	1	-	2.66E+06	4.45E+06	4.14E+06	0.00E+00	0.00E+00	0.00E+00	1.17E+00	2.08E-02	up
Zjhp090609	(E)-5,7-dihydroxy-6,8-dimethyl-3-(4'-hydroxybenzylidene)-chroman-4-one	Flavonoids	Other Flavonoids	C18H16O5	1	-	1.39E+06	3.14E+06	1.53E+06	0.00E+00	0.00E+00	0.00E+00	1.17E+00	6.94E-02	up
Hmgp006095	Lyciumamide C	Alkaloids	Amide	C28H29O7N	1	-	7.38E+05	1.28E+06	1.19E+06	0.00E+00	0.00E+00	0.00E+00	1.17E+00	2.37E-02	up
Lmdn009099	Coniferyl ferulate	Phenolic acids	Phenolic acids	C20H20O6	3	63644-62-2	8.52E+04	4.11E+05	2.28E+05	0.00E+00	0.00E+00	0.00E+00	1.16E+00	1.25E-01	up
Zjxp110301	3-Hydroxy-3,7,11-trimethyldodeca-1,6E,10-trien-9-yl isobutyrate	Terpenoids	Sesquiterpenoids	C18H30O3	1	-	4.30E+05	5.50E+05	4.58E+05	0.00E+00	0.00E+00	0.00E+00	1.17E+00	5.68E-03	up
Wagp009406	2'-Hydroxy-4,4',6'-Trimethoxychalcone; Flavokawain A	Flavonoids	Chalcones	C18H18O5	2	3420-72-2	2.80E+05	1.99E+06	8.61E+05	0.00E+00	0.00E+00	0.00E+00	1.16E+00	1.73E-01	up
Hmhp006830	6,8-Dihydroxy-2-(2-hydroxy-4-methoxybenzyl)-7-methyl-3,4-dihydronaphthalen-1(2H)-one	Flavonoids	Other Flavonoids	C18H18O6	2	-	1.09E+06	2.08E+06	1.88E+06	0.00E+00	0.00E+00	0.00E+00	1.17E+00	3.11E-02	up
Hmhn006723	Disporopsin	Flavonoids	Flavones	C16H14O6	1	-	1.40E+05	4.04E+06	1.71E+06	0.00E+00	0.00E+00	0.00E+00	1.13E+00	2.26E-01	up
Lamp007163	4'-Demethyleucomin glucoside	Flavonoids	Other Flavonoids	C22H22O10	1	-	5.28E+05	4.74E+06	3.65E+06	0.00E+00	0.00E+00	0.00E+00	1.15E+00	1.43E-01	up
Lmjn013497	Agathic acid	Terpenoids	Ditepenoids	C20H30O4	3	640-28-8	4.46E+05	3.98E+05	3.97E+05	0.00E+00	0.00E+00	0.00E+00	1.17E+00	1.56E-03	up
Wcdp007560	1-Vaccenoyl-Glycero-3-	Others	Others	C26H52NO7P	3	-	3.60E+07	3.64E+07	4.75E+07	0.00E+00	0.00E+00	0.00E+00	1.17E+00	8.80E-03	up
Lmlp004754	Ehretioside B	Alkaloids	Amide	C14H17NO7	3	156368-84-2	1.81E+05	1.65E+05	1.23E+05	0.00E+00	0.00E+00	0.00E+00	1.17E+00	1.23E-02	up
Zjhp090635	5,7-dihydroxy-6,8-dimethyl-3-(4'-methoxybenzyl)-chroman-4-one	Flavonoids	Other Flavonoids	C19H20O5	1	-	2.87E+05	1.24E+06	4.21E+05	0.00E+00	0.00E+00	0.00E+00	1.16E+00	1.61E-01	up
MWSmce089	p-Coumaroyltyramine	Alkaloids	Amide	C17H17NO3	1	36417-86-4	2.82E+07	4.02E+07	4.21E+07	0.00E+00	0.00E+00	0.00E+00	1.17E+00	1.36E-02	up
pmp000970	Hispanolone	Terpenoids	Ditepenoids	C20H30O3	1	18676-07-8	2.71E+05	2.50E+05	4.15E+05	0.00E+00	0.00E+00	0.00E+00	1.17E+00	2.66E-02	up
MW0139629	Sakuranetin	Flavonoids	Flavanones	C16H14O5	3	2957-21-3	2.58E+05	3.25E+06	1.38E+06	0.00E+00	0.00E+00	0.00E+00	1.15E+00	2.03E-01	up
MWStz038	Grossamide	Alkaloids	Amide	C36H36N2O8	1	80510-06-1	2.94E+06	3.69E+06	3.89E+06	0.00E+00	0.00E+00	0.00E+00	1.17E+00	6.71E-03	up
Zjhp090619	(3R)-5,7-dihydroxy-8-methyl-3-(2'-hydroxy-4'-methoxybenzyl)-chroman-4-one	Flavonoids	Other Flavonoids	C18H18O6	1	-	8.22E+04	1.80E+06	3.55E+05	0.00E+00	0.00E+00	0.00E+00	1.13E+00	2.97E-01	up
Zasn010433	9,12-Octadecadienoic acid,ethyl ester	Others	Others	C20H36O2	3	6114-21-2	2.88E+05	1.86E+05	1.76E+05	0.00E+00	0.00E+00	0.00E+00	1.17E+00	2.59E-02	up
Qayp008402	Sileneoside H	Steroids	Steroidal saponins	C35H56O14	3	-	2.85E+05	3.64E+05	3.06E+05	0.00E+00	0.00E+00	0.00E+00	1.17E+00	5.36E-03	up
Hmqp002567	4-Hydroxycinnamic acid p-hydroxyphenethylamine	Phenolic acids	Phenolic acids	C17H17NO3	3	-	2.80E+07	4.11E+07	4.08E+07	0.00E+00	0.00E+00	0.00E+00	1.17E+00	1.36E-02	up
pmn001498	Isodendrocrepine	Alkaloids	Alkaloids	C33H44N2O3	1	50906-94-0	5.38E+05	9.38E+05	5.31E+05	0.00E+00	0.00E+00	0.00E+00	1.17E+00	3.80E-02	up
Zjmp102043	2,5,7-trihydroxy-6,8-dimethyl-3-(4'-methoxybenzyl)chroman-4-one	Flavonoids	Other Flavonoids	C19H20O6	3	-	3.51E+04	9.82E+04	3.50E+04	0.00E+00	0.00E+00	0.00E+00	1.16E+00	1.17E-01	up
Wbtn006715	1,2,4,5,8-pentahydroxy-6-methylanthracene-9,10-dione	Flavonoids	Other Flavonoids	C15H10O7	3	-	3.86E+04	1.55E+05	5.14E+04	0.00E+00	0.00E+00	0.00E+00	1.15E+00	1.57E-01	up
Wmtn012065	Piperazirum	Alkaloids	Alkaloids	C15H30N2O	3	-	9.07E+05	1.09E+06	9.13E+05	0.00E+00	0.00E+00	0.00E+00	1.17E+00	3.70E-03	up
Jmmn008945	2 α,3 α,19 α,23-tetrahydroxyursolane-12-en-28-oic acid	Terpenoids	Triterpene	C29H46O6	3	-	1.12E+04	1.41E+04	1.02E+04	0.00E+00	0.00E+00	0.00E+00	1.17E+00	9.44E-03	up
Wdhp006324	(2e)-3-(4-hydroxyphenyl)-n-[2-(4-hydroxyphenyl)ethyl]prop-2-enimidic acid	Alkaloids	Isoquinoline alkaloids	C17H17NO3	1	-	1.30E+07	1.74E+07	1.73E+07	0.00E+00	0.00E+00	0.00E+00	1.17E+00	8.29E-03	up
Zjmp102044	5,7-dihydroxy-6-methyl-3(R)-(2,4-dihydroxybenzyl)chroman-4-one	Flavonoids	Other Flavonoids	C17H16O6	3	-	2.74E+04	6.14E+05	2.51E+05	0.00E+00	0.00E+00	0.00E+00	1.11E+00	2.24E-01	up
Wcsn010375	Methyl (4E,8E,11E)-5,9,13-trimethyltetradeca-4,8,11-trienoate	Others	Others	C18H30O2	3	-	1.95E+05	1.92E+05	1.73E+05	0.00E+00	0.00E+00	0.00E+00	1.17E+00	1.39E-03	up
pmn001573	Desmethylagrimonolide-6-O-glucoside	Phenolic acids	Phenolic acids	C23H26O10	2	1257408-55-1	6.08E+04	4.70E+05	3.02E+05	0.00E+00	0.00E+00	0.00E+00	1.14E+00	1.44E-01	up
Zjhp090618	5,7-Dihydroxy-6,8-dimethyl-3-(2',4'-hydroxybenzyl)chroman-4-One	Flavonoids	Other Flavonoids	C18H18O6	2	-	8.47E+05	1.69E+06	1.56E+06	0.00E+00	0.00E+00	0.00E+00	1.17E+00	3.51E-02	up
mws0914	3,5,7-Trihydroxyflavanone (Pinobanksin)	Flavonoids	Flavanonols	C15H12O5	1	548-82-3	6.41E+04	3.22E+05	1.83E+05	0.00E+00	0.00E+00	0.00E+00	1.15E+00	1.26E-01	up
Qagp006800	Lyciumamide B	Alkaloids	Amide	C36H36N2O8	1	-	1.59E+06	2.22E+06	2.10E+06	0.00E+00	0.00E+00	0.00E+00	1.17E+00	9.25E-03	up
mws4113	3'-Hydroxy-4'-O-methylglabridin	Flavonoids	Other Flavonoids	C21H22O5	2	175554-11-7	3.70E+04	3.75E+04	4.38E+04	0.00E+00	0.00E+00	0.00E+00	1.17E+00	3.05E-03	up
Zjmp102003	iso-ophiopogonanone B	Flavonoids	Other Flavonoids	C18H18O5	3	-	2.37E+05	3.50E+05	2.88E+05	0.00E+00	0.00E+00	0.00E+00	1.17E+00	1.23E-02	up
Lmjn102210	Desmethylagrimonolide-6-O-β-D-glucopyranoside	gnans and Coumarin	Coumarins	C23H26O10	2	-	4.90E+04	3.91E+05	3.08E+05	0.00E+00	0.00E+00	0.00E+00	1.14E+00	1.37E-01	up
Zbsp007084	Butin; 7,3',4'-Trihydroxyflavanone	Flavonoids	Flavanones	C15H12O5	3	492-14-8	8.81E+04	3.80E+05	2.18E+05	0.00E+00	0.00E+00	0.00E+00	1.16E+00	1.14E-01	up
Wbtn014060	Nigrolineaxanthone K	Flavonoids	Other Flavonoids	C23H22O5	2	864516-31-4	5.24E+04	4.44E+04	5.17E+04	0.00E+00	0.00E+00	0.00E+00	1.17E+00	2.62E-03	up
MWSprf162	(25R)-Spirost-4-ene-3,12-dione	Steroids	Steroidal saponins	C27H38O4	3	6875-60-1	6.26E+04	5.66E+04	4.91E+04	0.00E+00	0.00E+00	0.00E+00	1.17E+00	4.76E-03	up
Hmhp006887	6,8-Dihydroxy-2-(4-hydroxybenzyl)-5,7-dimethyl-3,4-dihydronaphthalen-1(2H)-one	Flavonoids	Other Flavonoids	C18H18O5	1	-	2.54E+05	2.18E+06	1.02E+06	0.00E+00	0.00E+00	0.00E+00	1.15E+00	1.76E-01	up
Hajp009554	2',4'-Dihydroxy-4-methoxychalcone; (E)-Cardamonin	Flavonoids	Chalcones	C16H14O4	1	19309-14-9	1.89E+04	1.16E+06	2.08E+05	0.00E+00	0.00E+00	0.00E+00	1.07E+00	3.21E-01	up
MWS20177	5-hydroxy-anthraquinone-2-carboxylic acid	Quinones	Anthraquinone	C15H8O5	2	-	5.99E+04	8.28E+04	7.59E+04	0.00E+00	0.00E+00	0.00E+00	1.17E+00	8.58E-03	up
Wbmn010746	Negundoin A	Terpenoids	Ditepenoids	C22H34O5	3	-	5.69E+06	5.44E+06	6.07E+06	0.00E+00	0.00E+00	0.00E+00	1.17E+00	1.04E-03	up
Zmwp005562	cis-N-p-Coumaroyltyramine	Alkaloids	Amide	C17H17NO3	1	-	2.63E+07	3.64E+07	4.06E+07	0.00E+00	0.00E+00	0.00E+00	1.17E+00	1.47E-02	up
Zjhp082605	Diosgenin	Steroids	Steroid	C27H42O3	1	-	1.15E+06	1.29E+06	1.66E+06	0.00E+00	0.00E+00	0.00E+00	1.17E+00	1.19E-02	up
pmp000579	Diosmetin-7-O-galactoside	Flavonoids	Flavones	C22H22O11	3	-	5.34E+05	3.37E+05	4.60E+05	0.00E+00	0.00E+00	0.00E+00	1.17E+00	1.64E-02	up
Zmdn006929	Aloe-Emodin-9-Anthrone	Quinones	Anthraquinone	C15H12O4	3	6247-99-0	3.32E+03	1.12E+05	2.63E+04	0.00E+00	0.00E+00	0.00E+00	1.01E+00	2.90E-01	up
pmb0142	Caffeic aldehyde	Phenolic acids	Phenolic acids	C9H8O3	3	141632-15-7	4.49E+05	4.77E+05	5.04E+05	0.00E+00	0.00E+00	0.00E+00	1.17E+00	1.09E-03	up
Lahp004442	3,4-Dihydro-4-(4'-hydroxyphenyl)-5,7-dihydroxycoumarin	gnans and Coumarin	Coumarins	C15H12O5	3	-	7.89E+04	3.72E+05	2.97E+05	0.00E+00	0.00E+00	0.00E+00	1.15E+00	1.05E-01	up
Lalp010122	7,3'-Dihydroxy-4'-methoxy-8-methylflavan	Flavonoids	Other Flavonoids	C17H18O4	3	87733-81-1	4.13E+04	3.26E+05	1.51E+05	0.00E+00	0.00E+00	0.00E+00	1.14E+00	1.73E-01	up
Lmjp003044	Isorhamnetin-3-O-Glucoside	Flavonoids	Flavonols	C22H22O12	3	5041-82-7	4.70E+05	8.40E+05	9.64E+05	0.00E+00	0.00E+00	0.00E+00	1.17E+00	3.62E-02	up
Wcgn012055	Araucarolone	Terpenoids	Ditepenoids	C20H30O4	3	-	4.99E+05	4.21E+05	4.39E+05	0.00E+00	0.00E+00	0.00E+00	1.17E+00	2.70E-03	up
Wbkp008793	(3S,3'S)-N,N'-((1R,2R)-4-((3,4,5-trihydroxy-6-methyltetrahydro-2H-pyran-2-yl)oxy)cyclohexane-1,2-diyl)bis(2-methyl-2,3,4,9-tetrahydro-1H-pyrido[3,4-b]indole-3-carboxamide)	Alkaloids	Alkaloids	C38H48N6O7	2	-	2.75E+05	2.64E+05	3.28E+05	0.00E+00	0.00E+00	0.00E+00	1.17E+00	4.74E-03	up
pmp001253	Cannabisin F	Alkaloids	Amide	C36H36N2O8	1	163136-19-4	1.45E+06	1.73E+06	1.59E+06	0.00E+00	0.00E+00	0.00E+00	1.17E+00	2.46E-03	up
Lamp009532	4'-demethyl-3,9-dihydroeucomin	Flavonoids	Other Flavonoids	C16H14O5	2	107585-77-3	6.72E+04	9.31E+05	3.83E+05	0.00E+00	0.00E+00	0.00E+00	1.13E+00	2.10E-01	up
pmp001073	Synephrine; 4-[1-Hydroxy-2-(methylamino)ethyl]phenol	Alkaloids	Amide	C9H13NO2	3	94-07-5	1.44E+06	1.62E+06	2.09E+06	0.00E+00	0.00E+00	0.00E+00	1.17E+00	1.23E-02	up
Jmyn006217	Polygonatone B	Others	Others	C18H18O7	3	-	2.20E+04	4.10E+04	9.82E+03	0.00E+00	0.00E+00	0.00E+00	1.14E+00	1.16E-01	up
Hmgp006701	Lyciumamide A	Alkaloids	Amide	C36H36O8N2	1	-	1.75E+06	2.13E+06	1.96E+06	0.00E+00	0.00E+00	0.00E+00	1.17E+00	3.29E-03	up
Lamp009984	4'-Demethyleucomin	Flavonoids	Other Flavonoids	C16H12O5	3	34818-83-2	2.95E+04	1.73E+05	7.85E+04	0.00E+00	0.00E+00	0.00E+00	1.14E+00	1.56E-01	up
Sadp008019	5-formyl-2,7-dihydroxy-1,6-dimethyl-9,10-dihydrophenanthrene	Terpenoids	Ditepenoids	C17H16O3	2	-	3.02E+05	4.04E+05	4.01E+05	0.00E+00	0.00E+00	0.00E+00	1.17E+00	8.05E-03	up
Wbjp006632	Tribulusamide A	Alkaloids	Alkaloids	C36H36N2O8	3	-	1.94E+06	2.32E+06	2.39E+06	0.00E+00	0.00E+00	0.00E+00	1.17E+00	4.00E-03	up
Wcdp010708	Octadeca-2,9,12,15-tetraen-1-ol	Others	Others	C18H30O	2	-	2.53E+06	2.18E+06	2.22E+06	0.00E+00	0.00E+00	0.00E+00	1.17E+00	2.36E-03	up
Zjhp090636	5,7-dihydroxy-3-(4'-hydroxybenzylidene)-chroman-4-one	Flavonoids	Other Flavonoids	C16H12O5	3	-	1.93E+04	1.70E+05	8.84E+04	0.00E+00	0.00E+00	0.00E+00	1.13E+00	1.67E-01	up
Zjmp102002	ophiopogonanone B	Flavonoids	Other Flavonoids	C18H18O5	1	1316759-83-7	2.78E+05	2.15E+06	9.31E+05	0.00E+00	0.00E+00	0.00E+00	1.15E+00	1.78E-01	up
pmp001187	1-(Dihydroxyphenyl)-N2,N3-bis(4-hydroxyphenethyl)-(5-8)-dimethoxy-1,2dihydronaphthalene-2,3-dicarboxamide	Alkaloids	Amide	C36H36N2O8	1	-	2.25E+06	2.73E+06	2.95E+06	0.00E+00	0.00E+00	0.00E+00	1.17E+00	5.91E-03	up
pmp001269	Hexadecyl ethanolamine	Alkaloids	Alkaloids	C18H39NO	3	-	3.79E+05	7.35E+05	4.21E+05	0.00E+00	0.00E+00	0.00E+00	1.17E+00	4.51E-02	up
Zjhp090620	5,7-dihydroxy-6,8-dimethyl-3(R,S)-(3'-hydroxy-4'-methoxybenzyl)-chroman-4-one	Flavonoids	Other Flavonoids	C19H20O6	1	-	4.27E+04	3.63E+05	6.85E+04	0.00E+00	0.00E+00	0.00E+00	1.13E+00	2.64E-01	up
WaYn006121	Salicin 7-O-Ferulate	Phenolic acids	Phenolic acids	C23H26O10	2	-	5.30E+03	6.61E+04	5.80E+04	0.00E+00	0.00E+00	0.00E+00	1.07E+00	1.52E-01	up
Zjhp090606	(3R)-5,7-dihydroxyl-6-methyl-3-(4'-hydroxylbenzyl)-chroman-4-one	Flavonoids	Other Flavonoids	C17H16O5	1	-	4.31E+05	6.28E+06	2.83E+06	0.00E+00	0.00E+00	0.00E+00	1.15E+00	2.02E-01	up
Zjmp102025	Desmethyl-ophiopogonone B	Flavonoids	Other Flavonoids	C17H14O5	3	-	1.17E+05	1.82E+05	8.94E+04	0.00E+00	0.00E+00	0.00E+00	1.17E+00	4.26E-02	up
Hasp010605	(S)-2,3-dihydroxypropyl (9Z,12Z,15Z)-octadeca-9,12,15-trienoate	Others	Others	C21H36O4	3	129828-41-7	2.86E+06	2.81E+06	2.91E+06	0.00E+00	0.00E+00	0.00E+00	1.17E+00	1.14E-04	up
Lmyp008454	2-(Hydroxymethyl)Anthraquinone	Quinones	Anthraquinone	C15H10O3	2	17241-59-7	6.00E+04	6.44E+04	8.49E+04	0.00E+00	0.00E+00	0.00E+00	1.17E+00	1.18E-02	up
mws0789	Pinocembrin (Dihydrochrysin)	Flavonoids	Flavanones	C15H12O4	2	480-39-7	5.59E+03	8.41E+04	2.28E+04	0.00E+00	0.00E+00	0.00E+00	1.07E+00	2.56E-01	up
Lmqn006260	2',3',4',5,7-Pentahydroxyflavone	Flavonoids	Flavones	C15H10O7	3	-	6.00E+04	1.78E+05	4.94E+04	0.00E+00	0.00E+00	0.00E+00	1.16E+00	1.46E-01	up
pmn001497	Dendrocrepine	Alkaloids	Alkaloids	C33H44N2O3	1	51020-39-4	4.40E+05	7.19E+05	2.92E+05	0.00E+00	0.00E+00	0.00E+00	1.17E+00	6.08E-02	up
Wcsn011332	Ethyl (10Z,13Z)-hexadeca-10,13-dienoate	Others	Others	C18H32O2	3	-	1.52E+06	1.46E+06	1.41E+06	0.00E+00	0.00E+00	0.00E+00	1.17E+00	5.14E-04	up
HJAP050	N-Feruloyl-3-methoxytyramine	Alkaloids	Amide	C19H21NO5	3	78510-19-7	1.37E+05	1.51E+05	1.13E+05	0.00E+00	0.00E+00	0.00E+00	1.17E+00	6.54E-03	up
Smdp007779	8-Demethylfarrerol	Flavonoids	Flavanones	C16H14O5	3	-	4.42E+04	3.58E+05	1.60E+05	0.00E+00	0.00E+00	0.00E+00	1.14E+00	1.78E-01	up
Hmgp002036	Nepetin-7-O-glucoside(Nepitrin)	Flavonoids	Flavones	C22H22O12	3	569-90-4	3.55E+05	7.87E+05	8.81E+05	0.00E+00	0.00E+00	0.00E+00	1.17E+00	5.32E-02	up
Labn004865	Hydroxy isoliquiritigenin glucoside	Flavonoids	Chalcones	C21H22O10	3	-	2.62E+04	1.05E+05	7.94E+04	0.00E+00	0.00E+00	0.00E+00	1.15E+00	9.45E-02	up
Wagp007639	N-(4-hydroxyphenethyl)cinnamamide	Alkaloids	Alkaloids	C17H17NO2	3	-	3.08E+04	2.18E+05	1.09E+05	0.00E+00	0.00E+00	0.00E+00	1.14E+00	1.59E-01	up
Wcsn010349	Moniliferanone D	Others	Others	C22H32O4	3	-	1.18E+07	1.42E+07	1.11E+07	0.00E+00	0.00E+00	0.00E+00	1.17E+00	5.74E-03	up
Zjmp102024	Desmethyl-iso-ophiopogonone B	Flavonoids	Other Flavonoids	C17H14O5	3	-	1.23E+05	1.71E+05	1.02E+05	0.00E+00	0.00E+00	0.00E+00	1.17E+00	2.27E-02	up
Lamp008416	3-(4-hydroxy)-5,7-dihydroxy-9-hydroxychroman-4-one	Flavonoids	Other Flavonoids	C16H14O6	2	-	1.78E+05	4.16E+06	1.57E+06	0.00E+00	0.00E+00	0.00E+00	1.13E+00	2.34E-01	up
Wbjp004916	Cephalanthrin A	Alkaloids	Alkaloids	C17H12N2O4	3	-	9.36E+06	1.26E+07	1.05E+07	0.00E+00	0.00E+00	0.00E+00	1.17E+00	7.63E-03	up
Zjhp090623	5-hydroxy-7-methoxy-6,8-dimethyl-3-(2'-hydroxy-4'-methoxybenzyl)-chroman-4-one	Flavonoids	Other Flavonoids	C20H22O6	2	-	4.90E+04	9.65E+04	1.58E+05	0.00E+00	0.00E+00	0.00E+00	1.16E+00	8.50E-02	up
Wbmp008021	(14-Ethyl-4,6,19-trimethoxy-9,11-dioxa-14-azaheptacyclo[10.7.2.12,5.01,13.03,8.08,12.016,20]docosan-21-yl)acetate	Alkaloids	Alkaloids	C26H39NO7	2	-	3.76E+06	4.05E+06	3.85E+06	0.00E+00	0.00E+00	0.00E+00	1.17E+00	5.03E-04	up
Hmhp006479	2-(2,4-dihydroxybenzyl)-6,8-dihydroxy-7-methyl-3,4-dihydronaphthalen-1(2H)-one	Flavonoids	Other Flavonoids	C17H16O6	3	-	1.09E+05	1.44E+05	1.41E+05	0.00E+00	0.00E+00	0.00E+00	1.17E+00	7.29E-03	up
Zjhp090611	(3S)-5,7-dihydroxy-3-[(4-methoxyphenyl)methyl]-6-methyl-2,3-dihydrochromen-4-one	Flavonoids	Other Flavonoids	C18H18O5	3	-	2.83E+05	3.54E+05	2.91E+05	0.00E+00	0.00E+00	0.00E+00	1.17E+00	5.21E-03	up
Zjhp090634	(E)-3-(3',4'-dihydroxybenzylidene)-5,7-dihydroxy-6,8-dimethylchroman-4-one	Flavonoids	Other Flavonoids	C18H16O6	3	-	6.78E+04	4.37E+04	5.50E+04	0.00E+00	0.00E+00	0.00E+00	1.17E+00	1.53E-02	up
Zjhp090604	5,7-dihydroxy-3-(4'-hydroxybenzyl)-chroman-4-one	Flavonoids	Other Flavonoids	C16H14O5	3	-	3.22E+04	3.29E+05	1.52E+05	0.00E+00	0.00E+00	0.00E+00	1.13E+00	1.86E-01	up
Zjhp090617	5-hydroxy-3-(2-hydroxy-4-methoxybenzyl)-7-methoxychroman-4-one	Flavonoids	Other Flavonoids	C18H18O6	3	-	2.23E+04	6.94E+05	1.09E+05	0.00E+00	0.00E+00	0.00E+00	1.09E+00	3.22E-01	up
pmf0557	Orange I	Alkaloids	Alkaloids	C16H11N2NaO4S	2	523-44-4	2.79E+04	2.21E+05	1.34E+05	0.00E+00	0.00E+00	0.00E+00	1.13E+00	1.50E-01	up
mws0066	Isorhamnetin; 3'-Methoxy-3,4',5,7-Tetrahydroxyflavone	Flavonoids	Flavonols	C16H12O7	3	480-19-3	2.78E+04	8.05E+04	1.99E+04	0.00E+00	0.00E+00	0.00E+00	1.15E+00	1.54E-01	up
mws1179	Naringenin-7-O-glucoside (Prunin)	Flavonoids	Flavanones	C21H22O10	3	529-55-5	2.74E+04	1.02E+05	9.19E+04	0.00E+00	0.00E+00	0.00E+00	1.15E+00	8.77E-02	up
pmb0382	O-Feruloyl 4-hydroxycoumarin	gnans and Coumarin	Coumarins	C19H14O6	3	-	1.77E+05	1.57E+05	2.21E+05	0.00E+00	0.00E+00	0.00E+00	1.17E+00	1.01E-02	up
pmp000436	13-Methyl-27-norolean-14-en-3-ol (Taraxerol)	Terpenoids	Triterpene	C30H50O	3	127-22-0	8.19E+04	7.99E+04	6.78E+04	0.00E+00	0.00E+00	0.00E+00	1.17E+00	3.29E-03	up
Hmjp005906	N-Trans-Feruloyl-3'-O-methyldopamine	Alkaloids	Amide	C19H21NO5	3	-	1.09E+05	1.40E+05	1.53E+05	0.00E+00	0.00E+00	0.00E+00	1.17E+00	9.17E-03	up
Zjhp090614	5,7-dihydroxy-6-methyl-3-(2',4'-dihydroxybenzyl)-chroman-4-one	Flavonoids	Other Flavonoids	C17H16O6	2	-	1.01E+05	2.68E+06	7.52E+05	0.00E+00	0.00E+00	0.00E+00	1.13E+00	2.68E-01	up
Zjhp090622	(3R)-5,7-dihydroxy-6,8-dimethyl-3-(2'-hydroxy-4'-methoxybenzyl)-chroman-4-one	Flavonoids	Other Flavonoids	C19H20O6	1	-	4.07E+05	4.26E+05	4.28E+05	0.00E+00	0.00E+00	0.00E+00	1.17E+00	2.58E-04	up
Lmzn005381	Jujuboside I	Terpenoids	Triterpene Saponin	C53H86O22	3	-	1.22E+04	9.45E+03	7.63E+03	0.00E+00	0.00E+00	0.00E+00	1.17E+00	1.82E-02	up
mws0983	N-Oleoylethanolamine	Alkaloids	Alkaloids	C20H39NO2	2	111-58-0	8.40E+04	1.11E+05	8.87E+04	0.00E+00	0.00E+00	0.00E+00	1.17E+00	7.71E-03	up
MWS1888	Phenyl-beta-D-glucoside	Phenolic acids	Phenolic acids	C12H16O6	3	1464-44-4	7.43E+04	5.30E+04	6.16E+04	0.00E+00	0.00E+00	0.00E+00	1.17E+00	9.49E-03	up
Wbjp006749	N-(4-Hydroxyphenethyl)-2-(3-methoxy-4-hydroxyphenyl)-5-[(Z)-2-[2-methoxy-4-[(E)-2-[(4-hydroxyphenethyl)carbamoyl]ethenyl]phenoxy]-2-[(4-hydroxyphenethyl)carbamoyl]ethenyl]-7-methoxy-2,3-dihydrobenzofuran-3-carboxamide	Alkaloids	Alkaloids	C54H53N3O12	2	-	2.29E+05	3.46E+05	2.67E+05	0.00E+00	0.00E+00	0.00E+00	1.17E+00	1.46E-02	up
Zbsn012113	Diacetoxy-[6]-gingerdiol	Phenolic acids	Phenolic acids	C21H32O6	3	143615-75-2	2.34E+04	2.86E+04	2.54E+04	0.00E+00	0.00E+00	0.00E+00	1.17E+00	3.35E-03	up
Ybnn006457	Artocarpanone	Flavonoids	Flavanones	C16H14O6	2	-	3.61E+04	1.39E+05	7.18E+04	0.00E+00	0.00E+00	0.00E+00	1.15E+00	1.12E-01	up
pme2960	Naringenin chalcone; 2',4,4',6'-Tetrahydroxychalcone	Flavonoids	Chalcones	C15H12O5	3	73692-50-9	4.97E+04	2.94E+05	1.42E+05	0.00E+00	0.00E+00	0.00E+00	1.15E+00	1.51E-01	up
Zjmp102023	iso-methyl-ophiopogonone B	Flavonoids	Other Flavonoids	C19H18O5	3	-	2.94E+04	4.45E+04	3.09E+04	0.00E+00	0.00E+00	0.00E+00	1.17E+00	1.83E-02	up
Lamn006359	4'-demethyl-3,9-dihydroeucomin glucoside	Flavonoids	Other Flavonoids	C22H24O10	3	-	1.92E+04	2.08E+05	1.38E+05	0.00E+00	0.00E+00	0.00E+00	1.12E+00	1.58E-01	up
mws0055	Tangeretin (4',5,6,7,8-Pentamethoxyflavone)	Flavonoids	Flavones	C20H20O7	3	481-53-8	5.29E+04	4.12E+04	2.59E+04	0.00E+00	0.00E+00	0.00E+00	1.17E+00	3.64E-02	up
Wdbn009958	21-hydroperoxy-8-hydroxy-5,19-dimethyl-15,18,20-trioxapentacyclo[14.5.1.04,13.05,10.019,22]docosa-1,10-dien-14-one	Steroids	Steroidal saponins	C21H28O7	2	-	6.88E+04	7.38E+04	7.06E+04	0.00E+00	0.00E+00	0.00E+00	1.17E+00	4.17E-04	up
Zjmp102041	5,7-dihydroxy-6,8-dimethyl-3-(4'-hydroxy-3'-methoxybenzyl)chroman-4-one	Flavonoids	Other Flavonoids	C19H20O6	1	-	3.67E+05	4.07E+05	5.20E+05	0.00E+00	0.00E+00	0.00E+00	1.17E+00	1.11E-02	up
MWSHY0064	Isorhamnetin-3-O-neohesperidoside	Flavonoids	Flavonols	C28H32O16	3	55033-90-4	1.32E+05	6.33E+04	6.19E+04	0.00E+00	0.00E+00	0.00E+00	1.17E+00	6.51E-02	up
pmp001265	2-Amino-4-dihydroxy octadecyl galactoside	Alkaloids	Alkaloids	C24H49NO8	2	-	4.01E+05	3.12E+05	3.00E+05	0.00E+00	0.00E+00	0.00E+00	1.17E+00	8.81E-03	up
MWS1852	4-Methoxybenzaldehyde	Others	Aldehyde compounds	C8H8O2	3	123-11-5	2.24E+05	2.75E+05	2.29E+05	0.00E+00	0.00E+00	0.00E+00	1.17E+00	4.42E-03	up
pmn001506	Oleanolic acid-3-O-glucoside	Terpenoids	Triterpene Saponin	C36H58O8	3	3391-80-8	1.85E+04	1.46E+04	2.16E+04	0.00E+00	0.00E+00	0.00E+00	1.17E+00	1.20E-02	up
Lssp210093	7-hydroxy-N-trans-ferulic tyramine	Alkaloids	Amide	C18H19NO5	1	-	3.15E+05	5.38E+05	3.20E+05	0.00E+00	0.00E+00	0.00E+00	1.17E+00	3.37E-02	up
Lmsn010558	Squamocin K	Others	Others	C35H62O6	3	161169-70-6	8.88E+03	1.32E+04	1.13E+04	0.00E+00	0.00E+00	0.00E+00	1.17E+00	1.21E-02	up
Yshs000041	Vibsanin J	Terpenoids	Ditepenoids	C26H36O7	3	-	8.20E+05	4.83E+05	9.09E+05	0.00E+00	0.00E+00	0.00E+00	1.17E+00	2.96E-02	up
Qmyp101334	JiangxiBaiyingsu I	Terpenoids	Sesquiterpenoids	C18H28O3	2	-	7.82E+04	1.02E+05	1.04E+05	0.00E+00	0.00E+00	0.00E+00	1.17E+00	7.67E-03	up
Wbmn010845	Pentandranoic acid C	Terpenoids	Ditepenoids	C20H30O4	3	-	8.46E+04	8.38E+04	7.71E+04	0.00E+00	0.00E+00	0.00E+00	1.17E+00	8.38E-04	up
pmp000608	Aurantio-obtusin-6-O-Glucoside	Quinones	Anthraquinone	C23H24O12	3	129025-96-3	2.56E+05	3.20E+05	3.46E+05	0.00E+00	0.00E+00	0.00E+00	1.17E+00	7.57E-03	up
Hmjp002999	Isorhamnetin-3-O-rutinoside	Flavonoids	Flavonols	C28H32O16	3	604-80-8	1.26E+05	1.16E+05	1.29E+05	0.00E+00	0.00E+00	0.00E+00	1.17E+00	9.58E-04	up
pme2954	Quercetin	Flavonoids	Flavonols	C15H10O7	3	117-39-5	6.31E+04	1.31E+05	4.40E+04	0.00E+00	0.00E+00	0.00E+00	1.16E+00	9.46E-02	up
MWSmce169	Pregna-4,17(20)-diene-3,16-dione (Guggulsterone)	Steroids	Steroid	C21H28O2	3	95975-55-6	1.04E+05	1.68E+05	9.88E+04	0.00E+00	0.00E+00	0.00E+00	1.17E+00	3.12E-02	up
pmp001080	Diosmetin-7-O-Neohesperidoside (Neodiosmin)	Flavonoids	Flavones	C28H32O15	3	38665-01-9	5.97E+04	3.97E+04	4.33E+04	0.00E+00	0.00E+00	0.00E+00	1.17E+00	1.64E-02	up
Lmsp004721	Tamarixetin-3-O-glucoside-7-O-rhamnoside	Flavonoids	Flavonols	C28H32O16	3	-	1.46E+05	1.27E+05	9.73E+04	0.00E+00	0.00E+00	0.00E+00	1.17E+00	1.28E-02	up
