# Supplementary material for: Investigation of the Functional Components in Health Beverages Made from Polygonatum cyrtonema Rhizomes Provides Primary Evidence to Support Their Claimed Health Benefits
Source: Metabolites. 2024 Jul 3;14(7):376. doi: 10.3390/metabo14070376 (PMC11279242; doi:10.3390/metabo14070376)
Supplement: Supplementary file 1 [file metabolites-14-00376-s001.zip › Table S3 Ten terpenoids clustering variation pattern 1.pdf]

Table S3 Ten terpenoids clustering variation pattern 1

Index	Compounds	Class I	Class II	Formula	Level	CAS	BW40a	BW40b	BW40c	W7Da	W7Db	W7Dc	W14Da	W14Db	W14Dc	W21Da	W21Db	W21Dc
Lmmn005168	Siegesbeckic acid	Terpenoids	Ditepenoids	C20H32O4	1	-	2.28E+05	4.18E+04	2.12E+05	6.56E+05	1.79E+06	5.27E+04	2.67E+05	5.14E+05	3.65E+05	2.29E+05	5.29E+04	5.05E+04
Lmbn014696	Pimaric acid	Terpenoids	Ditepenoids	C20H30O2	1	127-27-5	4.87E+05	6.65E+05	6.70E+05	4.01E+06	1.12E+07	1.90E+06	4.17E+06	3.30E+06	3.00E+06	2.38E+06	1.16E+06	9.95E+05
MWSslk208	Kaurenoic Acid	Terpenoids	Ditepenoids	C20H30O2	1	6730-83-2	5.13E+05	6.51E+05	7.94E+05	4.42E+06	1.13E+07	1.94E+06	4.90E+06	3.47E+06	3.19E+06	2.53E+06	1.22E+06	9.86E+05
Cmmn013378	Levopimaric acid	Terpenoids	Ditepenoids	C20H30O2	1	79-54-9	4.68E+05	5.21E+05	6.06E+05	3.90E+06	9.17E+06	1.85E+06	3.82E+06	2.99E+06	3.13E+06	2.20E+06	1.21E+06	9.82E+05
Lajp006846	Imbricatusol D	Terpenoids	Ditepenoids	C20H30O3	3	-	9.76E+04	1.82E+04	7.78E+04	5.49E+05	1.51E+06	6.98E+04	2.27E+05	4.51E+05	2.91E+05	1.57E+05	5.08E+04	5.77E+04
Sayp010368	phyllanflexoid A	Terpenoids	Ditepenoids	C20H26O3	3	-	1.47E+06	9.06E+05	1.59E+06	7.34E+06	1.98E+07	1.61E+06	6.38E+06	6.73E+06	5.08E+06	2.41E+06	1.12E+06	1.35E+06
Wmxp009647	Momilactone A	Terpenoids	Ditepenoids	C20H26O3	3	51415-07-7	1.10E+05	9.21E+04	1.24E+05	8.22E+05	2.18E+06	1.59E+05	5.21E+05	6.05E+05	4.80E+05	2.13E+05	1.55E+05	1.72E+05
Zaxn006988	Isopimaric acid	Terpenoids	Ditepenoids	C20H30O2	1	5835-26-7	3.65E+05	6.32E+05	6.22E+05	3.62E+06	9.41E+06	1.78E+06	4.04E+06	3.08E+06	2.79E+06	2.41E+06	1.13E+06	8.95E+05
Zaxn005783	Methyl neoabietate	Terpenoids	Ditepenoids	C21H32O2	2	3310-97-2	2.51E+04	2.10E+04	1.61E+04	1.73E+05	4.44E+05	1.95E+05	1.55E+05	1.69E+05	1.36E+05	0.00E+00	0.00E+00	0.00E+00
Sayp011213	Cleistanthol	Terpenoids	Ditepenoids	C20H28O3	3	24465-21-2	6.11E+04	4.68E+04	5.04E+04	1.81E+05	4.06E+05	3.98E+04	8.44E+04	1.15E+05	1.02E+05	4.83E+04	3.33E+04	2.78E+04
