# Supplementary material for: Investigation of the Functional Components in Health Beverages Made from Polygonatum cyrtonema Rhizomes Provides Primary Evidence to Support Their Claimed Health Benefits
Source: Metabolites. 2024 Jul 3;14(7):376. doi: 10.3390/metabo14070376 (PMC11279242; doi:10.3390/metabo14070376)
Supplement: Supplementary file 1 [file metabolites-14-00376-s001.zip › Table S4 KEGG pathways and annotated compounds.pdf]

Table S4 KEGG pathways and annotated compounds from three comparison groups

W7D vs BW40

Kegg_pathway	ko_ID	Sig_compound	Compound	Sig_compound_all	Compound_all	IndexList	CIDList	Pathway
Flavonoid biosynthesis	ko00941	12	13	35	110	pme0376;mws1068;MW0139629;mws0914;Zbsp007084;pmb3074;mws0178;mws0789;MWSHY0163;mws1179;pme2960;pme2954	C00509+C05903+C09833+C09826+C09614+C12208+C00852+C09827+C00974+C09099+C06561+C00389	https:\\www.genome.jp\dbget-bin\www_bget?map00941
Isoflavonoid biosynthesis	ko00943	1	1	35	110	pme0376	C00509	https:\\www.genome.jp\dbget-bin\www_bget?map00943
Metabolic pathways	ko01100	17	74	35	110	pme0376;MWSslk208;mws1068;mws0097;pme2693;Lmrn003000;pmb2497;pmf0070;pme3083;MWSHY0163;pme1292;mws0014;mws0467;mws1179;pme2960;pmb0496;pme2954	C00509+C11874+C05903+C05366+C02714+C05607+C05584+C01753+C05653+C00974+C00544+C01494+C01744+C09099+C06561+C18325+C00389	https:\\www.genome.jp\dbget-bin\www_bget?map01100
Biosynthesis of secondary metabolites	ko01110	19	52	35	110	pme0376;MWSslk208;mws1068;mws0097;MW0139629;Lmrn003000;pmb3074;pmb0142;mws0178;mws0906;pmf0070;pma0149;MWSHY0163;mws0014;Zmpn003044;pmp000436;pme2960;pmb0496;pme2954	C00509+C11874+C05903+C05366+C09833+C05607+C12208+C10945+C00852+C00761+C01753+C02887+C00974+C01494+C09315+C08637+C06561+C18325+C00389	https:\\www.genome.jp\dbget-bin\www_bget?map01110
Diterpenoid biosynthesis	ko00904	1	2	35	110	MWSslk208	C11874	https:\\www.genome.jp\dbget-bin\www_bget?map00904
Flavone and flavonol biosynthesis	ko00944	6	8	35	110	mws1068;pme3227;MWSHY0132;mws0047;mws1434;pme2954	C05903+C12628+C01750+C12627+C01714+C00389	https:\\www.genome.jp\dbget-bin\www_bget?map00944
Biosynthesis of various plant secondary metabolites	ko00999	3	9	35	110	mws0097;Zmpn003044;pmb0496	C05366+C09315+C18325	https:\\www.genome.jp\dbget-bin\www_bget?map00999
Arginine and proline metabolism	ko00330	1	8	35	110	pme2693	C02714	https:\\www.genome.jp\dbget-bin\www_bget?map00330
Tryptophan metabolism	ko00380	2	7	35	110	pmb0818;pme3083	C05660+C05653	https:\\www.genome.jp\dbget-bin\www_bget?map00380
Phenylalanine metabolism	ko00360	1	6	35	110	Lmrn003000	C05607	https:\\www.genome.jp\dbget-bin\www_bget?map00360
Tropane, piperidine and pyridine alkaloid biosynthesis	ko00960	1	5	35	110	Lmrn003000	C05607	https:\\www.genome.jp\dbget-bin\www_bget?map00960
Phenylpropanoid biosynthesis	ko00940	6	15	35	110	pmb3074;pmb0142;mws0178;mws0906;pma0149;mws0014	C12208+C10945+C00852+C00761+C02887+C01494	https:\\www.genome.jp\dbget-bin\www_bget?map00940
Stilbenoid, diarylheptanoid and gingerol biosynthesis	ko00945	2	3	35	110	pmb3074;mws0178	C12208+C00852	https:\\www.genome.jp\dbget-bin\www_bget?map00945
Tyrosine metabolism	ko00350	2	10	35	110	pmb2497;pme1292	C05584+C00544	https:\\www.genome.jp\dbget-bin\www_bget?map00350
Steroid biosynthesis	ko00100	1	2	35	110	pmf0070	C01753	https:\\www.genome.jp\dbget-bin\www_bget?map00100
Ubiquinone and other terpenoid-quinone biosynthesis	ko00130	1	4	35	110	pme1292	C00544	https:\\www.genome.jp\dbget-bin\www_bget?map00130
Biosynthesis of cofactors	ko01240	1	6	35	110	pme1292	C00544	https:\\www.genome.jp\dbget-bin\www_bget?map01240
Anthocyanin biosynthesis	ko00942	1	1	35	110	Zmcp002839	C16314	https:\\www.genome.jp\dbget-bin\www_bget?map00942
Glycolysis / Gluconeogenesis	ko00010	1	1	35	110	Ymjm000154	C01451	https:\\www.genome.jp\dbget-bin\www_bget?map00010
Sesquiterpenoid and triterpenoid biosynthesis	ko00909	1	1	35	110	pmp000436	C08637	https:\\www.genome.jp\dbget-bin\www_bget?map00909

W14D vs W7D

No KEGG pathways and annotated compounds

W21D vs W14D

Diterpenoid biosynthesis	ko00904	2	2	2	110	MWSslk208;Wmxp009647	C11874+C18015	https:\\www.genome.jp\dbget-bin\www_bget?map00904
Metabolic pathways	ko01100	1	74	2	110	MWSslk208	C11874	https:\\www.genome.jp\dbget-bin\www_bget?map01100
Biosynthesis of secondary metabolites	ko01110	2	52	2	110	MWSslk208;Wmxp009647	C11874+C18015	https:\\www.genome.jp\dbget-bin\www_bget?map01110
