# Supplementary material for: Investigation of the Functional Components in Health Beverages Made from Polygonatum cyrtonema Rhizomes Provides Primary Evidence to Support Their Claimed Health Benefits
Source: Metabolites. 2024 Jul 3;14(7):376. doi: 10.3390/metabo14070376 (PMC11279242; doi:10.3390/metabo14070376)
Supplement: Supplementary file 1 [file metabolites-14-00376-s001.zip › Table S5 Homoisoflavonoids and amide alkaloids.pdf]

Table S5 Homoisoflavonoids identified in this study

Index	Compounds	Formula	Level	W7Da	W7Db	W7Dc	BW40a	BW40b	BW40c	VIP	P-value	Log2FC	Type
Zjhp090605	(3R)-5,7-dihydroxy-8-methyl-3-(4'-hydroxybenzyl)-chroman-4-one	C17H16O5	2	8.00E+05	5.81E+06	3.03E+06	**0.00E+00**	**0.00E+00**	**0.00E+00**	1.16E+00	1.57E-01	#DIV/0!	up
Zjhp090621	5,7-dihydroxy-6,8-dimethyl-3-(2'-methoxy-4'-hydroxybenzyl)-chroman-4-one	C19H20O6	1	1.15E+06	1.86E+06	1.81E+06	**0.00E+00**	**0.00E+00**	**0.00E+00**	1.17E+00	1.97E-02	#DIV/0!	up
Zjhp090603	(3R)-5,7-dihydroxy-3-(4'-hydroxybenzyl)-chroman-4-one	C16H14O5	1	1.45E+05	2.18E+06	8.03E+05	**0.00E+00**	**0.00E+00**	**0.00E+00**	1.14E+00	2.24E-01	#DIV/0!	up
Lalp007415	5,7-dihydroxy-6-methyl-3-(4'-hydroxybenzyl)-4-chromanone	C17H16O5	1	4.07E+05	7.55E+06	3.34E+06	**0.00E+00**	**0.00E+00**	**0.00E+00**	1.14E+00	2.11E-01	#DIV/0!	up
Zjmp102042	Iso-5,7-dihydroxy-6,8-dimethyl-3-(4'-hydroxy-3'-methoxybenzyl)-chroman-4-one	C19H20O6	1	1.11E+06	1.59E+06	1.66E+06	**0.00E+00**	**0.00E+00**	**0.00E+00**	1.17E+00	1.39E-02	#DIV/0!	up
Zjhn090628	(3R)-5,7-Dihydroxy-8-methyl-3-(2',4'-dihydroxybenzyl)-chroman-4-one	C17H16O6	2	1.17E+05	2.79E+05	2.40E+05	7.09E+03	4.07E+03	7.57E+03	1.15E+00	5.16E-02	5.09E+00	up
Zjhp090607	Disporopsin/5,7-dihydroxy-3-(2',4'-dihydroxybenzyl)-chroman-4-one	C16H14O6	1	8.95E+04	3.82E+06	1.35E+06	**0.00E+00**	**0.00E+00**	**0.00E+00**	1.12E+00	2.51E-01	#DIV/0!	up
Zjhp090615	5,7-dihydroxy-8-methyl-3-(2',4'-dihydroxybenzyl)-chroman-4-one	C17H16O6	3	1.25E+04	7.25E+05	2.01E+05	**0.00E+00**	**0.00E+00**	**0.00E+00**	1.06E+00	2.80E-01	#DIV/0!	up
Jmyn006479	3,5,7-Trihydroxy-6,8-dimethyl-3-(4'-hydroxybenzyl)-chroman-4-one (Polygonatone C)	C18H18O6	1	2.66E+06	4.45E+06	4.14E+06	**0.00E+00**	**0.00E+00**	**0.00E+00**	1.17E+00	2.08E-02	#DIV/0!	up
Zjhp090609	(E)-5,7-dihydroxy-6,8-dimethyl-3-(4'-hydroxybenzylidene)-chroman-4-one	C18H16O5	1	1.39E+06	3.14E+06	1.53E+06	**0.00E+00**	**0.00E+00**	**0.00E+00**	1.17E+00	6.94E-02	#DIV/0!	up
Zjhp090635	5,7-dihydroxy-6,8-dimethyl-3-(4'-methoxybenzyl)-chroman-4-one	C19H20O5	1	2.87E+05	1.24E+06	4.21E+05	**0.00E+00**	**0.00E+00**	**0.00E+00**	1.16E+00	1.61E-01	#DIV/0!	up
Zjhp090619	(3R)-5,7-dihydroxy-8-methyl-3-(2'-hydroxy-4'-methoxybenzyl)-chroman-4-one	C18H18O6	1	8.22E+04	1.80E+06	3.55E+05	**0.00E+00**	**0.00E+00**	**0.00E+00**	1.13E+00	2.97E-01	#DIV/0!	up
Zjmp102043	2,5,7-trihydroxy-6,8-dimethyl-3-(4'-methoxybenzyl)-chroman-4-one	C19H20O6	3	3.51E+04	9.82E+04	3.50E+04	**0.00E+00**	**0.00E+00**	**0.00E+00**	1.16E+00	1.17E-01	#DIV/0!	up
Zjmp102044	5,7-dihydroxy-6-methyl-3(R)-(2,4-dihydroxybenzyl)-chroman-4-one	C17H16O6	3	2.74E+04	6.14E+05	2.51E+05	**0.00E+00**	**0.00E+00**	**0.00E+00**	1.11E+00	2.24E-01	#DIV/0!	up
Zjhp090618	5,7-Dihydroxy-6,8-dimethyl-3-(2',4'-hydroxybenzyl)-chroman-4-One	C18H18O6	2	8.47E+05	1.69E+06	1.56E+06	**0.00E+00**	**0.00E+00**	**0.00E+00**	1.17E+00	3.51E-02	#DIV/0!	up
Zjhp090636	5,7-dihydroxy-3-(4'-hydroxybenzylidene)-chroman-4-one	C16H12O5	3	1.93E+04	1.70E+05	8.84E+04	**0.00E+00**	**0.00E+00**	**0.00E+00**	1.13E+00	1.67E-01	#DIV/0!	up
Zjhp090620	5,7-dihydroxy-6,8-dimethyl-3(R,S)-(3'-hydroxy-4'-methoxybenzyl)-chroman-4-one	C19H20O6	1	4.27E+04	3.63E+05	6.85E+04	**0.00E+00**	**0.00E+00**	**0.00E+00**	1.13E+00	2.64E-01	#DIV/0!	up
Zjhp090606	(3R)-5,7-dihydroxyl-6-methyl-3-(4'-hydroxylbenzyl)-chroman-4-one	C17H16O5	1	4.31E+05	6.28E+06	2.83E+06	**0.00E+00**	**0.00E+00**	**0.00E+00**	1.15E+00	2.02E-01	#DIV/0!	up
Lamp008416	3-(4-hydroxy)-5,7-dihydroxy-9-hydroxychroman-4-one	C16H14O6	2	1.78E+05	4.16E+06	1.57E+06	**0.00E+00**	**0.00E+00**	**0.00E+00**	1.13E+00	2.34E-01	#DIV/0!	up
Zjhp090623	5-hydroxy-7-methoxy-6,8-dimethyl-3-(2'-hydroxy-4'-methoxybenzyl)-chroman-4-one	C20H22O6	2	4.90E+04	9.65E+04	1.58E+05	**0.00E+00**	**0.00E+00**	**0.00E+00**	1.16E+00	8.50E-02	#DIV/0!	up
Zjhp090634	(E)-3-(3',4'-dihydroxybenzylidene)-5,7-dihydroxy-6,8-dimethylchroman-4-one	C18H16O6	3	6.78E+04	4.37E+04	5.50E+04	**0.00E+00**	**0.00E+00**	**0.00E+00**	1.17E+00	1.53E-02	#DIV/0!	up
Zjhp090604	5,7-dihydroxy-3-(4'-hydroxybenzyl)-chroman-4-one	C16H14O5	3	3.22E+04	3.29E+05	1.52E+05	**0.00E+00**	**0.00E+00**	**0.00E+00**	1.13E+00	1.86E-01	#DIV/0!	up
Zjhp090617	5-hydroxy-3-(2-hydroxy-4-methoxybenzyl)-7-methoxychroman-4-one	C18H18O6	3	2.23E+04	6.94E+05	1.09E+05	**0.00E+00**	**0.00E+00**	**0.00E+00**	1.09E+00	3.22E-01	#DIV/0!	up
Zjhp090625	(E)-7-O-β-D-glucopyranoside-5-hydroxy-3-(4'-hydroxybenzylidene)-chroman-4-one	C22H22O10	1	7.88E+05	4.68E+06	3.42E+06	3.21E+04	3.68E+04	2.42E+04	1.13E+00	1.25E-01	6.58E+00	up
Zjhp090614	5,7-dihydroxy-6-methyl-3-(2',4'-dihydroxybenzyl)-chroman-4-one	C17H16O6	2	1.01E+05	2.68E+06	7.52E+05	**0.00E+00**	**0.00E+00**	**0.00E+00**	1.13E+00	2.68E-01	#DIV/0!	up
Zjhp090622	(3R)-5,7-dihydroxy-6,8-dimethyl-3-(2'-hydroxy-4'-methoxybenzyl)-chroman-4-one	C19H20O6	1	4.07E+05	4.26E+05	4.28E+05	**0.00E+00**	**0.00E+00**	**0.00E+00**	1.17E+00	2.58E-04	#DIV/0!	up
Zjmp102041	5,7-dihydroxy-6,8-dimethyl-3-(4'-hydroxy-3'-methoxybenzyl)chroman-4-one	C19H20O6	1	3.67E+05	4.07E+05	5.20E+05	**0.00E+00**	**0.00E+00**	**0.00E+00**	1.17E+00	1.11E-02	#DIV/0!	up
Jmyp005575	5,7-Dihydroxy-6,8-dimethyl-3-(3'-hydroxy-4'-methoxybenzyl)-chroman-4-one	C19H20O6	1	1.21E+06	2.33E+06	2.06E+06	**0.00E+00**	**0.00E+00**	**0.00E+00**	1.17E+00	3.13E-02	#DIV/0!	up

Table S5 Amide alkaloids identified in this study

Index	Compounds	Formula	Level	W7Da	W7Db	W7Dc	BW40a	BW40b	BW40c	VIP	P-value	Log2FC	Type
Lmmp001410	Dihydrocaffeoylputrescine	C13H20N2O3	2	4.93E+06	6.02E+06	6.04E+06	3.12E+06	2.93E+06	2.10E+06	1.09E+00	3.95E-03	1.06E+00	up
pmp001189	(5-8)-Hydroxy-1-(hydroxyldimethoxyphenyl)-N2,N3-bis(4-hydroxyphenethyl)-(5-8)-dimethoxy-1,2-dihydronaphthalene-2,3-dicarboxamide	C38H40N2O10	2	3.52E+05	3.84E+05	2.95E+05	2.54E+04	6.06E+03	8.02E+03	1.13E+00	4.30E-03	4.71E+00	up
pmp001190	(5-8)-Hydroxy-1-(hydroxylmethoxyphenyl)-N2,N3-bis(4-hydroxyphenethyl)-(5-8)-dimethoxy-1,2-dihydronaphthalene-2,3-dicarboxamide	C37H38N2O9	3	1.81E+05	3.08E+05	3.46E+05	**0.00E+00**	**0.00E+00**	**0.00E+00**	1.17E+00	3.07E-02	#DIV/0!	up
MWS1854	4-Aminophenol	C6H7NO	2	8.83E+05	9.33E+05	9.75E+05	**0.00E+00**	**0.00E+00**	**0.00E+00**	1.17E+00	8.22E-04	#DIV/0!	up
Zahp006974	Cinnamoyltyramine	C17H17NO2	3	3.35E+04	2.44E+05	1.06E+05	**0.00E+00**	**0.00E+00**	**0.00E+00**	1.14E+00	1.74E-01	#DIV/0!	up
Hmgp006095	Lyciumamide C	C28H29O7N	1	7.38E+05	1.28E+06	1.19E+06	**0.00E+00**	**0.00E+00**	**0.00E+00**	1.17E+00	2.37E-02	#DIV/0!	up
Lmlp004754	Ehretioside B	C14H17NO7	3	1.81E+05	1.65E+05	1.23E+05	**0.00E+00**	**0.00E+00**	**0.00E+00**	1.17E+00	1.23E-02	#DIV/0!	up
MWSmce089	p-Coumaroyltyramine	C17H17NO3	1	2.82E+07	4.02E+07	4.21E+07	**0.00E+00**	**0.00E+00**	**0.00E+00**	1.17E+00	1.36E-02	#DIV/0!	up
Lssp210092	N-trans-ferulic tyramine	C18H19NO4	1	3.59E+07	5.18E+07	4.89E+07	1.74E+05	1.94E+05	1.97E+05	1.17E+00	1.14E-02	7.92E+00	up
MWStz038	Grossamide	C36H36N2O8	1	2.94E+06	3.69E+06	3.89E+06	**0.00E+00**	**0.00E+00**	**0.00E+00**	1.17E+00	6.71E-03	#DIV/0!	up
Wafp004987	N-Feruloylphenylacetamide	C18H17NO4	1	1.16E+07	9.09E+06	1.19E+07	8.04E+05	6.27E+05	5.89E+05	1.17E+00	7.44E-03	4.01E+00	up
MWStz221	N-Cis-Feruloyltyramine	C18H19NO4	1	3.86E+07	5.12E+07	5.11E+07	1.68E+05	2.23E+05	1.78E+05	1.17E+00	7.96E-03	7.95E+00	up
Wafp004641	N-Feruloyltyramine 4-glucoside	C24H29NO9	1	3.55E+06	5.67E+06	4.46E+06	9.53E+05	9.33E+05	1.16E+06	1.15E+00	2.73E-02	2.17E+00	up
Qagp006800	Lyciumamide B	C36H36N2O8	1	1.59E+06	2.22E+06	2.10E+06	**0.00E+00**	**0.00E+00**	**0.00E+00**	1.17E+00	9.25E-03	#DIV/0!	up
HJN011	Tataramide A	C17H17NO4	2	8.39E+05	1.63E+06	1.37E+06	3.50E+04	2.50E+04	2.06E+04	1.16E+00	3.30E-02	5.58E+00	up
pmb0492	N',N'',N'''-p-Coumaroyl-cinnamoyl-caffeoyl spermidine	C34H37N3O6	3	4.53E+05	6.13E+05	4.87E+05	1.85E+05	2.09E+05	1.53E+05	1.14E+00	1.36E-02	1.51E+00	up
Zahp005843	N-Trans-Sinapoyltyramine	C19H21NO5	1	1.92E+06	2.95E+06	2.93E+06	6.51E+04	6.93E+04	6.37E+04	1.17E+00	1.74E-02	5.30E+00	up
mws1433	N-Feruloyltyramine; Moupinamide	C18H19NO4	1	3.79E+07	5.22E+07	4.95E+07	1.44E+05	1.36E+05	2.06E+05	1.17E+00	8.77E-03	8.17E+00	up
Zmwp005562	cis-N-p-Coumaroyltyramine	C17H17NO3	1	2.63E+07	3.64E+07	4.06E+07	**0.00E+00**	**0.00E+00**	**0.00E+00**	1.17E+00	1.47E-02	#DIV/0!	up
pmp001253	Cannabisin F	C36H36N2O8	1	1.45E+06	1.73E+06	1.59E+06	**0.00E+00**	**0.00E+00**	**0.00E+00**	1.17E+00	2.46E-03	#DIV/0!	up
pmp001073	Synephrine; 4-[1-Hydroxy-2-(methylamino)ethyl]phenol	C9H13NO2	3	1.44E+06	1.62E+06	2.09E+06	**0.00E+00**	**0.00E+00**	**0.00E+00**	1.17E+00	1.23E-02	#DIV/0!	up
pmn001727	N-(4-O-(Glucosyl)-E-feruloyl)-tyramine	C24H29NO9	1	9.99E+05	1.62E+06	1.31E+06	2.45E+05	4.16E+05	2.71E+05	1.12E+00	2.36E-02	2.08E+00	up
Hmgp006701	Lyciumamide A	C36H36O8N2	1	1.75E+06	2.13E+06	1.96E+06	**0.00E+00**	**0.00E+00**	**0.00E+00**	1.17E+00	3.29E-03	#DIV/0!	up
Hmjp005089	(2Z)-N-[2-(3,4-Dihydroxyphenyl)-2-hydroxyethyl]-3-(4-methoxyphenyl)-2-propenamide	C18H19NO5	1	8.03E+04	7.95E+04	1.15E+05	1.53E+03	6.16E+03	8.00E+03	1.11E+00	1.62E-02	4.13E+00	up
pmp001187	1-(Dihydroxyphenyl)-N2,N3-bis(4-hydroxyphenethyl)-(5-8)-dimethoxy-1,2dihydronaphthalene-2,3-dicarboxamide	C36H36N2O8	1	2.25E+06	2.73E+06	2.95E+06	**0.00E+00**	**0.00E+00**	**0.00E+00**	1.17E+00	5.91E-03	#DIV/0!	up
Zblp005777	N-trans-ferulicacidacylp-hydroxyphenylethylamine	C18H19NO4	1	3.77E+07	5.65E+07	5.21E+07	1.66E+05	1.34E+05	1.69E+05	1.17E+00	1.34E-02	8.29E+00	up
HJAP050	N-Feruloyl-3-methoxytyramine	C19H21NO5	3	1.37E+05	1.51E+05	1.13E+05	**0.00E+00**	**0.00E+00**	**0.00E+00**	1.17E+00	6.54E-03	#DIV/0!	up
Hmjp005906	N-Trans-Feruloyl-3'-O-methyldopamine	C19H21NO5	3	1.09E+05	1.40E+05	1.53E+05	**0.00E+00**	**0.00E+00**	**0.00E+00**	1.17E+00	9.17E-03	#DIV/0!	up
pmb0500	N-p-Coumaroyl-N'-feruloylputrescine	C23H26N2O5	3	2.01E+05	1.53E+05	3.43E+05	3.62E+04	5.92E+04	6.72E+04	1.08E+00	8.58E-02	2.10E+00	up
MWStz208	N-Feruloyloctopamine	C18H19NO5	1	2.77E+05	4.39E+05	1.14E+06	4.30E+04	7.38E+04	5.14E+04	1.09E+00	1.68E-01	3.47E+00	up
pmb0496	N-Feruloylagmatine	C15H22N4O3	3	3.47E+05	3.66E+05	2.55E+05	6.01E+04	6.81E+04	8.47E+04	1.15E+00	1.50E-02	2.18E+00	up
Qmjp110218	N-Feruloyl dopamine	C18H19NO5	1	7.64E+04	5.39E+04	4.87E+04	1.49E+04	9.74E+03	4.02E+03	1.08E+00	1.80E-02	2.65E+00	up
Lssp210093	7-hydroxy-N-trans-ferulic tyramine	C18H19NO5	1	3.15E+05	5.38E+05	3.20E+05	**0.00E+00**	**0.00E+00**	**0.00E+00**	1.17E+00	3.37E-02	#DIV/0!	up
