# Supplementary figures and images for: Investigation of the Functional Components in Health Beverages Made from Polygonatum cyrtonema Rhizomes Provides Primary Evidence to Support Their Claimed Health Benefits
Source: Metabolites. 2024 Jul 3;14(7):376. doi: 10.3390/metabo14070376 (PMC11279242; doi:10.3390/metabo14070376)

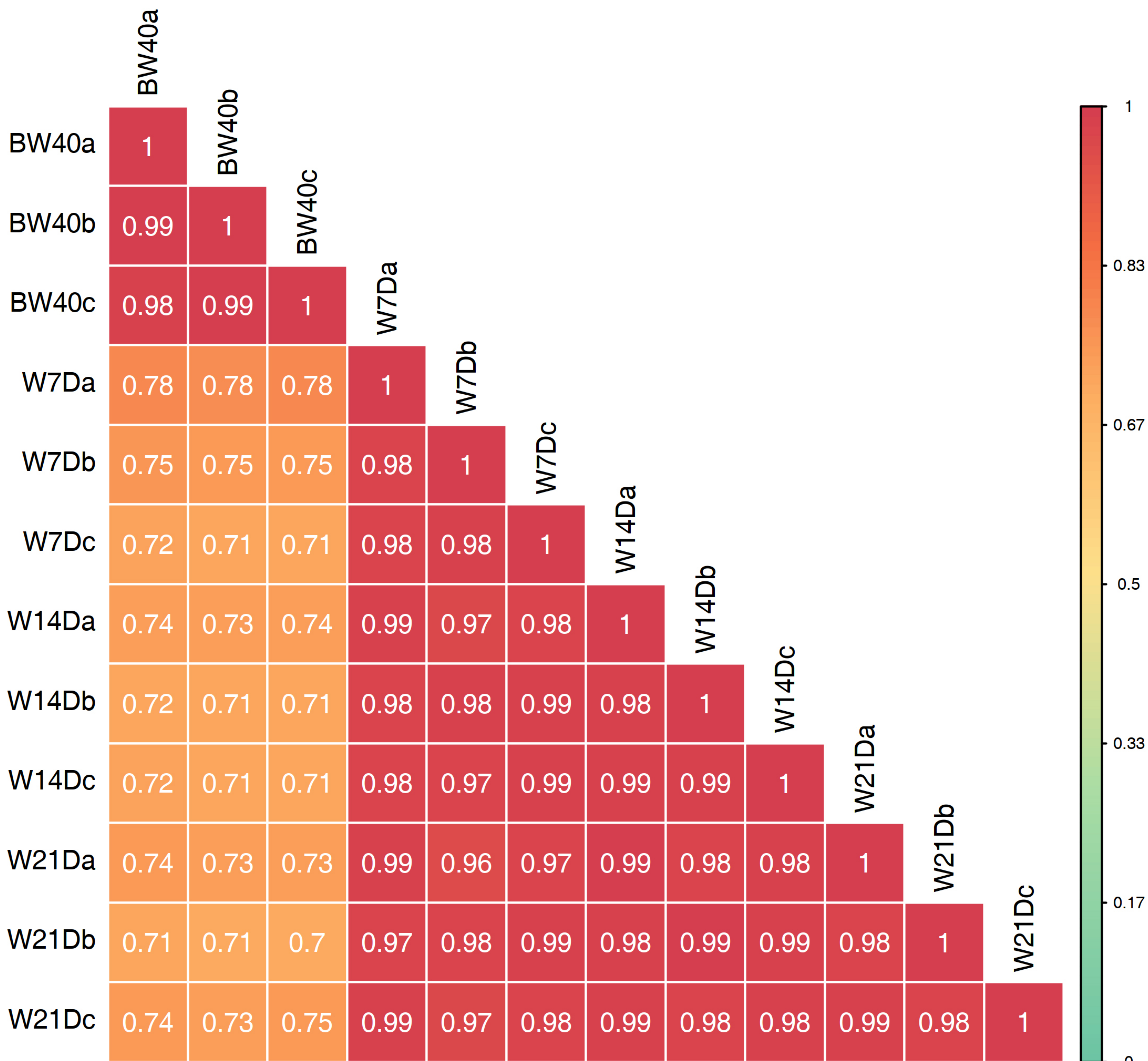

Supplement: Supplementary file 1 [file metabolites-14-00376-s001.zip › Fig. S1 Correlation analysis of secondary metabolites.pdf]
