# Supplementary material for: Investigation of the Functional Components in Health Beverages Made from Polygonatum cyrtonema Rhizomes Provides Primary Evidence to Support Their Claimed Health Benefits
Source: Metabolites. 2024 Jul 3;14(7):376. doi: 10.3390/metabo14070376 (PMC11279242; doi:10.3390/metabo14070376)
Supplement: Supplementary file 1 [file Fig. S2 Flavonoid biosynthesis pathways.pdf › Fig. S2 Flavonoid biosynthesis pathways.pdf]

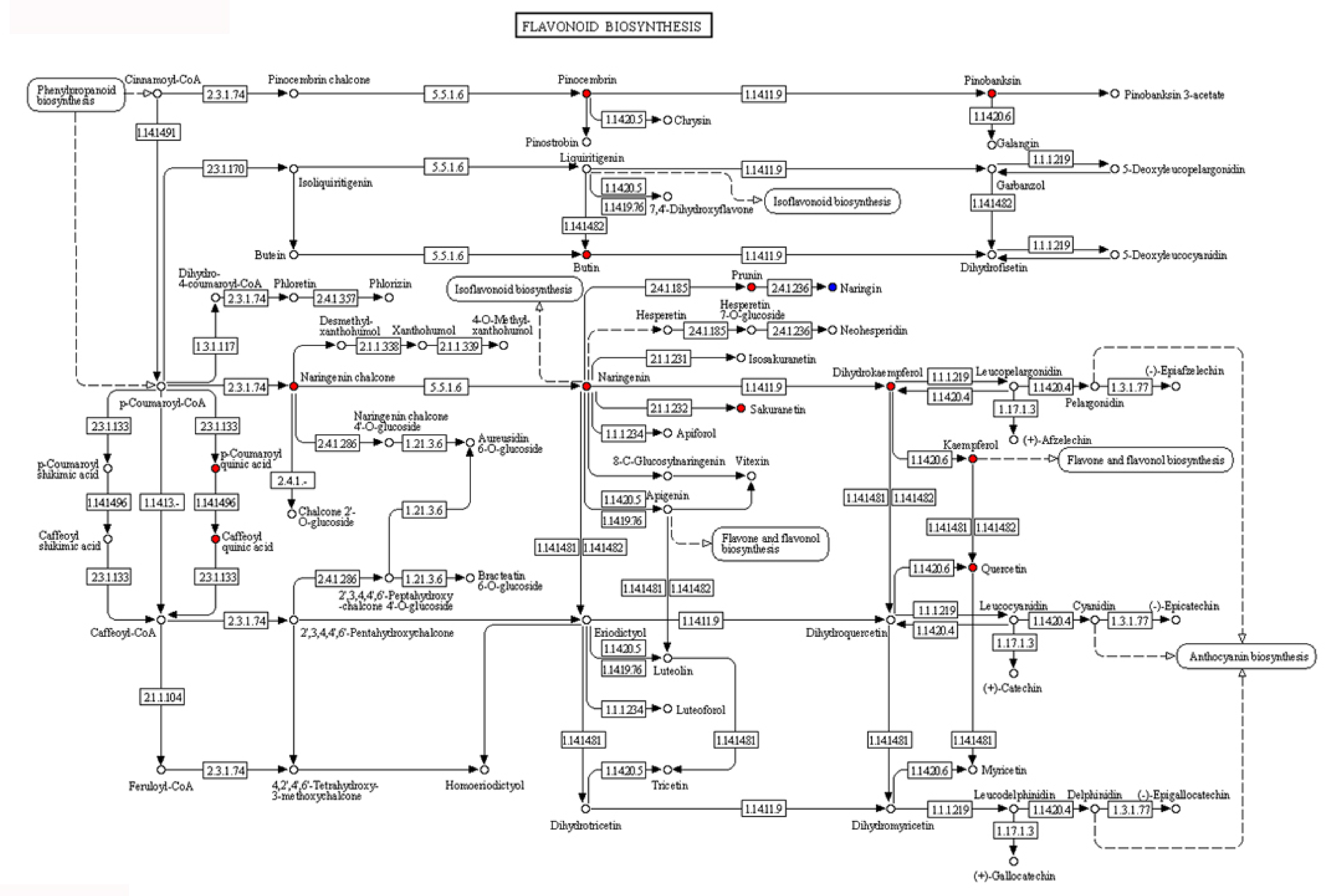
FLAVONOID BIOSYNTHESIS
Phenylpropanoid biosynthesis
Cinnamoyl-CoA
Pinocembrin chalcone
Pinocembrin
Pinobanksin
Pinobanksin 3-acetate
Chrysin
Pinostrobin
Galangin
Isoliquiritigenin
Liquiritigenin
7,4'-Dihydroxyflavone
Isoflavonoid biosynthesis
5-Deoxyleucopelargonidin
Garbanzol
Butein
Butin
Dihydrofisetin
5-Deoxyleucocyanidin
Dihydro-4-coumaroyl-CoA
Phloretin
Phlorizin
Desmethylxanthohumol
Xanthohumol
4-O-Methylxanthohumol
Naringin
Prunin
Hesperetin
Hesperetin 7-O-glucoside
Neohesperidin
Isosakuranetin
Sakuranetin
Naringenin
Apiforol
8-C-Glucosylnaringenin
Vitexin
Apigenin
Flavone and flavonol biosynthesis
p-Coumaroyl-CoA
Naringenin chalcone
Naringenin chalcone 4'-O-glucoside
Isosalipurposide
p-Coumaroyl shikimic acid
p-Coumaroyl quinic acid
Chalcone 2'-O-glucoside
Caffeoyl shikimic acid
Caffeoyl quinic acid
Butein
Bracteatin 6-O-glucoside
2',3,4,4',6'-Pentahydroxychalcone
2',3,4,4',6'-Pentahydroxychalcone 4'-O-glucoside
Caffeoyl-CoA
Feruloyl-CoA
4,2',4',6'-Tetrahydroxy-3-methoxychalcone
Homoeriodictyol
Eriodictyol
Luteolin
Luteoforol
Tricetin
Dihydrotricetin
Dihydrokaempferol
Leucopelargonidin
Pelargonidin
(-)-Epiafzelechin
(+)-Afzelechin
Kaempferol
Dihydroquercetin
Quercetin
Leucocyanidin
Cyanidin
(-)-Epicatechin
(+)-Catechin
Anthocyanin biosynthesis
Dihydromyricetin
Myricetin
Leucodelphinidin
Delphinidin
(-)-Epigallocatechin
(+)-Gallocatechin

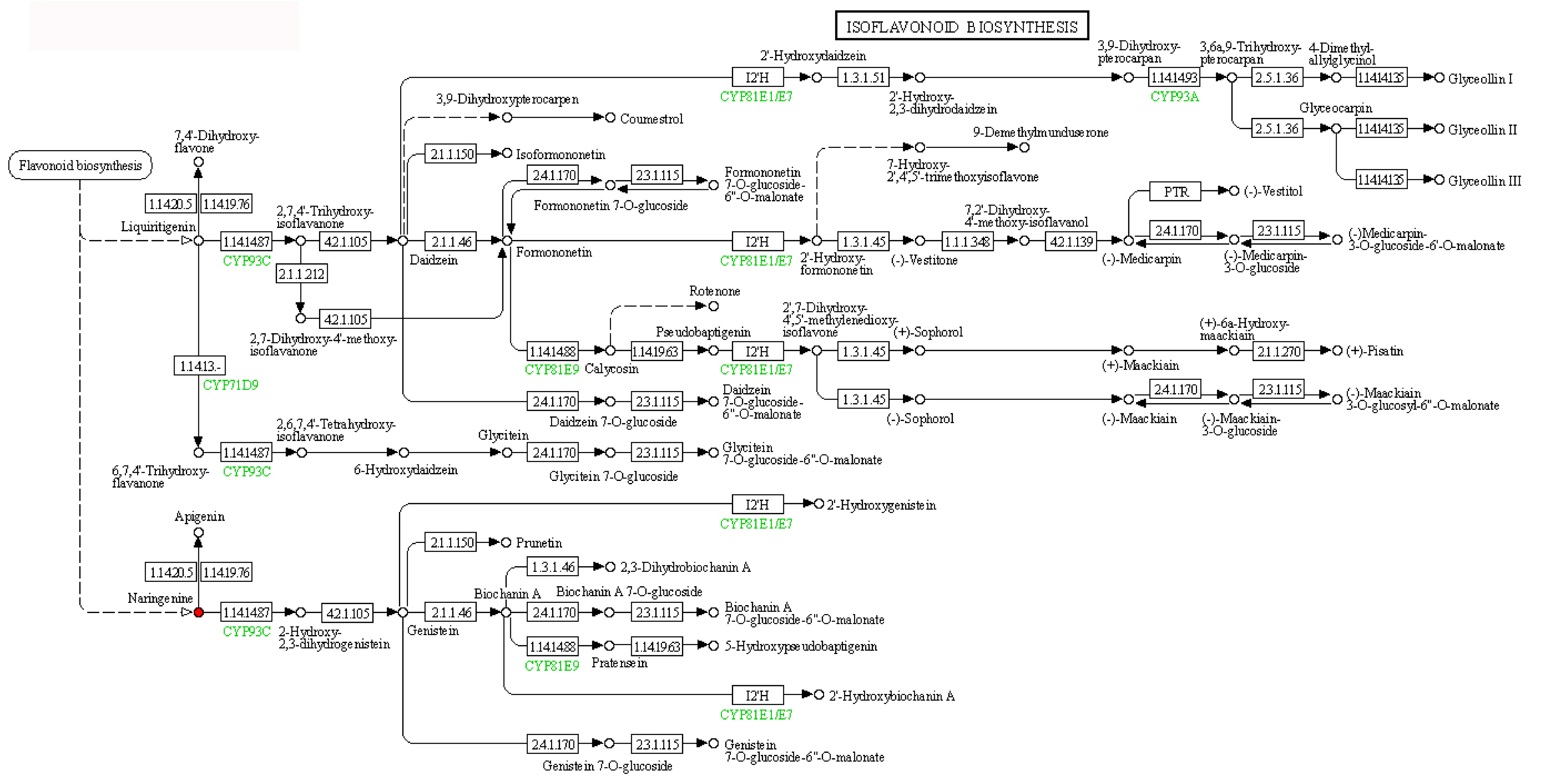
ISOFLAVONOID BIOSYNTHESIS
Flavonoid biosynthesis
7,4'-Dihydroxyflavone
Liquiritigenin
CYP93C
2,7,4'-Trihydroxyisoflavanone
Daidzein
3,9-Dihydroxypterocarpan
Coumestrol
Isoformononetin
Formononetin
Formononetin 7-O-glucoside
Formononetin 7-O-glucoside-6''-O-malonate
2'-Hydroxydaidzein
CYP81E1/E7
2'-Hydroxy-2,3-dihydrodaidzein
3,9-Dihydroxypterocarpan
CYP93A
3,6a,9-Trihydroxypterocarpan
4-Dimethylallylglycinol
Glyceocarpin
Glyceollin I
Glyceollin II
Glyceollin III
9-Demethylmedicarpin
7-Hydroxy-2',4',5'-trimethoxyisoflavone
7,2'-Dihydroxy-4'-methoxy-isoflavanol
PTR
(-)-Vestitol
(-)-Medicarpin
(-)-Medicarpin-3-O-glucoside
(-)-Medicarpin-3-O-glucoside-6'-O-malonate
2'-Hydroxyformononetin
(-)-Vestitone
2,7-Dihydroxy-4'-methoxy-isoflavanone
CYP71D9
Rotenone
Pseudobaptigenin
Calycosin
CYP81E9
2,7-Dihydroxy-4',5'-methylenedioxy-isoflavone
(+)-Sophorol
(-)-Sophorol
(+)-6a-Hydroxymaackiain
(+)-Pisatin
(+)-Maackiain
(-)-Maackiain
(-)-Maackiain-3-O-glucoside
(-)-Maackiain-3-O-glucoside-6''-O-malonate
Daidzein 7-O-glucoside
Daidzein 7-O-glucoside-6''-O-malonate
2,6,7,4'-Tetrahydroxyisoflavanone
6,7,4'-Trihydroxyflavanone
6-Hydroxydaidzein
Glycitein
Glycitein 7-O-glucoside
Glycitein 7-O-glucoside-6''-O-malonate
Apigenin
Naringenine
2-Hydroxy-2,3-dihydrogenistein
Genistein
2'-Hydroxygenistein
Prunetin
2,3-Dihydrobiochanin A
Biochanin A
Biochanin A 7-O-glucoside
Biochanin A 7-O-glucoside-6''-O-malonate
Pratensein
5-Hydroxypseudobaptigenin
2'-Hydroxybiochanin A
Genistein 7-O-glucoside
Genistein 7-O-glucoside-6''-O-malonate

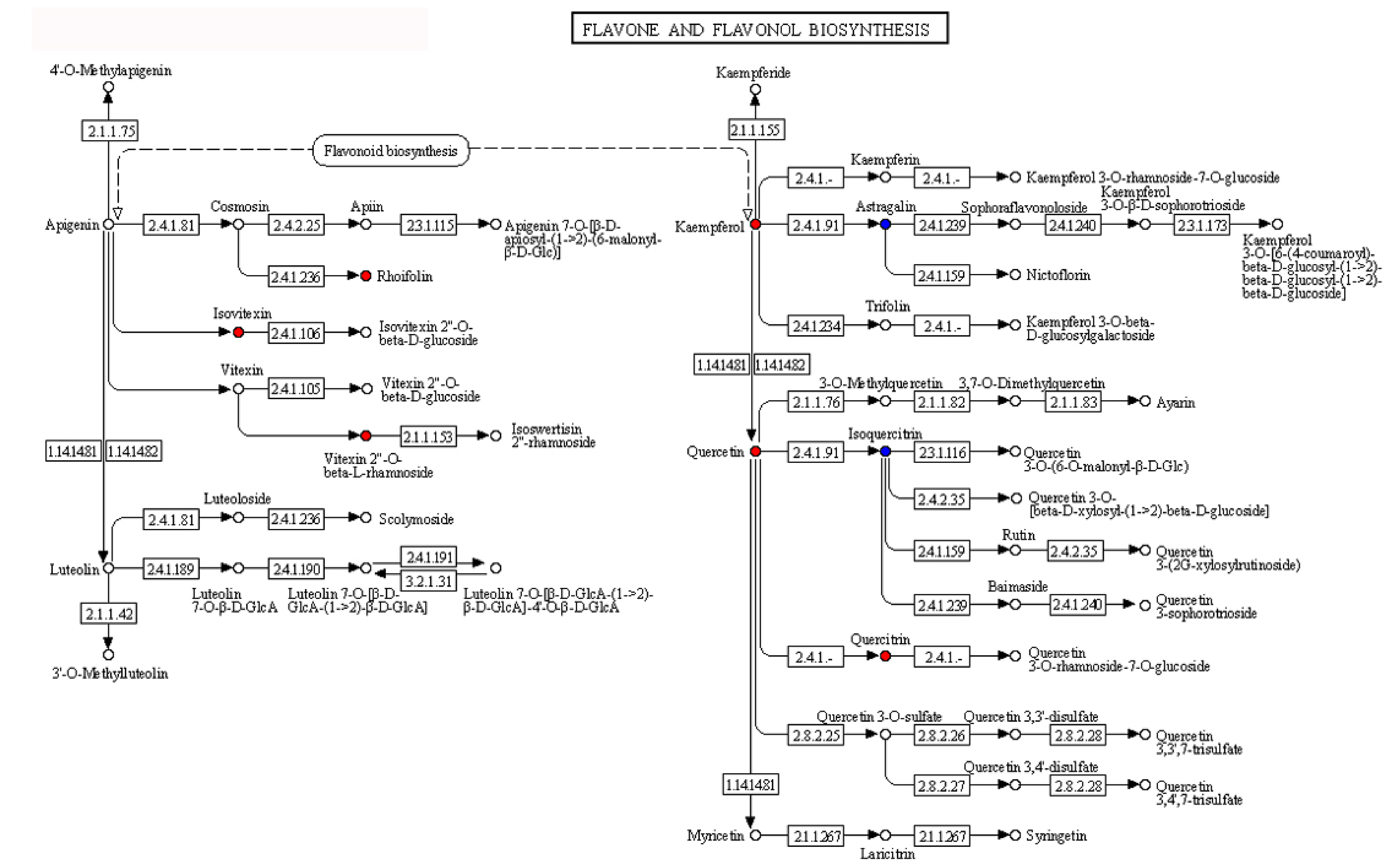
FLAVONE AND FLAVONOL BIOSYNTHESIS
4'-O-Methylapigenin
Flavonoid biosynthesis
Apigenin
Cosmosiin
Apiin
Apigenin 7-O-[β-D-apiosyl-(1->2)-6-malonyl-β-D-Glc]
Rhoifolin
Isovitexin
Isovitexin 2''-O-beta-D-glucoside
Vitexin
Vitexin 2''-O-beta-D-glucoside
Vitexin 2''-O-beta-L-rhamnoside
Isoswertisin 2''-rhamnoside
Luteoloside
Scolymoside
Luteolin
Luteolin 7-O-β-D-GlcA
Luteolin 7-O-[β-D-GlcA-(1->2)-β-D-GlcA]
Luteolin 7-O-[β-D-GlcA-(1->2)-β-D-GlcA]-4'-O-β-D-GlcA
3'-O-Methylluteolin
Kaempferide
Kaempferol
Kaempferin
Kaempferol 3-O-rhamnoside-7-O-rhamnoside
Astragalin
Sophoraflavonoloside
Kaempferol 3-O-sophoroside
Kaempferol 3-O-[6-(4-coumaroyl)-beta-D-glucosyl-(1->2)-beta-D-glucosyl-(1->2)-beta-D-glucoside]
Nicotiflorin
Trifolin
Kaempferol 3-O-beta-D-glucosylgalactoside
3-O-Methylquercetin
3,7-O-Dimethylquercetin
Ayanin
Quercetin
Isoquercitrin
Quercetin 3-O-(6-O-malonyl-β-D-Glc)
Quercetin 3-O-[beta-D-xylosyl-(1->2)-beta-D-glucoside]
Rutin
Quercetin 3-(2G-xylosylrutinoside)
Baimaside
Quercetin 3-O-sophoroside
Quercitrin
Quercetin 3-O-rhamnoside-7-O-glucoside
Quercetin 3-O-sulfate
Quercetin 3,3'-disulfate
Quercetin 3,3',7-trisulfate
Quercetin 3,4'-disulfate
Quercetin 3,4',7-trisulfate
Myricetin
Laricitrin
Syringetin
